# Supplementary material for: Rhodium(II)-catalyzed multicomponent assembly of α,α,α-trisubstituted esters via formal insertion of O–C(sp3)–C(sp2) into C–C bonds
Source: Nat Commun. 2020 Aug 24;11:4219. doi: 10.1038/s41467-020-17990-z (PMC7445163; doi:10.1038/s41467-020-17990-z)
Supplement: Supplementary file 1 — Supplementary Information [file 41467_2020_17990_MOESM1_ESM.pdf]

Supplementary Information

Rhodium(II)-catalyzed multicomponent assembly of α,α,α-trisubstituted esters via formal insertion of O–C(sp^3)–C(sp^2) into C–C bonds

Ba et al

Supplementary Methods

General Information

All the solvents were used without further purification. The other commercial chemicals were used without further purification. All reactions were performed under an inert atmosphere of nitrogen in flame-dried glassware, unless otherwise stated. Analytical thin-layer chromatography was performed on 0.25 mm silica gel, 60-F254. Visualization was carried out with UV light and Vogel's permanganate. Preparative TLC was performed on 1.0 mm silica gel. ^{1}H NMR spectra were recorded on a Bruker Avance III instrument (500 MHz). ^{13}C NMR spectra were recorded on a Bruker Avance III instrument (126 MHz) and were fully decoupled by broad band proton decoupling. High-resolution mass spectra (HRMS) were recorded on an Agilent 1290 mass spectrometer using ESI-TOF (electrospray ionization time-of-flight). NMR spectra were recorded in $CDCl_3$. ^{1}H NMR spectra were referenced to residual $CHCl_3$ at 7.26 ppm, and ^{13}C NMR spectra were referenced to the central peak of $CDCl_3$ at 77.0 ppm. Chemical shifts (δ) are reported in ppm, and coupling constants (*J*) are reported in hertz (Hz). Multiplicities are reported using the following abbreviations: s = singlet, d = doublet, t = triplet, q = quartet, m = multiplet.

General Procedure for the Synthesis of 1-Aryl-1,3-butadione 1

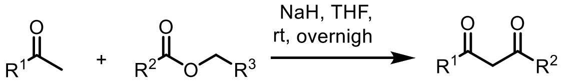

1

Following a modified literature procedure,[1,2] the corresponding ester (20 mmol, 2 equiv) and NaH (1.2 g, 28 mmol, 2.8 equiv, 60% in mineral oil) were dissolved in dry THF (20 mL) in oven-dried glassware under N_2. A solution of the corresponding ketone (10 mmol) in dry THF (20 mL) was added slowly, and the reaction mixture was heated to reflux and stirred overnight. The reaction mixture was quenched with aqueous NH_4Cl (25 mL, 1 M) and dichloromethane (50 mL). The aqueous layer was extracted with dichloromethane (2 x 20 mL) and the combined organic phase washed with brine (20 mL). The organic layerwas dried $MgSO_4$ and the solvent removed under reduced pressure to yield the crude product. The crude material was purified by column chromatography (ethyl acetate / petroleum ether = 1 / 50) to give 1,3-diones **1**.

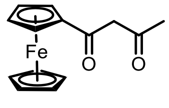

1ae

1-(ferrocenyl)-1,3-butadione (**1ae**) as a red solid (2.56 g, 95% yield). ^{1}H NMR(500 MHz, Chloroform-d) δ 5.72 (s, 1H), 4.78 – 4.76 (m, 2H), 4.50 (t, *J*=1.9, 2H), 4.22 (s, 2H), 4.19 (s, 5H), 2.08 (s, 3H); ^{13}C NMR (126 MHz, Chloroform-d) δ 192.6, 186.2, 97.0, 72.0, 70.3, 70.1, 69.8, 68.6, 24.2. HRMS (EI) for $C_{14}H_{14}FeNaO_2$ $(M + Na)^+$. Calculated: 293.0235, found: 293.0234.

General Procedure for the Synthesis of 2

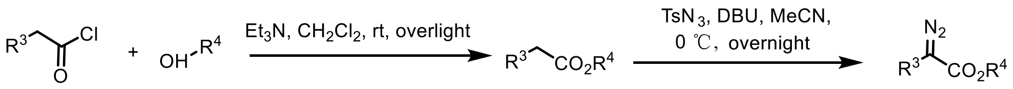

2

Following a modified literature procedure.[3,4] To a mixture of ester (10 mmol) and tosyl azide (2.96 g, 15 mmol) in anhydrous MeCN (15 mL), 1,8-diazabicyclo[5.4.0]undec-7-ene (DBU) (2.24 mL, 2.28 g, 15 mmol) was added. The reaction mixture was stirred at room temperature overnight. Upon complete consumption of the starting materials, the reaction mixture was quenched with saturated aqueous solution of NH_4Cl (5 mL), extracted with dichloromethane (3 × 30 mL), washed with brine (3 × 10 mL), dried over $MgSO_4$, and concentrated under reduced pressure to give the crude product. The residue was purified by flash chromatography (ethyl acetate / petroleum ether = 1 : 50) to afford the α-diazoester.

Procedure for the Preparation of 3 and 4

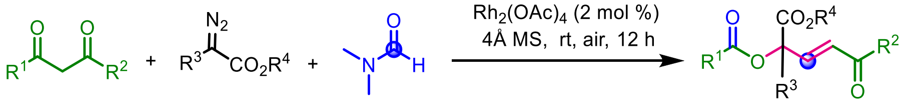

1 **2** **3** and **4**

General procedure for the synthesis of **3** (with **3a** as an example) : A screw capped reaction vial was charged with 1,3-diphenyl-1,3-propanedione (**1a**) (44.8 mg, 0.2 mmol, 2 equiv), methyl 2-(4-(*tert*-butyl)phenyl)-2-diazoacetate (**2a**) (23.2 mg, 0.1 mmol, 1 equiv) 4Å MS (50 mg), and DMF (0.5 mL), followed by the addition of $Rh_2(OAc)_4$ (0.8 mg, 0.002 mmol, 2 mol%). The resulting mixture was stirred at room temperature for 12 h, until TLC showed the complete consumption of **2a**. After the reaction was completed, the reaction mixture was evaporated under reduced pressure to leave a crude mixture, which was purified by column chromatography on silica gel (eluting with ethyl acetate/petroleum = 1:10) to afford (*E*)-2-(4-(*tert*-butyl)phenyl)-1-methoxy-1,5-dioxo-5-phenylpent-3-en-2-yl benzoate **(3a)**, as a colorless crystal (38.8 mg, 85% yield). M. p. 124 - 125°C. ^{1}H NMR(500 MHz, Chloroform-d) δ 8.21 (dd, *J*=8.3, 1.6, 2H), 7.88 – 7.78 (m, 3H), 7.70 – 7.61 (m, 1H), 7.61 – 7.49 (m, 5H), 7.48 – 7.37 (m, 4H), 6.92 (dd, *J*=16.0, 4.2, 1H), 3.80 (s, 3H), 1.34 (d, *J*=1.5, 9H); ^{13}C NMR (126 MHz, Chloroform-d) δ 190.8, 169.4, 164.9, 152.0, 145.1, 137.5, 134.0, 133.7, 132.9, 130.0, 129.3, 128.8, 128.7, 128.5, 126.3, 125.9, 125.6, 82.5, 53.2, 34.6, 31.2; HRMS (EI) for $C_{29}H_{28}NaO_5$ $(M + Na)^+$. Calculated: 479.1829, found: 479.1831.

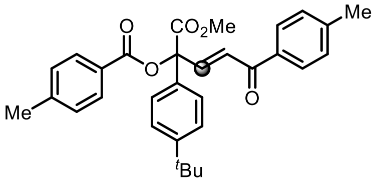

3b

(*E*)-2-(4-(*tert*-butyl)phenyl)-1-methoxy-1,5-dioxo-5-(p-tolyl)pent-3-en-2-yl 4-methylbenzoate (**3b**) (39.2 mg, 81%) as white solid. M. p. 143°C. Ethyl acetate : petroleum ether = 1 : 5. ^{1}H NMR(500 MHz, Chloroform-d) δ 8.13 – 8.05 (m, 2H), 7.80 (d, *J*=16.0, 1H), 7.77 – 7.70 (m, 2H), 7.60 – 7.53 (m, 2H), 7. 47 – 7.41 (m, 2H), 7.32 (d, *J*=8.0, 2H), 7.21 (d, *J*=8.0, 2H), 6.89 (d, *J*=15.9, 1H), 3.78 (s, 3H), 2.46 (s, 3 H), 2.38 (s, 3H), 1.33 (s, 9H); ^{13}C NMR (126 MHz, Chloroform-d) δ 190.4, 169.5, 164.9, 151.9, 144.8, 144.6, 143.7, 135.0, 134.2, 130.1, 129.3, 129.2, 128.9, 126.6, 126.3, 125.8, 125.6, 82.4, 53.1, 34.6, 31.2, 21.8, 21.6; HRMS (EI) for$C_{31}H_{32}NaO_5$ (M + Na)$^+$. Calculated: 507.2142, found: 507.2140.

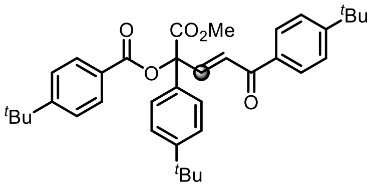

3c

(*E*)-2,5-bis(4-(*tert*-butyl)phenyl)-1-methoxy-1,5-dioxopent-3-en-2-yl 4-(*tert*-butyl)benzoate (**3c**) (3 4.3 mg, 60%) as light pink solid. M. p. 119 - 120°C. Ethyl acetate : petroleum ether = 1 : 10. ^{1}H NMR (500 MHz, Chloroform-d) 8.16 – 8.11 (m, 2H), 7.85 – 7.76 (m, 3H), 7.58 – 7.52 (m, 4H), 7.43 (dq, *J*=9. 0, 2.4, 4H), 6.88 (d, *J*=15.9, 1H), 3.77 (s, 3H), 1.37 (s, 9H), 1.33 (s, 9H), 1.31 (s, 9H); ^{13}C NMR (126 M Hz, Chloroform-d) 190.5, 169.6, 164.9, 157.6, 156.6, 151.9, 144.8, 134.8, 134.2, 129.9, 128.8, 126.5, 12 6.5, 126.4, 125.8, 125.6, 125.5, 82.3, 53.1, 35.2, 35.1, 34.6, 31.2, 31.1, 31.1; HRMS (EI) for $C_{37}H_{44}NaO_5$ (M + Na)$^+$. Calculated: 591.3081, found: 591.3082.

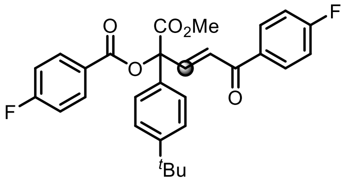

3d

(E)-2-(4-(*tert*-butyl)phenyl)-5-(4-fluorophenyl)-1-methoxy-1,5-dioxopent-3-en-2-yl 4-fluorobenzo ate (**3d**) (44.0 mg, 89%) as gray-green oily liquid. Ethyl acetate : petroleum ether = 1 : 10. ^{1}H NMR (50 0 MHz, Chloroform-d) 8.21 (dd, *J*=8.7, 5.5, 2H), 7.90 – 7.84 (m, 2H), 7.78 (d, *J*=15.9, 1H), 7.56 – 7.51 (m, 2H), 7.48 – 7.43 (m, 2H), 7.23 – 7.16 (m, 2H), 7.14 – 7.07 (m, 2H), 6.87 (d, *J*=16.0, 1H), 3.79 (s, 3 H), 1.33 (s, 9H); ^{13}C NMR (126 MHz, Chloroform-d) δ 189.2, 169.3, 166.3 (d, *J* = 255.7 Hz), 165.7 (d, *J* = 254.8 Hz), 164.0, 152.2, 145.1, 133.8, 132.7 (d, *J* = 9.5 Hz), 131.6 (d, *J* = 9.4 Hz), 131.4 (d, *J* = 9.4 Hz), 126.1, 125.9, 125.6, 116.3 (d, *J* = 22.1 Hz), 115.9 (d, *J* = 22.1 Hz), 115.7 (d, *J* = 21.8 Hz), 82.6, 53. 3, 34.7, 31.2; HRMS (EI) for $C_{29}H_{26}F_2NaO_5$ (M + Na)$^+$. Calculated: 551.1641, found: 551.1644.

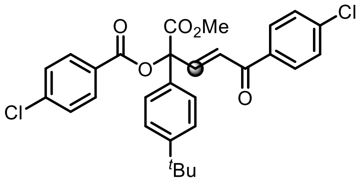

3e

(*E*)-2-(4-(*tert*-butyl)phenyl)-5-(4-chlorophenyl)-1-methoxy-1,5-dioxopent-3-en-2-yl 4-chlorobenzoate (**3e**) (51.5 mg, 97%) as white solid. M. p. 167-168°C. Ethyl acetate : petroleum ether = 1 : 10. ^{1}H NMR (500 MHz, Chloroform-d) δ 8.15 – 8.09 (m, 2H), 7.83 – 7.74 (m, 3H), 7.55 – 7.48 (m, 4H), 7.46 (d, *J*=8.6, 2H), 7.43 – 7.38 (m, 2H), 6.86 (d, *J*=15.9, 1H), 3.79 (s, 3H), 1.33 (s, 9H); ^{13}C NMR (126 MHz, Chloroform-d) δ 189.5, 169.1, 164.1, 152.3, 145.3, 140.5, 139.5, 135.7, 133.7, 131.4, 130.2, 129.1, 128.9, 127.6, 126.0, 125.9, 125.6, 82.7, 53.3, 34.7, 31.2; HRMS (ESI-TOF) for $C_{29}H_{26}Cl_2NaO_5$ $(M + Na)^+$. Calculated: 547.1050, found: 547.1052.

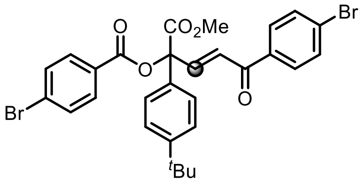

3f

(*E*)-5-(4-bromophenyl)-2-(4-(*tert*-butyl)phenyl)-1-methoxy-1,5-dioxopent-3-en-2-yl 4-bromobenzoate (**3f**) (56.5 mg, 92%) as white solid. M. p. 154-155°C. Ethyl acetate : petroleum ether = 1 : 10. ^{1}H NMR (500 MHz, Chloroform-d) δ 8.08 – 8.00 (m, 2H), 7.79 (d, *J*=15.9, 1H), 7.71 – 7.65 (m, 4H), 7.60 – 7.55 (m, 2H), 7.55 – 7.51 (m, 2H), 7.48 – 7.43 (m, 2H), 6.85 (d, *J*=15.9, 1H), 3.79 (s, 3H), 1.33 (s, 9H); ^{13}C NMR (126 MHz, Chloroform-d) δ 189.5, 169.1, 164.1, 152.3, 145.3, 140.5, 139.5, 135.7, 133.7, 131.4, 130.2, 129.1, 128.9, 127.6, 126.0, 125.9, 125.6, 82.7, 53.3, 34.7, 31.2; HRMS (ESI-TOF) for $C_{29}H_{26}Br_2NaO_5$ $(M + Na)^+$. Calculated: 635.0039, found: 635.0040.

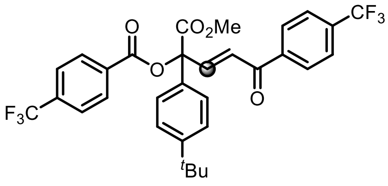

3g

(*E*)-2-(4-(*tert*-butyl)phenyl)-1-methoxy-1,5-dioxo-5-(4-(trifluoromethyl)phenyl)pent-3-en-2-yl 4-(trifluoromethyl)benzoate **(3g)** (50.9 mg, 86%) as white solid. M. p. 172 - 173°C. Ethyl acetate : petroleum ether = 1 : 10. ^{1}H NMR (500 MHz, Chloroform-d) δ 8.31 (d, *J*=8.0, 2H), 7.93 (d, *J*=8.1, 2H), 7.84 (d, *J*=15.9, 1H), 7.80 (d, *J*=8.2, 2H), 7.71 (d, *J*=8.1, 2H), 7.59 – 7.51 (m, 2H), 7.51 – 7.44 (m, 2H), 6.89 (d,

J=15.9, 1H), 3.81 (s, 3H), 1.34 (s, 9H); ^{13}C NMR (126 MHz, Chloroform-d) δ 189.9, 168.9, 163.8, 152.5, 145.9, 140.1, 135.4 (q, *J* = 32.8 Hz), 134.3 (q, *J* = 32.7 Hz), 133.4, 132.4, 130.4, 129.1, 126.3, 126.0, 125.8 (q, J=3.8), 125.7 (q, J=3.7), 125.6, 123.5 (q, *J* = 272.8 Hz) 123.3 (q, *J* = 272.8 Hz), 83.0, 53.4, 34.7, 31; HRMS (ESI-TOF) for $C_{31}H_{26}F_6NaO_5$ (M + Na)$^+$. Calculated: 615.1577, found: 615.1577.

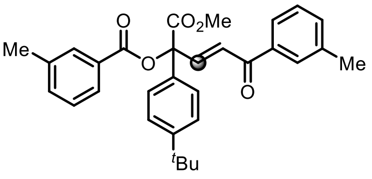

3h

(*E*)-2-(4-(*tert*-butyl)phenyl)-1-methoxy-1,5-dioxo-5-(m-tolyl)pent-3-en-2-yl 3-methylbenzoate **(3h)** (39.2.9 mg, 81%) as white solid. M. p. 143-144°C. Ethyl acetate : petroleum ether = 1 : 10. ^{1}H NMR (500 MHz, Chloroform-d) δ 8.04 – 7.98 (m, 2H), 7.80 (d, *J*=15.9, 1H), 7.65 – 7.54 (m, 4H), 7.48 – 7.40 (m, 4H), 7.32 (dd, *J*=15.8, 7.7, 2H), 6.89 (d, *J*=15.9, 1H), 3.79 (s, 3H), 2.45 (s, 3H), 2.35 (s, 3H), 1.33 (s, 9H); ^{13}C NMR (126 MHz, Chloroform-d) δ 191.1, 169.5, 165.1, 152.0, 145.0, 138.5, 138.3, 137.5, 134.5, 134.1, 133.6, 130.5, 129.3, 129.2, 128.5, 128.4, 127.1, 126.5, 126.0, 125.8, 125.6, 82.5, 53.1, 34.6, 31.2, 21.3, 21.3; HRMS (ESI-TOF) for $C_{31}H_{32}NaO_5$ (M + Na)$^+$. Calculated: 507.2142, found: 507.2143.

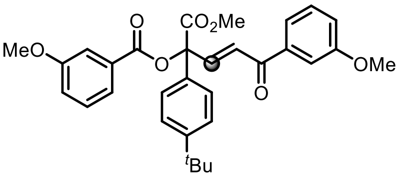

3i

(*E*)-2-(4-(*tert*-butyl)phenyl)-1-methoxy-5-(3-methoxyphenyl)-1,5-dioxopent-3-en-2-yl 3-methoxybenzoate **(3i)** (32.0 mg, 62%) as white solid. M. p. 145 - 146°C. Ethyl acetate : petroleum ether = 1 : 10. ^{1}H NMR (500 MHz, Chloroform-d) δ 7.85 – 7.79 (m, 2H), 7.68 (dd, *J*=2.7, 1.5, 1H), 7.59 – 7.53 (m, 2H), 7.45 (d, *J*=2.6, 1H), 7.44 – 7.29 (m, 5H), 7.20 (dd, *J*=2.7, 1.0, 1H), 7.11 – 7.05 (m, 1H), 6.87 (d, *J*=15.9, 1H), 3.88 (s, 3H), 3.80 (s, 3H), 3.79 (s, 3H), 1.33 (s, 9H); ^{13}C NMR (126 MHz, Chloroform-d) δ 190.6, 169.4, 164.8, 159.7, 159.7, 152.1, 145.1, 138.8, 134.0, 130.5, 129.7, 129.5, 126.3, 125.9, 125.6, 122.3, 121.4, 120.2, 119.8, 114.6, 112.8, 82.6, 55.5, 55.4, 53.2, 34.6, 31.2; HRMS (ESI-TOF) for $C_{31}H_{32}NaO_7$ (M + Na)$^+$. Calculated: 539.2040, found: 539.2039.

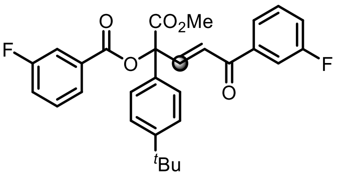

3j

(***E***)-2-(4-(*tert*-butyl)phenyl)-5-(3-fluorophenyl)-1-methoxy-1,5-dioxopent-3-en-2-yl 3-fluorobenzoate (**3j**) (35.3 mg, 71%) as white solid. M. p. 117°C. Ethyl acetate : petroleum ether = 1 : 10. ^{1}H NMR (500 MHz, Chloroform-d) δ 7.99 (dt, *J*=7.8, 1.3, 1H), 7.86 (ddd, *J*=9.1, 2.6, 1.5, 1H), 7.83 (d, *J*=15.9, 1H), 7.61 (dt, *J*=7.7, 1.3, 1H), 7.57 – 7.49 (m, 4H), 7.49 – 7.44 (m, 2H), 7.44 – 7.33 (m, 2H), 7.26 – 7.21 (m, 1H), 6.88 (d, *J*=15.9, 1H), 3.80 (s, 3H), 1.34 (s, 9H). ^{13}C NMR (126 MHz, Chloroform-d) δ 189.3, 169.0, 163.9, 162.8 (d, *J* = 248.2 Hz), 162.7 (d, *J* = 248.2 Hz), 152.3, 145.5, 139.5 (d, *J* = 6.1 Hz), 133.6, 131.3, 121.0 (d, *J* = 20.9 Hz), 120.0 (d, *J* = 21.3 Hz), 126.0 (d, *J*=6.7), 125.9, 125.8 (d, *J*=3.4), 125.6, 124.5 (d, *J*=3.0), 121.0 (d, *J* = 20.9 Hz), 120.0 (d, *J* = 21.3 Hz), 116.9 (d, *J* = 23.0 Hz), 115.5 (d, *J* = 22.4 Hz), 82.8, 53.3, 34.7, 31.2; HRMS (ESI-TOF) for $C_{29}H_{26}F_2NaO_5$ $(M + Na)^+$. Calculated: 551.1641, found: 551.1643.

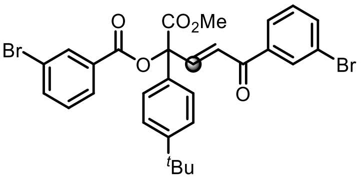

3k

(***E***)-5-(3-bromophenyl)-2-(4-(*tert*-butyl)phenyl)-1-methoxy-1,5-dioxopent-3-en-2-yl 3-bromobenz oate (**3k**) (49.7 mg, 81%) as white solid. M. p. 161 - 162°C. Ethyl acetate : petroleum ether = 1 : 10. ^{1}H NMR (500 MHz, Chloroform-d) δ 8.15 – 8.09 (m, 1H), 7.95 (t, *J*=1.8, 1H), 7.83 – 7.72 (m, 3H), 7.66 (d dd, *J*=8.0, 2.0, 1.0, 1H), 7.56 – 7.52 (m, 2H), 7.49 – 7.45 (m, 2H), 7.41 (t, *J*=7.9, 1H), 7.31 (t, *J*=7.9, 1 H), 6.85 (d, *J*=15.9, 1H), 3.80 (s, 3H), 1.34 (s, 9H); ^{13}C NMR (126 MHz, Chloroform-d) δ 189.2, 169.0, 163.7, 152.3, 145.6, 139.1, 136.8, 135.8, 133.5, 132.9, 131.8, 131.1, 130.2, 130.1, 128.6, 127.3, 126.0, 126.0, 125.6, 122.9, 122.8, 82.8, 53.3, 34.7, 31.2; HRMS (ESI-TOF) for $C_{29}H_{27}Br_2NaO_5$ $(M + Na)^+$. Cal culated: 635.0039, found: 635.0041.

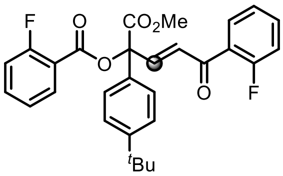

3l

(***E***)-2-(4-(*tert*-butyl)phenyl)-5-(2-fluorophenyl)-1-methoxy-1,5-dioxopent-3-en-2-yl 2-fluorobenzo ate (**3l**) (45.3 mg, 81%) as white solid. M. p. 87 - 89°C. Ethyl acetate : petroleum ether = 1 : 10. ^{1}H NM R (500 MHz, Chloroform-d) δ 8.07 (td, *J*=7.5, 1.9, 1H), 7.83 (dd, *J*=15.8, 1.5, 1H), 7.72 (td, *J*=7.5, 1.8, 1H), 7.65 – 7.55 (m, 3H), 7.52 – 7.40 (m, 3H), 7.31 – 7.18 (m, 3H), 7.06 (ddd, *J*=10.8, 8.4, 1.0, 1H), 6.8 8 (dd, *J*=15.8, 2.7, 1H), 3.80 (s, 3H), 1.32 (s, 9H); ^{13}C NMR (126 MHz, Chloroform-d) δ 188.7, 169.1, 162.8 (d, *J* = 3.5 Hz), 162.2 (d, *J* = 261.0 Hz), 161.3 (d, *J* = 254.4 Hz), 151.9, 145.0, 135.3 (d, *J* = 9.0 H

z), 134.2 (d, J = 8.7 Hz), 133.6, 132.6, 131.1 (d, J = 2.4 Hz), 129.1 (d, J = 6.4 Hz), 126.3 (d, J = 12.9 Hz), 125.8, 125.6, 124.4 (d, J = 3.5 Hz), 124.2 (d, J = 3.9 Hz), 117.8 (d, J = 10.1 Hz), 117.2 (d, J = 22.4 Hz), 116.5 (d, J = 22.9 Hz), 83.0, 53.2, 34.6, 31.2; HRMS (ESI-TOF) for $C_{29}H_{26}F_2NaO_5$ $(M + Na)^+$. Calculated: 551.1641, found: 551.1644.

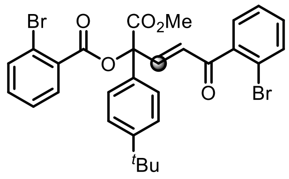

3m

(*E*)-5-(2-bromophenyl)-2-(4-(*tert*-butyl)phenyl)-1-methoxy-1,5-dioxopent-3-en-2-yl 2-bromobenzoate (**3m**) (43.1 mg, 70%) as off-white solid. M. p. 155 - 156°C. Ethyl acetate : petroleum ether = 1 : 10. ^{1}H NMR (500 MHz, Chloroform-d) δ 7.97 (d, J=7.4, 1H), 7.71 (dd, J=7.6, 1.5, 1H), 7.63 (d, J=16.2, 1H), 7.59 (dd, J=8.0, 1.0, 1H), 7.53 – 7.48 (m, 2H), 7.45 – 7.36 (m, 6H), 7.30 (ddd, J=7.9, 7.1, 2.1, 1H), 6.66 (d, J=16.2, 1H), 3.79 (s, 3H), 1.31 (s, 9H); ^{13}C NMR (126 MHz, Chloroform-d) δ 193.9, 169.0, 164.4, 152.2, 146.7, 140.2, 134.6, 133.5, 133.2, 133.2, 131.7, 131.7, 131.1, 130.1, 129.6, 127.4, 127.3, 125.8, 125.7, 122.0, 119.6, 83.1, 53.3, 34.6, 31.2; HRMS (ESI-TOF) for $C_{29}H_{27}Br_2NaO_5$ $(M + Na)^+$. Calculated: 635.0039, found: 635.0042.

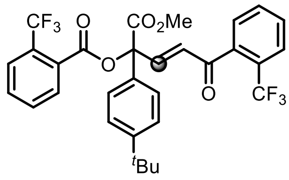

3n

(*E*)-2-(4-(*tert*-butyl)phenyl)-1-methoxy-1,5-dioxo-5-(2-(trifluoromethyl)phenyl)pent-3-en-2-yl 2-(trifluoromethyl)benzoate (**3n**) (48.5 mg, 82%) as White solid. M. p. 145 - 147°C. Ethyl acetate : petroleum ether = 1 : 10. ^{1}H NMR (500 MHz, Chloroform-d) δ 8.0 – 8.0 (m, 1H), 7.9 – 7.8 (m, 1H), 7.8 (d, J=7.7, 1H), 7.7 (dd, J=6.0, 3.0, 2H), 7.7 – 7.6 (m, 2H), 7.5 (d, J=16.4, 1H), 7.5 – 7.4 (m, 5H), 6.6 (d, J=16.4, 1H), 3.8 (s, 3H), 1.3 (s, 9H); ^{13}C NMR (126 MHz, Chloroform-d) δ 194.7, 168.8, 164.7, 152.3, 148.3, 137.7 (d, J=2.2), 132.7, 131.9 (d, J=2.8), 131.7, 131.6, 130.5, 130.1, 129.8, 129.1 (q, J = 32.8 Hz), 128.2, 127.9 (q, J = 32.4 Hz), 127.0 (q, J = 5.5 Hz), 126.7 (q, J = 4.7 Hz), 126.3, 125.8, 125.6, 123.5 (q, J = 274.0 Hz), 123.1 (q, J = 273.7 Hz), 83.07, 53.3, 34.6, 31.2; HRMS (ESI-TOF) for HRMS (ESI-TOF) for $C_{31}H_{26}F_6NaO_5$ $(M + Na)^+$. Calculated: 615.1577, found: 615.1573.

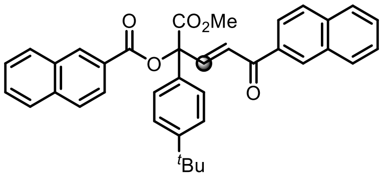

3o

(*E*)-2-(4-(*tert*-butyl)phenyl)-1-methoxy-5-(naphthalen-2-yl)-1,5-dioxopent-3-en-2-yl 2-naphthoate (**3o**) (50.3 mg, 90%) as light brown solid. M. p. 138 - 139°C. Ethyl acetate : petroleum ether = 1 : 10. ^{1}H NMR (500 MHz, Chloroform-d) δ 8.84 – 8.76 (m, 1H), 8.33 – 8.28 (m, 1H), 8.22 (dd, *J*=8.6, 1.7, 1H), 8.03 (d, *J*=8.1, 1H), 7.98 (d, *J*=8.6, 1H), 7.96 – 7.91 (m, 3H), 7.87 – 7.80 (m, 3H), 7.69 – 7.63 (m, 3H), 7.62 – 7.53 (m, 2H), 7.53 – 7.46 (m, 3H), 7.12 (d, *J*=15.9, 1H), 3.83 (s, 3H), 1.36 (s, 9H); ^{13}C NMR (12 6 MHz, Chloroform-d) δ 190.6, 169.5, 165.2, 152.1, 145.1, 135.9, 135.5, 134.8, 134.1, 132.5, 132.4, 13 1.8, 130.6, 129.6, 129.5, 128.8, 128.6, 128.5, 128.4, 127.9, 127.7, 126.9, 126.7, 126.5, 126.3, 125.9, 125. 7, 125.2, 124.5, 82.7, 53.3, 34.7, 31.2; HRMS (ESI-TOF) for $C_{37}H_{32}NaO_5$ (M + Na)$^+$. Calculated: 579.2 142, found: 579.2143.

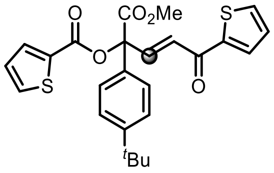

3p

(*E*)-2-(4-(*tert*-butyl)phenyl)-1-methoxy-1,5-dioxo-5-(thiophen-2-yl)pent-3-en-2-yl thiophene-2-car boxylate (**3p**) (31.9 mg, 68%) as white solid. M. p. 172 - 173°C. Ethyl acetate : petroleum ether = 1 : 10. ^{1}H NMR (500 MHz, Chloroform-d) δ 7.98 (dd, *J*=3.7, 1.3, 1H), 7.87 (d, *J*=15.7, 1H), 7.68 (dd, *J*=4.9, 1. 2, 1H), 7.66 – 7.60 (m, 2H), 7.55 (d, *J*=8.4, 2H), 7.44 (d, *J*=8.6, 2H), 7.19 (d, *J*=1.2, 1H), 7.09 (d, *J*=4.3, 1H), 6.90 (d, *J*=15.7, 1H), 3.80 (s, 3H), 1.32 (s, 9H); ^{13}C NMR (126 MHz, Chloroform-d) δ 182.0, 169. 1, 160.3, 152.0, 144.6, 144.0, 134.7, 134.4, 133.7, 133.5, 132.7, 132.3, 128.2, 128.1, 125.8, 125.8, 125.5, 82.5, 53.3, 34.6, 31.2; HRMS (ESI-TOF) for $C_{25}H_{24}NaO_5S_2$ (M + Na)$^+$. Calculated: 491.0957, found: 4 91.0959.

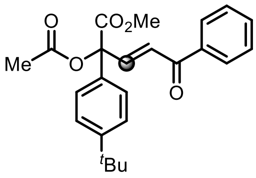

3q

Methyl **(*E*)**-2-acetoxy-2-(4-(*tert*-butyl)phenyl)-5-oxo-5-phenylpent-3-enoate (**3q**) (31.8 mg, 81%) as white solid. M. p. 110 - 111°C. Ethyl acetate : petroleum ether = 1 : 10. ^{1}H NMR (500 MHz, Chlorof

orm-d) δ7.86 (dd, *J*=8.3, 1.4, 2H), 7.69 (d, *J*=16.0, 1H), 7.56 (s, 1H), 7.45 (dd, *J*=16.4, 8.3, 4H), 7.39 (d, *J*=8.7, 2H), 6.85 (d, *J*=15.9, 1H), 3.78 (s, 3H), 2.29 (s, 3H), 1.31 (s, 9H); ^{13}C NMR (126 MHz, Chloroform-d) δ 190.9, 169.4, 169.3, 152.0, 145.6 – 144.4 (m), 137.5, 133.6, 132.9, 128.8, 128.6, 126.3, 125.7, 125.6, 82.2, 53.2, 34.6, 31.2, 21.0; HRMS (ESI-TOF) for $C_{24}H_{26}NaO_5$ (M + Na)$^+$. Calculated: 417.1672, found: 417.1675.

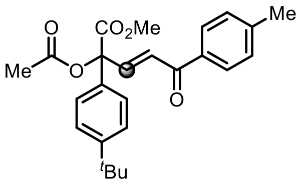

3r

Methyl **(*E*)**-2-acetoxy-2-(4-(*tert*-butyl)phenyl)-5-oxo-5-(p-tolyl)pent-3-enoate (**3r**) (39.2 mg, 96%) as white solid. M. p. 114°C. Ethyl acetate : petroleum ether = 1 : 10. ^{1}H NMR (500 MHz, Chloroform-d) δ 7.82 – 7.74 (m, 2H), 7.67 (d, *J*=15.9, 1H), 7.47 – 7.36 (m, 4H), 7.26 (d, *J*=1.7, 2H), 6.84 (d, *J*=15.9, 1H), 3.78 (s, 3H), 2.41 (s, 3H), 2.28 (s, 3H), 1.31 (s, 9H); ^{13}C NMR (126 MHz, Chloroform-d) δ 190.4, 169.4, 169.3, 152.0, 144.6, 143.8, 134.9, 133.7, 129.3, 128.9, 126.3, 125.7, 125.6, 82.2, 53.2, 34.6, 31.2, 21.7, 21.0; HRMS (ESI-TOF) for $C_{25}H_{28}NaO_5$ (M + Na)$^+$. Calculated: 431.1829, found: 431.1833.

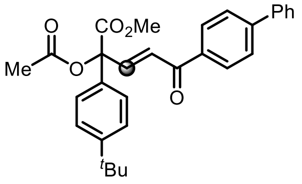

3s

Methyl **(*E*)**-5-([1,1'-biphenyl]-4-yl)-2-acetoxy-2-(4-(*tert*-butyl)phenyl)-5-oxopent-3-enoate (**3s**) (33.2 mg, 71%) as white solid. M. p. 174-175°C. Ethyl acetate : petroleum ether = 1 : 10. ^{1}H NMR (500 MHz, Chloroform-d) δ 7.98 – 7.93 (m, 2H), 7.72 (d, *J*=15.9, 1H), 7.70 – 7.66 (m, 2H), 7.65 – 7.60 (m, 2H), 7.50 – 7.43 (m, 4H), 7.43 – 7.36 (m, 3H), 6.90 (d, *J*=15.9, 1H), 3.79 (s, 3H), 2.30 (s, 3H), 1.31 (s, 9H); ^{13}C NMR (126 MHz, Chloroform-d) δ 190.3, 169.4, 169.3, 152.0, 145.7, 145.0, 139.9, 136.2, 133.6, 129.4, 128.9, 128.2, 127.3, 126.3, 125.9, 125.7, 125.6, 82.2, 53.2, 34.6, 31.2, 21.0; HRMS (ESI-TOF) for $C_{30}H_{30}NaO_5$ (M + Na)$^+$. Calculated: 493.198, found: 493.1984.

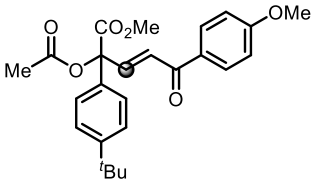

3t

Methyl (***E***)-2-acetoxy-2-(4-(*tert*-butyl)phenyl)-5-(4-methoxyphenyl)-5-oxopent-3-enoate (**3t**) (33.2 mg, 71%) as white solid. M. p. 144 - 146°C. Ethyl acetate : petroleum ether = 1 : 10. ^{1}H NMR (500 MHz, Chloroform-d) δ 7.92 – 7.84 (m, 2H), 7.64 (d, *J*=15.9, 1H), 7.46 – 7.36 (m, 4H), 6.98 – 6.90 (m, 2H), 6.85 (d, *J*=15.9, 1H), 3.87 (s, 3H), 3.78 (s, 3H), 2.28 (s, 3H), 1.31 (s, 9H); ^{13}C NMR (126 MHz, Chloroform-d) δ 189.1, 169.4, 169.3, 163.6, 151.9, 144.0, 133.8, 131.1, 130.5, 126.3, 125.7, 125.6, 113.8, 82.3, 55.5, 53.1, 34.6, 31.2, 21.0; HRMS (ESI-TOF) for $C_{25}H_{28}NaO_6$ (M + Na)$^+$. Calculated: 447.1778, found: 447.1780.

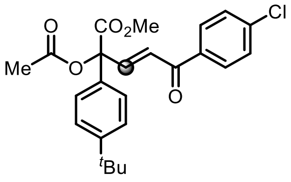

3u

Methyl (***E***)-2-acetoxy-2-(4-(*tert*-butyl)phenyl)-5-(4-chlorophenyl)-5-oxopent-3-enoate (**3u**) (34.1 mg, 80%) as off-white solid. M. p. 104-105°C. Ethyl acetate : petroleum ether = 1 : 10. ^{1}H NMR (500 MHz, Chloroform-d) δ 7.83 – 7.77 (m, 2H), 7.67 (d, *J*=16.0, 1H), 7.47 – 7.37 (m, 6H), 6.81 (d, *J*=15.9, 1H), 3.78 (s, 3H), 2.29 (s, 3H), 1.31 (s, 9H); ^{13}C NMR (126 MHz, Chloroform-d) δ 189.7, 169.4, 169.3, 152.1, 145.6, 139.5, 135.8, 133.5, 130.2, 128.9, 125.9, 125.8, 125.5, 82.1, 53.2, 34.6, 31.2, 21.0; HRMS (ESI-TOF) for $C_{24}H_{25}ClNaO_5$ (M + Na)$^+$. Calculated: 451.1283, found: 451.1284.

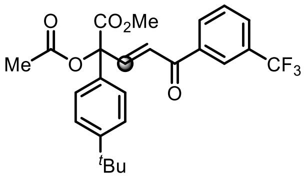

3v

Methyl (***E***)-2-acetoxy-2-(4-(*tert*-butyl)phenyl)-5-oxo-5-(3-(trifluoromethyl)phenyl)pent-3-enoate (**3v**) (32.8 mg, 71%) as light yellow oily liquid. Ethyl acetate : petroleum ether = 1 : 10. ^{1}H NMR (500 MHz, Chloroform-d) δ 8.09 (s, 1H), 8.04 (d, *J*=7.8, 1H), 7.81 (d, *J*=7.8, 1H), 7.71 (d, *J*=16.0, 1H), 7.61 (t, *J*=7.8, 1H), 7.49 – 7.35 (m, 4H), 6.81 (d, *J*=16.1, 1H), 3.79 (s, 3H), 2.30 (s, 3H), 1.31 (s, 9H); ^{13}C NMR (126 MHz, Chloroform-d) δ 189.7, 169.4, 169.2, 152.2, 146.5, 143.9, 138.0, 133.3, 131.9, 131.2 (q, *J* = 33.0 Hz), 130.3, 129.3 (q, *J* = 3.7 Hz), 129.2, 125.8, 125.4, 123.6 (q, *J* = 272.5 Hz), 82.1, 53.2, 34.6, 31.2, 21.0; HRMS (ESI-TOF) for $C_{25}H_{25}F_3NaO_5$ (M + Na)$^+$. Calculated: 485.1546, found: 485.1547.

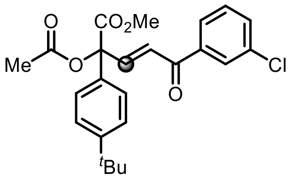

3w

Methyl ***(E)***-2-acetoxy-2-(4-(*tert*-butyl)phenyl)-5-(3-chlorophenyl)-5-oxopent-3-enoate (**3w**) (39.0 mg, 91%) as off-white solid. M. p. 112 - 113°C. Ethyl acetate : petroleum ether = 1 : 10. ^{1}H NMR (500 MHz, Chloroform-d) δ 7.83 (t, *J*=1.9, 1H), 7.77 – 7.68 (m, 2H), 7.53 (ddd, *J*=8.0, 2.2, 1.0, 1H), 7.41 (t, *J*=7.6, 5H), 6.80 (d, *J*=15.9, 1H), 3.79 (s, 3H), 2.30 (s, 3H), 1.31 (s, 9H); ^{13}C NMR (126 MHz, Chloroform-d) δ 189.5, 169.4, 169.2, 152.1, 146.1, 139.0, 134.9, 133.4, 132.9, 129.9, 128.8, 126.9, 125.8, 125.7, 125.5, 82.1, 53.2, 34.6, 31.2, 21.0; HRMS (ESI-TOF) for $C_{24}H_{25}ClNaO_5$ (M + Na)$^+$. Calculated: 451.1283, found: 451.1286.

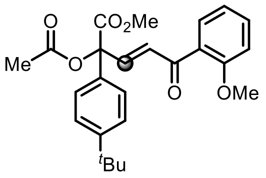

3x

Methyl ***(E)***-2-acetoxy-2-(4-(*tert*-butyl)phenyl)-5-(2-methoxyphenyl)-5-oxopent-3-enoate (**3x**) (31.8 mg, 75%) as white solid. M. p. 123 - 124°C. Ethyl acetate : petroleum ether = 1 : 10. ^{1}H NMR (500 MHz, Chloroform-d) δ 7.60 – 7.53 (m, 2H), 7.47 – 7.36 (m, 5H), 7.05 – 6.96 (m, 1H), 6.92 (d, J=8.3, 1H), 6.70 (d, J=15.9, 1H), 3.76 (s, 3H), 3.76 (s, 3H), 2.26 (s, 3H), 1.31 (s, 9H); ^{13}C NMR (126 MHz, Chloroform-d) δ 192.3, 169.5, 169.1, 158.3, 151.8, 143.0, 133.9, 133.2, 130.7, 130.5, 128.5, 125.7, 125.6, 120.8, 111.6, 82.3, 55.4, 53.0, 34.6, 31.2, 21.0; HRMS (ESI-TOF) for $C_{25}H_{28}NaO_6$ (M + Na)$^+$. Calculated: 447.1778, found: 447.1779.

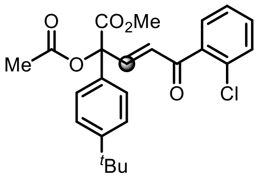

3y

Methyl ***(E)***2-acetoxy-2-(4-(*tert*-butyl)phenyl)-5-(2-chlorophenyl)-5-oxopent-3-enoate (**3y**) (37.7 mg, 88%) as colorless oily liquid. Ethyl acetate : petroleum ether = 1 : 10. ^{1}H NMR (500 MHz, Chloroform-d) δ7.55 (d, *J*=16.1, 1H), 7.44 – 7.40 (m, 3H), 7.39 (s, 4H), 7.33 (ddd, *J*=7.3, 5.8, 2.8, 1H), 6.52 (d, *J* =16.1, 1H), 3.75 (s, 3H), 2.26 (s, 3H), 1.31 (s, 9H); ^{13}C NMR (126 MHz, Chloroform-d) δ 193.2, 169.3,

152.0, 146.7, 138.1, 133.2, 131.8, 131.5, 130.3, 129.8, 129.6, 129.5, 126.8, 125.7, 125.5, 81.8, 53.2, 34.6, 31.2, 20.9; HRMS (ESI-TOF) for $C_{24}H_{25}ClNaO_5$ (M + Na)$^+$. Calculated: 451.1283, found: 451.1285.

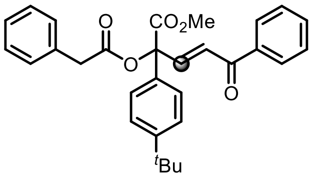

3z

Methyl ***(E)***-2-(4-(*tert*-butyl)phenyl)-5-oxo-5-phenyl-2-(2-phenylacetoxy)pent-3-enoate (**3z**) (39.0 mg, 62%) as colorless oily liquid. Ethyl acetate : petroleum ether = 1 : 10. ^{1}H NMR (500 MHz, Chloroform-d) δ 7.74 (d, J=15.9, 1H), 7.64 (d, J=7.7, 2H), 7.55 (d, J=7.1, 1H), 7.45 – 7.37 (m, 11H), 6.61 (d, J=15.9, 1H), 3.93 – 3.82 (m, 2H), 3.77 (s, 3H), 1.32 (s, 9H); ^{13}C NMR (126 MHz, Chloroform-d) δ 190.1, 169.6, 169.2, 152.0, 144.6, 137.2, 133.6, 133.2, 132.9, 130.0, 129.5, 129.4, 128.9, 128.7, 128.6, 128.5, 127.5, 125.8, 125.8, 125.5, 125.4, 124.9, 82.4, 53.2, 41.6, 34.6, 31.2; HRMS (ESI-TOF) for $C_{30}H_{30}NaO_5$ (M + Na)$^+$. Calculated: 493.1985, found: 493.1986.

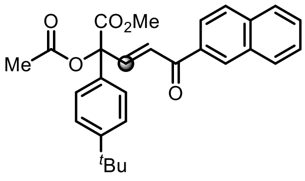

3aa

Methyl ***(E)***-2-acetoxy-2-(4-(*tert*-butyl)phenyl)-5-(naphthalen-2-yl)-5-oxopent-3-enoate (**3aa**) (42.9 mg, 97%) as White solid. M. p. 129°C. Ethyl acetate : petroleum ether = 1 : 10. ^{1}H NMR (500 MHz, Chloroform-d) δ 8.36 (d, *J*=1.6, 1H), 7.99 – 7.84 (m, 4H), 7.74 (d, *J*=15.9, 1H), 7.57 (d, *J*=25.5, 2H), 7.47 (d, *J*=8.6, 2H), 7.41 (d, *J*=8.6, 2H), 7.00 (d, *J*=15.9, 1H), 3.80 (s, 3H), 2.31 (s, 3H), 1.31 (s, 9H); ^{13}C NMR (126 MHz, Chloroform-d) δ 190.7, 169.4, 169.4, 152.0, 145.1, 135.5, 134.8, 133.7, 132.4, 130.6, 129.6, 128.5, 128.5, 127.8, 126.8, 126.4, 125.8, 125.6, 124.5, 82.3, 53.2, 34.6, 31.2, 21.0; HRMS (ESI-TOF) for $C_{28}H_{28}NaO_5$ (M + Na)$^+$. Calculated: 467.1829, found: 467.1826.

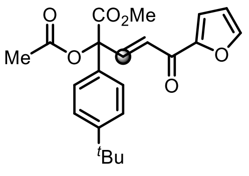

3ab

Methyl (*E*)-2-acetoxy-2-(4-(*tert*-butyl)phenyl)-5-(furan-2-yl)-5-oxopent-3-enoate (**3ab**) (26.8 mg, 70%) as white solid. M. p. 114-115°C. Ethyl acetate : petroleum ether = 1 : 10. ^{1}H NMR (500 MHz, Chloroform-d) δ 7.83 (d, J=15.9, 1H), 7.61 (d, J=1.7, 1H), 7.47 – 7.37 (m, 4H), 7.28 – 7.21 (m, 1H), 6.82 (d,

J=15.9, 1H), 6.55 (dd, J=3.6, 1.7, 1H), 3.79 (s, 3H), 2.29 (s, 3H), 1.30 (s, 9H); ^{13}C NMR (126 MHz, Chloroform-d) δ 179.3, 169.5, 169.2, 151.8, 141.7, 134.0, 131.9, 131.4, 127.4, 125.7, 125.6, 120.0, 108.3, 82.3, 53.1, 34.6, 31.2, 21.0; HRMS (ESI-TOF) for $C_{22}H_{24}NaO_6$ $(M + Na)^+$. Calculated: 407.1465, found: 407.1464.

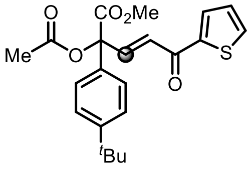

3ac

Methyl (*E*)-2-acetoxy-2-(4-(*tert*-butyl)phenyl)-5-oxo-5-(thiophen-2-yl)pent-3-enoate (**3ac**) (33.2 mg, 83%) as yellow solid. M. p. 154-155°C. Ethyl acetate : petroleum ether = 1 : 10. ^{1}H NMR (500 MHz, Chloroform-d) δ 7.81 – 7.62 (m, 3H), 7.48 – 7.36 (m, 4H), 7.13 (dd, *J*=5.0, 3.8, 1H), 6.79 (d, *J*=15.7, 1H), 3.78 (s, 3H), 2.28 (s, 3H), 1.31 (s, 9H); ^{13}C NMR (126 MHz, Chloroform-d) δ 182.1, 169.3, 169.3, 152.0, 144.6, 144.3, 134.3, 133.6, 132.6, 128.2, 126.0, 125.7, 125.6, 82.1, 53.1, 34.6, 31.2, 21.0; HRMS (ESI-TOF) for $C_{22}H_{24}NaO_5S$ $(M + Na)^+$. Calculated: 423.1237, found: 423.1239.

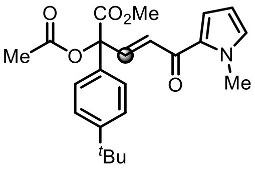

3ad

Methyl (*E*)-2-acetoxy-2-(4-(*tert*-butyl)phenyl)-5-(1-methyl-1H-pyrrol-2-yl)-5-oxopent-3-enoate (**3ad**) (32.2 mg, 81%) as white solid. M. p. 111-112°C. Ethyl acetate : petroleum ether = 1 : 10. ^{1}H NMR (500 MHz, Chloroform-d) δ 7.66 (d, *J*=15.7, 1H), 7.47 – 7.42 (m, 2H), 7.41 – 7.35 (m, 2H), 6.89 (dd, *J*=4.1, 1.7, 1H), 6.84 (t, *J*=2.0, 1H), 6.76 (d, *J*=15.7, 1H), 6.13 (dd, *J*=4.2, 2.5, 1H), 3.96 (s, 3H), 3.77 (s, 3H), 2.26 (s, 3H), 1.30 (s, 9H); ^{13}C NMR (126 MHz, Chloroform-d) δ 179.3, 169.5, 169.2, 151.8, 141.7, 134.0, 131.9, 131.4, 127.4, 125.7, 125.6, 120.0, 108.3, 82.3, 53.1, 37.7, 34.6, 31.2, 21.0; HRMS (ESI-TOF) for $C_{23}H_{27}NNaO_5$ $(M + Na)^+$. Calculated: 420.1781, found: 420.1784.

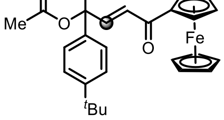

3ae

Methyl (*E*)-2-acetoxy-2-(4-(*tert*-butyl)phenyl)-5-oxo-5-(Ferrocenyl)pent-3-enoate (**3ae**) (47.7 mg, 95%) as red solid. M. p. 175 - 176°C. Ethyl acetate : petroleum ether = 1 : 10. ^{1}H NMR (500 MHz, Chlo

roform-d) δ 7.75 (d, J=15.8, 1H), 7.52–7.35 (m, 4H), 6.51 (d, J=15.8, 1H), 4.83–4.68 (m, 2H), 4.60–4.49 (m, 2H), 4.14 (s, 5H), 3.79 (s, 3H), 2.30 (s, 3H), 1.31 (s, 9H); ^{13}C NMR (126 MHz, Chloroform-d) δ 192.5, 169.5, 169.2, 151.9, 140.9, 134.0, 127.6, 125.7, 125.6, 82.2, 79.9, 72.9, 70.1, 70.0, 69.7, 53.1, 34.6, 31.2, 21.1; HRMS (ESI-TOF) for $C_{28}H_{30}FeNaO_5$ (M + Na)$^+$. Calculated: 525.1335 $^+$, found: 525.1339.

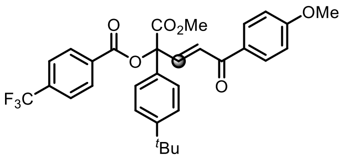

3af

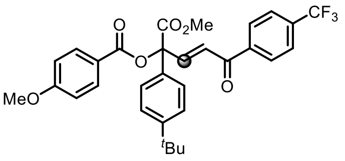

3ag

(*E*)-2-(4-(*tert*-butyl)phenyl)-1-methoxy-5-(4-methoxyphenyl)-1,5-dioxopent-3-en-2-yl 4-(trifluoromethyl)benzoate (**3af**); (*E*)-2-(4-(*tert*-butyl)phenyl)-1-methoxy-1,5-dioxo-5-(4-(trifluoromethyl)phenyl)pent-3-en-2-yl 4-methoxybenzoate (**3ag**) (**3af**：**3ag** = 1：0.9, 43.2 mg, 78%) as white solid. ^{1}H NMR (500 MHz, Chloroform-*d*) δ 8.31 (d, *J* = 8.0 Hz, 2H), 8.20 – 8.13 (m, 2H), 7.91 (d, *J* = 8.1 Hz, 2H), 7.88 – 7.74 (m, 6H), 7.69 (d, *J* = 8.0 Hz, 2H), 7.55 (dd, *J* = 8.6, 2.3 Hz, 4H), 7.46 (dd, *J* = 8.5, 1.8 Hz, 4H), 7.00 (d, *J* = 8.8 Hz, 2H), 6.97 – 6.81 (m, 4H), 3.90 (s, 3H), 3.85 (s, 3H), 3.80 (s, 3H), 3.79 (s, 3H), 1.33 (d, *J* = 1.9 Hz, 18H); ^{13}C NMR (126 MHz, Chloroform-*d*) δ 190.2, 188.8, 169.5, 169.1, 164.6, 164.2, 163.7, 163.6, 152.2, 152.1, 146.8, 143.4, 140.3, 135.2 (q, *J* = 32.8 Hz), 134.1 (q, *J* = 32.6 Hz), 133.9, 133.7, 132.6, 132.2, 131.1, 130.4, 130.3, 129.1, 126.5, 125.9, 125.9, 125.7, 125.7, 125.7, 125.6, 125.6, 125.5, 123.6 (q, *J* = 272.9 Hz), 123.5 (q, *J* = 272.7 Hz), 121.3, 114.0, 113.8, 83.1, 82.1, 55.5, 55.4, 53.3, 53.2, 34.7, 34.6, 31.2; HRMS (ESI-TOF) for $C_{31}H_{29}F_3NaO_6$ (M + Na)$^+$. Calculated: 577.1808, found: 577.1808.

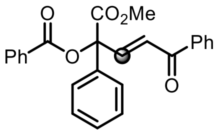

4a

(*E*)-1-methoxy-1,5-dioxo-2,5-diphenylpent-3-en-2-yl benzoate (**4a**) (36.0 mg, 90%) as white solid. M. p. 94-95°C. Ethyl acetate : petroleum ether = 1 : 10. ^{1}H NMR (500 MHz, Chloroform-d) δ 8.21 (dt, *J*=8.4, 1.5, 2H), 7.88 – 7.78 (m, 3H), 7.72 – 7.61 (m, 3H), 7.53 (td, *J*=7.6, 1.5, 3H), 7.49 – 7.36 (m, 5H), 6.91 (dd, *J*=15.9, 2.6, 1H), 3.79 (s, 3H); ^{13}C NMR (126 MHz, Chloroform-d) δ190.7, 169.3, 164.9, 144.9, 137.4, 137.2, 133.8, 132.9, 130.0, 129.2, 129.0, 128.9, 128.8, 128.7, 128.6, 126.7, 125.9, 82.5, 53.2; HRMS (ESI-TOF) for $C_{25}H_{20}NaO_5$ (M + Na)$^+$. Calculated: 423.1203, found: 423.1204.

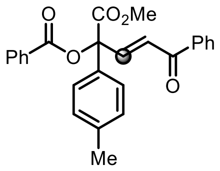

4b

(*E*)-1-methoxy-1,5-dioxo-5-phenyl-2-(p-tolyl)pent-3-en-2-yl benzoate (**4b**) (27.3 mg, 66%) as colorless oily liquid. Ethyl acetate : petroleum ether = 1 : 10. ^{1}H NMR (500 MHz, Chloroform-d) δ 8.22 – 8.17 (m, 2H), 7.86 – 7.80 (m, 3H), 7.68 – 7.63 (m, 1H), 7.56 – 7.50 (m, 5H), 7.42 (dd, J=8.3, 7.1, 2H), 7.26 (s, 2H), 6.90 (d, J=16.0, 1H), 3.78 (s, 3H), 2.38 (s, 3H); ^{13}C NMR (126 MHz, Chloroform-d) δ 190.8, 169.4, 164.9, 145.1, 139.0, 137.4, 134.2, 133.8, 132.9, 130.0, 129.6, 129.2, 128.8, 128.7, 128.5, 126.4, 125.8, 82.4, 53.2, 21.1; HRMS (ESI-TOF) for $C_{26}H_{22}NaO_5$ $(M + Na)^+$. Calculated: 437.1359, found: 437.1361.

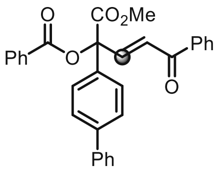

4c

(*E*)-2-([1,1'-biphenyl]-4-yl)-1-methoxy-1,5-dioxo-5-phenylpent-3-en-2-yl benzoate (**4c**) (28.5 mg, 60%) as white solid. M. p. 52 - 54°C. Ethyl acetate : petroleum ether = 1 : 10. ^{1}H NMR (500 MHz, Chloroform-d) δ 8.26 – 8.21 (m, 2H), 7.89 (d, *J*=15.9, 1H), 7.86 – 7.82 (m, 2H), 7.76 – 7.71 (m, 3H), 7.68 – 7.64 (m, 2H), 7.62 – 7.58 (m, 2H), 7.58 – 7.52 (m, 2H), 7.44 (dt, *J*=16.1, 7.7, 4H), 7.38 (d, *J*=7.3, 1H), 6.97 (d, *J*=16.0, 1H), 3.81 (s, 3H); ^{13}C NMR (126 MHz, Chloroform-d) δ 190.7, 169.3, 164.8, 144.9, 141.9, 140.1, 137.3, 136.0, 133.9, 133.0, 130.0, 129.1, 128.9, 128.8, 128.7, 128.6, 127.7, 127.6, 127.1, 126.6, 126.3, 82.4, 53.3; HRMS (ESI-TOF) for $C_{31}H_{24}NaO_5$ $(M + Na)^+$ .Calculated: 499.1516, found: 499.1513.

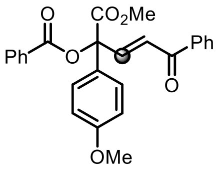

4d

(*E*)-1-methoxy-2-(4-methoxyphenyl)-1,5-dioxo-5-phenylpent-3-en-2-yl benzoate (**4d**) (32.9 mg, 77%) as colorless oily liquid. Ethyl acetate : petroleum ether = 1 : 10. ^{1}H NMR (500 MHz, Chloroform-d) δ 8.19 (dt, J=8.2, 1.1, 2H), 7.89 – 7.79 (m, 3H), 7.68 – 7.62 (m, 1H), 7.59 – 7.55 (m, 2 H), 7.53 (td, J=7.5, 4.3, 3 H), 7.42 (t, J=7.7, 2H), 6.99 – 6.90 (m, 3H), 3.83 (s, 3H), 3.78 (s, 3 H); ^{13}C NMR (126 MHz,

Chloroform-d) δ 190.7, 169.4, 164.9, 160.0, 145.1, 137.4, 133.7, 132.9, 130.0, 129.3, 129.1, 128.7, 128.6, 128.5, 127.5, 126.5, 114.2, 82.3, 55.3, 53.2; HRMS (ESI-TOF) for $C_{26}H_{22}NaO_6$ (M + Na)$^+$. Calculated: 453.1309, found: 453.1311.

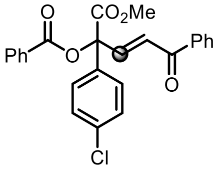

4e

(*E*)-2-(4-chlorophenyl)-1-methoxy-1,5-dioxo-5-phenylpent-3-en-2-yl benzoate (**4e**) (21.7 mg, 50%) as colorless oily liquid. Ethyl acetate : petroleum ether = 1 : 10. ^{1}H NMR (500 MHz, Chloroform-d) δ 8.20 – 8.15 (m, 2H), 7.85 – 7.77 (m, 3H), 7.67 (s, 1H), 7.63 – 7.58 (m, 2H), 7.57 – 7.51 (m, 3H), 7.46 – 7.40 (m, 4H), 6.94 (d, *J*=15.9, 1H), 3.79 (s, 3H); ^{13}C NMR (126 MHz, Chloroform-d) δ 190.4, 169.0, 164.7, 144.4, 137.2, 135.7, 135.1, 134.0, 133.1, 130.0, 129.1, 128.9, 128.7, 128.7, 128.6, 127.4, 126.9, 81.9, 53.4; HRMS (ESI-TOF) for $C_{25}H_{19}ClNaO_5$ (M + Na)$^+$. Calculated : 457.0813, found: 457.0814.

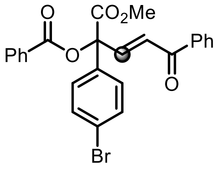

4f

(*E*)-2-(4-bromophenyl)-1-methoxy-1,5-dioxo-5-phenylpent-3-en-2-yl benzoate (**4f**) (20.8 mg, 42%) as colorless oily liquid. Ethyl acetate : petroleum ether = 1 : 10. ^{1}H NMR (500 MHz, Chloroform-d) δ 8.20 – 8.15 (m, 2H), 7.85 – 7.81 (m, 2H), 7.79 (d, *J*=15.9, 1H), 7.66 (d, *J*=7.5, 1H), 7.59 – 7.51 (m, 7H), 7.43 (t, *J*=7.7, 2H), 6.93 (d, *J*=15.9, 1H), 3.79 (s, 3H); ^{13}C NMR (126 MHz, Chloroform-d) δ 190.4, 169.0, 164.7, 144.3, 137.2, 136.2, 134.0, 133.1, 132.1, 130.0, 128.9, 128.8, 128.7, 128.6, 127.7, 126.9, 123.4, 81.9, 53.4; HRMS (ESI-TOF) for $C_{25}H_{19}BrNaO_5$ (M + Na)$^+$. Calculated: 501.0308, found: 501.0311.

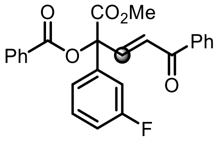

4g

(*E*)-2-(3-fluorophenyl)-1-methoxy-1,5-dioxo-5-phenylpent-3-en-2-yl benzoate (**4g**) (26.8 mg, 64%) as colorless oily liquid. Ethyl acetate : petroleum ether = 1 : 10. ^{1}H NMR (500 MHz, Chloroform-d) δ 8.25 – 8.13 (m, 2H), 7.86 – 7.77 (m, 3H), 7.72 – 7.63 (m, 1H), 7.54 (td, *J*=7.7, 2.1, 3H), 7.47 – 7.39 (m, 5H), 7.10 (dt, *J*=5.8, 2.9, 1H), 6.93 (d, *J*=15.9, 1H), 3.80 (s, 3H); ^{13}C NMR (126 MHz, Chloroform-d) δ1

90.5, 168.9, 164.7, 162.8 (d, *J* = 247.5 Hz), 144.2, 139.5 (d, *J* = 7.4 Hz), 137.2, 134.0, 133.1, 130.5 (d, *J* = 8.2 Hz), 130.0, 128.9, 128.8, 128.6, 126.9, 121.6 (d, *J* = 3.0 Hz), 116.0 (d, *J* = 21.0 Hz), 113.3 (d, *J* = 24.0 Hz), 81.8, 53.4; HRMS (ESI-TOF) for $C_{25}H_{19}FNaO_5(M + K)^+$. Calculated: 457.0848, found: 457.0 841.

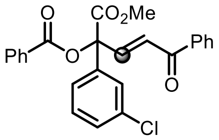

4h

(*E*)-2-(3-chlorophenyl)-1-methoxy-1,5-dioxo-5-phenylpent-3-en-2-yl benzoate (**4h**) (21.2 mg, 49%) as colorless oily liquid. Ethyl acetate : petroleum ether = 1 : 10. ^{1}H NMR (500 MHz, Chloroform-d) δ 8. 21 – 8.15 (m, 2H), 7.88 – 7.80 (m, 4H), 7.63 (d, *J*=7.5, 1H), 7.55 – 7.48 (m, 3H), 7.46 – 7.38 (m, 4H), 7. 37 – 7.33 (m, 1H), 6.90 (d, *J*=15.9, 1H), 3.86 (s, 3H); ^{13}C NMR (126 MHz, Chloroform-d) δ 190.6, 168. 2, 164.4, 143.0, 137.4, 135.2, 133.7, 133.0, 132.6, 131.7, 130.3, 130.1, 129.3, 129.3, 128.8, 128.6, 128.6, 127.6, 126.9, 83.1, 53.4; HRMS (ESI-TOF) for $C_{25}H_{19}ClNaO_5(M + Na)^+$. Calculated: 457.0813, found: 457.0810.

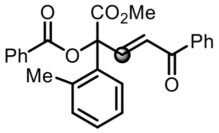

4i

(*E*)-1-methoxy-1,5-dioxo-5-phenyl-2-(o-tolyl)pent-3-en-2-yl benzoate (**4i**) (29.7 mg, 72%) as color less oily liquid. Ethyl acetate : petroleum ether = 1 : 10. ^{1}H NMR (500 MHz, Chloroform-d) δ 8.20 – 8. 14 (m, 2H), 7.88 (d, *J*=15.9, 1H), 7.85 – 7.80 (m, 2H), 7.69 – 7.61 (m, 2H), 7.55 – 7.49 (m, 3H), 7.42 (t, *J*=7.8, 2H), 7.34 – 7.29 (m, 2H), 7.24 (s, 1H), 6.85 (d, *J*=15.9, 1H), 3.82 (s, 3H), 2.47 (s, 3H); ^{13}C NM R (126 MHz, Chloroform-d) δ 190.5, 169.1, 164.9, 144.6, 137.3, 136.6, 135.4, 133.7, 133.0, 132.8, 130. 0, 129.4, 129.0, 128.7, 128.7, 128.6, 127.6, 127.2, 126.1, 84.0, 53.2, 21.8; HRMS (ESI-TOF) for $C_{26}H_{22}NaO_5$ $(M + Na)^+$. Calculated: 437.1359, found: 437.1357.

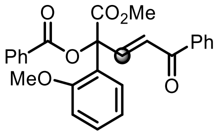

4j

(*E*)-1-methoxy-2-(2-methoxyphenyl)-1,5-dioxo-5-phenylpent-3-en-2-yl benzoate (**4j**) (23.7 mg, 55%) as colorless oily liquid. Ethyl acetate : petroleum ether = 1 : 10. ^{1}H NMR (500 MHz, Chloroform-d) δ 8.20 (d, *J*=7.7, 2H), 7.90 (dd, *J*=24.6, 11.9, 2H), 7.80 (d, *J*=7.7, 2H), 7.62 (t, *J*=7.5, 1H), 7.50 (q, *J*=6.9, 6.3, 3H), 7.44 – 7.31 (m, 3H), 7.10 (t, *J*=7.6, 1H), 6.93 (d, *J*=8.2, 1H), 6.81 (d, *J*=15.9, 1H), 3.82 (s, 3H), 3.80 (s, 3H); ^{13}C NMR (126 MHz, Chloroform-d) δ 191.0, 169.1, 164.4, 155.8, 144.8, 137.4, 133.6, 132.7, 130.2, 129.8, 129.3, 128.6, 128.5, 128.4, 127.1, 126.4, 124.8, 121.0, 111.9, 81.7, 55.7, 52.8; HRMS (ESI-TOF) for $C_{26}H_{22}NaO_6$ (M + Na)$^+$. Calculated: 453.1309, found: 453.1310.

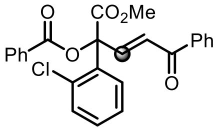

4k

(*E*)-2-(2-chlorophenyl)-1-methoxy-1,5-dioxo-5-phenylpent-3-en-2-yl benzoate (**4k**) (20.3 mg, 47%) as colorless oily liquid. Ethyl acetate : petroleum ether = 1 : 10. ^{1}H NMR (500 MHz, Chloroform-d) δ 8.21 – 8.14 (m, 2H), 7.85 – 7.75 (m, 3H), 7.67 (t, *J*=7.5, 1H), 7.58 (d, *J*=8.7, 2H), 7.56 – 7.51 (m, 5H), 7.43 (t, *J*=7.6, 2H), 6.93 (d, *J*=15.9, 1H), 3.79 (s, 3H); ^{13}C NMR (126 MHz, Chloroform-d) δ 190.4, 168.9, 164.7, 144.3, 137.2, 136.2, 134.0, 133.1, 132.1, 130.0, 129.0, 128.8, 128.7, 128.6, 127.7, 126.9, 123.4, 82.0, 53.4; HRMS (ESI-TOF) for $C_{25}H_{19}ClNaO_5$ (M + Na)$^+$. Calculated: 457.0813, found: 457.0815.

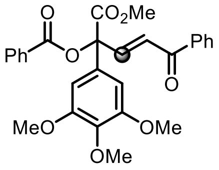

4l

(*E*)-1-methoxy-1,5-dioxo-5-phenyl-2-(3,4,5-trimethoxyphenyl)pent-3-en-2-yl benzoate (**4l**) (31.9 mg, 65%) as white solid. M. p.74 - 75°C. Ethyl acetate : petroleum ether = 1 : 10. ^{1}H NMR (500 MHz, Chloroform-d) δ 8.22 – 8.15 (m, 2H), 7.83 (s, 2H), 7.81 (s, 1 H), 7.66 (d, J=7.5, 1H), 7.57 – 7.51 (m, 3H), 7.43 (t, J=7.8, 2H), 6.94 (d, J=15.9, 1H), 6.88 (s, 2H), 3.91 (s, 6H), 3.88 (s, 3H), 3.80 (s, 3H); ^{13}C NMR (126 MHz, Chloroform-d) δ 190.6, 169.3, 164.7, 153.4, 144.5, 138.7, 137.4, 133.9, 133.0, 132.4, 129.9, 129.2, 129.0, 128.8, 128.6, 126.6, 103.5, 82.2, 60.8, 56.3, 53.3; HRMS (ESI-TOF) for $C_{28}H_{26}NaO_8$ (M + Na)$^+$. Calculated: 513.1520, found: 513.1521.

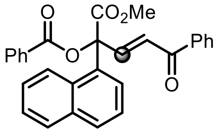

4m

(*E*)-1-methoxy-2-(naphthalen-1-yl)-1,5-dioxo-5-phenylpent-3-en-2-yl benzoate (**4m**) (30.0 mg, 67%) as colorless oily liquid. Ethyl acetate : petroleum ether = 1 : 10. ^{1}H NMR (500 MHz, Chloroform-d) δ 8.40 – 8.34 (m, 1H), 8.20 – 8.13 (m, 2H), 8.09 (d, *J*=16.0, 1H), 7.98 – 7.88 (m, 3H), 7.78 – 7.72 (m, 2H), 7.65 – 7.60 (m, 1H), 7.56 (s, 1H), 7.50 (dq, *J*=7.4, 4.2, 3.2, 5H), 7.38 (t, *J*=7.8, 2H), 6.85 (d, *J*=15.9, 1H), 3.79 (s, 3H); ^{13}C NMR (126 MHz, Chloroform-d) δ 190.6, 169.3, 164.7, 144.7, 137.3, 134.7, 133.7, 133.0, 132.9, 130.6, 130.1, 130.0, 129.4, 129.2, 128.8, 128.7, 128.7, 128.5, 126.5, 126.1, 125.9, 125.8, 124.9, 84.2, 53.3; HRMS (ESI-TOF) for $C_{29}H_{22}NaO_5$ $(M + Na)^+$. calculated: 473.1359, found: 473.1362.

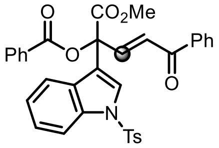

4n

(*E*)-1-methoxy-1,5-dioxo-5-phenyl-2-(1-tosyl-1H-indol-3-yl)pent-3-en-2-yl benzoate (**4n**) (52.8 mg, 89%) as colorless oily liquid. Ethyl acetate : petroleum ether = 1 : 10. ^{1}H NMR (500 MHz, Chloroform-d) δ 8.16 – 8.10 (m, 2H), 7.98 (d, *J*=8.4, 1H), 7.92 (s, 1H), 7.85 – 7.76 (m, 5H), 7.66 – 7.61 (m, 2H), 7.52 (dt, *J*=15.4, 7.6, 3H), 7.42 (t, *J*=7.8, 2H), 7.35 – 7.32 (m, 1H), 7.29 – 7.18 (m, 3H), 7.00 (d, *J*=15.9, 1H), 3.79 (s, 3H), 2.33 (s, 3H); ^{13}C NMR (126 MHz, Chloroform-d) δ 190.2, 168.4, 164.6, 145.3, 142.9, 137.2, 134.9, 133.1, 130.0, 130.0, 129.0, 128.8, 128.7, 128.6, 128.0, 127.5, 127.0, 125.6, 125.1, 123.7, 121.7, 118.4, 113.7, 80.1, 53.4, 21.6; HRMS (ESI-TOF) for $C_{34}H_{27}NNaO_7S$ $(M + Na)^+$. Calculated: 616.1400, found: 616.1403.

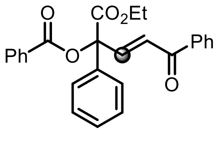

4o

(*E*)-1-ethoxy-1,5-dioxo-2,5-diphenylpent-3-en-2-yl benzoate (**4o**) (35.9 mg, 87%) as colorless oily liquid. Ethyl acetate : petroleum ether = 1 : 10. ^{1}H NMR (500 MHz, Chloroform-d) δ 8.25 – 8.17 (m, 2H), 7.88 – 7.80 (m, 3H), 7.71 – 7.63 (m, 3H), 7.53 (td, *J*=7.5, 7.1, 1.4, 3H), 7.48 – 7.36 (m, 5H), 6.90 (d, *J*=15.9, 1H), 4.27 (dd, *J*=20.3, 7.1, 2H), 1.21 (t, *J*=7.1, 3H); ^{13}C NMR (126 MHz, Chloroform-d) δ 190.

8, 168.7, 164.8, 145.1, 137.4, 137.3, 133.7, 132.9, 130.0, 129.3, 128.9, 128.8, 128.8, 128.7, 128.5, 126.6, 125.9, 82.6, 62.4, 13.9; HRMS (ESI-TOF) for $C_{26}H_{22}NaO_5$ $(M + Na)^+$. Calculated: 437.1359, found: 437.1361.

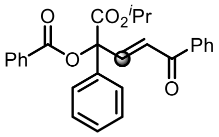

4p

(*E*)-1-isopropoxy-1,5-dioxo-2,5-diphenylpent-3-en-2-yl benzoate (**4p**) (30.8 mg, 72%) as white solid. M. p. 65 - 66°C. Ethyl acetate : petroleum ether = 1 : 10. ^{1}H NMR (500 MHz, Chloroform-d) δ 8.20 (dd, J=8.1, 1.4, 2H), 7.87 – 7.79 (m, 3H), 7.69 – 7.61 (m, 3H), 7.53 (t, J=7.7, 3H), 7.47 – 7.35 (m, 5H), 6.89 (d, J=16.0, 1H), 5.13 (p, J=6.3, 1H), 1.19 (dd, J=8.4, 6.2, 6H); ^{13}C NMR (126 MHz, Chloroform-d) δ 190.9, 168.1, 164.8, 145.3, 137.5, 137.4, 137.3, 133.7, 132.9, 129.9, 129.4, 128.8, 128.8, 128.7, 128.5, 126.5, 125.9, 82.7, 70.3, 21.4; HRMS (ESI-TOF) for $C_{27}H_{24}NaO_5$ $(M + Na)^+$. Calculated: 451.1516, found: 451.1519.

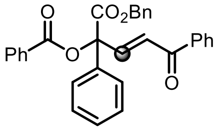

4q

(*E*)-1-(benzyloxy)-1,5-dioxo-2,5-diphenylpent-3-en-2-yl benzoate (**4q**) (37.1 mg, 78%) as colorless oily liquid. Ethyl acetate : petroleum ether = 1 : 10. ^{1}H NMR (500 MHz, Chloroform-d) δ 8.18 (d, *J*=7.6, 2H), 7.85 (d, *J*=15.9, 1H), 7.80 (d, *J*=7.7, 2H), 7.65 – 7.60 (m, 3H), 7.51 (t, *J*=7.6, 3H), 7.39 (dd, *J*=9.1, 7.0, 5H), 7.24 (d, *J*=6.9, 3H), 7.20 – 7.15 (m, 2H), 6.92 (d, *J*=15.9, 1H), 5.22 (d, *J*=2.8, 2H); ^{13}C NMR (126 MHz, Chloroform-d) δ 190.8, 168.5, 164.9, 144.9, 137.3, 136.9, 134.8, 133.8, 132.9, 129.1, 128.9, 128.8, 128.8, 128.6, 128.5, 128.4, 128.2, 128.0, 126.7, 126.5, 125.9, 82.5, 67.9; HRMS (ESI-TOF) for $C_{31}H_{24}NaO_5$ $(M + Na)^+$. Calculated: 499.1516, found: 499.1515.

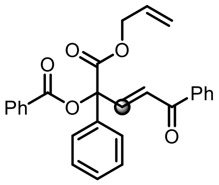

4r

(*E*)-1-(allyloxy)-1,5-dioxo-2,5-diphenylpent-3-en-2-yl benzoate (**4r**) (27.0 mg, 63%) as white solid. M. p. 72 - 73°C. Ethyl acetate : petroleum ether = 1 : 10. ^{1}H NMR (500 MHz, Chloroform-d) δ 8.27 – 8.18 (m, 2H), 7.91 – 7.82 (m, 3H), 7.70 (dd, J=7.4, 1.7, 3H), 7.56 (t, J=7.6, 3H), 7.51 – 7.42 (m, 5H), 6.95 (d, J=15.9, 1H), 5.92 – 5.78 (m, 1H), 5.27 – 5.15 (m, 2H), 4.72 (dt, J=5.7, 1.4, 2H); ^{13}C NMR (126 MHz, Chloroform-d) δ 190.8, 168.4, 164.8, 144.9, 137.4, 137.2, 133.8, 132.9, 131.1, 130.0, 129.3, 129.0, 128.9, 128.8, 128.7, 128.5, 126.7, 125.9, 118.8, 82.5, 66.7; HRMS (ESI-TOF) for $C_{27}H_{22}NaO_5$ (M + Na)$^+$. Calculated: 449.1359, found: 449.1360.

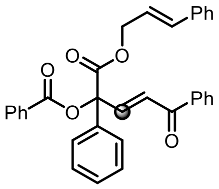

4s

(*E*)-1-(cinnamyloxy)-1,5-dioxo-2,5-diphenylpent-3-en-2-yl benzoate (**4s**) (28.6 mg, 57%) as colorless oily liquid. Ethyl acetate : petroleum ether = 1 : 10. ^{1}H NMR (500 MHz, Chloroform-d) δ 8.26 – 8.21 (m, 2H), 7.93 – 7.83 (m, 3H), 7.75 – 7.70 (m, 2H), 7.67 (d, *J*=7.4, 1H), 7.55 (td, *J*=7.6, 1.6, 3H), 7.50 – 7.41 (m, 5H), 7.33 – 7.26 (m, 5H), 6.97 (d, *J*=15.9, 1H), 6.52 (s, 1H), 6.19 (d, *J*=15.9, 1H), 4.95 – 4.83 (m, 2H); ^{13}C NMR (126 MHz, Chloroform-d) δ 190.8, 168.5, 164.9, 145.0, 137.4, 137.1, 136.0, 134.4, 133.8, 132.9, 130.0, 129.2, 129.0, 128.9, 128.8, 128.7, 128.5, 128.5, 128.0, 126.7, 126.6, 125.9, 122.1, 82.6, 66.6; HRMS (ESI-TOF) for $C_{33}H_{26}NaO_5$ (M + Na)$^+$. Calculated: 525.1672, found: 525.1675.

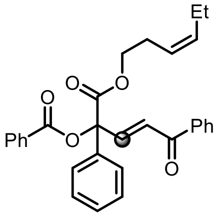

4t

(*E*)-1-(((*Z*)-hex-3-en-1-yl)oxy)-1,5-dioxo-2,5-diphenylpent-3-en-2-yl benzoate (**4t**) (31.3 mg, 67%) as white solid. M. p. 62 - 63°C. Ethyl acetate : petroleum ether = 1 : 10. ^{1}H NMR (500 MHz, Chloroform-d) δ 8.23 – 8.17 (m, 2H), 7.88 – 7.79 (m, 3H), 7.70 – 7.62 (m, 3H), 7.58 – 7.50 (m, 3H), 7.48 – 7.37 (m, 5H), 6.91 (d, *J*=16.0, 1H), 5.34 (dtt, *J*=10.5, 7.2, 1.5, 1H), 5.15 (dtt, *J*=10.7, 7.3, 1.7, 1H), 4.18 (qt, *J*=10.5, 6.9, 2H), 2.39 – 2.24 (m, 2H), 1.98 – 1.87 (m, 2H), 0.86 (t, *J*=7.6, 3H); ^{13}C NMR (126 MHz, Chloroform-d) δ 190.8, 168.8, 164.8, 145.1, 137.5, 137.3, 134.7, 133.7, 132.9, 130.0, 129.4, 128.9, 128.8, 128.8, 128.6, 128.5, 126.7, 126.0, 123.1, 82.6, 65.9, 26.4, 20.5, 14.1; HRMS (ESI-TOF) for $C_{30}H_{28}NaO_5$ (M + Na)$^+$. Calculated:491.1829, found: 491.1828.

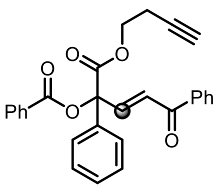

4u

(*E*)-1-(but-3-yn-1-yloxy)-1,5-dioxo-2,5-diphenylpent-3-en-2-yl benzoate (**4u**) (25.8 mg, 59%) as white solid. M. p.116-117°C. Ethyl acetate : petroleum ether = 1 : 10. ^{1}H NMR (500 MHz, Chloroform-d) δ 8.20 (dd, J=8.2, 1.4, 2H), 7.89 – 7.80 (m, 3H), 7.73 – 7.63 (m, 3H), 7.53 (td, J=7.6, 1.7, 3H), 7.49 – 7.37 (m, 5H), 6.92 (d, J=15.9, 1H), 4.35 (dt, J=10.6, 6.9, 1H), 4.24 (dt, J=10.6, 6.8, 1H), 2.48 (ddd, J=6.8, 4.1, 2.8, 2H), 1.78 (t, J=2.7, 1H); ^{13}C NMR (126 MHz, Chloroform-d) δ 190.8, 168.5, 164.9, 144.8, 137.4, 137.0, 133.8, 133.0, 130.0, 129.2, 129.0, 128.9, 128.8, 128.7, 128.6, 126.8, 126.0, 82.4, 79.3, 70.0, 63.7, 18.7; HRMS (ESI-TOF) for $C_{28}H_{22}NaO_5$ $(M + Na)^+$. Calculated: 461.1359, found: 461.1361.

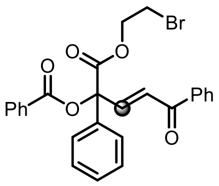

4v

(*E*)-1-(2-bromoethoxy)-1,5-dioxo-2,5-diphenylpent-3-en-2-yl benzoate (**4v**) (28.6 mg, 57%) as colorless oily liquid. Ethyl acetate : petroleum ether = 1 : 10. ^{1}H NMR (500 MHz, Chloroform-d) δ 8.21 (d, *J*=7.7, 2H), 7.87 – 7.80 (m, 3H), 7.67 (dd, *J*=11.6, 7.4, 3H), 7.54 (t, *J*=7.7, 3H), 7.49 – 7.40 (m, 5H), 6.93 (d, *J*=15.9, 1H), 4.61 – 4.50 (m, 1H), 4.49 – 4.33 (m, 1H), 3.63 (dd, *J*=15.1, 6.6, 1H), 3.52 – 3.35 (m, 1H); ^{13}C NMR (126 MHz, Chloroform-d) δ 190.7, 168.4, 165.0, 144.6, 137.3, 136.8, 133.9, 133.0, 130.0, 129.1, 128.9, 128.8, 128.7, 128.6, 126.8, 125.9, 125.8, 82.3, 65.2, 27.7.; HRMS (ESI-TOF) for $C_{26}H_{21}BrNaO_5$ $(M + Na)^+$. Calculated:515.0465, found: 515.0469.

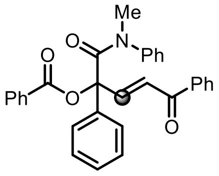

4w

(*E*)-1-(methyl(phenyl)amino)-1,5-dioxo-2,5-diphenylpent-3-en-2-yl benzoate (**4w**) (26.1 mg, 55%) as white solid. M. p. 78 - 79°C. Ethyl acetate : petroleum ether = 1 : 10. ^{1}H NMR (500 MHz, Chloroform-d) δ 8.04 (d, *J*=15.8, 1H), 7.76 (dd, *J*=17.3, 7.7, 4H), 7.61 (t, *J*=7.5, 2H), 7.46 (dq, *J*=44.3, 7.5, 13H),

6.90 (d, *J*=96.3, 3H), 6.39 (s, 1H), 6.27 (d, *J*=16.2, 1H), 3.32 (s, 3H); ^{13}C NMR (126 MHz, Chloroform-d) δ 192.7, 168.4, 167.8, 148.4, 142.2, 137.4, 133.7, 133.6, 133.6, 133.6, 132.6, 129.7, 129.0, 128.7, 128.5, 128.4, 128.3, 127.3, 125.8, 124.8, 110.0, 84.0, 29.7; HRMS (ESI-TOF) for $C_{31}H_{25}NNaO_4$ (M + Na)$^+$. Calculated: 498.1676, found: 498.1675.

Procedure for the Preparation of 5

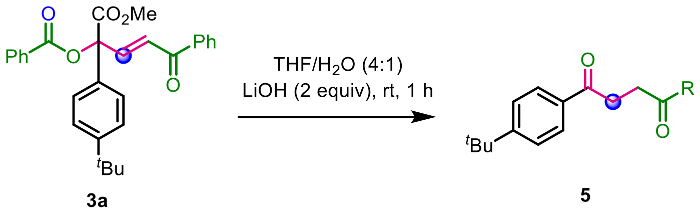

The mixture of **3a** (0.1 mmol, 45.6 mg, 1.0 equiv), LiOH (4.8 mg, 2 equiv), and THF/H_2O=4:1(1 mL) was stirred for 12 h at room temparature. Upon completion, the reaction mixture was then diluted with Et_2O (10 mL) and H_2O (10 mL). The aqueous phase was washed with Et_2O (2 × 10 mL), the combined organic phase was washed with brine, dried (Na_2SO_4) and concentrated to give the crude of product. The residue was purified by column chromatography over silica gel (ethyl acetate / petroleum ether = 1:10) to give product **5**.

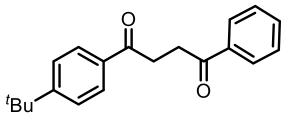

5a

1-(4-(*tert*-butyl)phenyl)-4-phenylbutane-1,4-dione (5a) (29.1 mg, 99% yield). ^{1}H NMR(500 MHz, Chloroform-d) δ 8.07 – 8.02 (m, 2H), 8.01 – 7.96 (m, 2H), 7.61 – 7.55 (m, 1H), 7.49 (ddd, *J*=10.3, 7.2, 2.5, 4H), 3.46 (d, *J*=2.3, 4H), 1.35 (s, 9H); ^{13}C NMR (126 MHz, Chloroform-d) δ 198.8, 198.4, 163.9, 156.9, 136.8, 133.1, 128.6, 128.1, 128.1, 125.6, 35.1, 32.7, 32.5, 31.1. HRMS (EI) for $C_{20}H_{22}NaO_2$ (M + Na)$^+$. calculated: 317.1512, found: 317.1514.

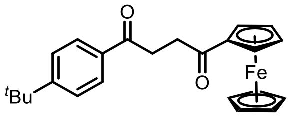

5b

1-(4-(*tert*-butyl)phenyl)-4-ferrocenyl butane-1,4-dione (5b) (39.3 mg, 98% yield). ^{1}H NMR(500 MHz, Chloroform-d) δ 8.04 – 7.96 (m, 1H), 7.52 – 7.47 (m, 1H), 4.85 (t, *J*=2.0, 1H), 4.51 (t, *J*=2.0, 1H), 4.28 (s, 2H), 3.37 (t, *J*=6.5, 1H), 3.23 (t, *J*=6.5, 1H), 1.35 (s, 4H); ^{13}C NMR (126 MHz, Chloroform-d) δ 202.8, 198.7, 156.8, 134.3, 128.1, 125.5, 78.7, 72.2, 69.9, 69.3, 35.1, 33.7, 32.1, 31.1. HRMS (EI) for $C_{24}H_{26}FeNaO_2$ (M + Na)$^+$. calculated: 425.1174, found: 425.1175.

Procedure for the Preparation of 6

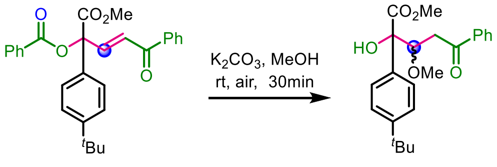

To a solution of **3a** (45.63 mg, 0.1 mmol, 1.0 equiv) in MeOH (1 mL) was added K_2CO_3 (27.6 mg, 2 equiv). The solution was stirred for 1h at room temparature. Upon completion, the reaction mixture was quenched with HOAc (0.16 mL, 2.5 equiv) and H_2O (10 mL). The mixture was extracted with dichloromethane (3 × 10 mL) and the combined organic phase was washed with brine, dried (Na_2SO_4) and concentrated to give the crude product. The residue was purified by column chromatography over silica gel (ethyl acetate / petroleum ether = 1 : 4) to give product methyl 2-(4-(*tert*-butyl)phenyl)-2-hydroxy-3-methoxy-5-oxo-5-phenylpentanoate (**6**) (34.6 mg, 90% yield, dr=5:1). ^{1}H NMR(500 MHz, Chloroform-d) δ 7.86 – 7.82 (m, 2H), 7.61 – 7.57 (m, 2H), 7.54 – 7.49 (m, 1H), 7.39 (t, *J*=7.7, 2H), 7.33 – 7.29 (m, 2H), 3.85 (s, 1H), 3.83 (s, 3H), 3.43 (s, 3H), 1.26 (s, 9H); ^{13}C NMR (126 MHz, Chloroform-d) δ 198.6, 174.3, 151.2, 136.9, 134.4, 133.1, 128.4, 128.2, 125.7, 125.4, 81.6, 81.2, 59.4, 53.1, 38.7, 34.4, 31.2. HRMS (EI) for $C_{23}H_{28}NO_5$ $(M + Na)^+$. Calculated: 407.1829, found: 407.1828.

Procedure for the Preparation of 7

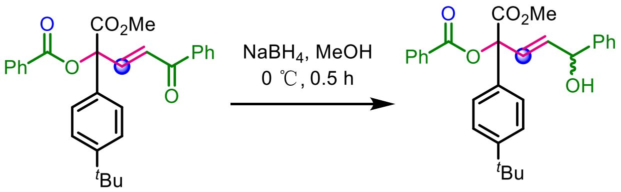

Following a modified literature procedure.[6] $NaBH_4$ (5.8 mg, 0.15 mmol, 1.5equiv) was added slowly to a stirred solution of **3a** (45.6 mg, 0.1 mmol) in MeOH (1.0 ml) at 0-5 ˚C. After stirring at the same temperature for 0.5 h, the reaction mixture was quenched with water (5.0 ml). The mixture was extracted twice with EtOAc, and the combined organic phase was washed with brine, dried (Na_2SO_4) and concentrated to give the product of methyl (*E*)-2-(4-(*tert*-butyl)phenyl)-2-hydroxy-5-oxo-5-phenylpent-3-enoate (**7**) (44.4 mg, 97%, d.r. = 1:1). ^{1}H NMR(500 MHz, Chloroform-d) δ 8.13 (td, *J*=8.1, 1.4, 2H), 7.63 – 7.57 (m, 1H), 7.56 – 7.44 (m, 4H), 7.44 – 7.37 (m, 2H), 7.32 – 7.24 (m, 5H), 6.95 (ddd, *J*=15.8, 5.8, 1.4, 1H), 5.80 (ddd, *J*=15.8, 6.0, 3.0, 1H), 5.28 (dt, *J*=6.4, 1.9, 1H), 3.74 (s, 3H), 1.32 (d, *J*=3.0, 9H); ^{13}C NMR (126 MHz, Chloroform-d) δ 170.4, 165.0, 151.4, 141.9, 135.1, 135.0, 134.8, 133.5, 130.0, 129.9, 129.9, 129.7, 128.5, 128.5, 127.8, 126.6, 126.5, 125.8, 125.8, 125.5, 125.5, 82.7, 74.1, 74.0, 53.0, 34.6, 31.2. HRMS (EI) for $C_{29}H_{30}NaO_5$ $(M + Na)^+$. Calculated: 481.1985, found: 481.1986.

Procedure for the Preparation of 8

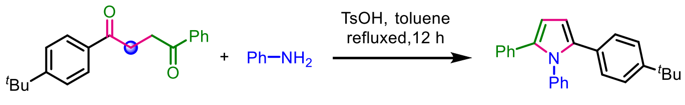

5a **8**, 85%

1-(4-(*tert*-butyl)phenyl)-4-phenylbutane-1,4-dione **(5a)** (29.4 mg, 1.0 equiv), aniline (11.2 mg, 1.2 equiv), and *p*-toluenesulfonic acid (1.7 mg, 0.1 equiv) were dissolved in toluene (1 mL). The solution was refluxed for 12 h, then cooled to room temperature and concentrated in vacuo. The residue was purified by column chromatography over silica gel (ethyl acetate / petroleum ether = 1:10) to give intermediate product 2-(4-(*tert*-butyl)phenyl)-1,5-diphenyl-1*H*-pyrrole (**8**) (29.8 mg, 85% yield).[5] ^{1}H NMR(500 MHz, Chloroform-d) δ 7.25 (d, J=6.8, 4H), 7.19 – 7.09 (m, 5H), 7.09 – 7.03 (m, 4H), 6.98 (d, J=8.3, 2H), 6.47 (q, J=3.7, 2H), 1.27 (s, 9H); ^{13}C NMR (126 MHz, Chloroform-d) δ 149.0, 139.1, 135.8, 135.5, 133.3, 130.2, 128.9, 128.7, 128.7, 128.2, 127.8, 127.2, 126.1, 124.8, 109.9, 109.6, 34.4, 31.3. HRMS (EI) for $C_{26}H_{25}NNa$ (M + Na)$^+$. calculated: 374.1879, found: 374.1881.

Deuteration Reactions

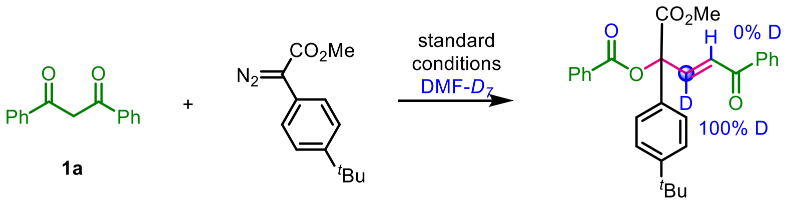

1a **2a** **3a-D-1**

Deuteration procedure for the synthesis of **3a-D-1**: A screw capped reaction vial was charged with **1a** (44.8 mg, 0.2 mmol, 2 equiv), **2a** (23.2 mg, 0.1 mmol, 1 equiv), 4Å MS (50 mg), and DMF-D_7 (0.5 mL), followed by addition of $Rh_2(OAc)_4$ (0.8 mg, 0.002 mmol, 2 mol %). The resulting mixture was stirred at room temperature for 12 h, until TLC showed complete consumption of **2a**. After the reaction was completed, the reaction mixture was evaporated under reduced pressure to leave a crude mixture, which was purified by column chromatography on silica gel (eluting with ethyl acetate/petroleum = 1:10) to afford (E)-2-(4-(*tert*-butyl)phenyl)-1-methoxy-1,5-dioxo-5-phenylpent-3-en-2-yl-3-d benzoate (**3a-D-1**), as a colorless crystal (39.3 mg, 86% yield). ^{1}H NMR(500 MHz, Chloroform-d) δ 8.26 – 8.19 (m, 2H), 7.89 – 7.83 (m, 2H), 7.68 (t, *J*=7.3, 1H), 7.61 – 7.54 (m, 5H), 7.50 – 7.43 (m, 4H), 6.92 (s, 1H), 3.82 (s, 2H), 1.36 (s, 9H); HRMS (EI) for $C_{29}H_{27}DNaO_5$ (M + Na)$^+$. Calculated: 480.1892, found: 480.1894.

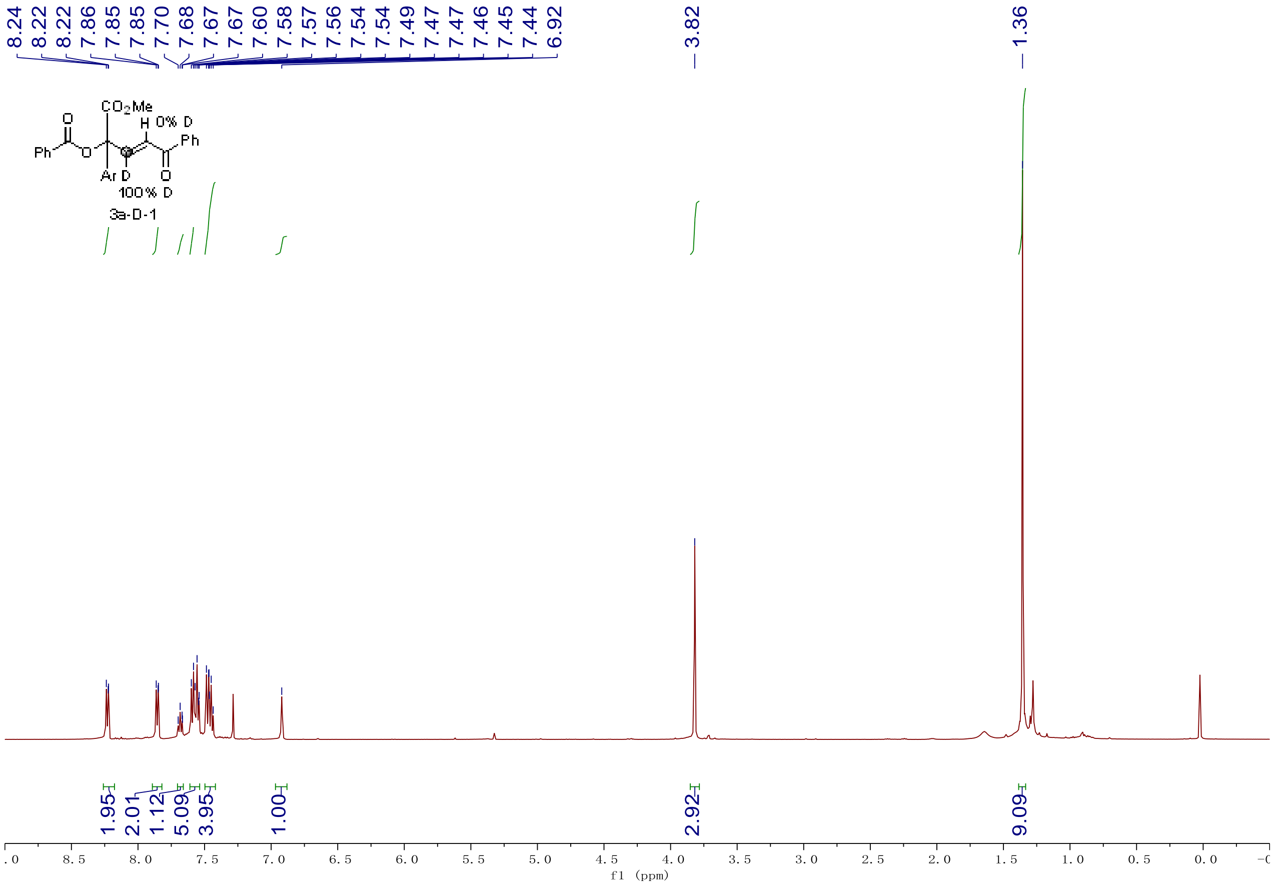

Supplementary Figure 1. ^{1}H NMR spectrum of **3a-D-1**.

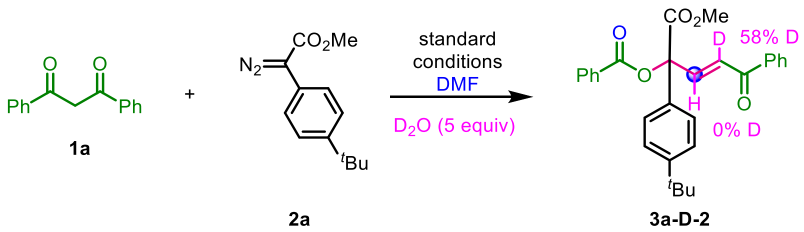

Deuteration procedure for the synthesis of **3a-D-2**: A screw capped reaction vial was charged with **1a** (44.8 mg, 0.2 mmol, 2 equiv), **2a** (23.2 mg, 0.1 mmol, 1 equiv), D_2O (9.1 mg, 5 equiv), 4Å MS (50 mg), and 0.5 mL DMF, and followed by addition of $Rh_2(OAc)_4$ (0.8 mg, 0.002 mmol, 2 mol %). The resulting mixture was stirred at room temperature for 12 h, until TLC showed complete consumption of **2a**. After the reaction was completed, the reaction mixture was evaporated under reduced pressure to leave a crude mixture, which was purified by column chromatography on silica gel (eluting with ethyl acetate/petroleum = 1:10) to afford **3a-D-2** (19.2 mg, 42% yield). ^{1}H NMR(500 MHz, Chloroform-d) δ 8.23 – 8.18 (m, 2H), 7.83 (d, *J*=7.3, 3H), 7.66 (t, *J*=7.4, 1H), 7.58 – 7.51 (m, 5H), 7.47 – 7.40 (m, 4H), 6.91 (d, *J*=15.9, 0.42 H), 3.80 (s, 3H), 1.33 (s, 9H); HRMS (EI) for $C_{29}H_{27}DNaO_5$ $(M + Na)^+$. Calculated: 480.1892, found: 480.1895.

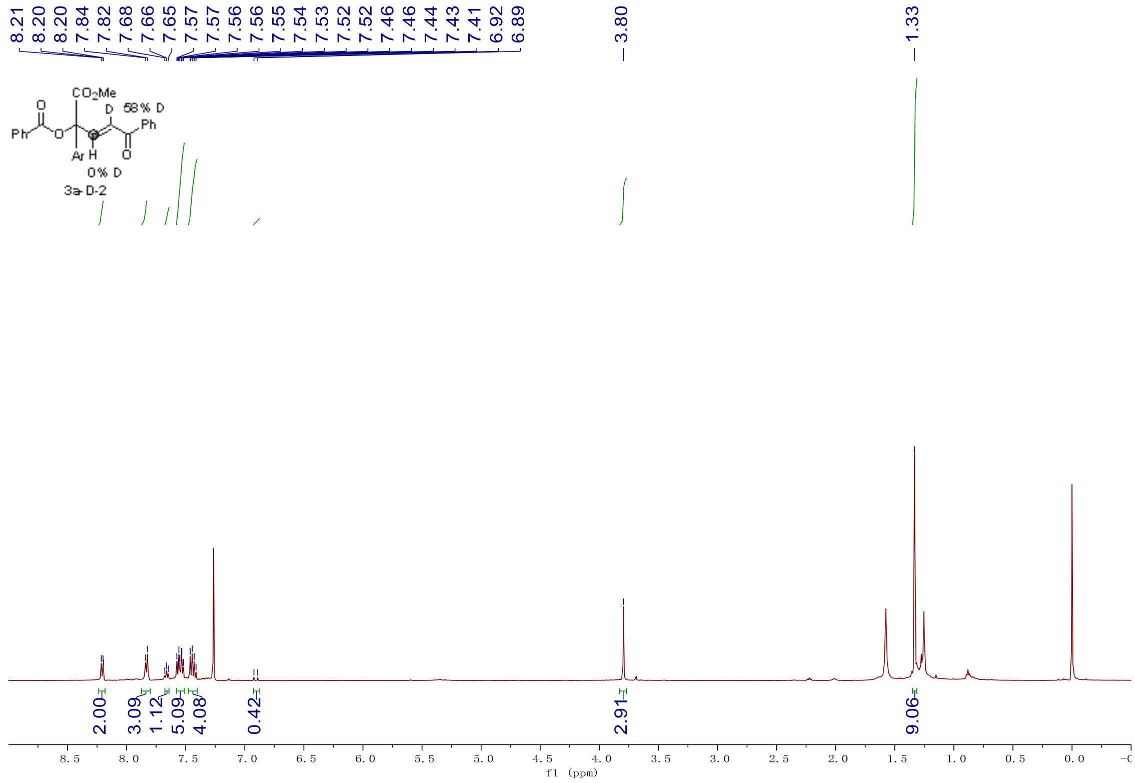

Supplementary Figure 2. ^{1}H NMR spectrum of **3a-D-2**.

Isotope-Labeling Reactions

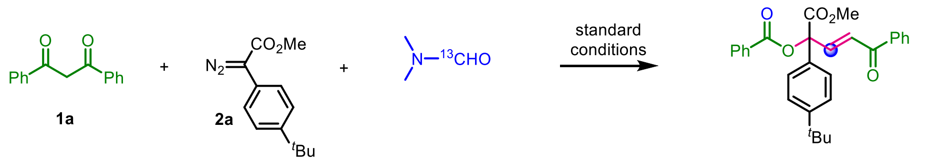

A screw capped reaction vial was charged with 1,3-diphenyl-1,3-propanedione (**1a**) (44.8 mg, 0.2 mmol, 2 equiv), methyl 2-(4-(*tert*-butyl)phenyl)-2-diazoacetate (**2a**) (23.2 mg, 0.1 mmol, 1 equiv) 4Å MS (50 mg), and DMF-formyl-^{13}C (0.5 mL), followed by the addition of $Rh_2(OAc)_4$ (0.8 mg, 0.002 mmol, 2 mol%). The resulting mixture was stirred at room temperature for 12 h, until TLC showed the complete consumption of **2a**. After the reaction was completed, the reaction mixture was evaporated under reduced pressure to leave a crude mixture, which was purified by column chromatography on silica gel (eluting with ethyl acetate/petroleum = 1:10) to afford (*E*)-2-(4-(*tert*-butyl)phenyl)-1-methoxy-1,5-dioxo-5-phenylpent-3-en-2-yl benzoate **(3a-^{13}C)**, as a colorless crystal (38.9 mg, 86% yield); M. p. 124 - 125°C. ^{1}H NMR (500 MHz, Chloroform-*d*) δ 8.24 – 8.18 (m, 2H), 7.84 (dd, *J* = 166.8 Hz, 15.9 Hz, 1H), 7.83 (dd, *J* = 8.1, 1.4 Hz, 2H), 7.69 – 7.63 (m, 1H), 7.59 – 7.51 (m, 5H), 7.48 – 7.40 (m, 4H), 6.91 (dd, *J* = 15.9, 3.3 Hz, 1H), 3.79 (s, 3H), 1.33 (s, 9H); ^{13}C NMR (126 MHz, Chloroform-*d*) δ 190.9, 169.4, 164.9, 152.1, **145.1**(**^{13}C**), 137.4, 137.1, 133.8, 132.9, 130.0, 129.3, 128.8, 128.7, 128.5, 126.6, 125.9, 125.6, 82.7, 53.2, 34.6, 31.2; HRMS (EI) for $C_{28}{}^{13}CH_{28}NaO_5$ $(M + Na)^+$. Calculated: 480.1862, found: 480.1863.

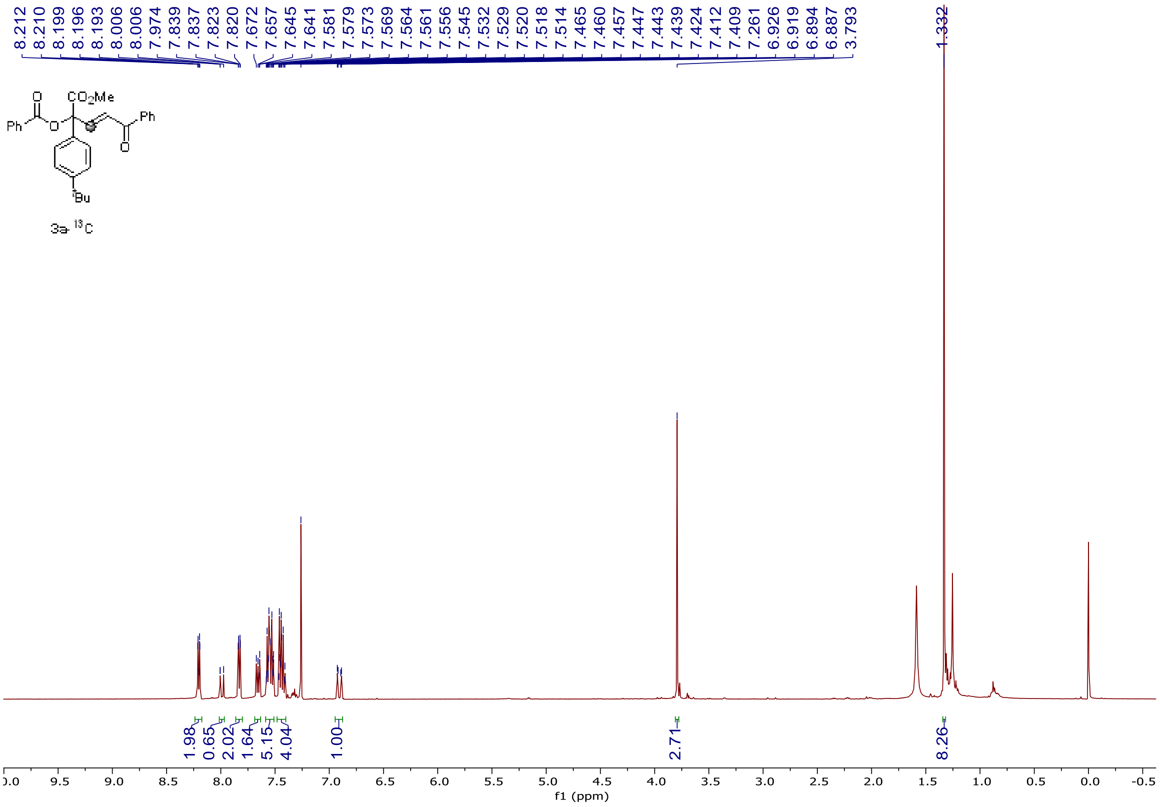

Supplementary Figure 3. ^{1}H NMR spectrum of **3a-^{13}C**.

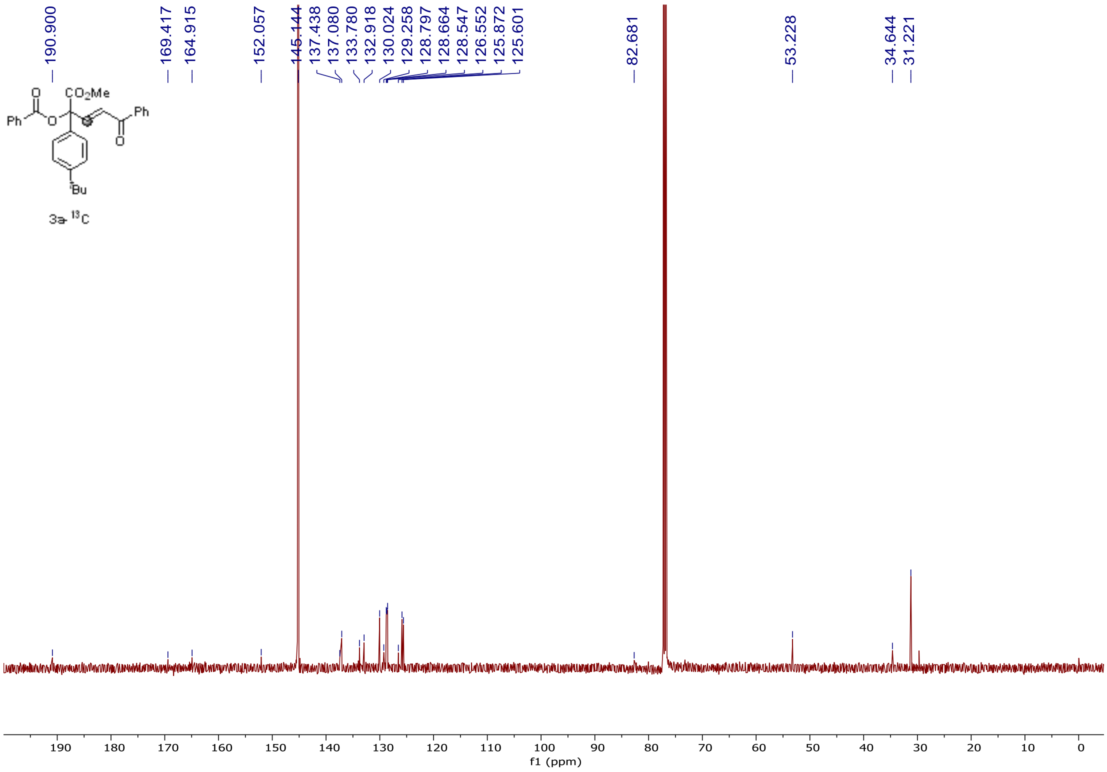

Supplementary Figure 4. ^{1}H NMR spectrum of **3a-^{13}C**.

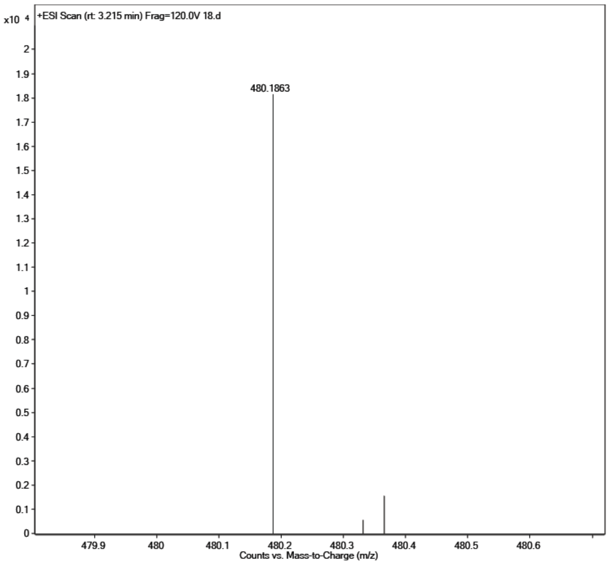

	A	B
1	m/z	Abund
2	336.1688	461060.88
3	337.1713	101191.11
4	338.1739	11921.02
5	362.3266	8717.21
6	398.1422	38019.72
7	399.1453	9076.98
8	405.262	7563.45
9	447.3474	7910.32
10	449.2876	27099.55
11	449.3592	4533.57
12	450.2908	7344.69
13	475.2312	5264.57
14	479.1826	6439.86
15	480.1875	2591648.25
16	481.1907	706224.81
17	482.1934	117243.7
18	483.1955	13810.08
19	493.3141	7083.17
20	496.162	465453.19

Supplementary Figure 5. HRMS (EI) of **3a-^{13}C**.

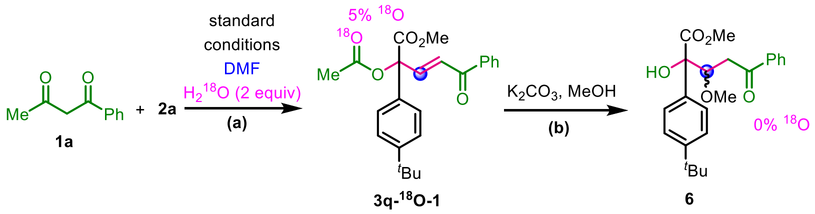

(a) Synthesis of 3q-^{18}O-1

A screw capped reaction vial was charged with **1a** (448 mg, 2 mmol, 2 equiv), **2a** (232 mg, 1 mmol, 1 equiv), 4Å MS (500 mg), $H_2{}^{18}O$ (41 mg, 2 mmol, 2 equiv), and DMF (5 mL), followed by addition of $Rh_2(OAc)_4$ (8 mg, 0.02 mmol, 2 mol %). The resulting mixture was stirred at room temperature for 12 h until TLC showed complete consumption of **2a**. After the reaction was completed, the reaction mixture was evaporated under reduced pressure to leave a crude mixture, which was purified by column chromatography on silica gel (eluting with ethyl acetate/petroleum = 1:10) to afford **3q-^{18}O-1** (231.5 mg, 55% yield). HRMS (EI) for $C_{24}H_{26}NO_4{}^{18}O$ (M + Na)$^+$. Calculated: 419.1715, found: 419.1716.

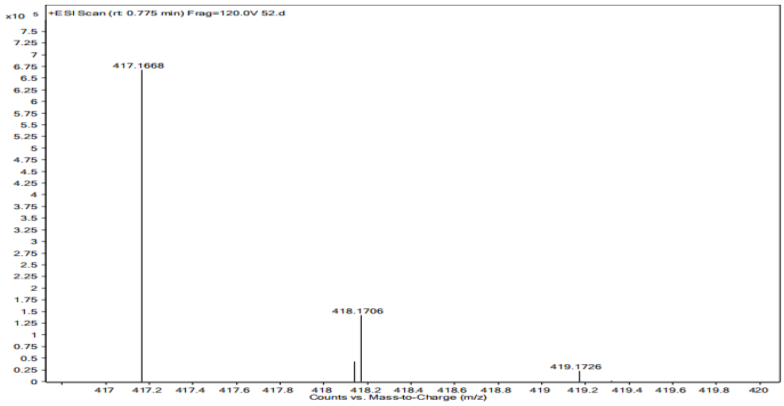

	A	B
1	m/z	Abund
2	273.1386	37804.51
3	274.1455	44019.31
4	274.2518	63474.89
5	274.274	213405.89
6	275.2769	36181.28
7	287.1539	32553
8	288.1612	39785.08
9	318.2397	22220.87
10	335.1637	31423.71
11	340.2267	22137.7
12	412.2119	58282.41
13	417.1668	667455.81
14	418.1415	42623.1
15	418.1706	141871.78
16	419.1726	32461.75
17	425.2152	2906732.25
18	426.2179	471103.22
19	427.2198	78839.48
20	433.1125	57326.11

Supplementary Figure 6. HRMS (EI) of **3q-^{18}O-1**

(b) Synthesis of 6 (from 3q-^{18}O-1)

To a solution of **3q-^{18}O-1** (45.63 mg, 0.1 mmol, 1.0 equiv) in MeOH (1 mL) was added K_2CO_3 (27.6 mg, 2 equiv). The solution was stirred for 1h at room temparature. Upon completion, the reaction mixture was quenched with HOAc (0.16 mL, 2.5 equiv) and H_2O (10 mL).. The mixture was extracted with dichloromethane (3 × 10 mL) and the combined organic phase was washed with brine, dried (Na_2SO_4) and concentrated to give the crude product. The residue was purified by column chromatography over silica gel (ethyl acetate / petroleum ether = 1:4) to give product **6** (34.1 mg, 89% yield). HRMS (EI) for $C_{23}H_{28}NO_5$ $(M + Na)^+$. Calculated: 407.1829, found: 407.1828.

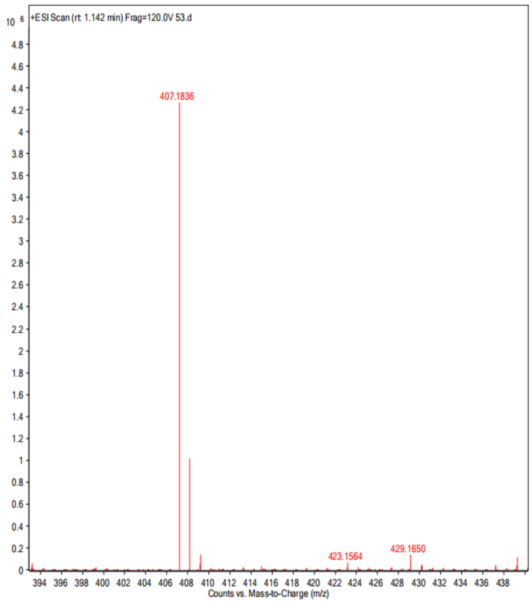

	A	B
1	m/z	Abund
2	340.2814	49064.63
3	349.1828	86054.95
4	353.2669	100612.27
5	360.3234	55572.84
6	367.1646	186375.59
7	367.19	736181.75
8	368.1931	141347.61
9	375.2509	89392.78
10	393.2092	59212.7
11	407.1836	4265222.5
12	408.1861	1009992.5
13	409.1606	46930.3
14	409.1892	133528.56
15	423.1564	67596.07
16	429.1363	42462.6
17	429.165	133758.03
18	430.168	34727.09
19	430.2594	42356.19
20	437.2355	39813.21

Supplementary Figure 7. HRMS (EI) of **6**

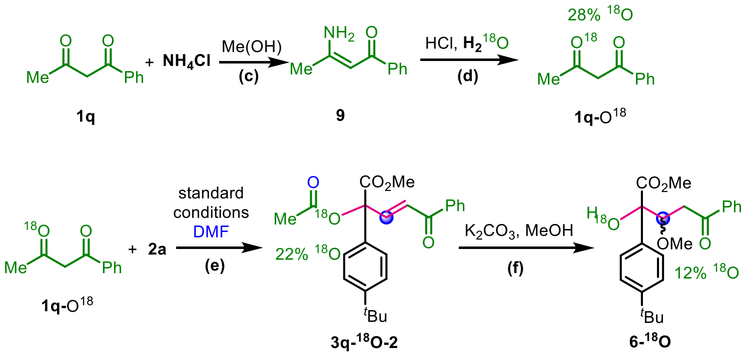

(c) Synthesis of (*Z*)-3-Amino-1-phenylbut-2-en-1-one (9)

The title compound was prepared according to the literature procedure with a slight modification.[7] A solution of 1-phenylbutane-1,3-dione (3.00 g, 18.5 mmol) and ammonium acetate (7.14 g, 92.4 mmol) in dry methanol (30 mL) was refluxed for 3 h. After cooling to room temperature, water (25 mL)

was added and the mixture was stirred well. Precipitated colorless crystals were collected by filtration and dried at 55 °C under vacuum to give **9** (2.53 g, 85%). Spectroscopic data were consistent with the literature data.[7]

(d) Synthesis of 1q-^{18}O

A screw capped reaction vial was charged with **9** (161 mg, 1mmol, 1 equiv), concentrated sulfuric acid (0.1 mL), $H_2{}^{18}O$ (30 mg, 1.5 mmol, 1.5 equiv), and MeOH (1 mL). The resulting mixture was stirred at room temperature for 1 h until TLC showed complete consumption of **9**. After the reaction was completed, the reaction mixture was evaporated under reduced pressure to leave a crude mixture, which was purified by column chromatography on silica gel (eluting with ethyl acetate/petroleum = 1:10) to afford **1q-^{18}O** (138.7 mg, 95% yield). HRMS (EI) for $C_{10}H_{10}NaO^{18}O$ $(M + Na)^+$. Calculated: 187.0615, found: 187.0619.

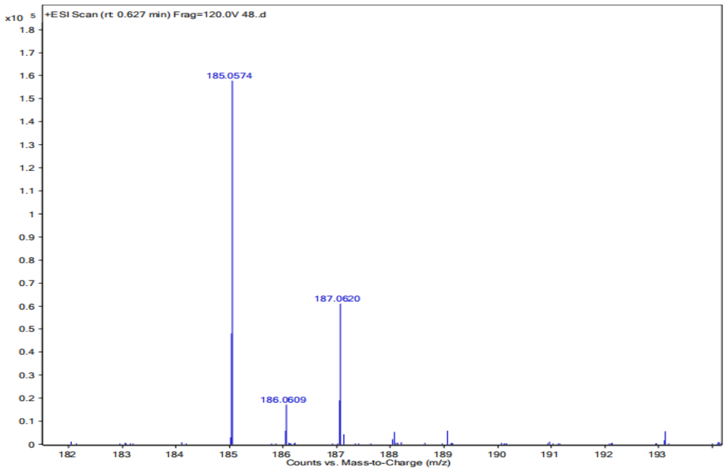

	A	B
1	m/z	Abund
2	102.1149	10861.68
3	102.1279	35880.43
4	121.0513	21861.35
5	130.1593	21013.69
6	149.0234	15539.17
7	151.1442	27563.82
8	185.0405	47938.72
9	185.0574	157771.25
10	186.0609	17226.12
11	187.0436	19051.76
12	187.062	60956.98
13	198.1278	25401.54
14	217.1053	13044.48
15	218.2111	23074.41
16	225.1962	19989.84
17	242.2842	14773.12
18	246.243	29475.26
19	247.1567	16333.83
20	247.1778	58756.61

Supplementary Figure 8. HRMS (EI) of **1q-^{18}O**

(e) Synthesis of 3q-^{18}O-2

A screw capped reaction vial was charged with **1q-^{18}O** (89.6 mg, 0.4 mmol, 2 equiv), **2a** (46.4 mg, 0.2 mmol, 1 equiv), 4Å MS (100 mg), and DMF (1.0 mL), followed by addition of $Rh_2(OAc)_4$ (1.6 mg, 0.004 mmol, 2 mol %). The resulting mixture was stirred at room temperature for 12 h until TLC showed complete consumption of **2a**. After the reaction was completed, the reaction mixture was evaporated under reduced pressure to leave a crude mixture, which was purified by column chromatography on silica gel (eluting with ethyl acetate/petroleum = 1:10) to afford **3q-^{18}O-2** (65.7 mg, 83% yield). HRMS (EI) for $C_{24}H_{26}NaO_4{}^{18}O$ $(M + Na)^+$. Calculated: 419.1715, found: 419.1716.

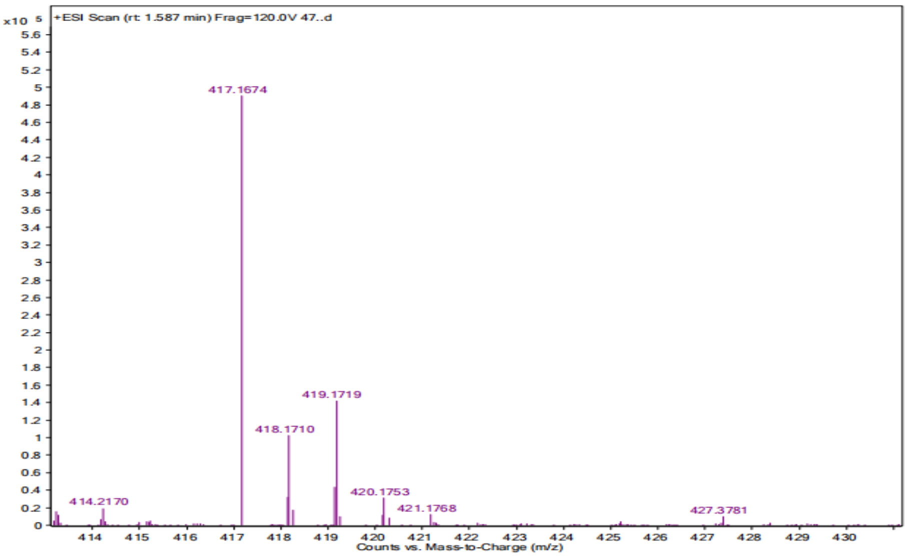

	A	B
1	m/z	Abund
2	412.2118	63820.54
3	413.2153	15485.61
4	413.2657	11606.69
5	414.217	19299.37
6	417.1674	490371.44
7	418.1425	32415.64
8	418.171	102923.44
9	418.2658	17396.51
10	419.1454	43208.89
11	419.1719	142159.25
12	419.2426	9747.37
13	420.1483	11883.85
14	420.1753	30885.06
15	421.1768	12216.04
16	427.3781	9980.02
17	433.1409	26243.03
18	437.2356	19892.26
19	440.2438	14672.31
20	447.3475	15902.53

Supplementary Figure 9. HRMS (EI) of **3q-^{18}O-2**

(f) Synthesis of 6-^{18}O (from 3q-^{18}O-2)

To a solution of **3q-^{18}O-2** (45.9 mg, 0.1 mmol, 1.0 equiv) in MeOH (1 mL) was added K_2CO_3 (27.6 mg, 2 equiv). The solution was stirred for 1h at room temparature. Upon completion, the reaction mixture was quenched with HOAc (0.16 mL, 2.5 equiv) and H_2O (10 mL).. The mixture was extracted with dichloromethane (3 × 10 mL) and the combined organic phase was washed with brine, dried (Na_2SO_4) and concentrated to give the crude product. The residue was purified by column chromatography over silica gel (ethyl acetate / petroleum ether = 1:4) to give product **6-^{18}O** (35.1 mg, 91% yield). HRMS (EI) for $C_{23}H_{28}NaO_3{}^{18}O$ (M + Na)$^+$. Calculated: 409.1871, found: 409.1872.

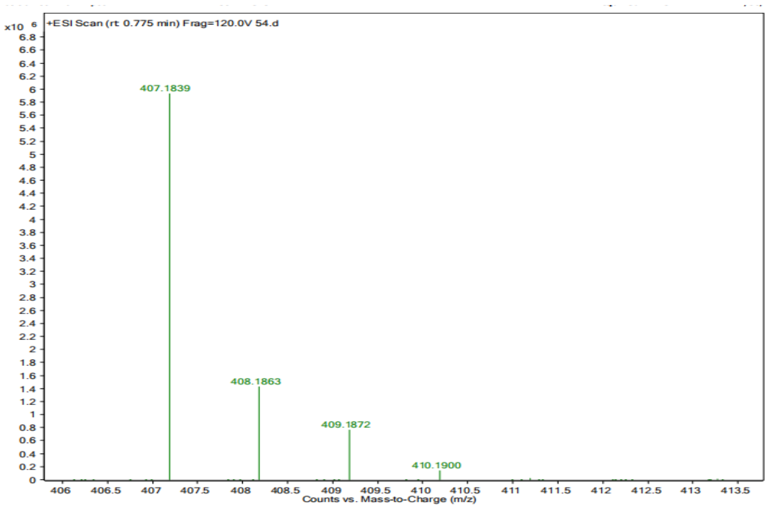

	A	B
1	m/z	Abund
2	301.141	222144.97
3	305.1569	99616.72
4	335.164	133126.28
5	345.1671	86766.78
6	349.1833	77553.52
7	367.1643	188660.5
8	367.1899	734062.81
9	368.193	143356.83
10	393.209	64820.01
11	407.1839	5930614.5
12	408.1863	1433011.12
13	409.1872	766277.69
14	410.19	139566.44
15	423.1561	103692.47
16	423.2276	69812.93
17	429.1365	79871.33
18	429.165	239918.09
19	430.1675	58098.65
20	439.2312	103939.38

Supplementary Figure 10. HRMS (EI) of **6-^{18}O**

The Gram Scale Experiment of 3a

A dried 100 mL round-bottomed flask was charged with **1a** (2.24 g, 10 mmol, 2 equiv), **2a** (1.19 g, 5 mmol, 1 equiv), 4Å MS (2.5g), and DMF (25 mL), followed by addition of $Rh_2(OAc)_4$ (40 mg, 0.002 mmol, 2 mol %). The resulting mixture was stirred at room temperature for 12 h until TLC showed complete consumption of **2a**. After the reaction was completed, the reaction mixture was evaporated under reduced pressure to leave a crude mixture, which was purified by column chromatography on silica gel (eluting with ethyl acetate/petroleum = 1:10) to afford **3a**. This was further purified by recrystallization from EtOH to give the clean product of colorless crystal (1.7325 g, 76% yield).

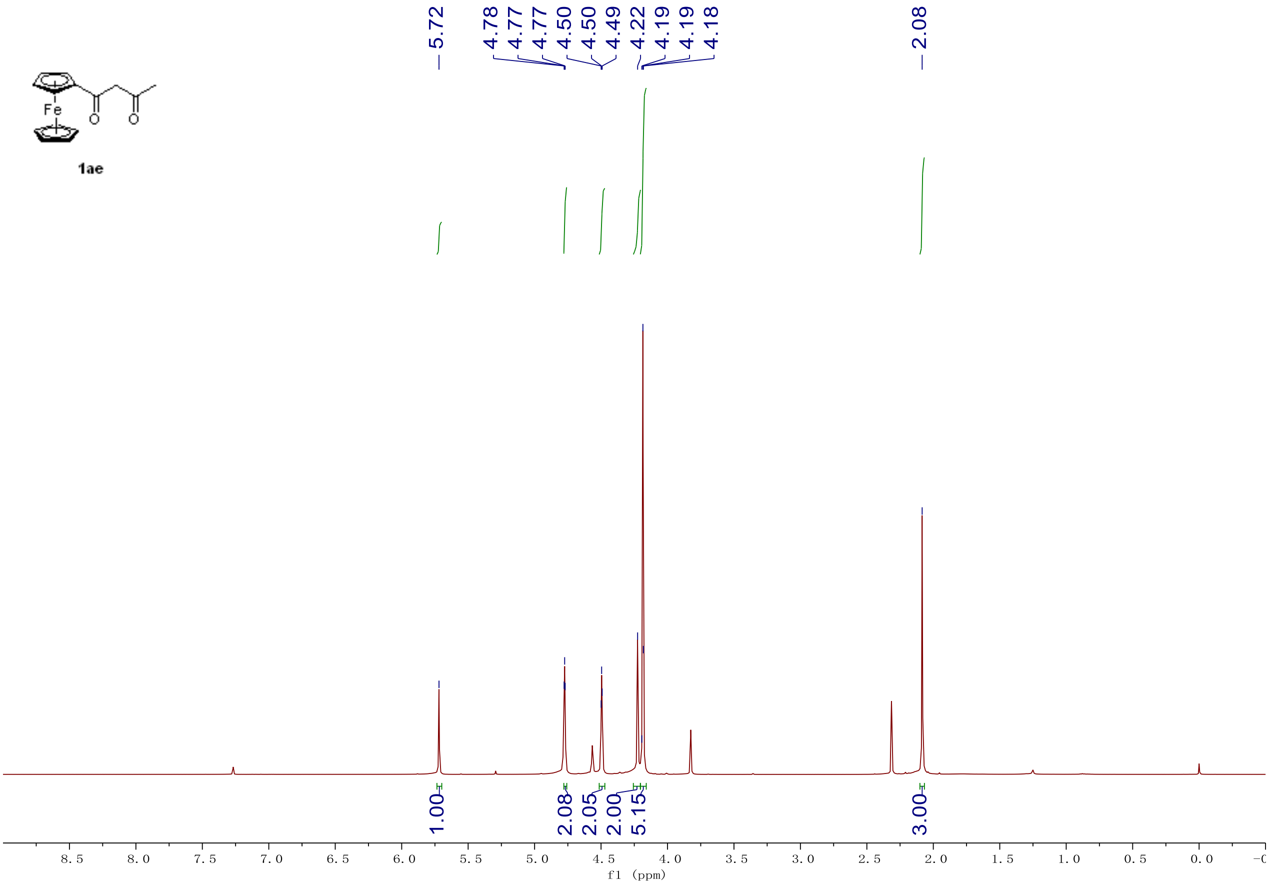

Supplementary Figure 11. ^{1}H NMR spectrum of **1ae**.

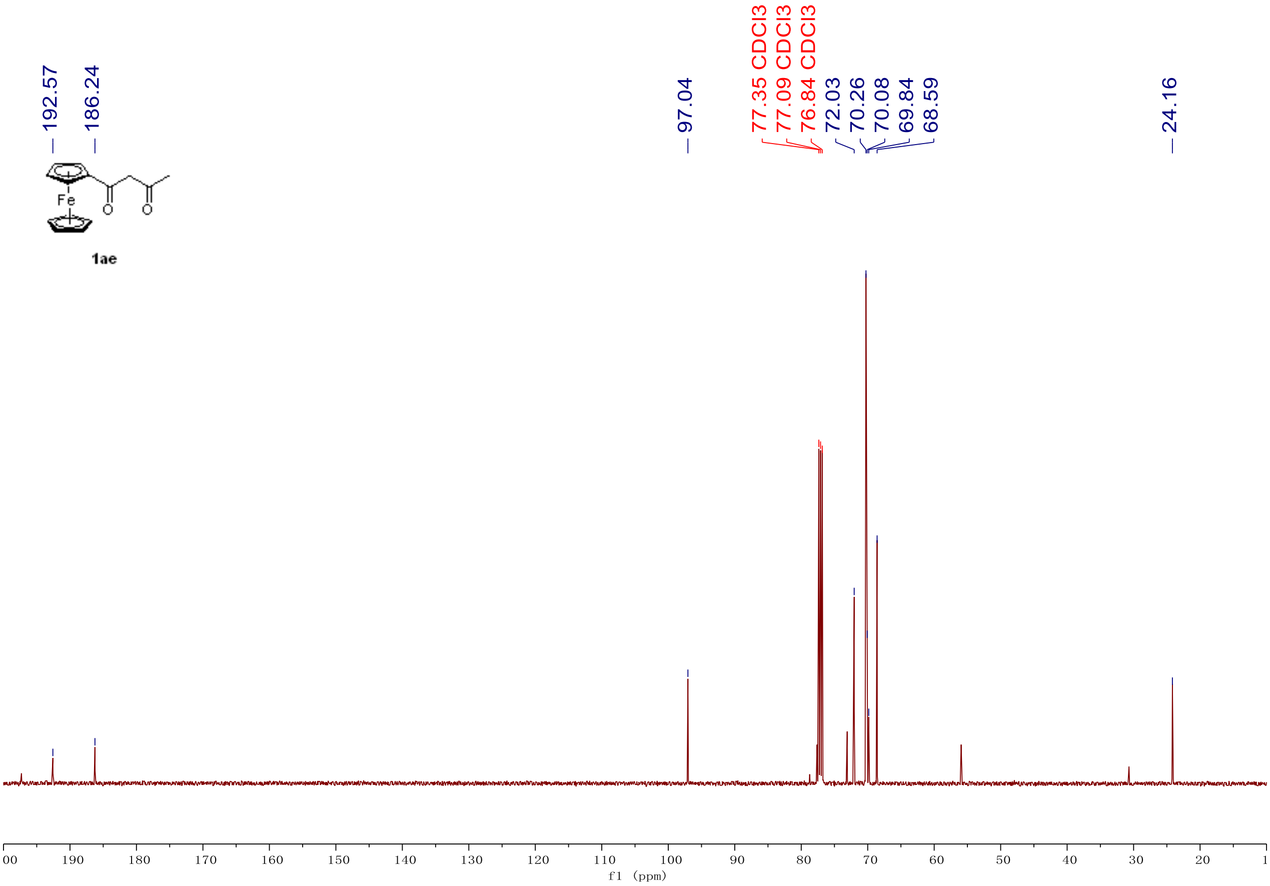

Supplementary Figure 12. ^{13}C NMR spectrum of **1ae**.

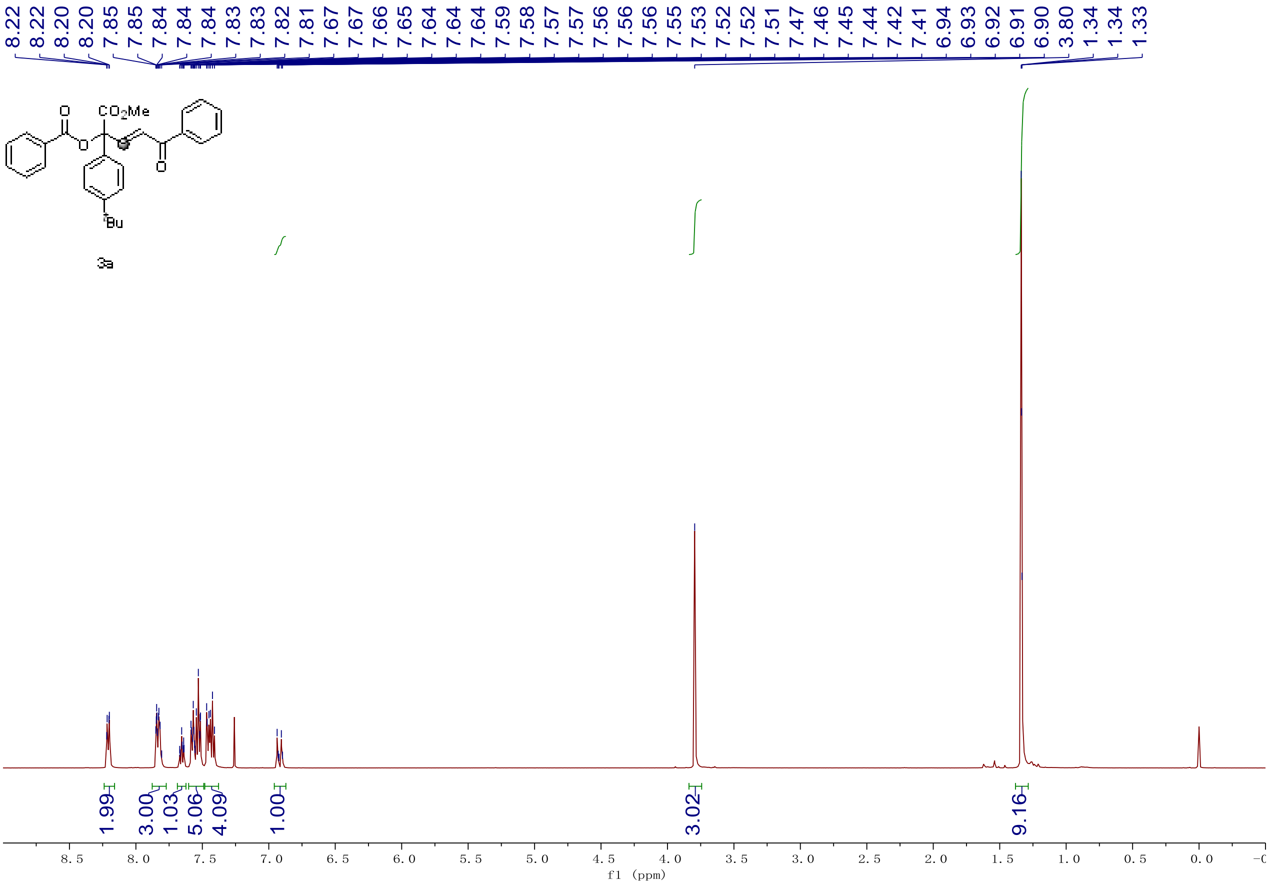

Supplementary Figure 13. ^{1}H NMR spectrum of **3a**.

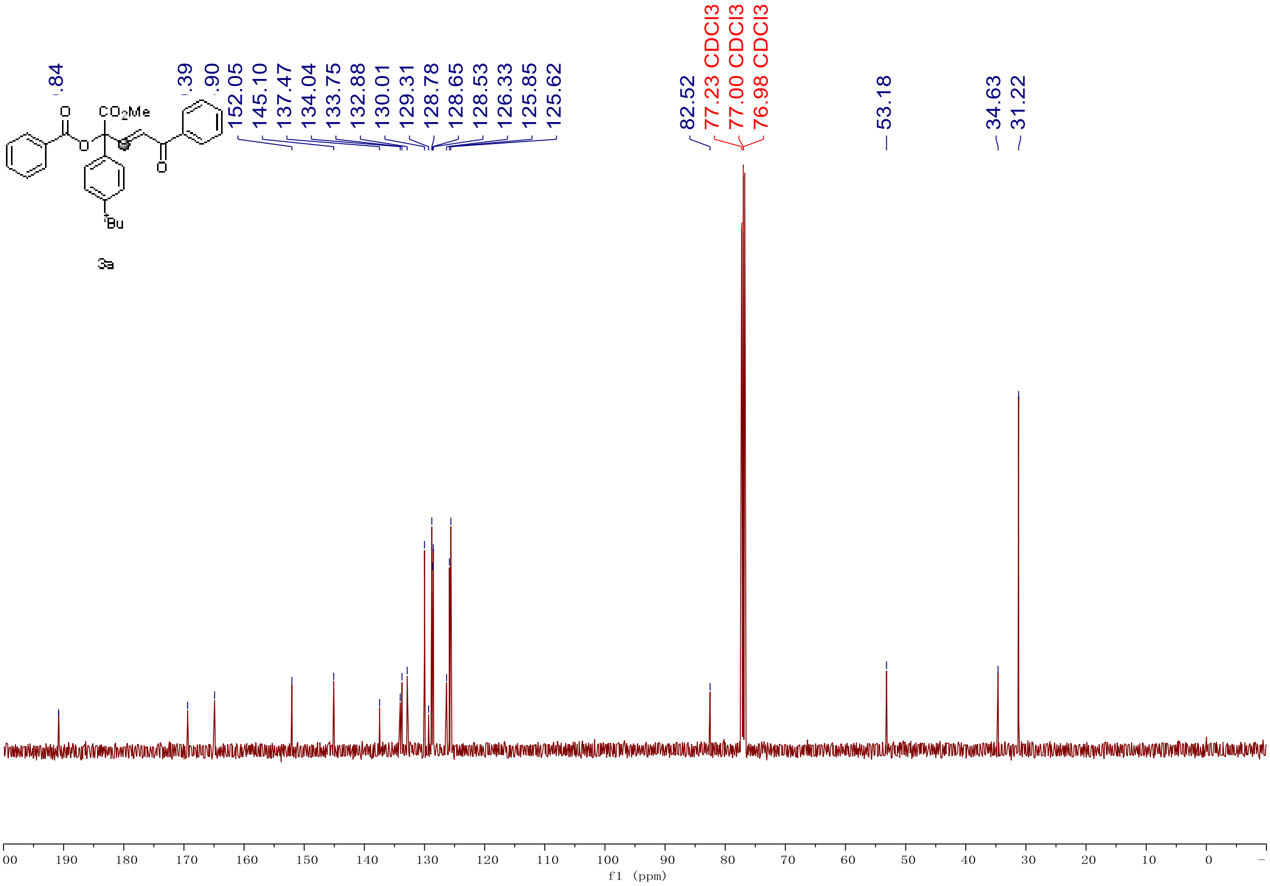

Supplementary Figure 14. ^{13}C NMR spectrum of **3a**.

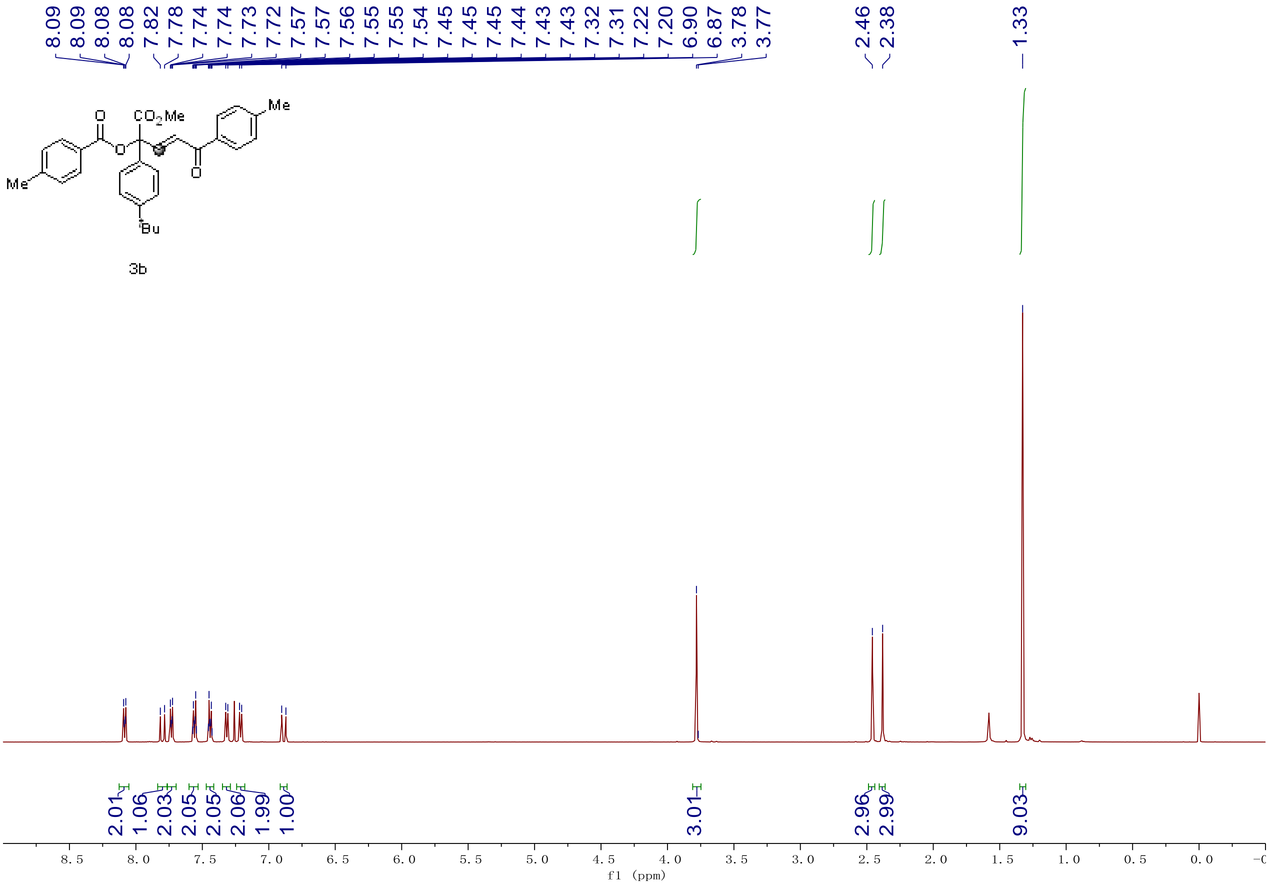

Supplementary Figure 15. ^{1}H NMR spectrum of **3b**.

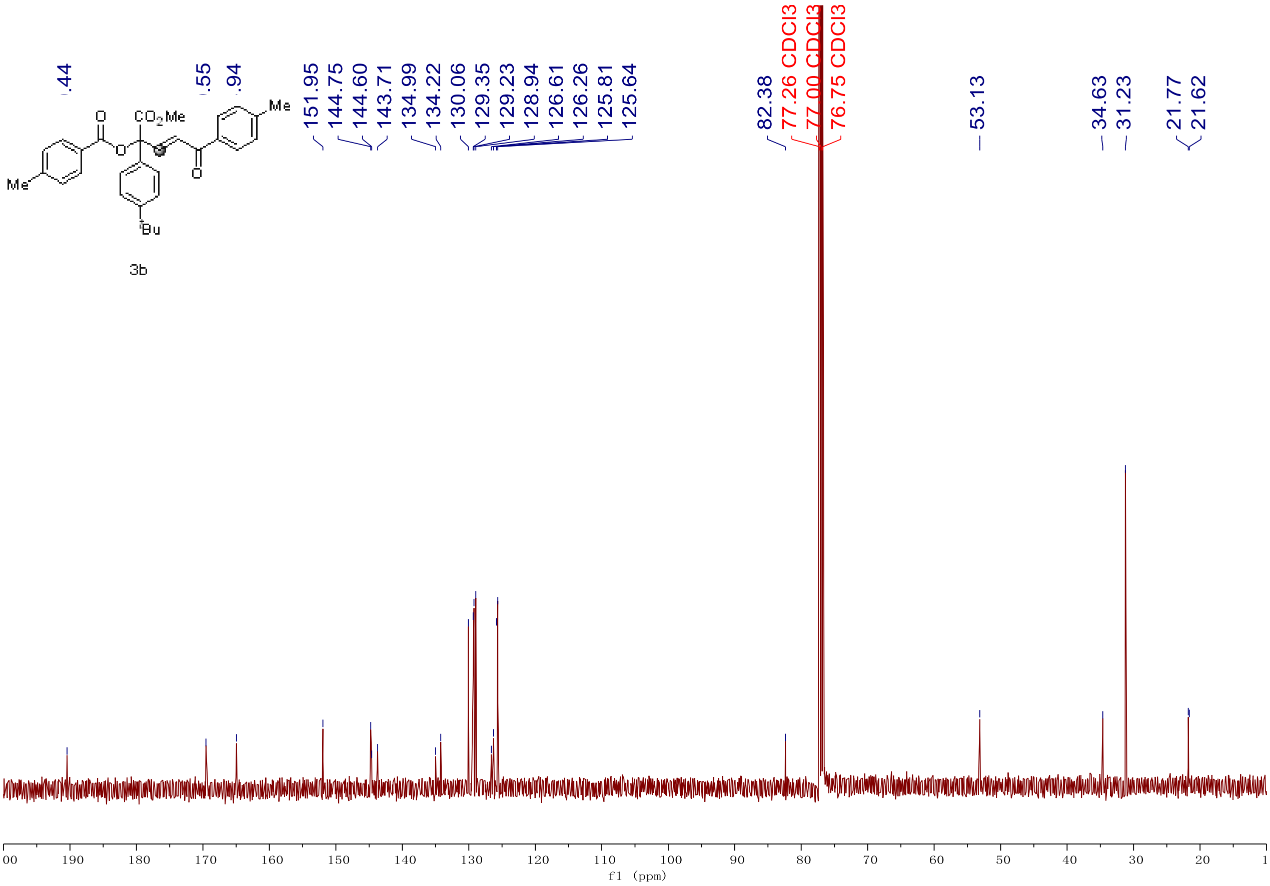

Supplementary Figure 16. ^{13}C NMR spectrum of **3b**.

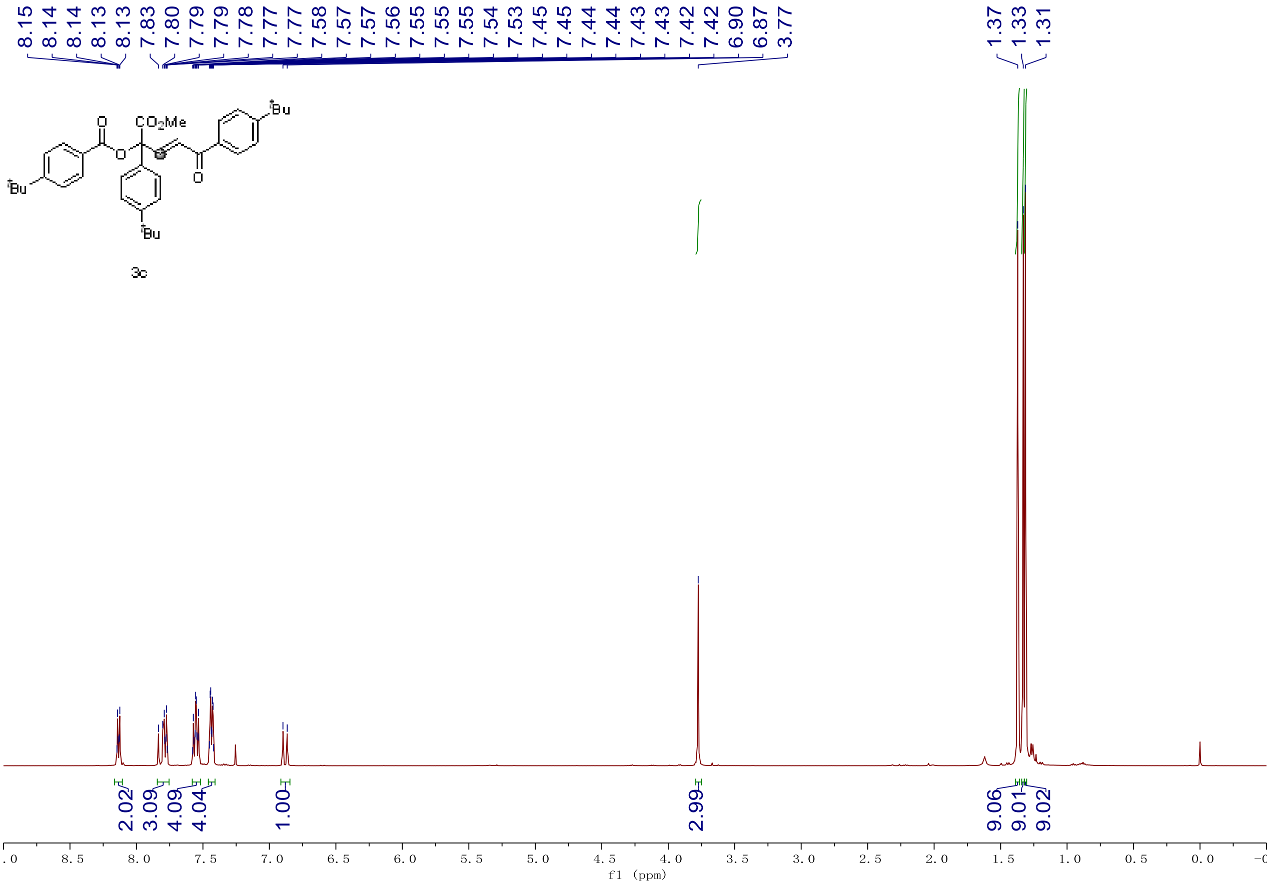

Supplementary Figure 17. ^{1}H NMR spectrum of **3c**.

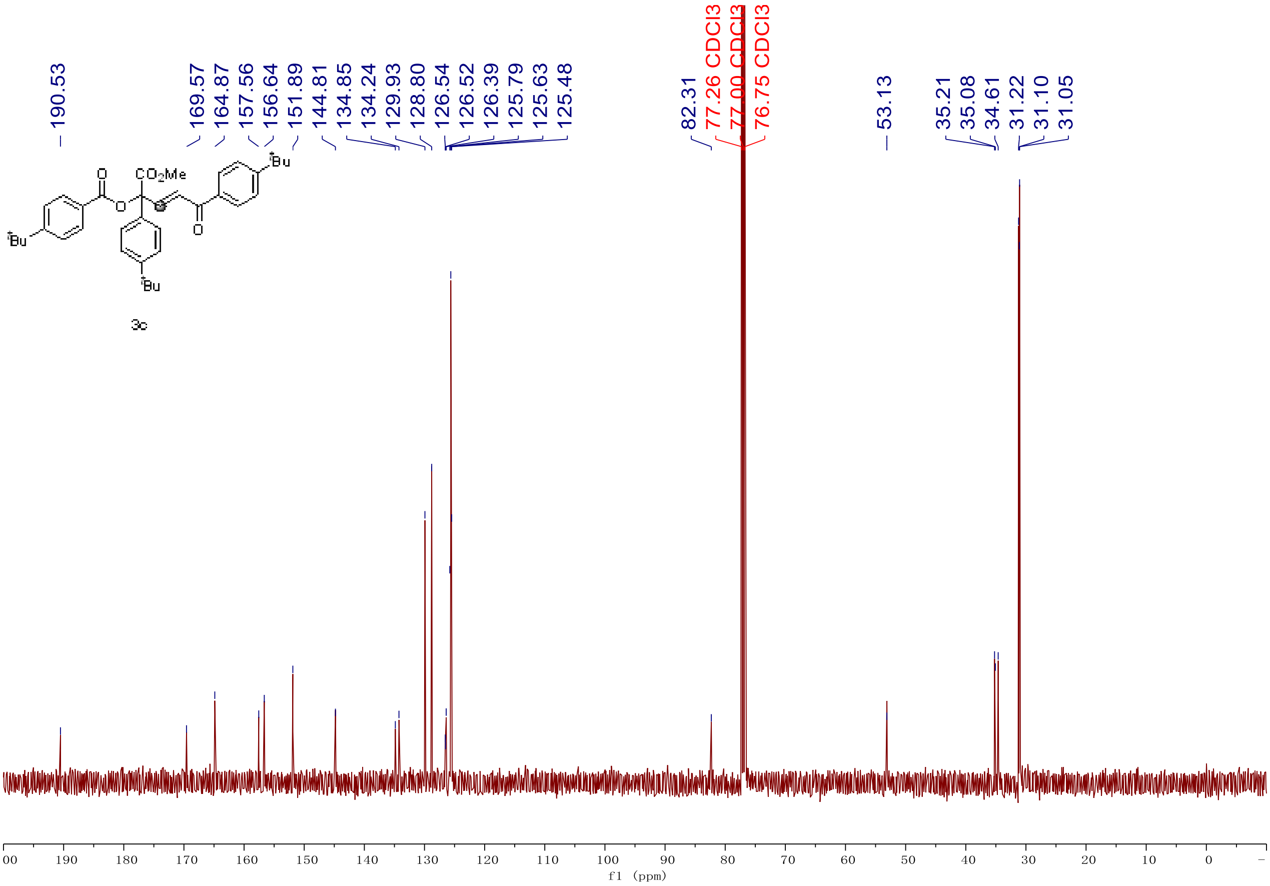

Supplementary Figure 18. ^{13}C NMR spectrum of **3c**.

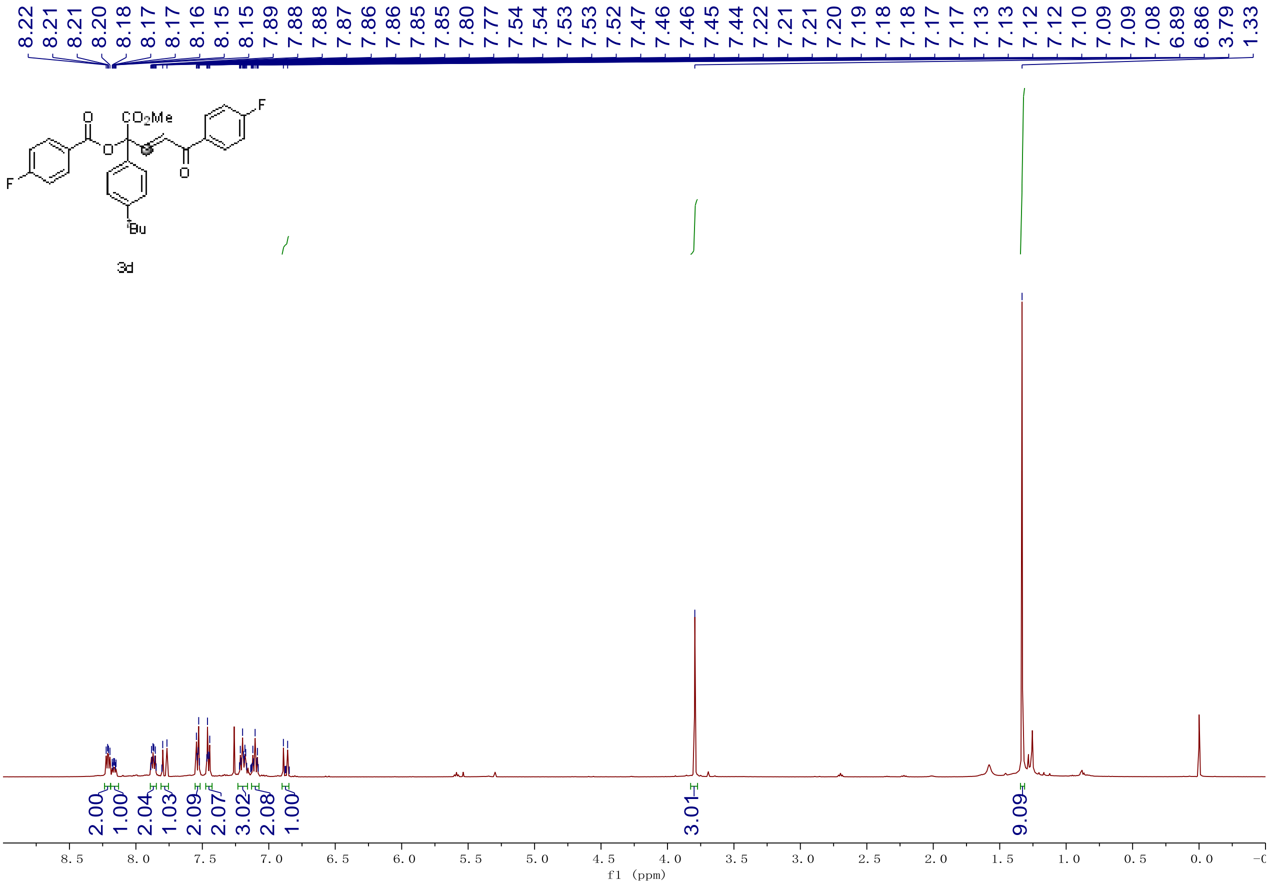

Supplementary Figure 19. ^{1}H NMR spectrum of **3d**.

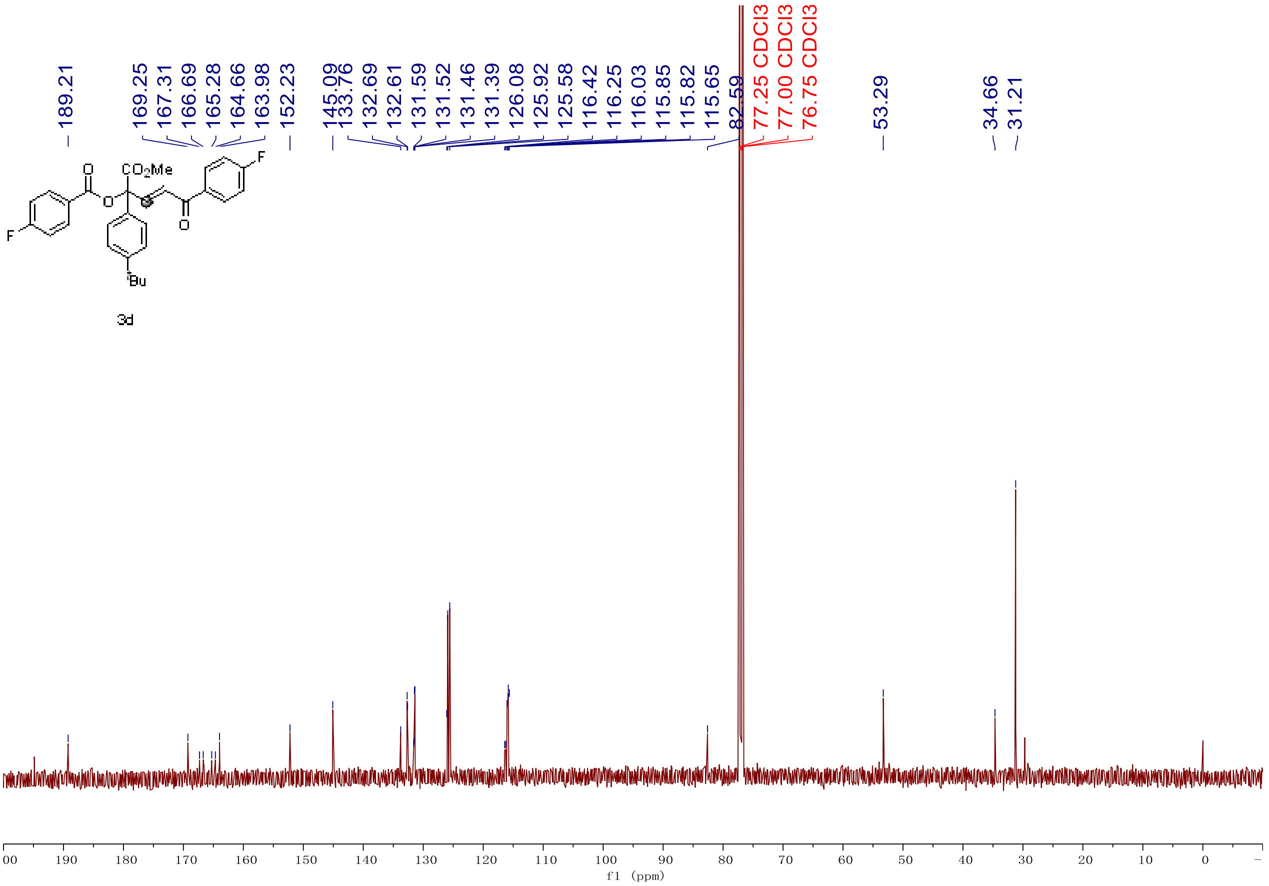

Supplementary Figure 20. ^{13}C NMR spectrum of **3d**.

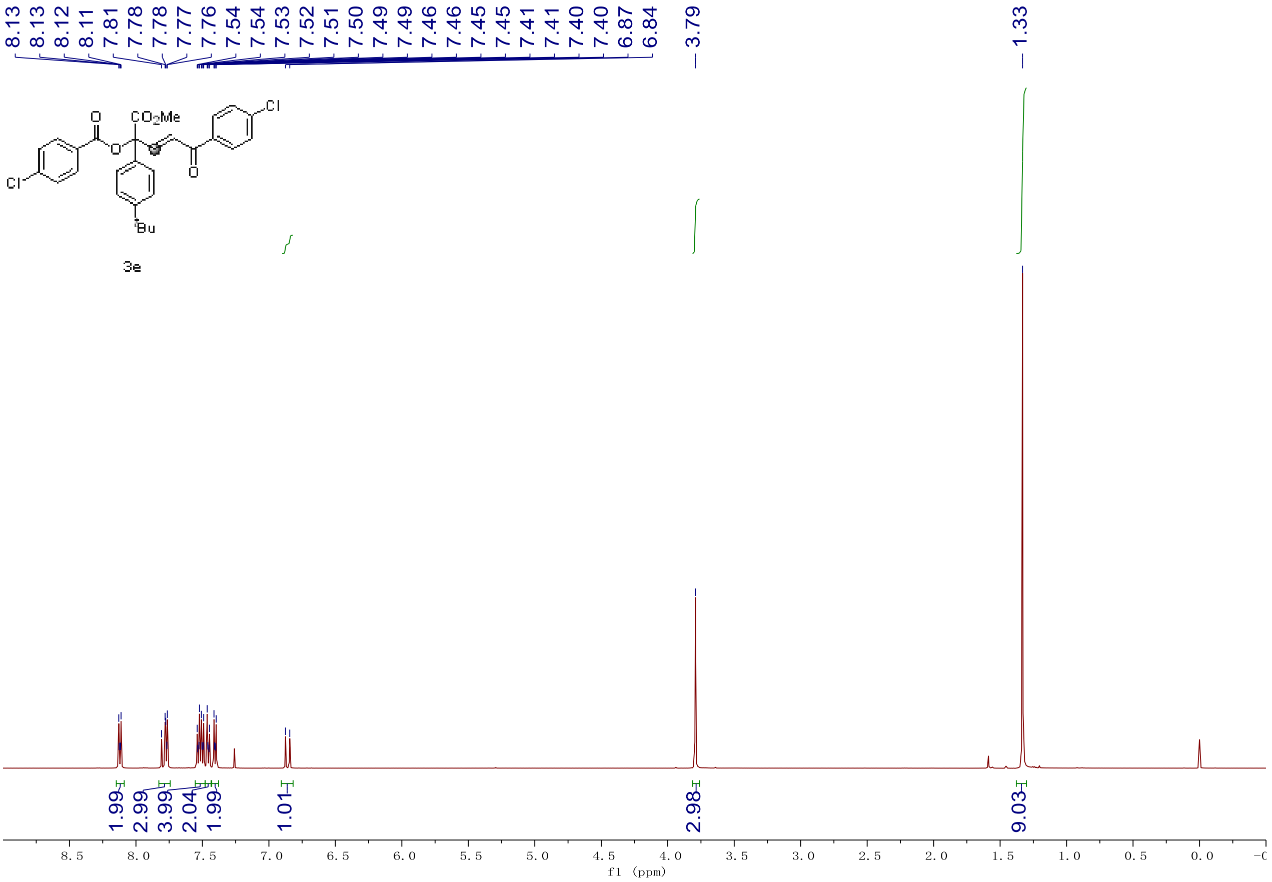

Supplementary Figure 21. ^{1}H NMR spectrum of **3e.**

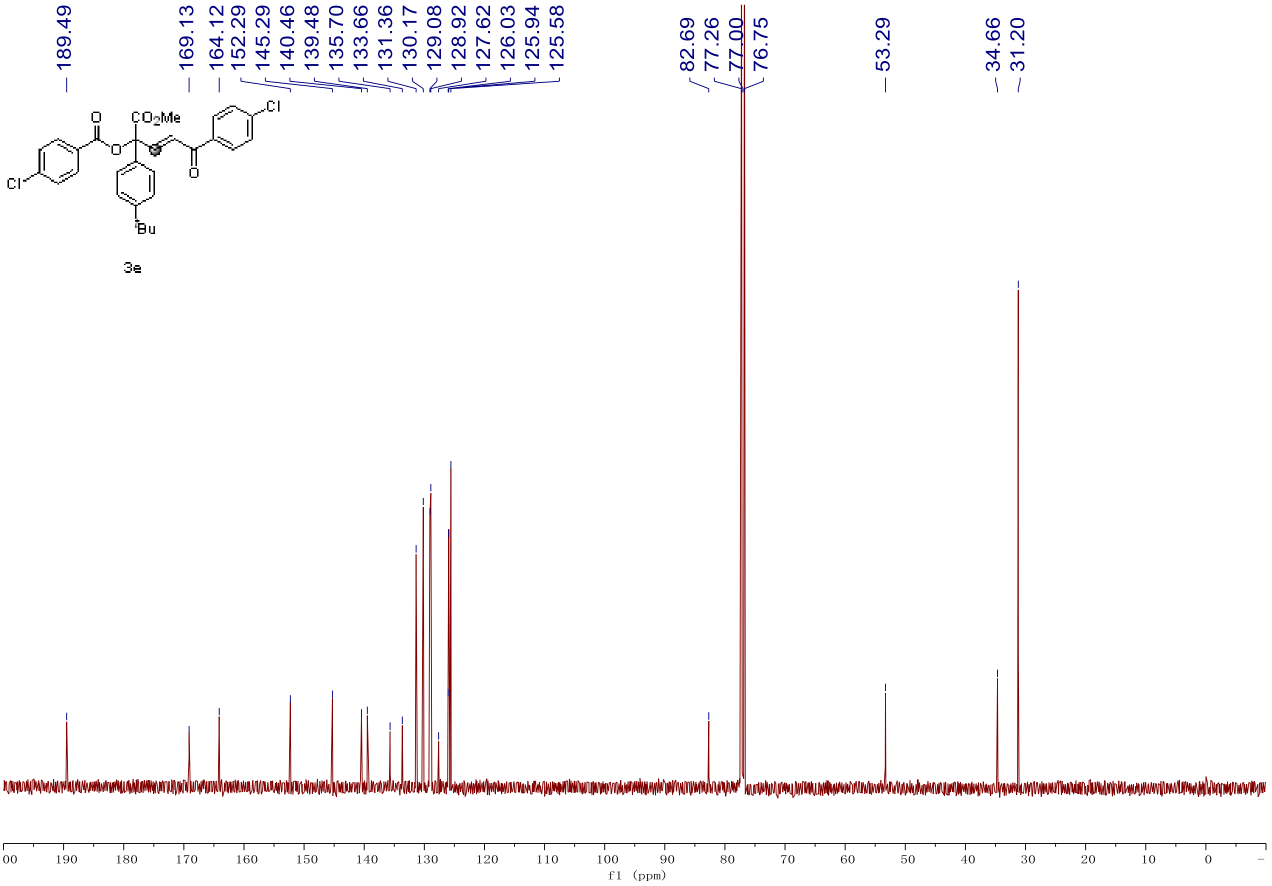

Supplementary Figure 22. ^{13}C NMR spectrum of **3e**.

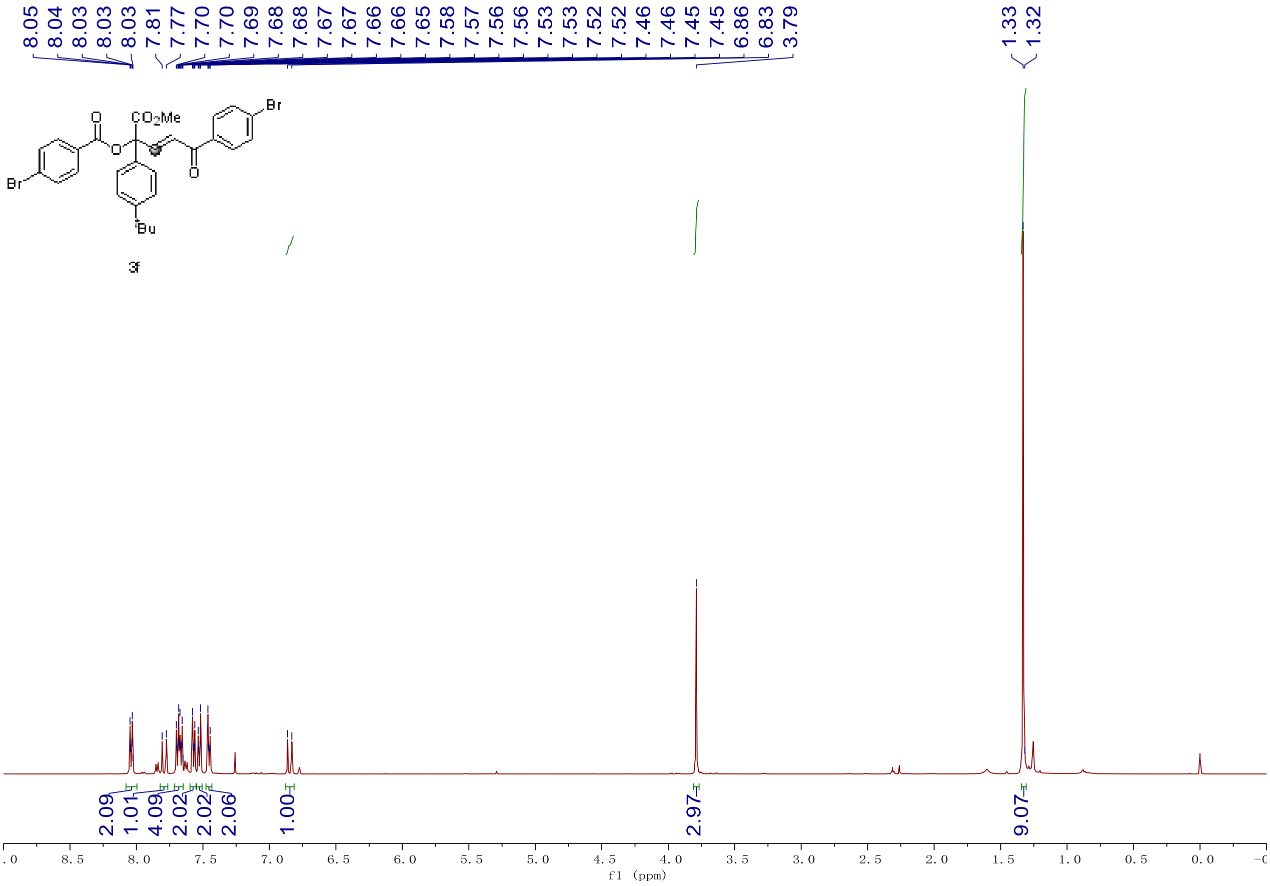

Supplementary Figure 23. ^{1}H NMR spectrum of **3f**.

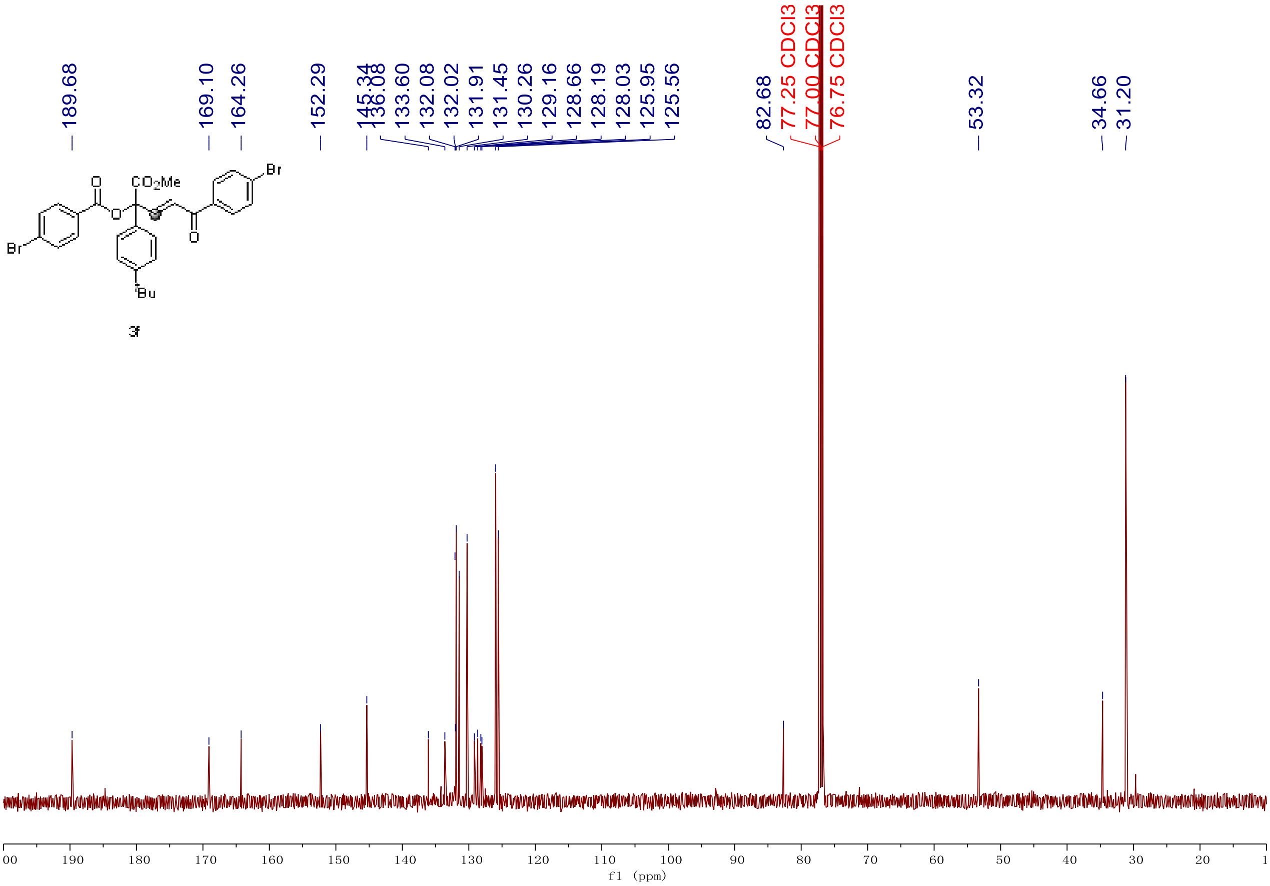

Supplementary Figure 24. ^{13}C NMR spectrum of **3f**.

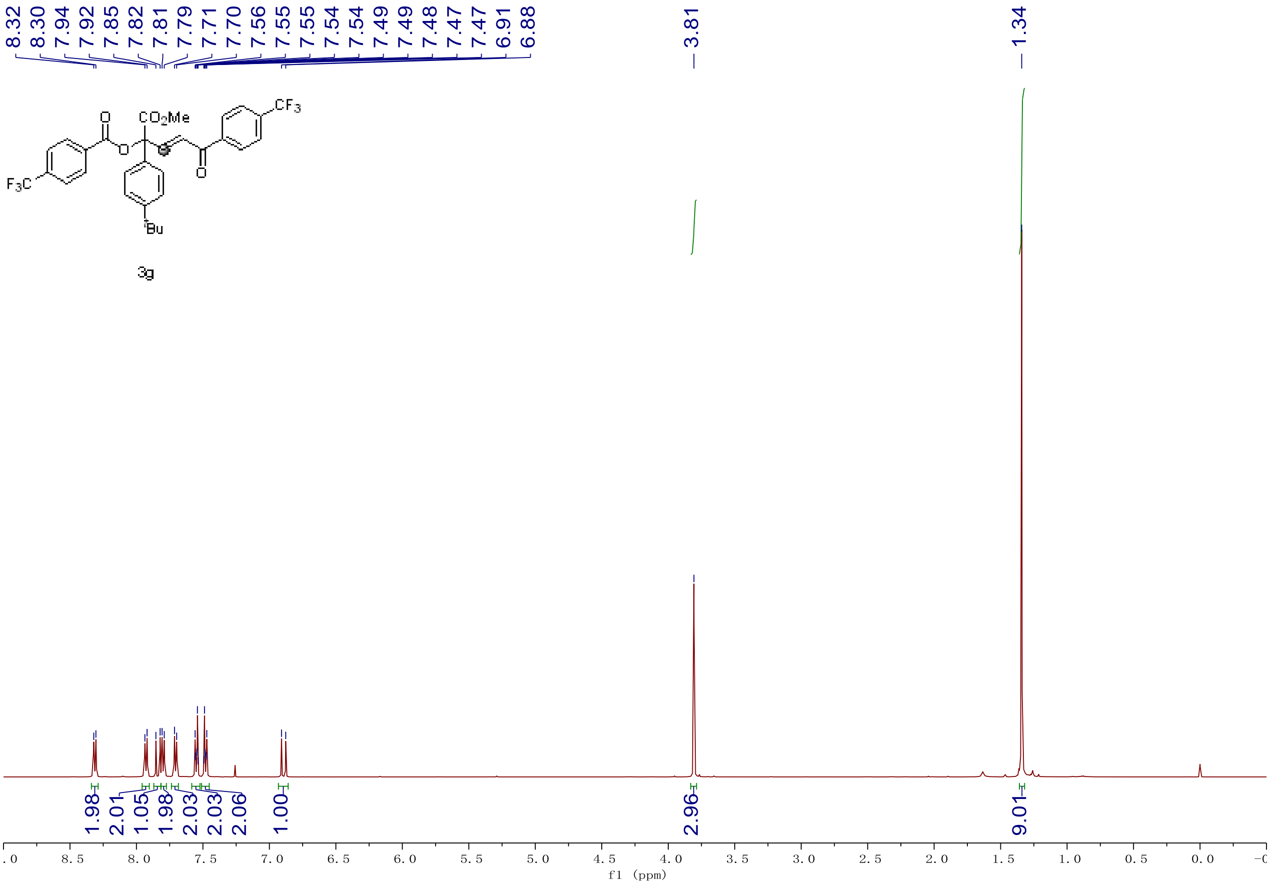

Supplementary Figure 25. ^{1}H NMR spectrum of **3g**.

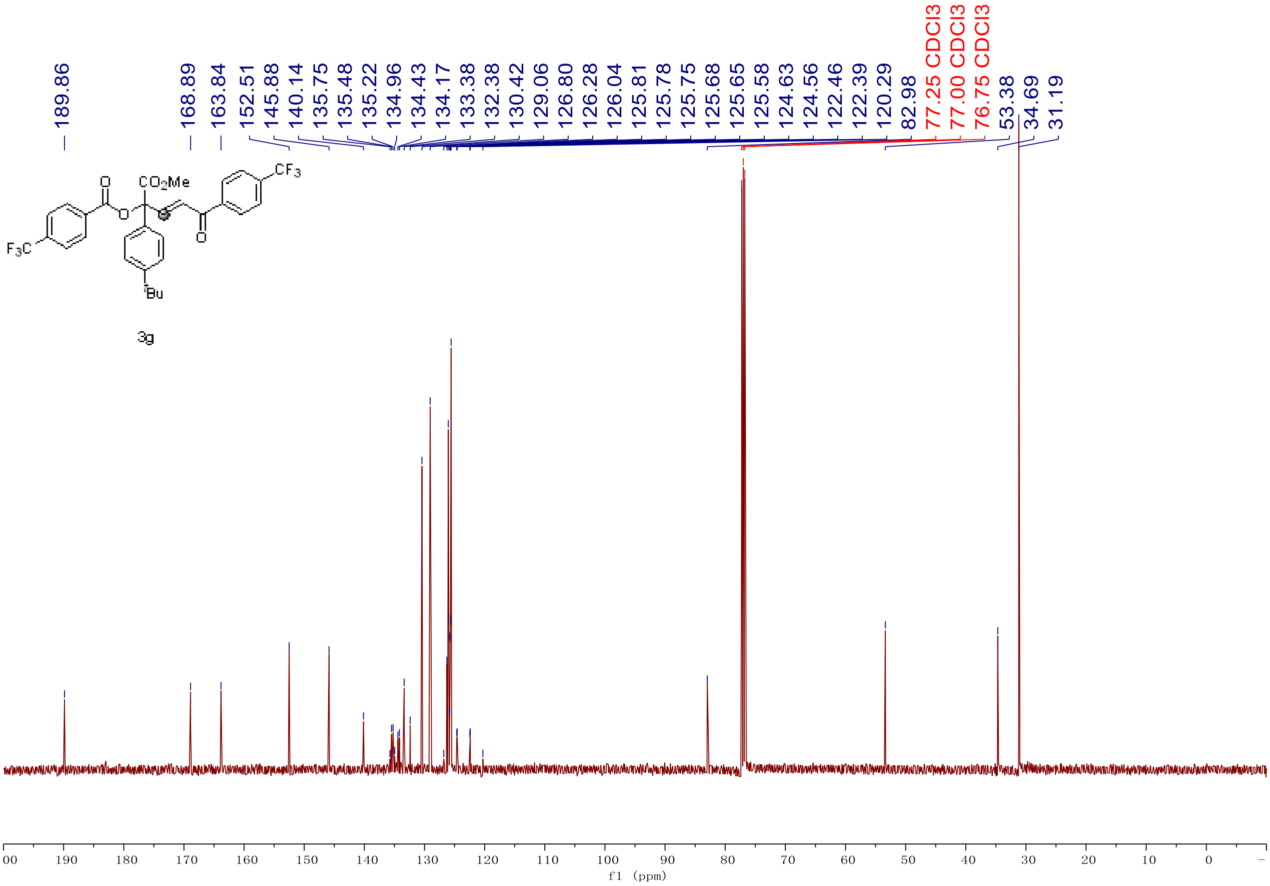

Supplementary Figure 26. ^{13}C NMR spectrum of **3g**.

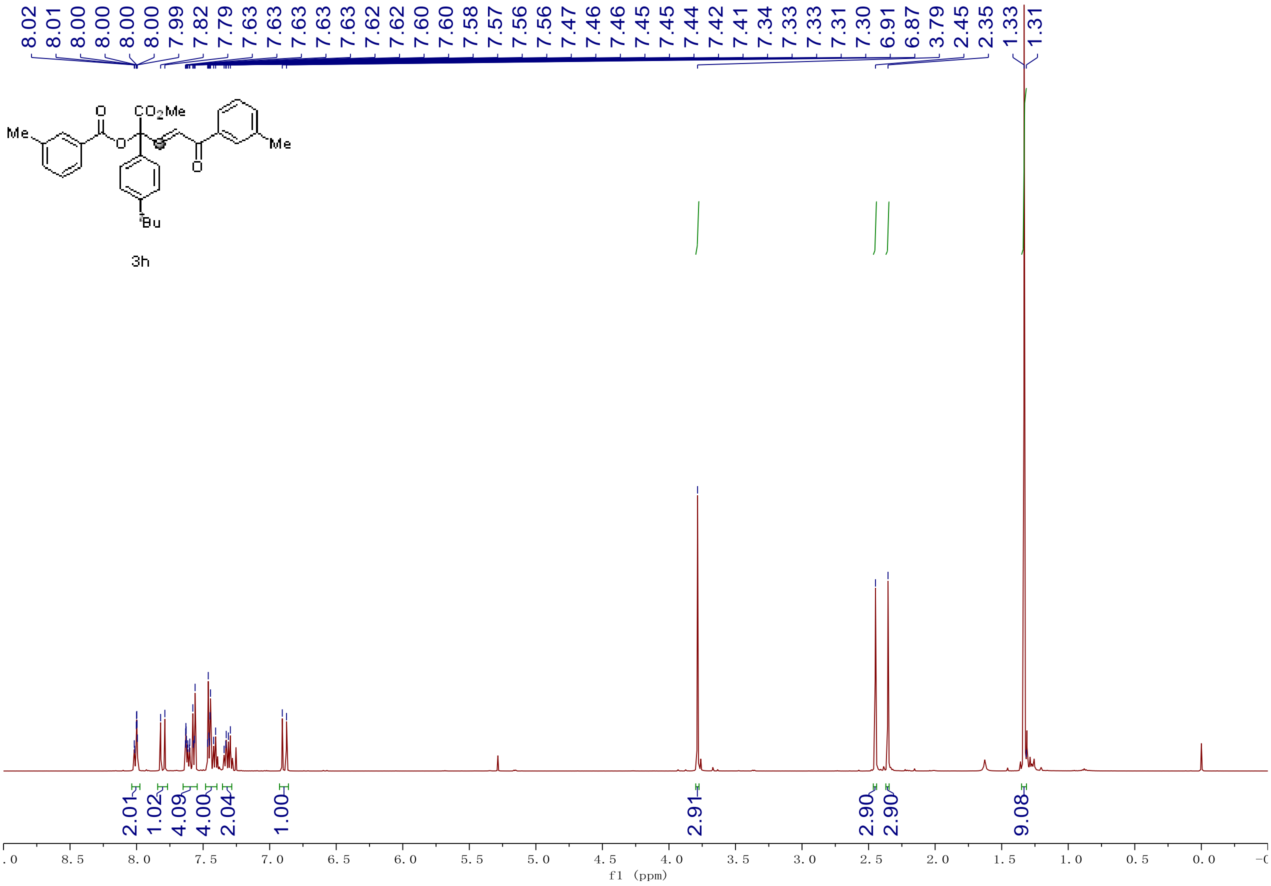

Supplementary Figure 27. ^{1}H NMR spectrum of **3h**.

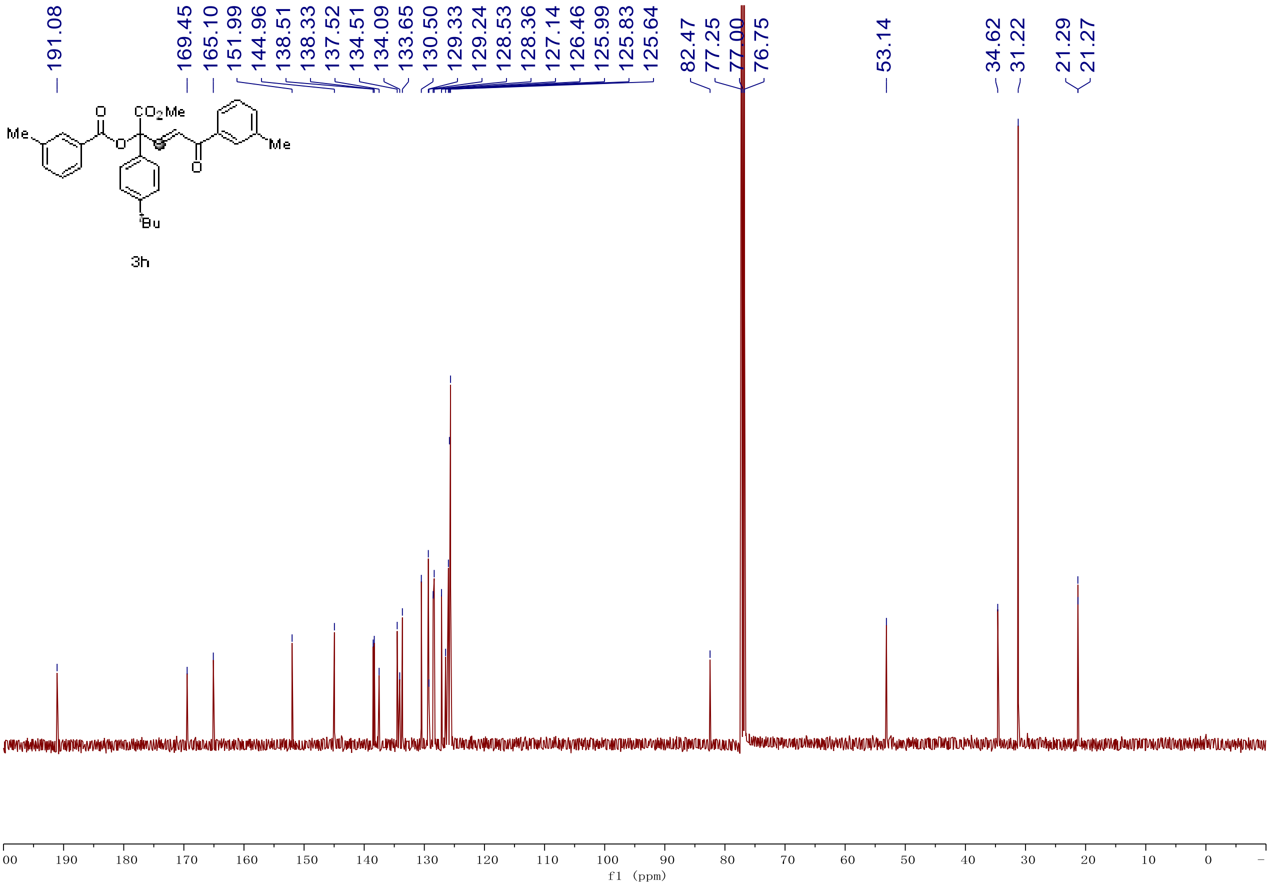

Supplementary Figure 28. ^{13}C NMR spectrum of **3h**.

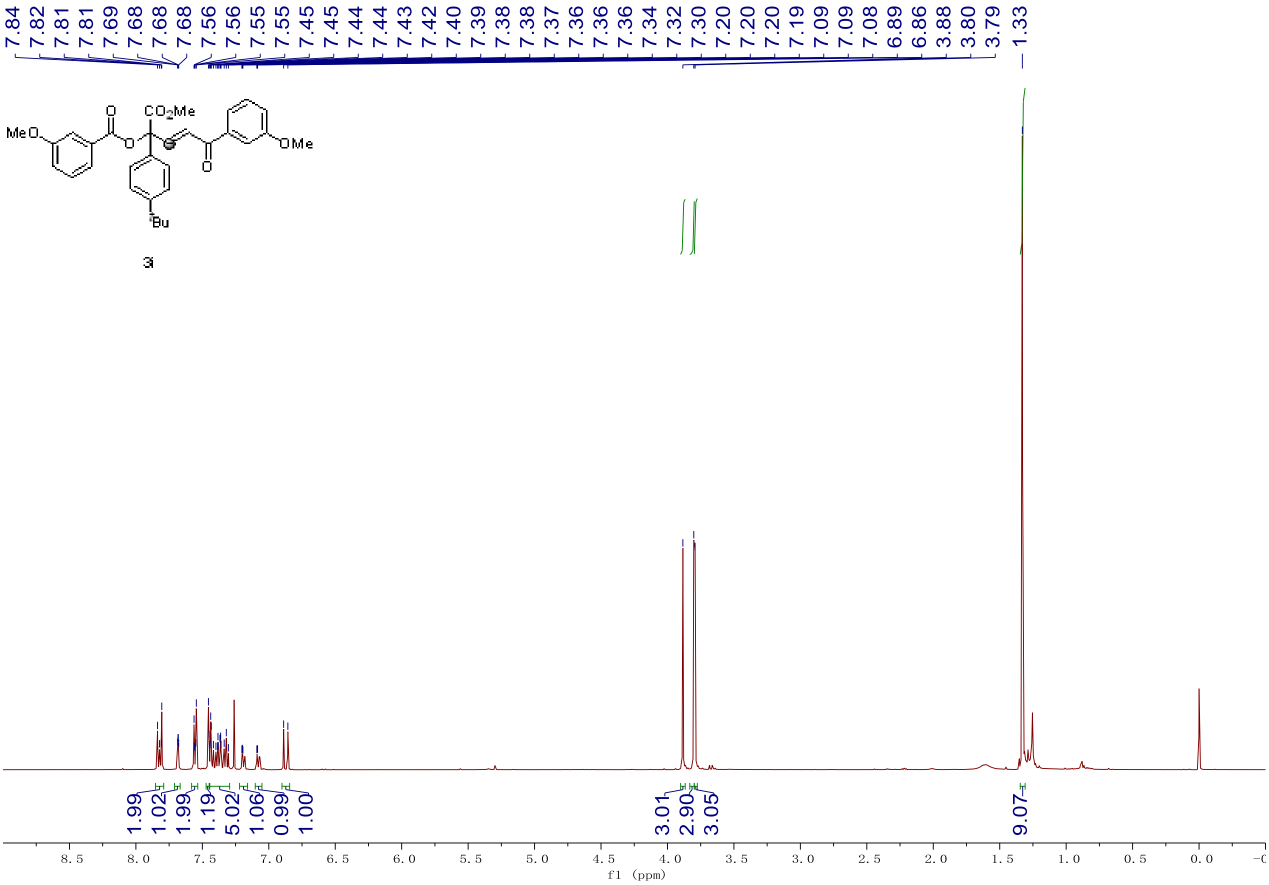

Supplementary Figure 29. ^{1}H NMR spectrum of **3i**.

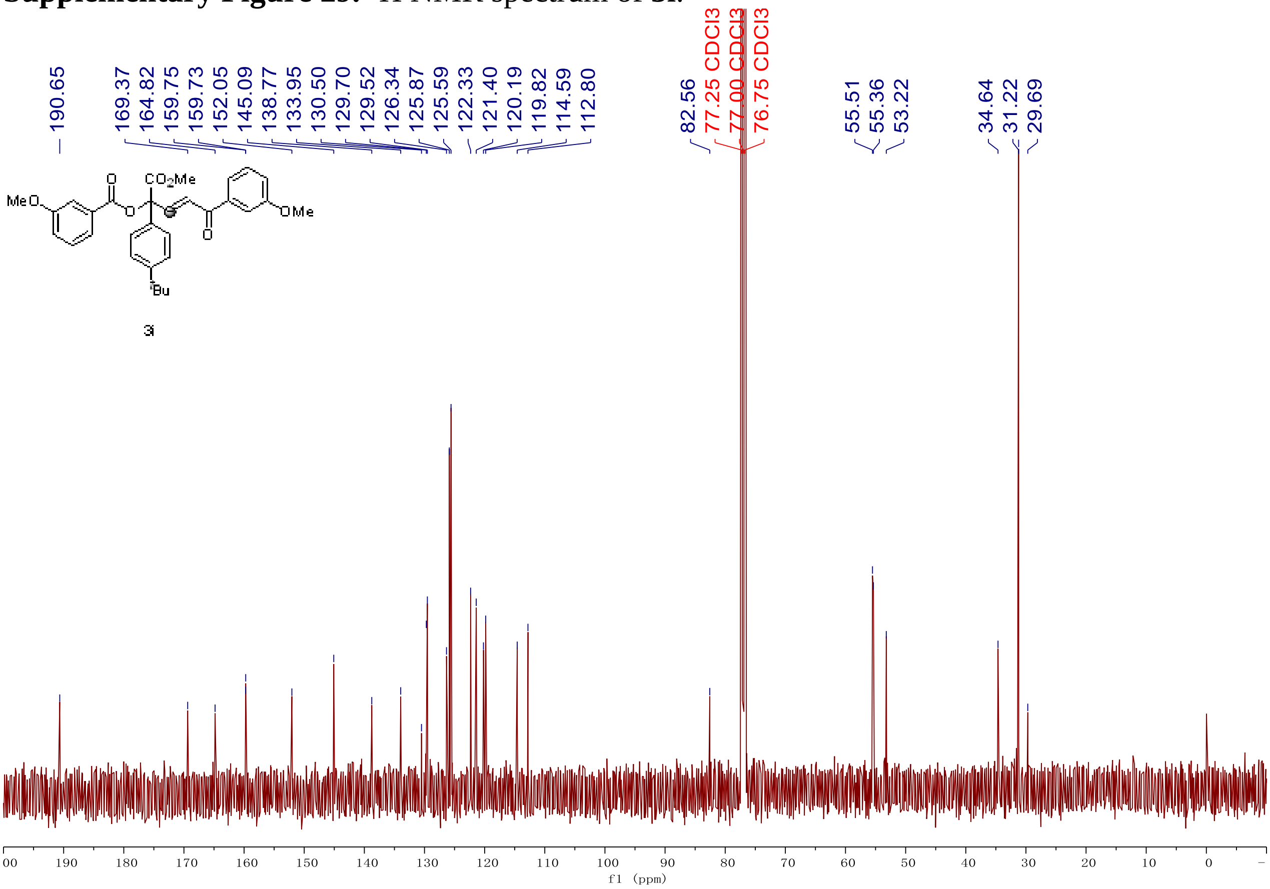

Supplementary Figure 30. ^{13}C NMR spectrum of **3i**.

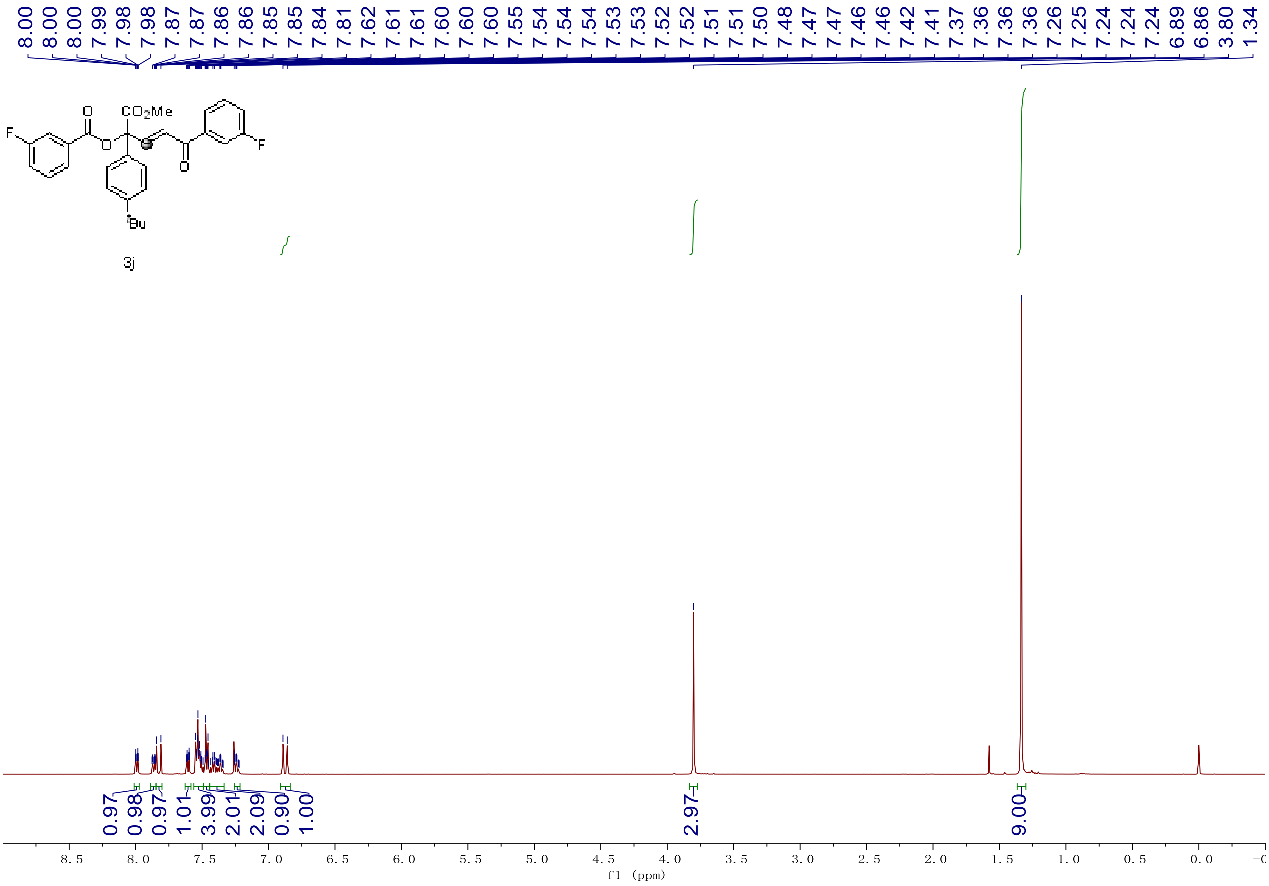

Supplementary Figure 31. ^{1}H NMR spectrum of **3j**.

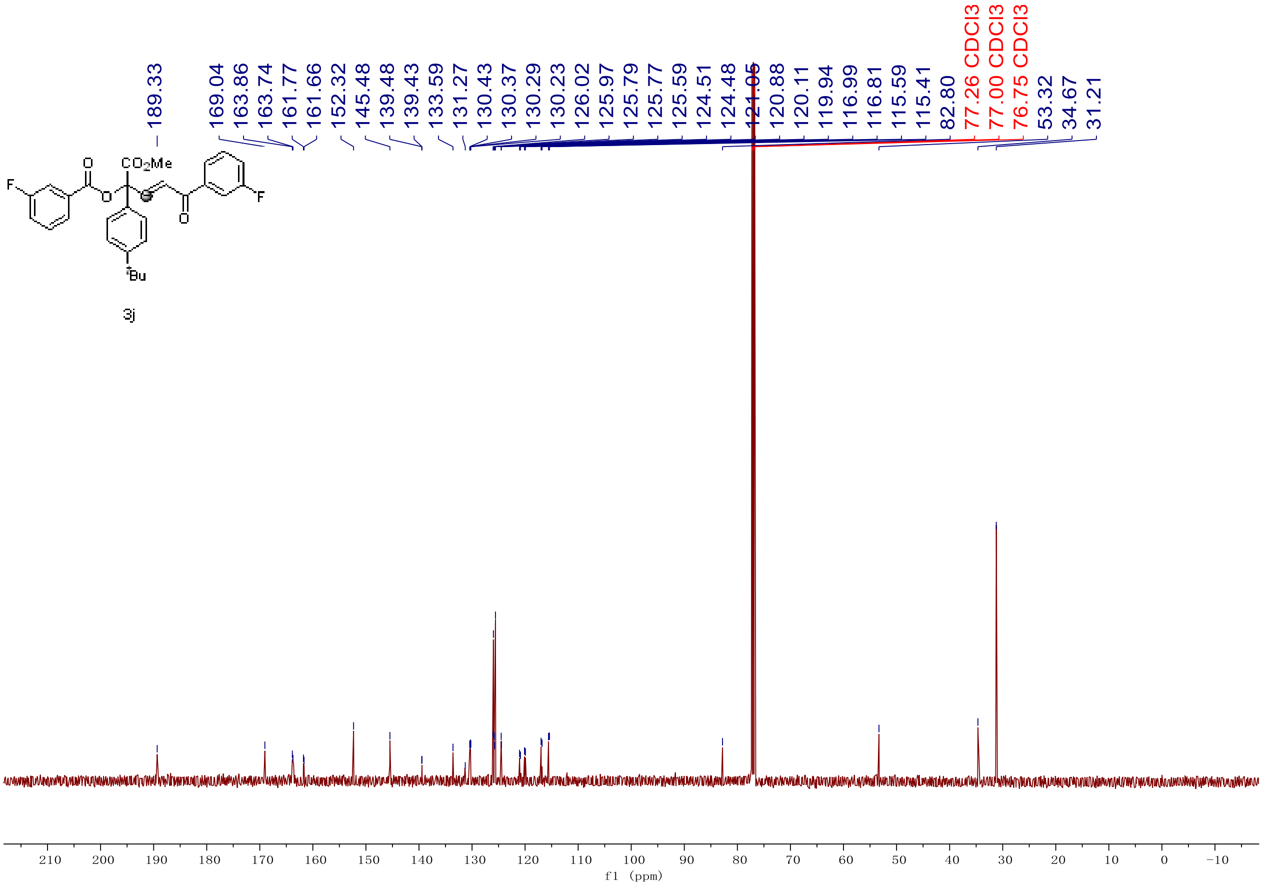

Supplementary Figure 32. ^{13}C NMR spectrum of **3j**.

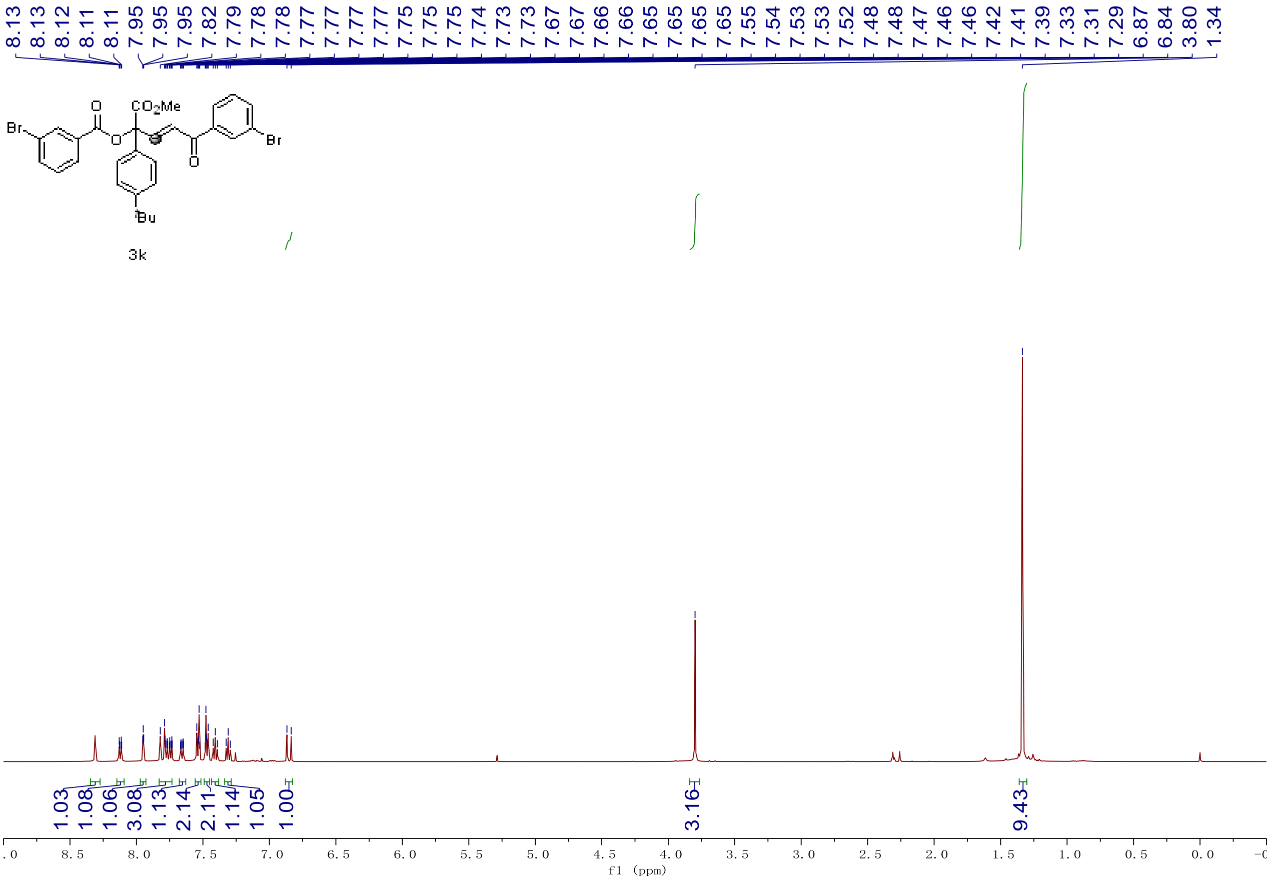

Supplementary Figure 33. ^{1}H NMR spectrum of **3k**.

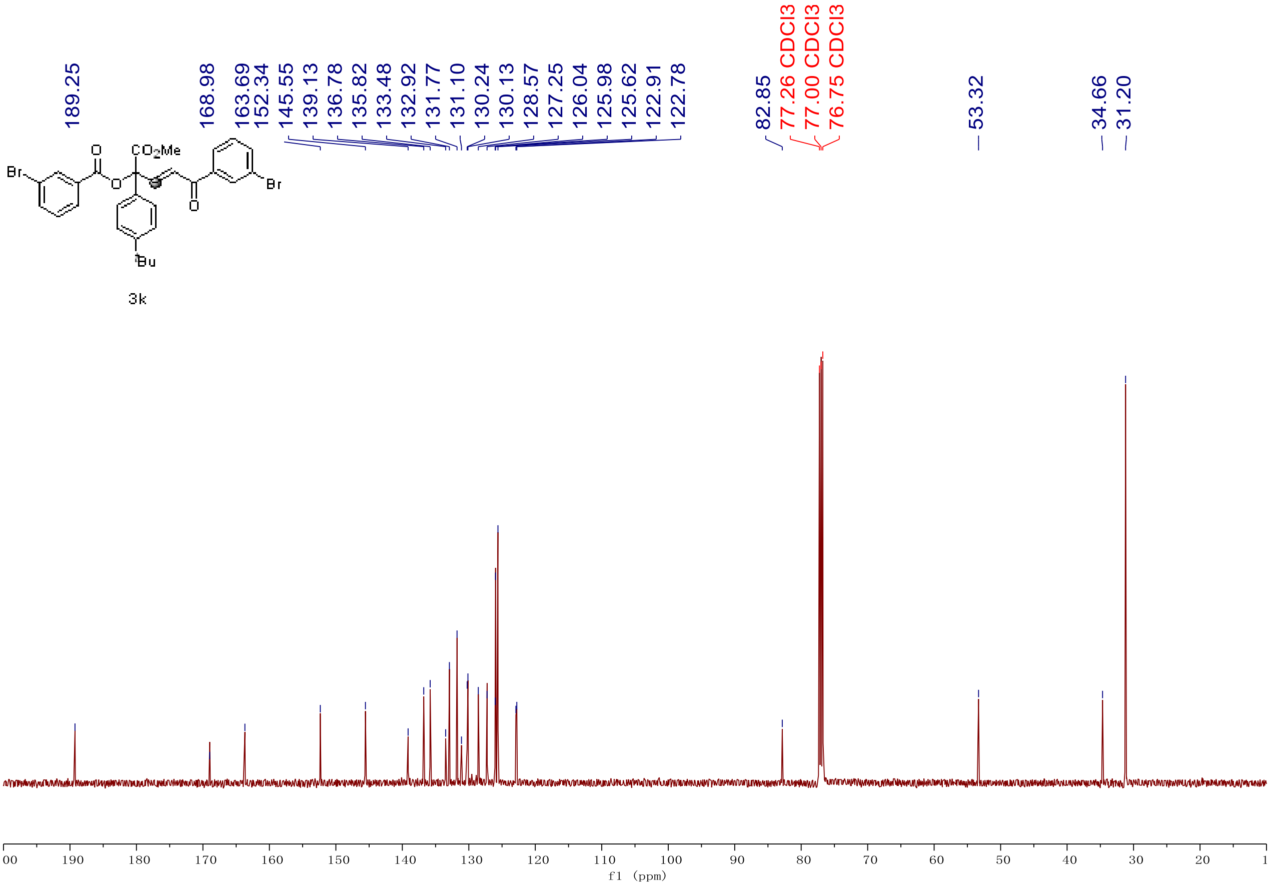

Supplementary Figure 34. ^{13}C NMR spectrum of **3k**.

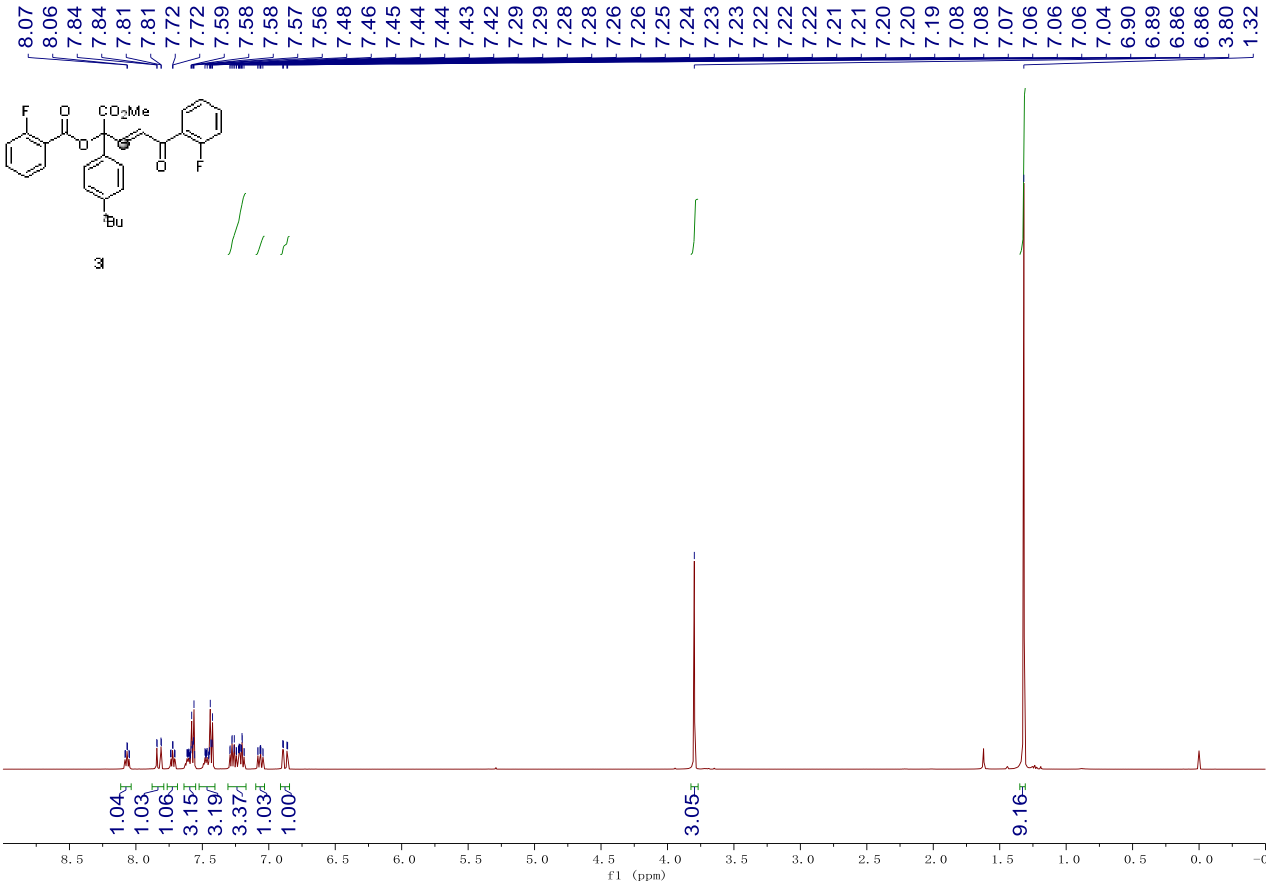

Supplementary Figure 35. ^{1}H NMR spectrum of **3l**.

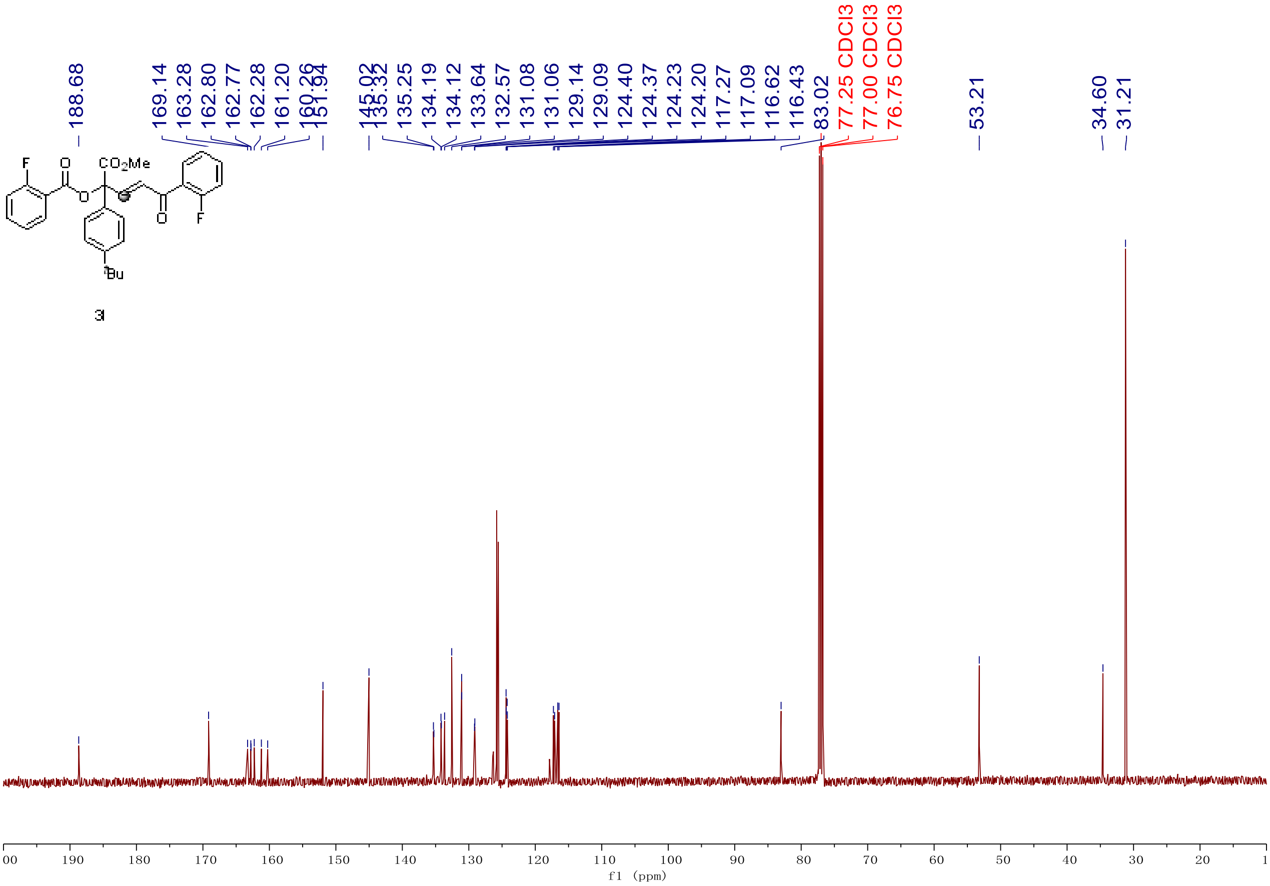

Supplementary Figure 36. ^{13}C NMR spectrum of **3l**.

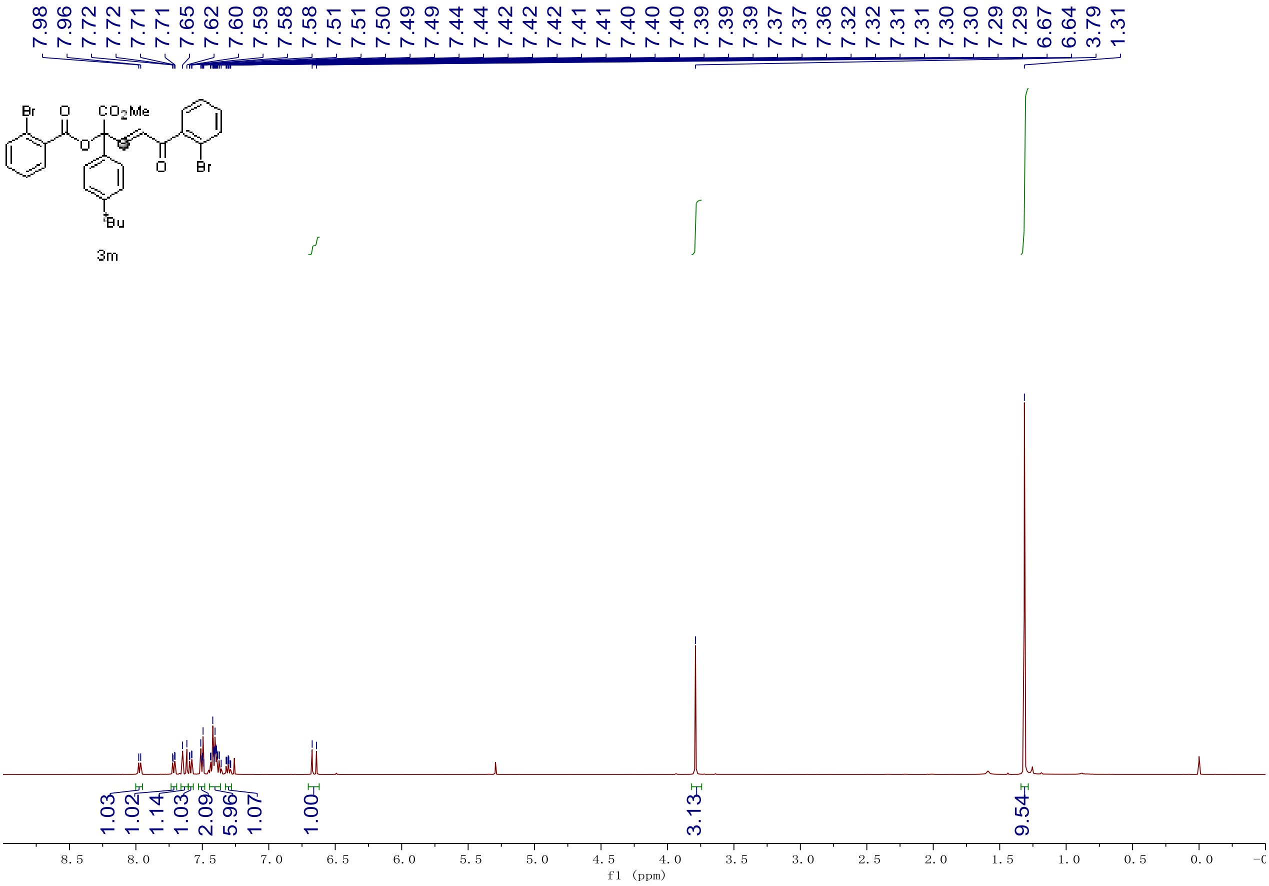

Supplementary Figure 37. ^{1}H NMR spectrum of **3m**.

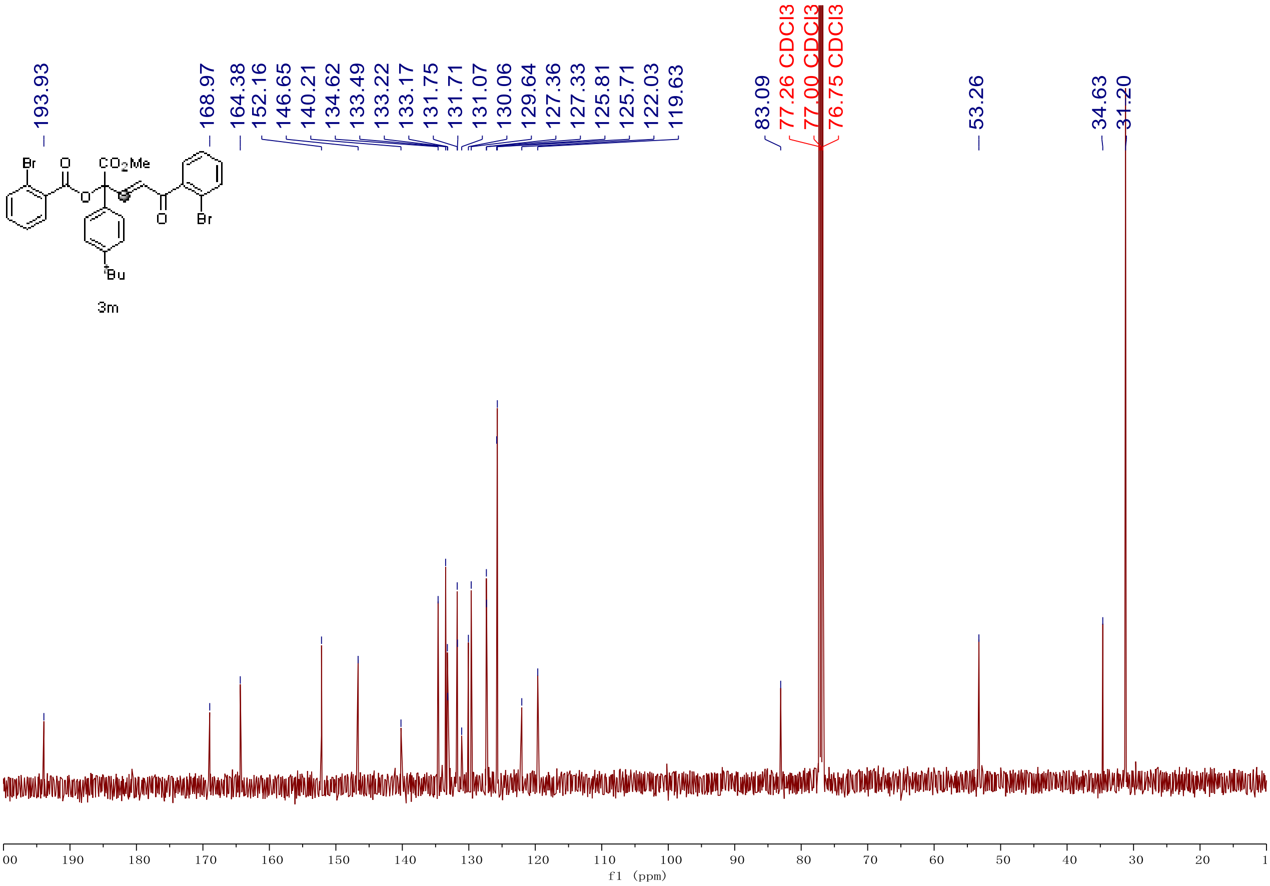

Supplementary Figure 38. ^{13}C NMR spectrum of **3m**.

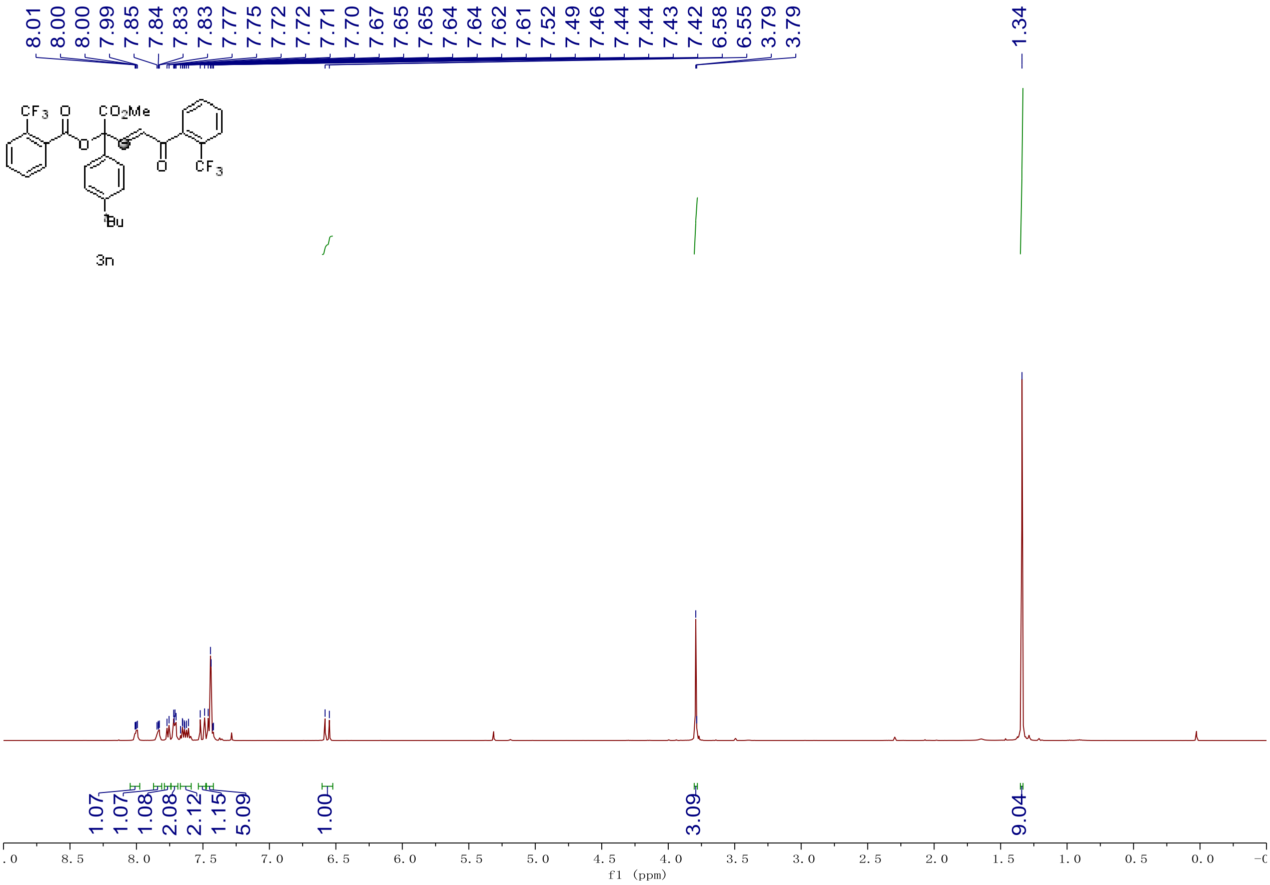

Supplementary Figure 39. ^{1}H NMR spectrum of **3n**.

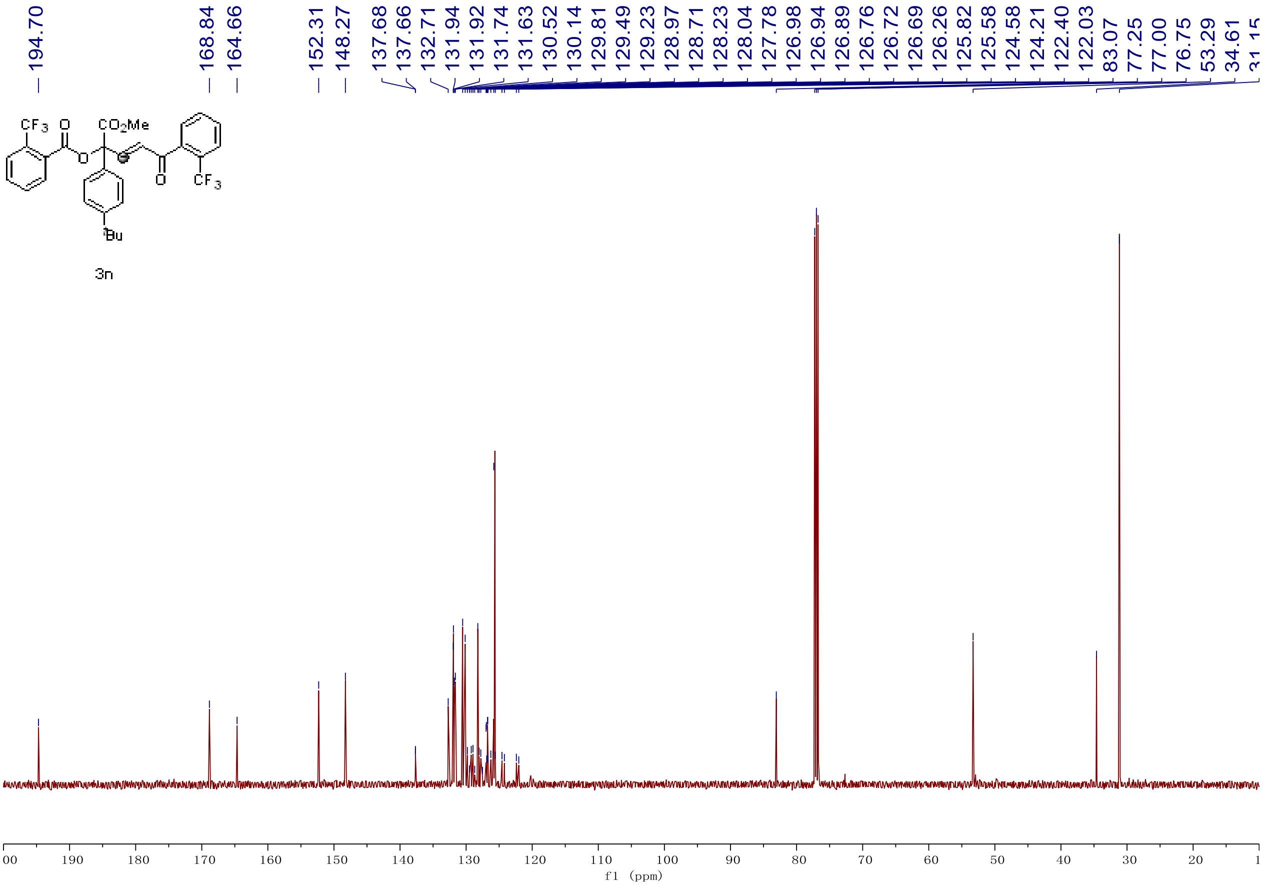

Supplementary Figure 40. ^{13}C NMR spectrum of **3n**.

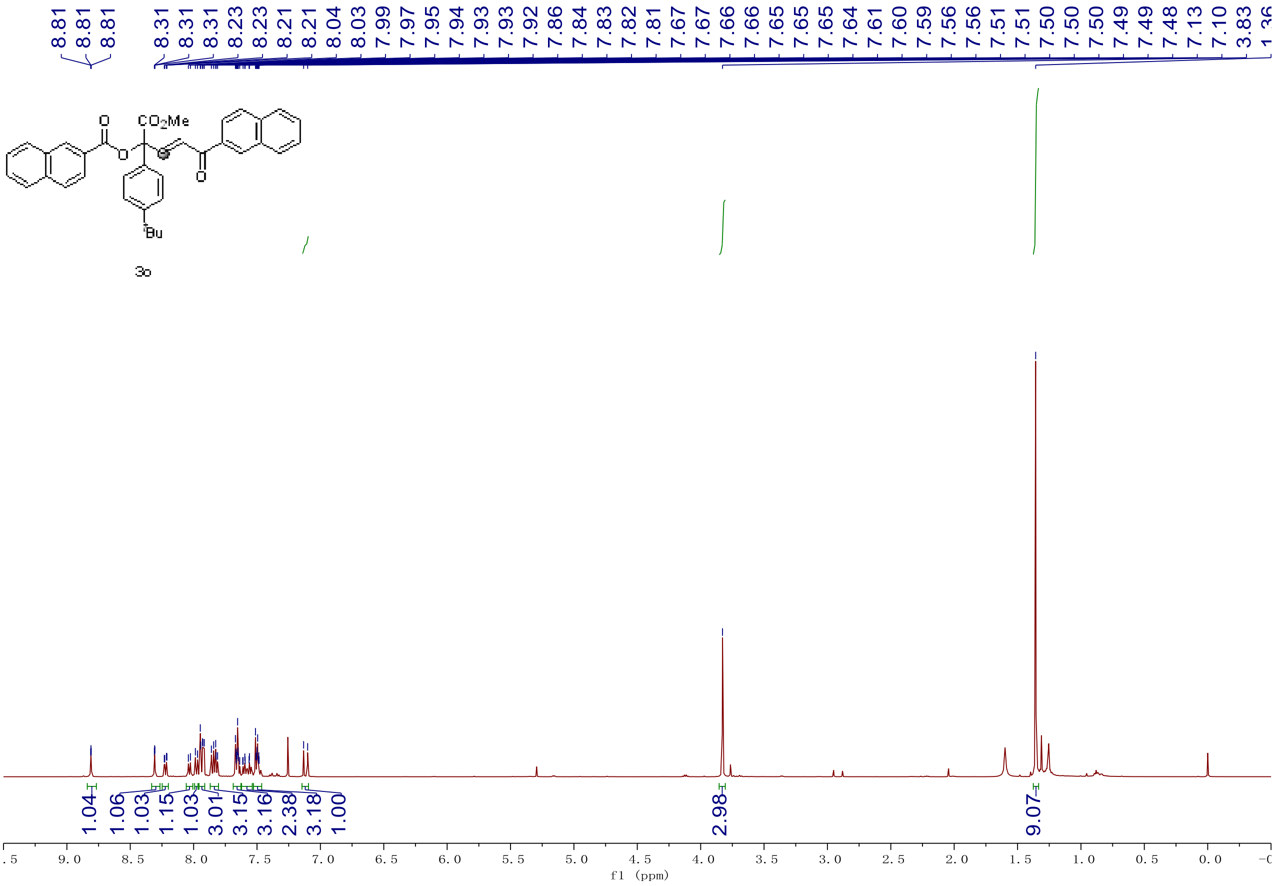

Supplementary Figure 41. ^{1}H NMR spectrum of **3o**.

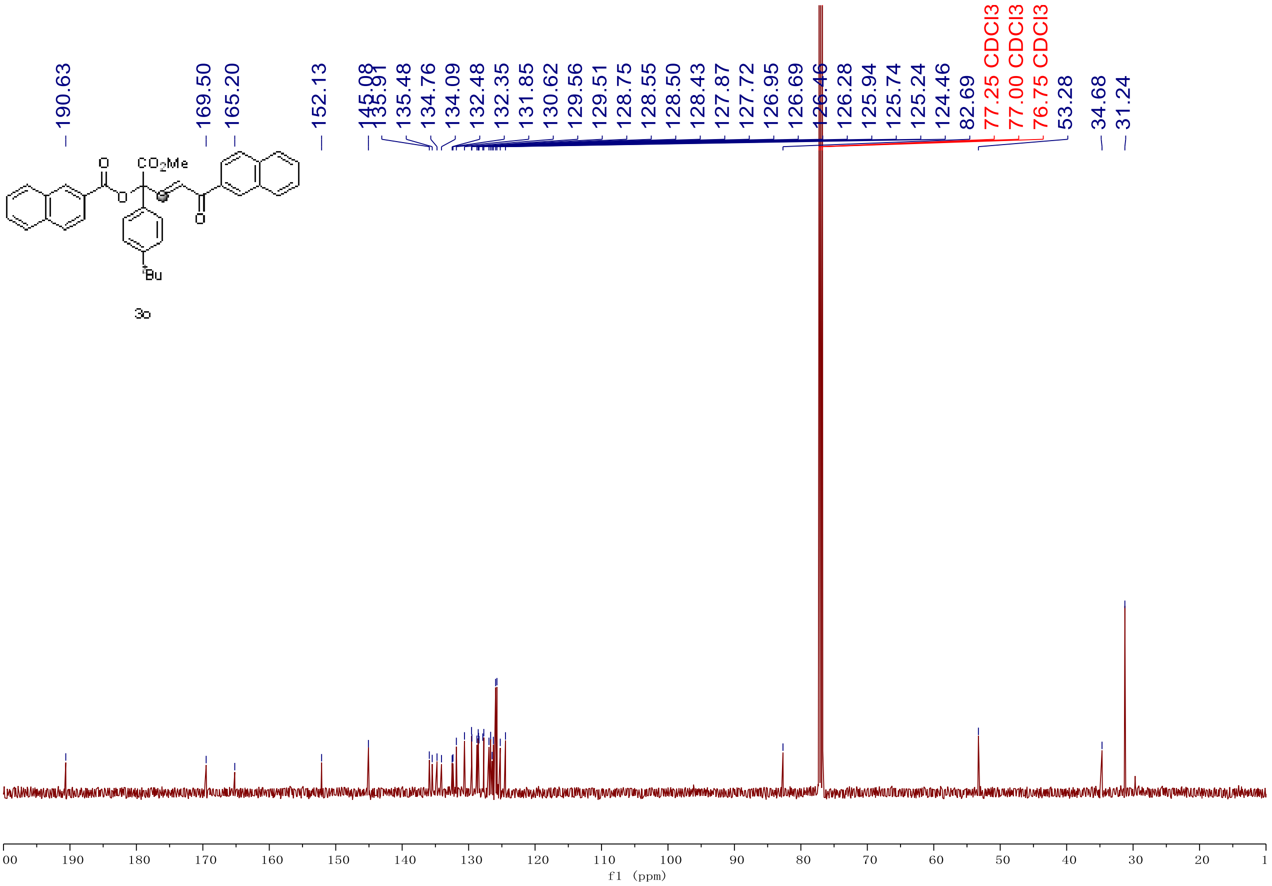

Supplementary Figure 42. ^{13}C NMR spectrum of **3o**.

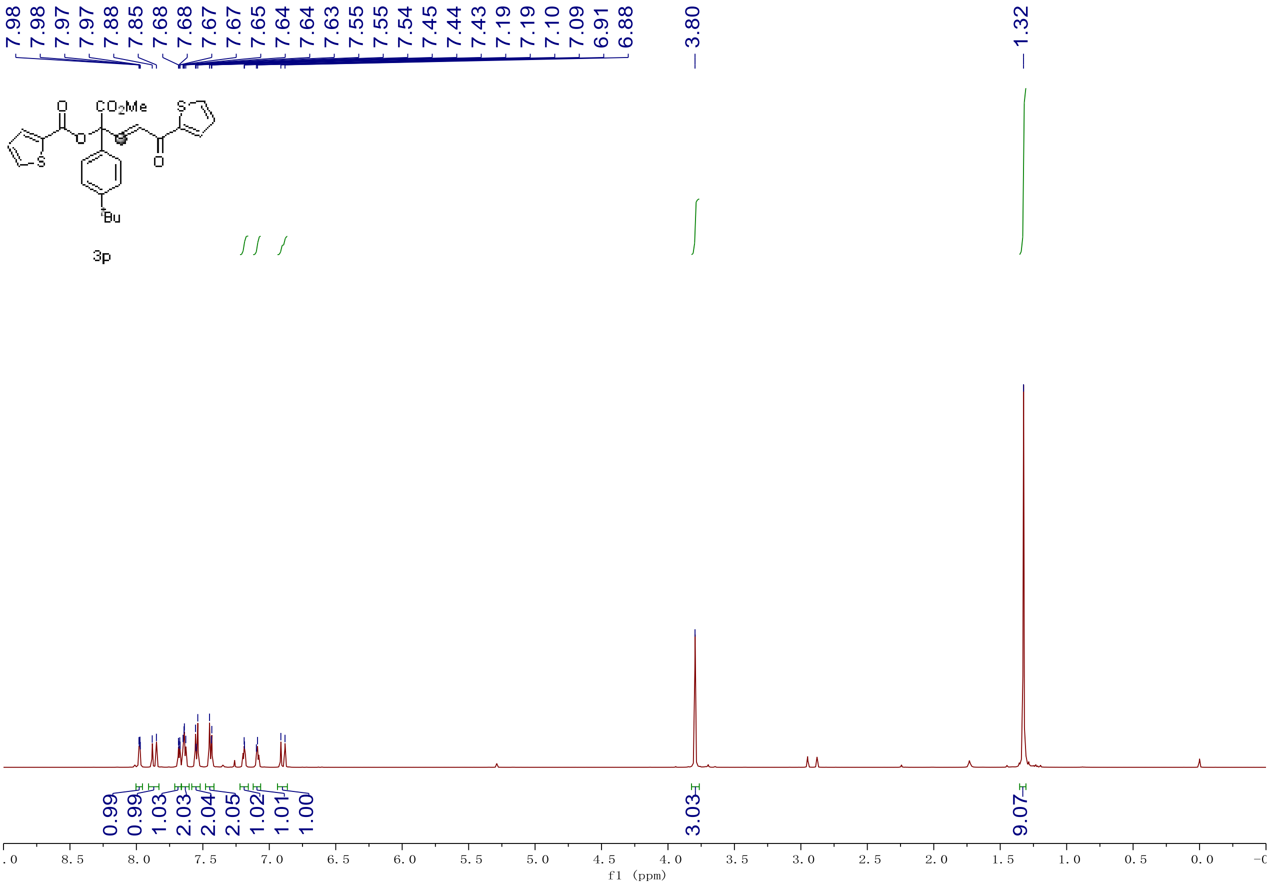

Supplementary Figure 43. ^{1}H NMR spectrum of **3p**.

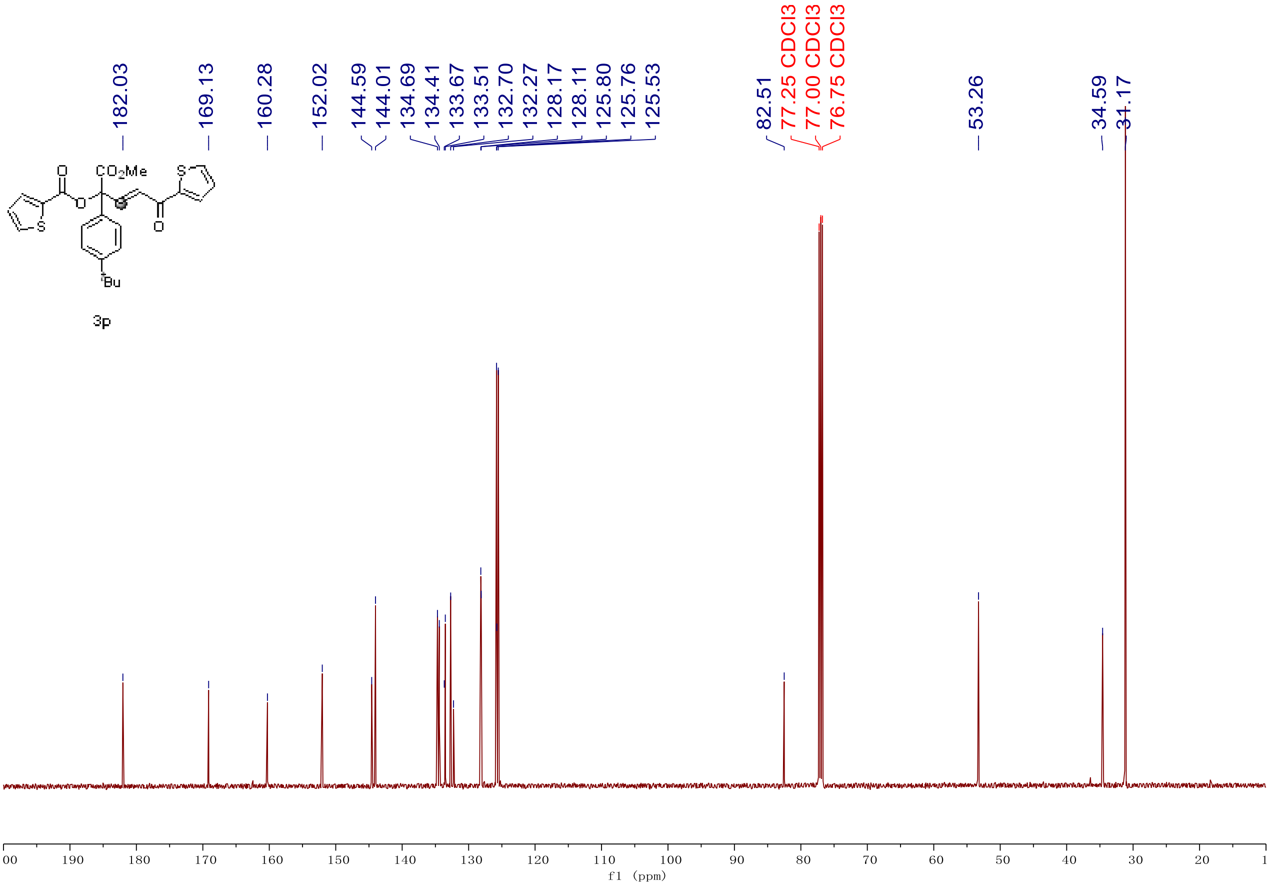

Supplementary Figure 44. ^{13}C NMR spectrum of **3p**.

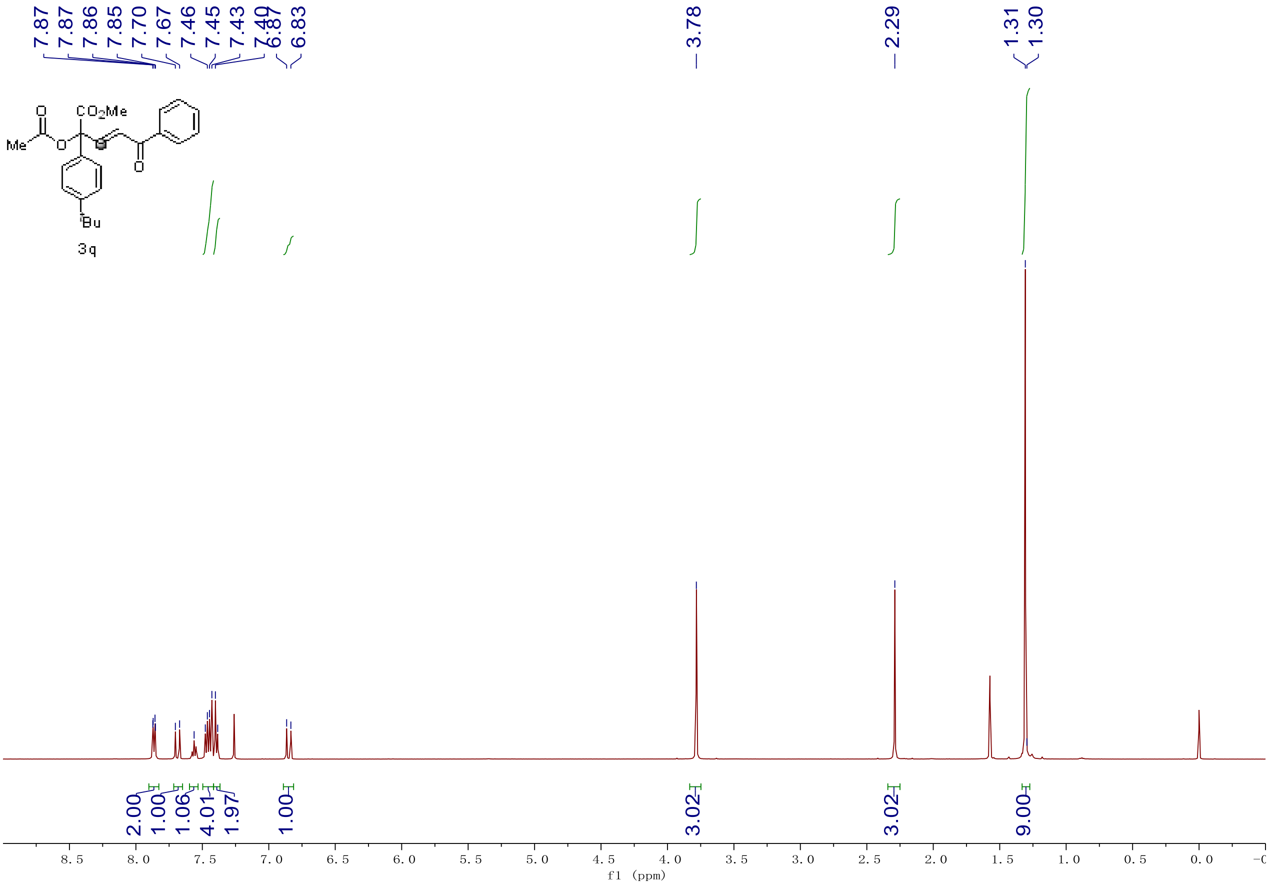

Supplementary Figure 45. ^{1}H NMR spectrum of **3q**.

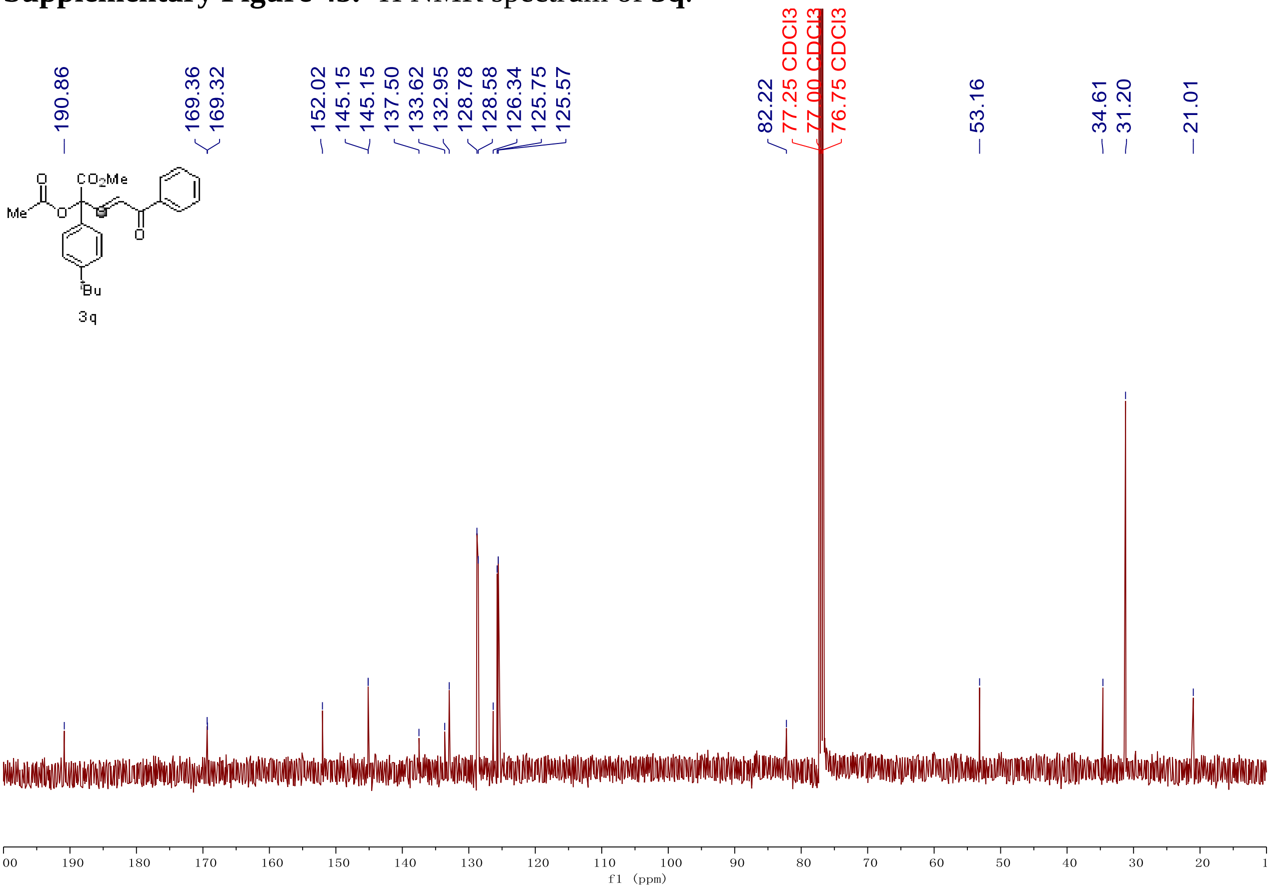

Supplementary Figure 46. ^{13}C NMR spectrum of **3q**.

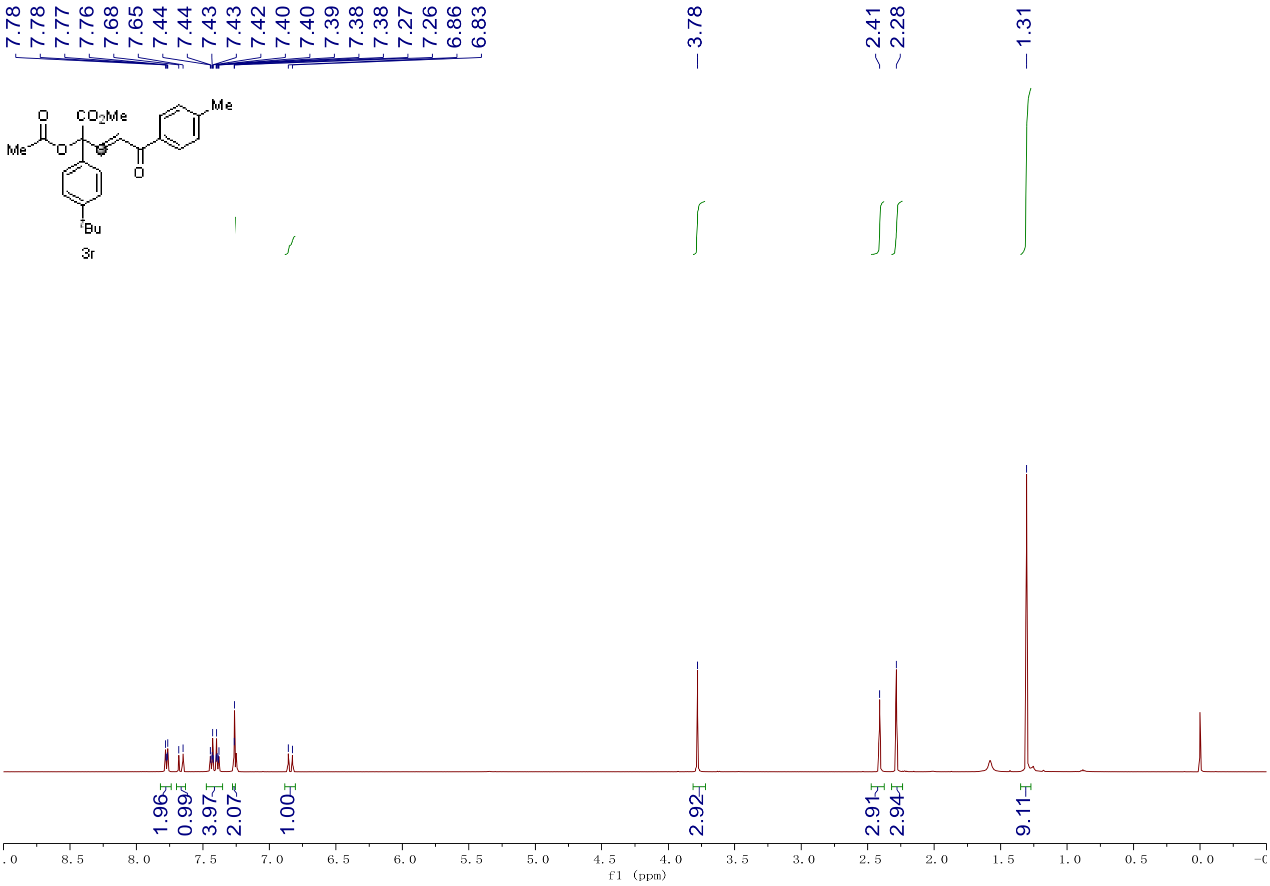

Supplementary Figure 47. ^{1}H NMR spectrum of **3r**.

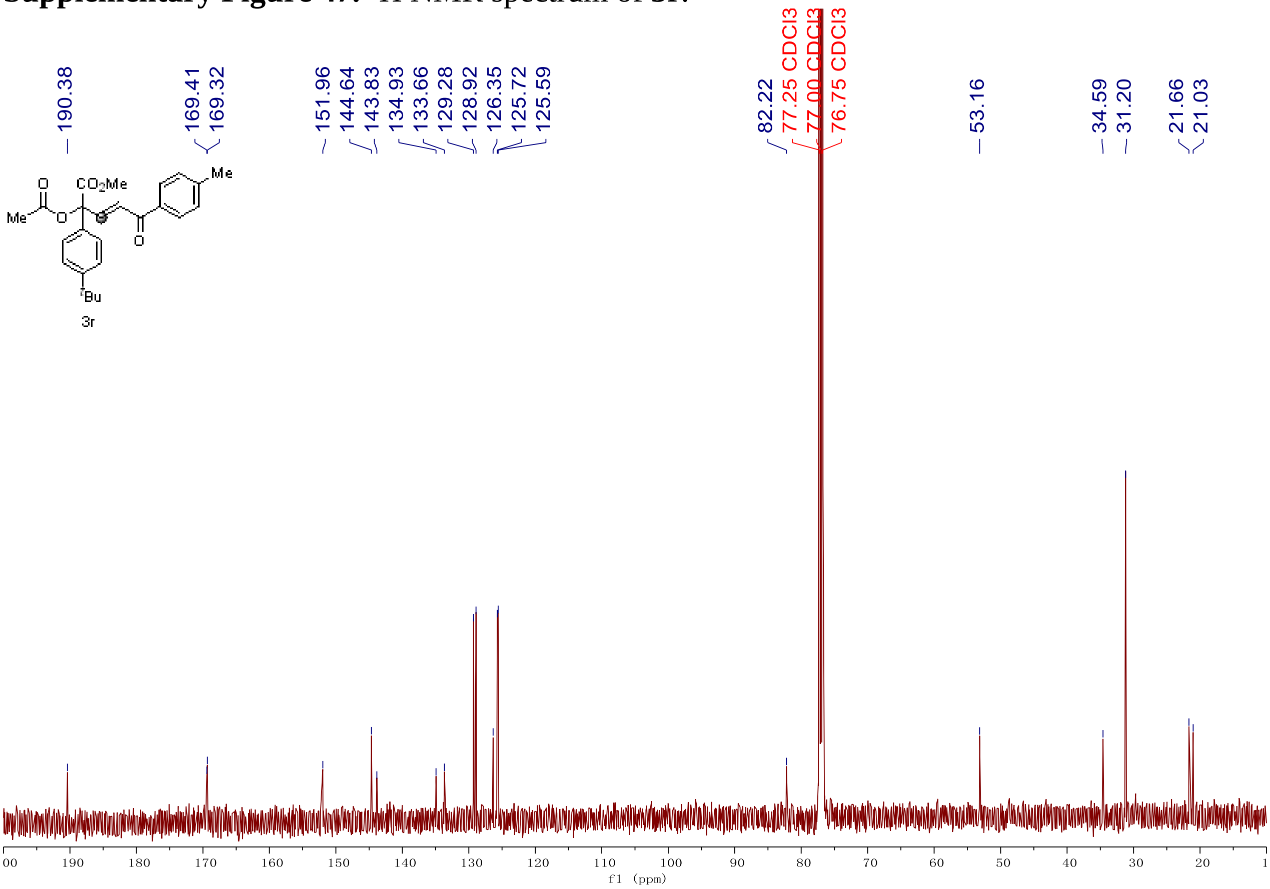

Supplementary Figure 48. ^{13}C NMR spectrum of **3r**.

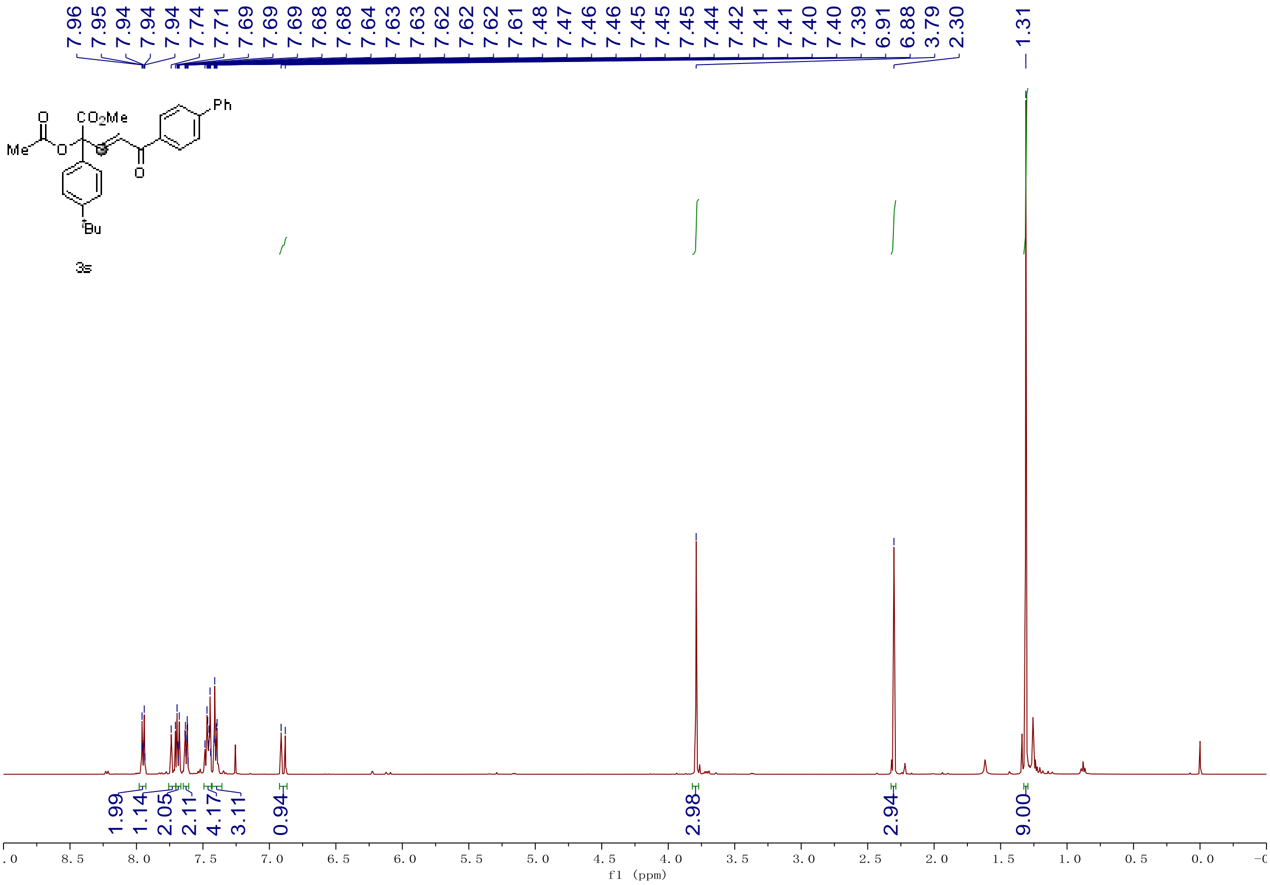

Supplementary Figure 49. ^{1}H NMR spectrum of **3s**.

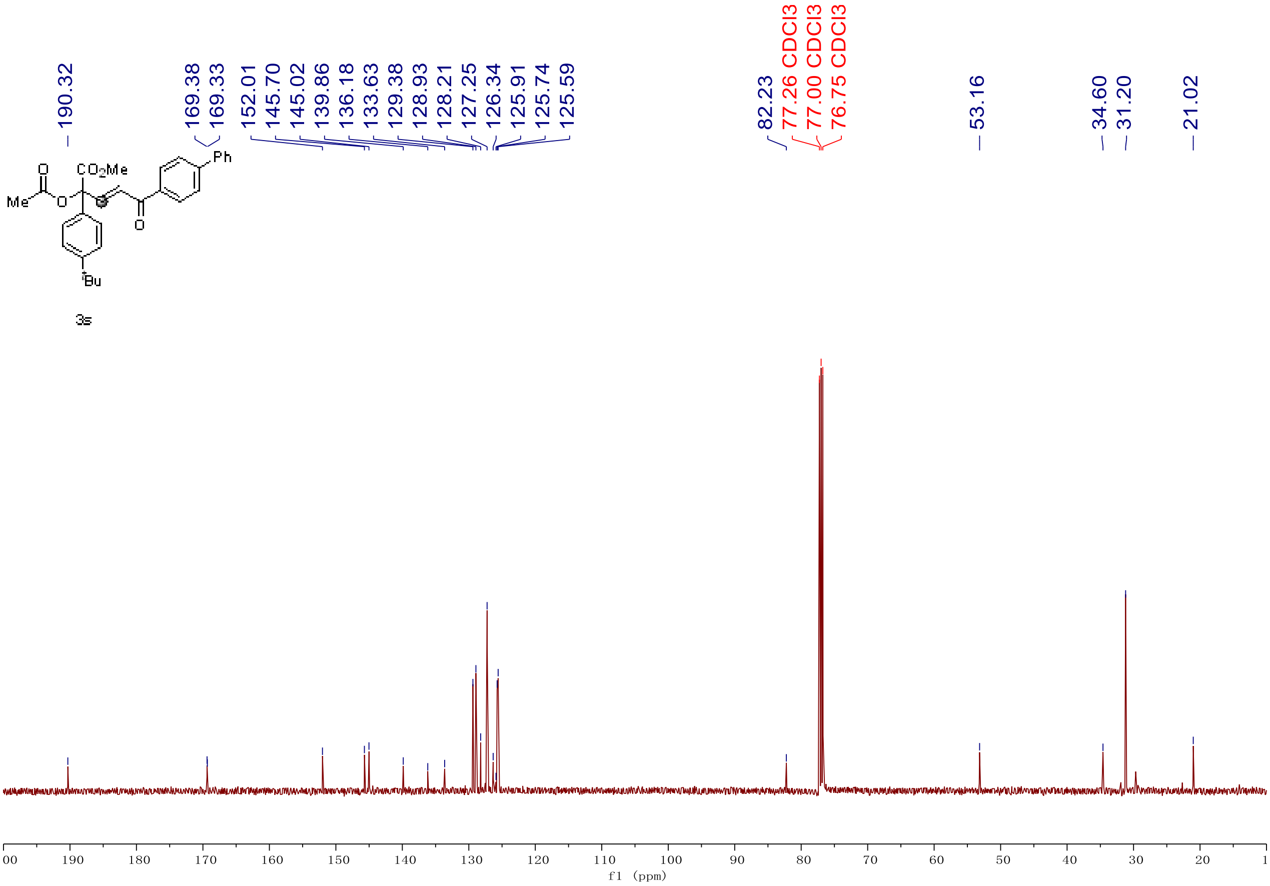

Supplementary Figure 50. ^{13}C NMR spectrum of **3s**.

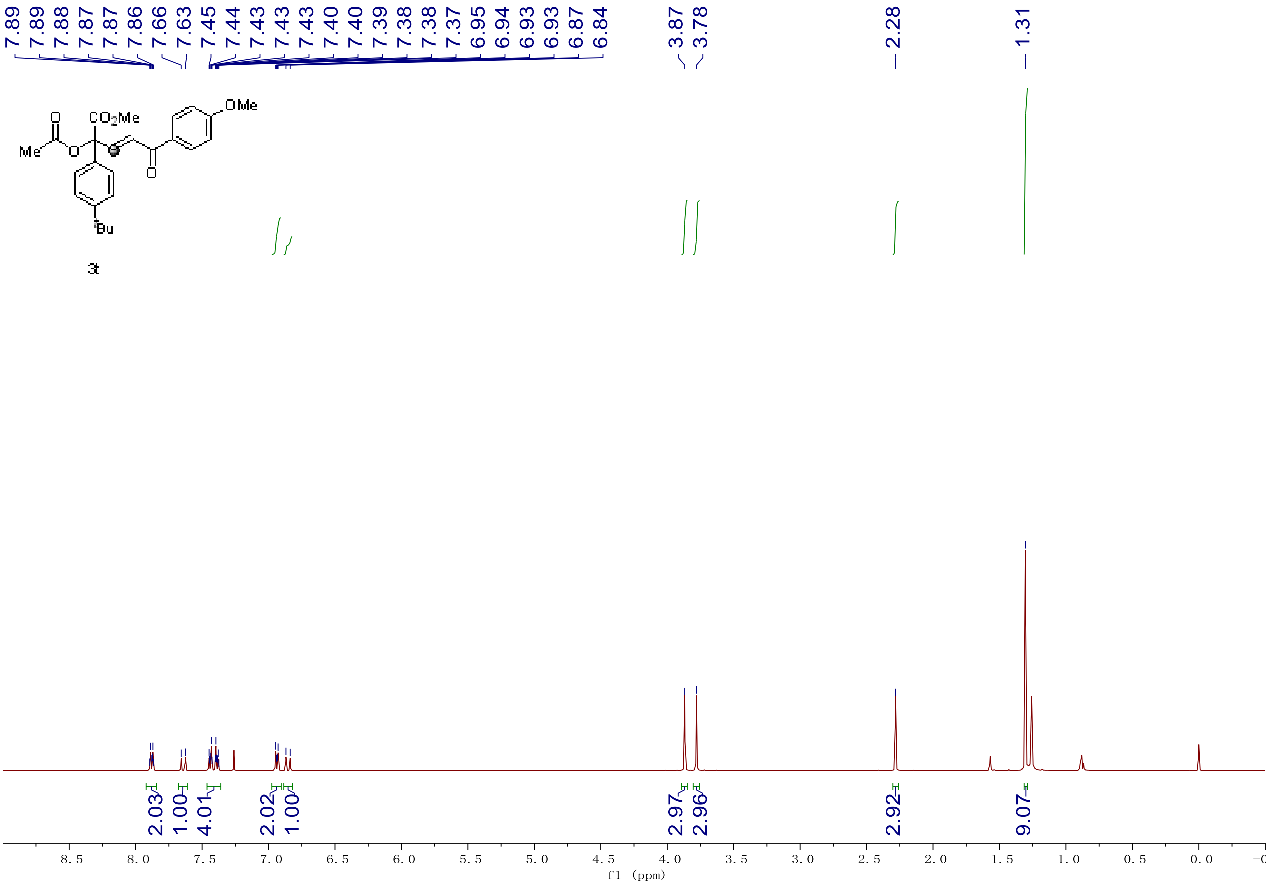

Supplementary Figure 51. ^{1}H NMR spectrum of **3t**.

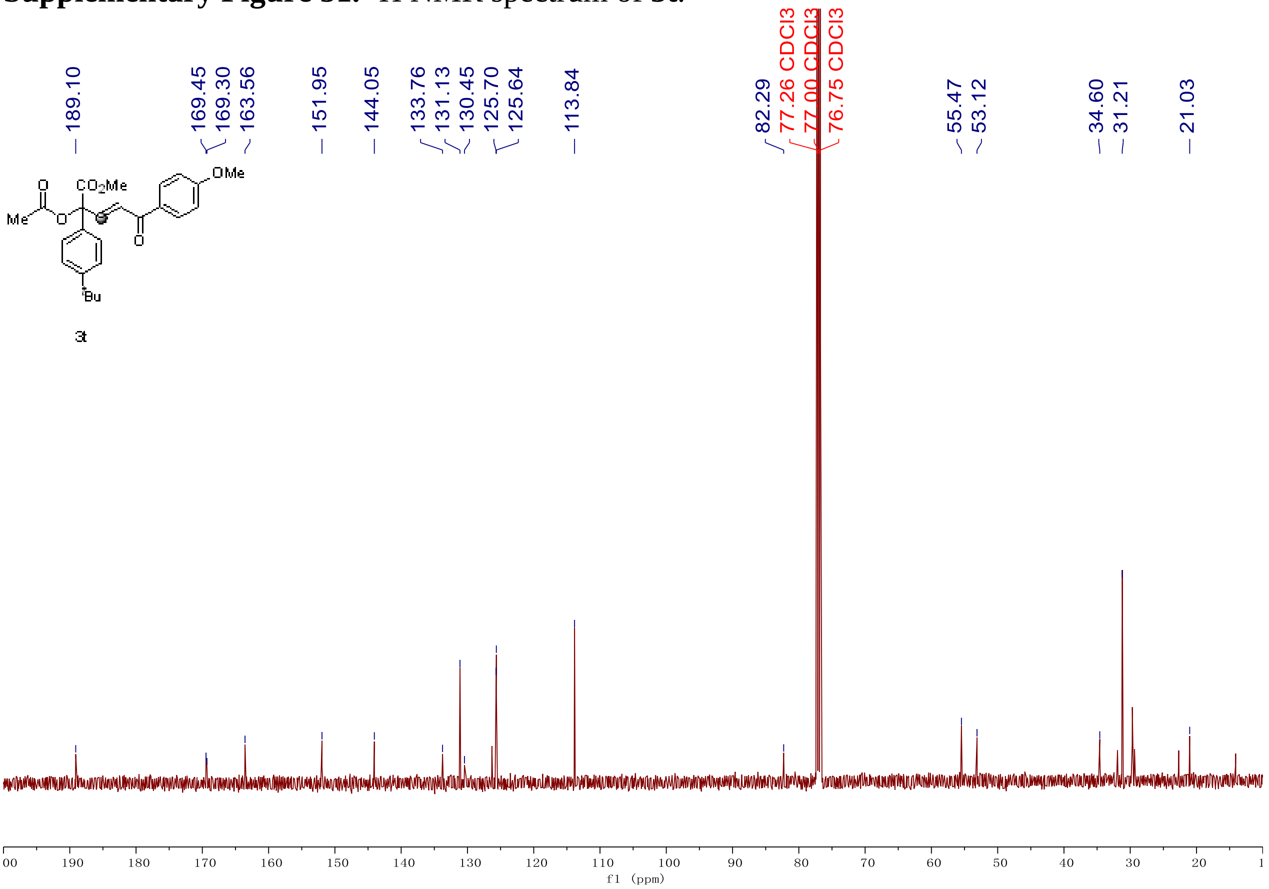

Supplementary Figure 52. ^{13}C NMR spectrum of **3t**.

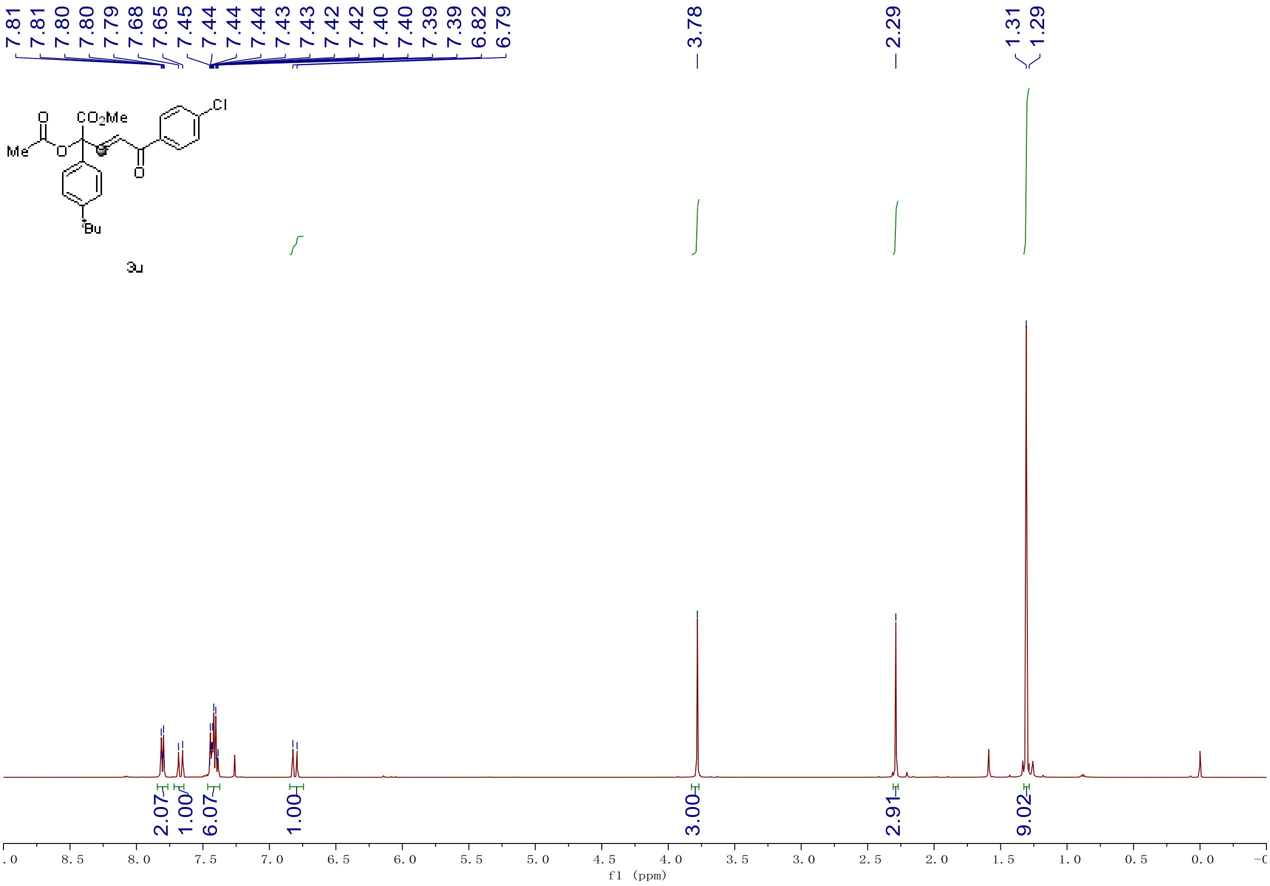

Supplementary Figure 53. ^{1}H NMR spectrum of **3u**.

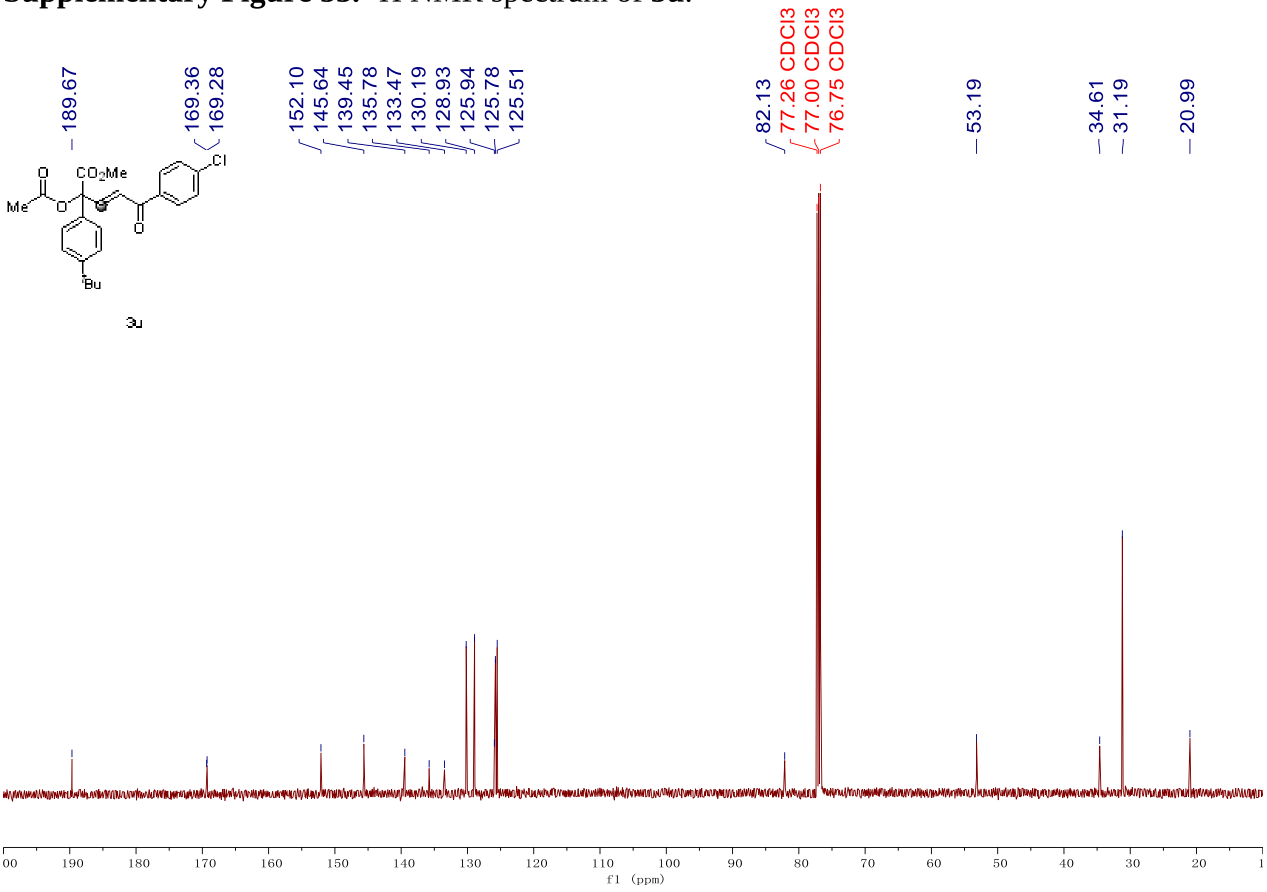

Supplementary Figure 54. ^{13}C NMR spectrum of **3u**.

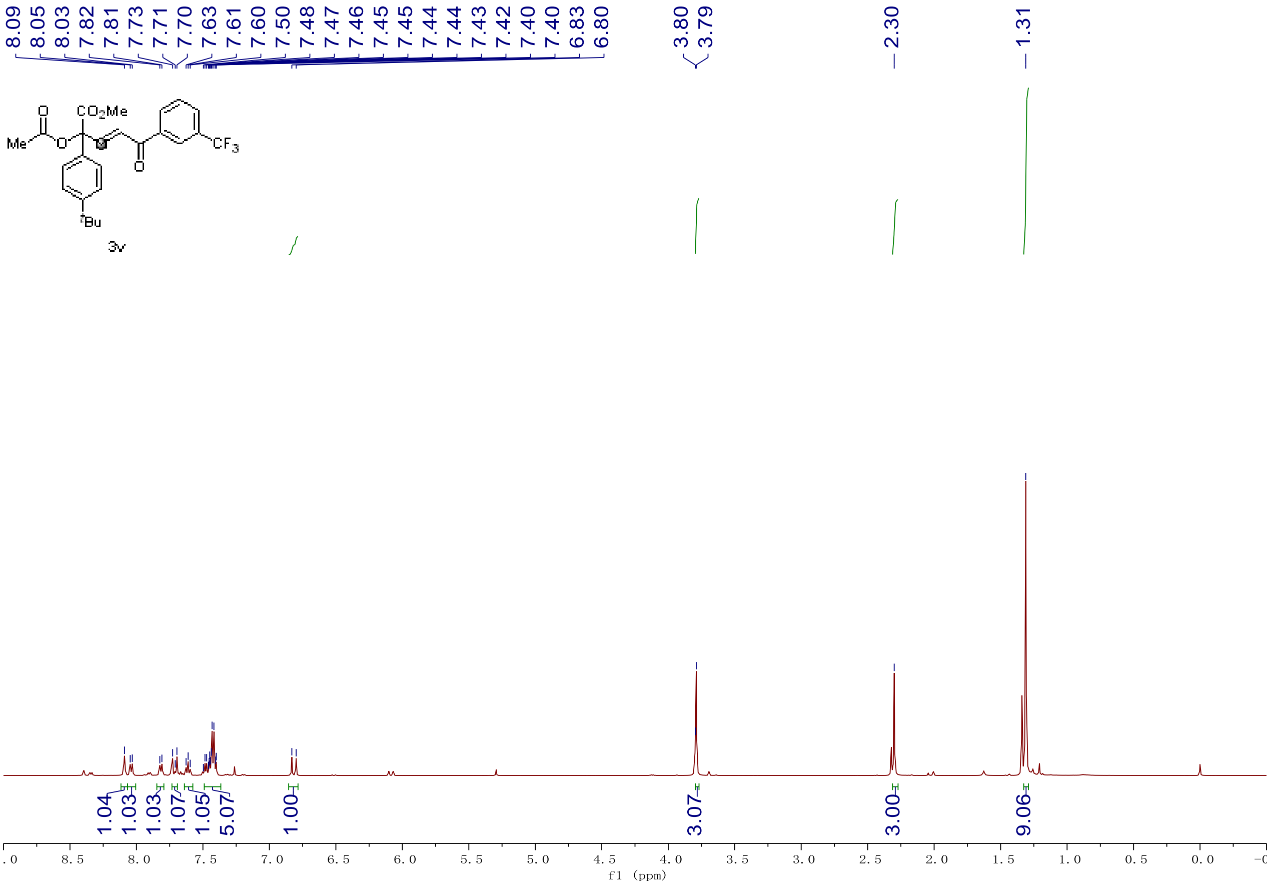

Supplementary Figure 55. ^{1}H NMR spectrum of **3v**.

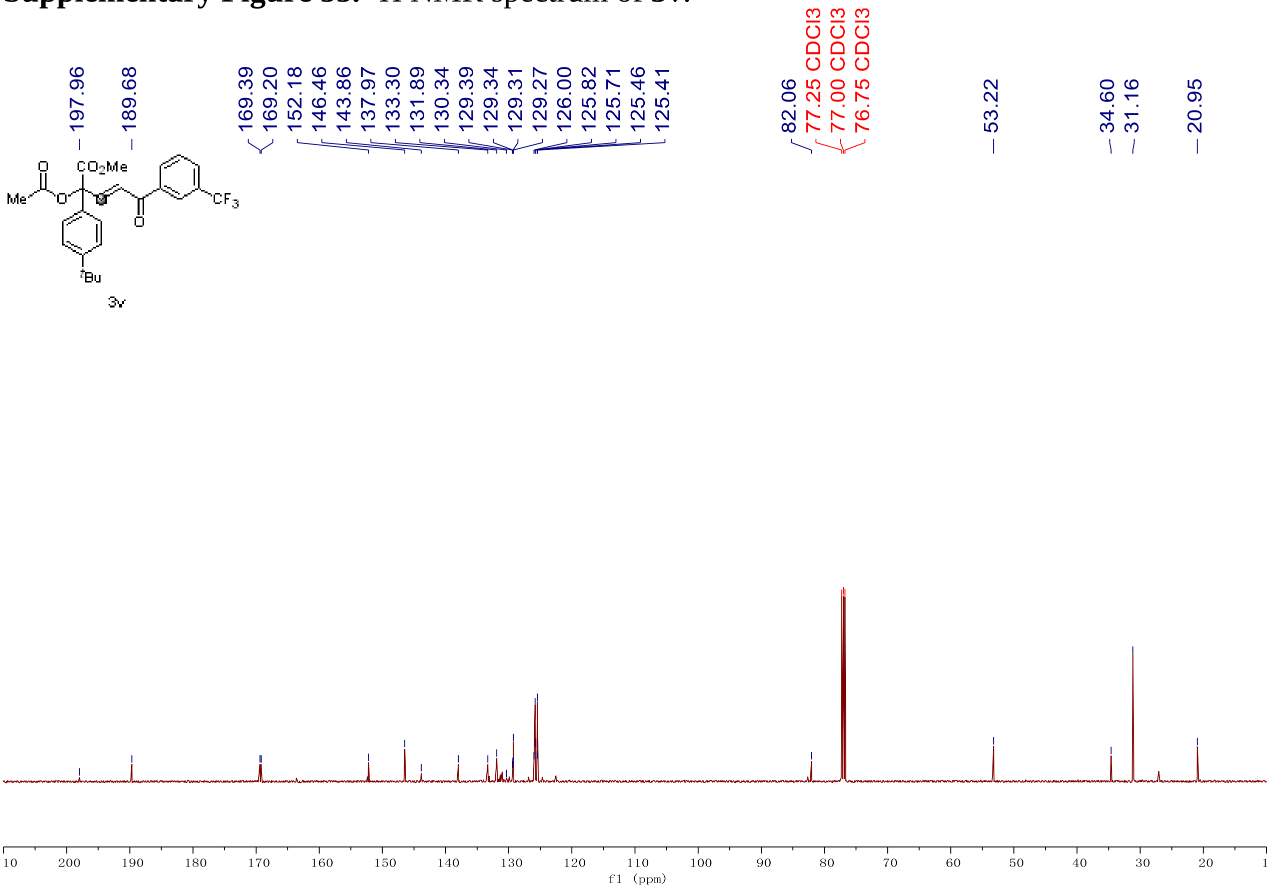

Supplementary Figure 56. ^{13}C NMR spectrum of **3v**.

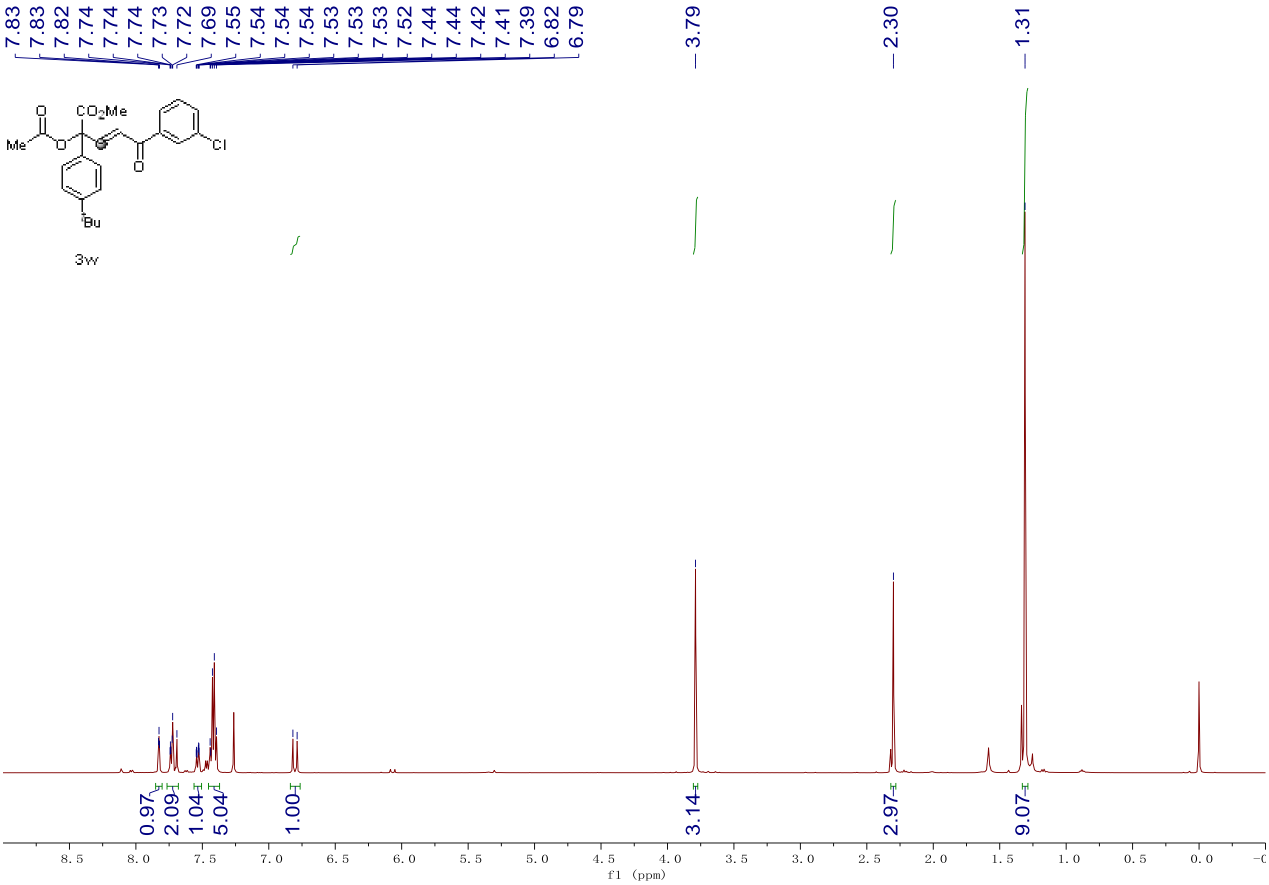

Supplementary Figure 57. ^{1}H NMR spectrum of **3w**.

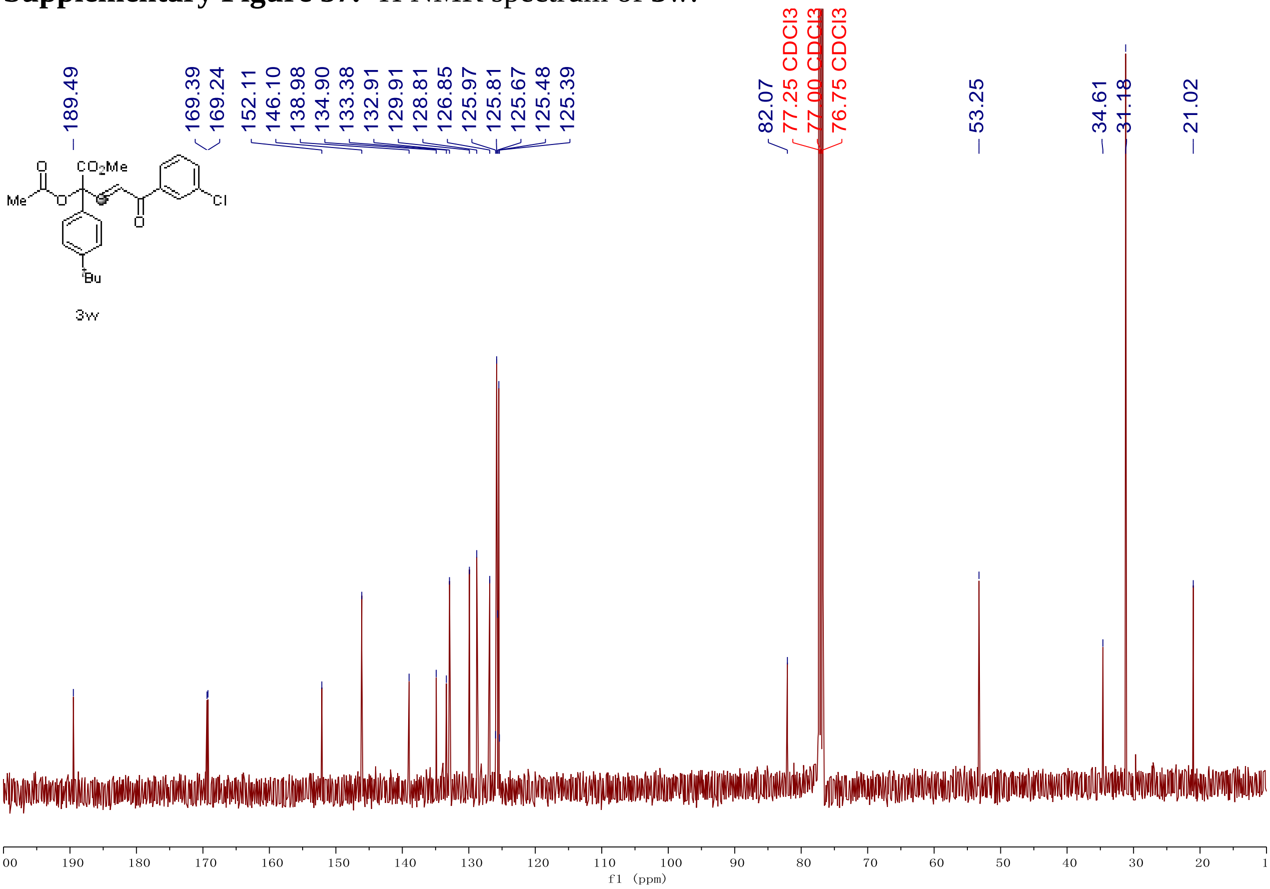

Supplementary Figure 58. ^{13}C NMR spectrum of **3w**.

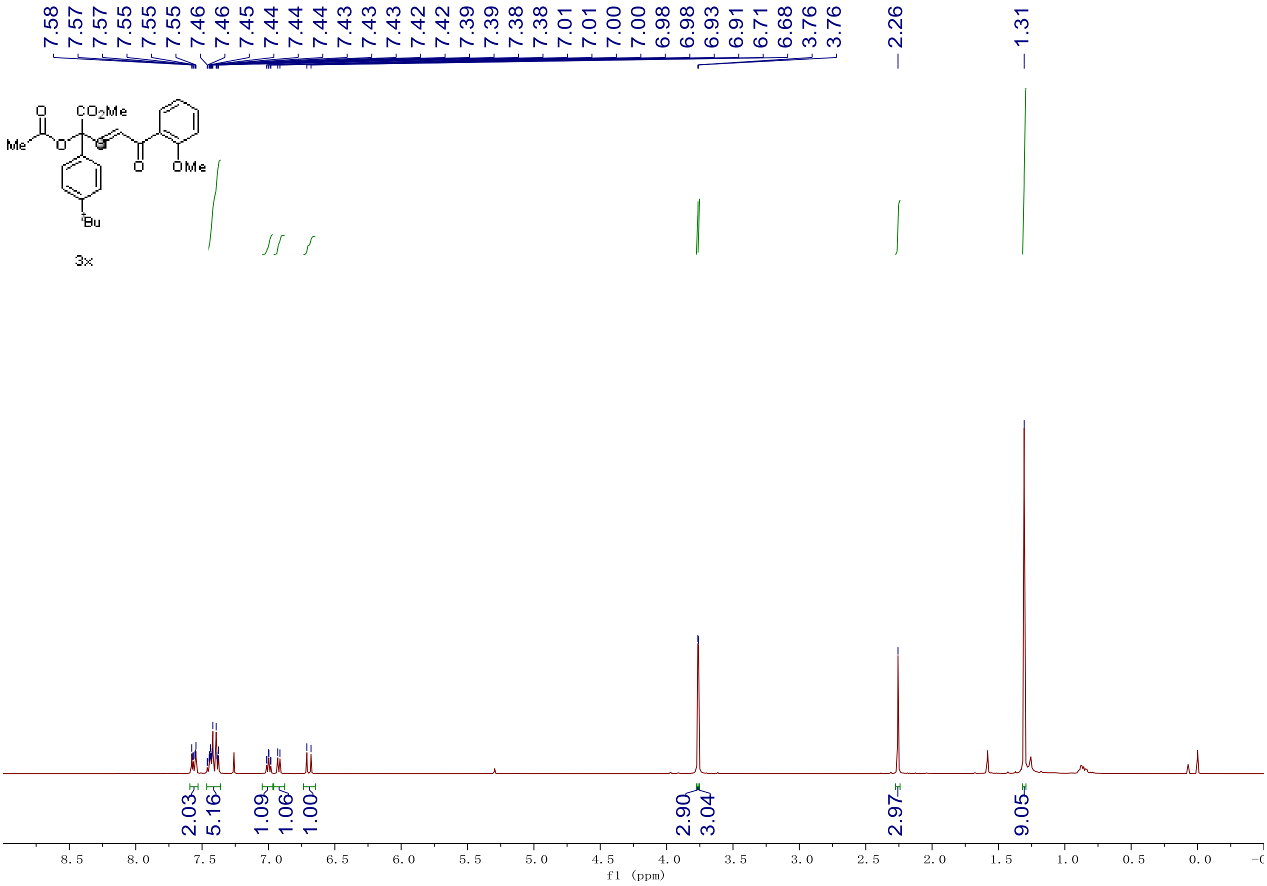

Supplementary Figure 59. ^{1}H NMR spectrum of **3x**.

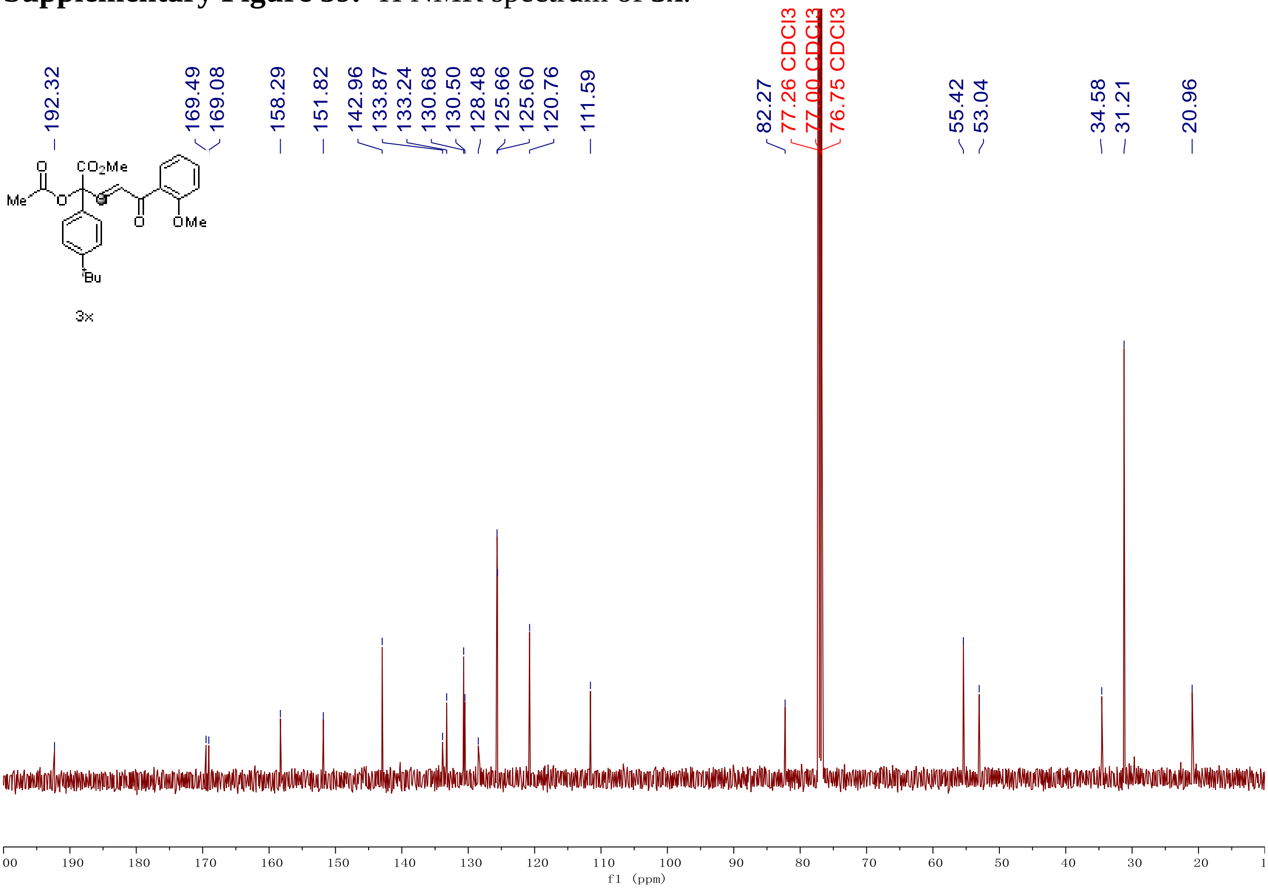

Supplementary Figure 60. ^{13}C NMR spectrum of **3x**.

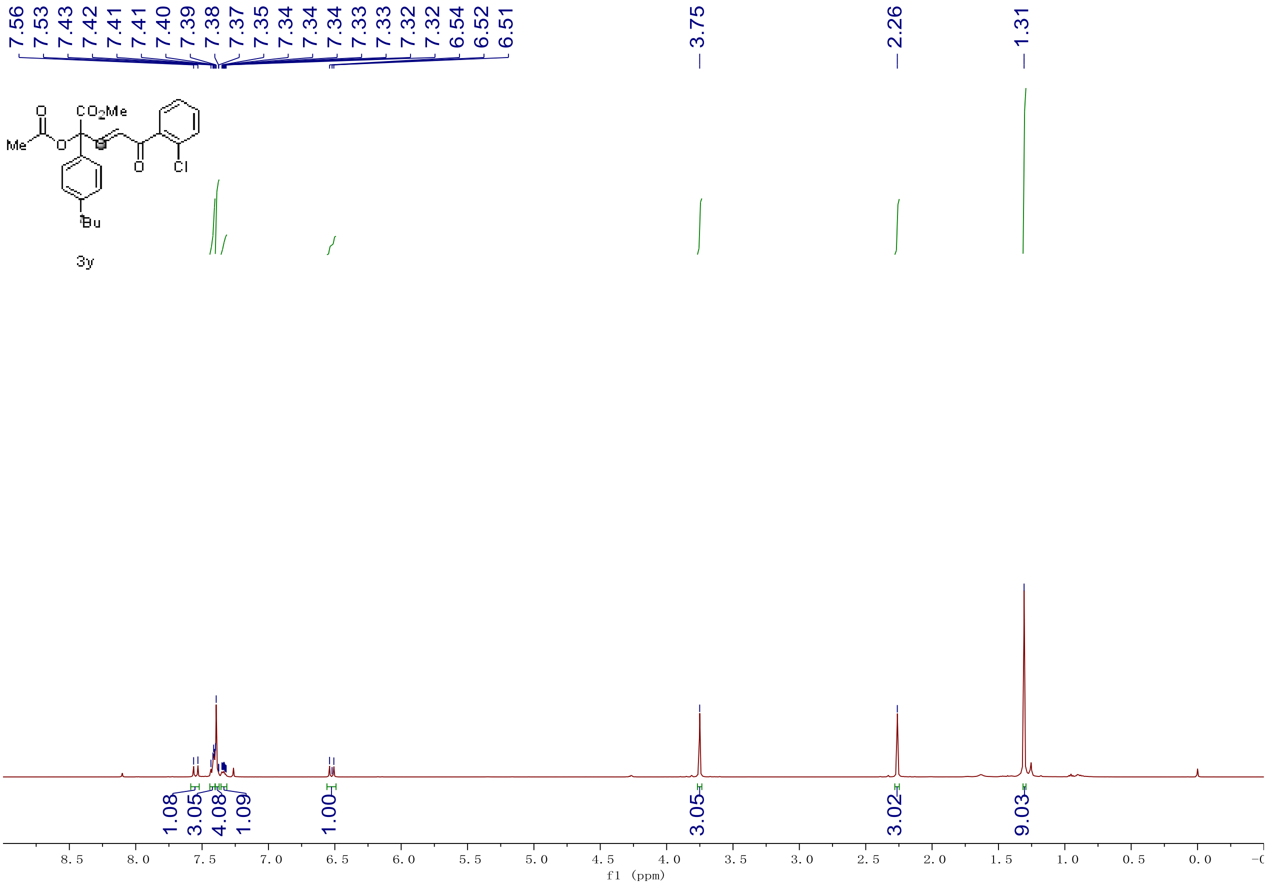

Supplementary Figure 61. ^{1}H NMR spectrum of **3y**.

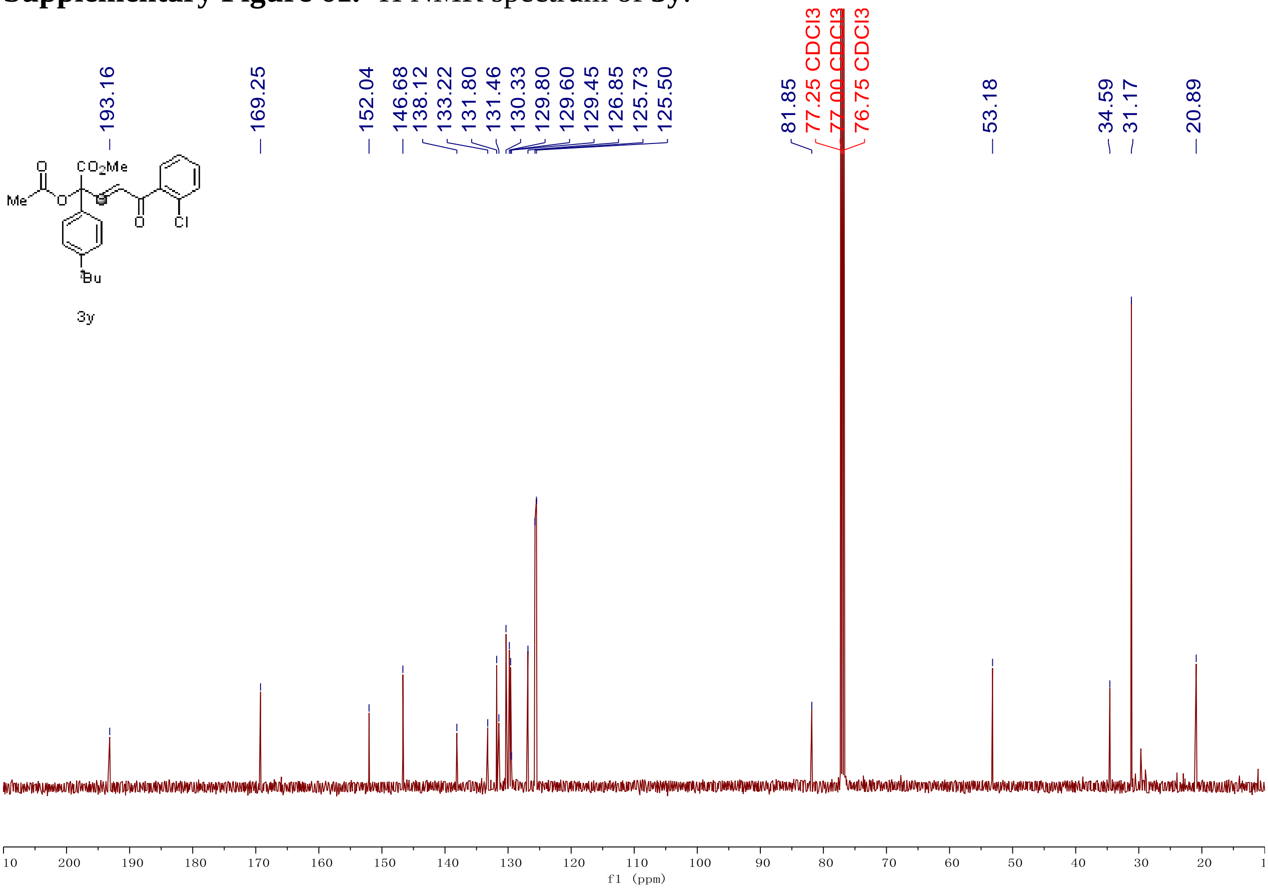

Supplementary Figure 62. ^{13}C NMR spectrum of **3y**.

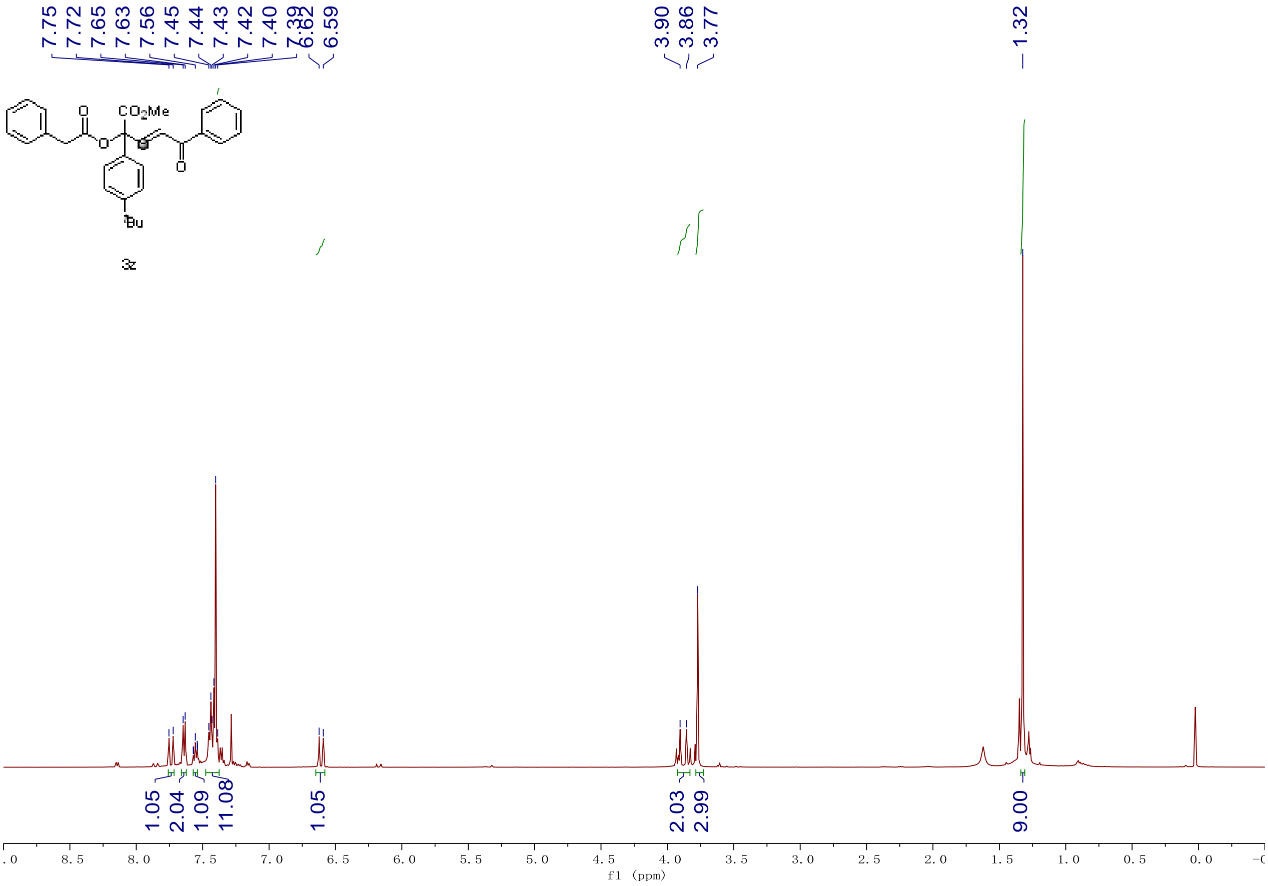

Supplementary Figure 63. ^{1}H NMR spectrum of **3z**.

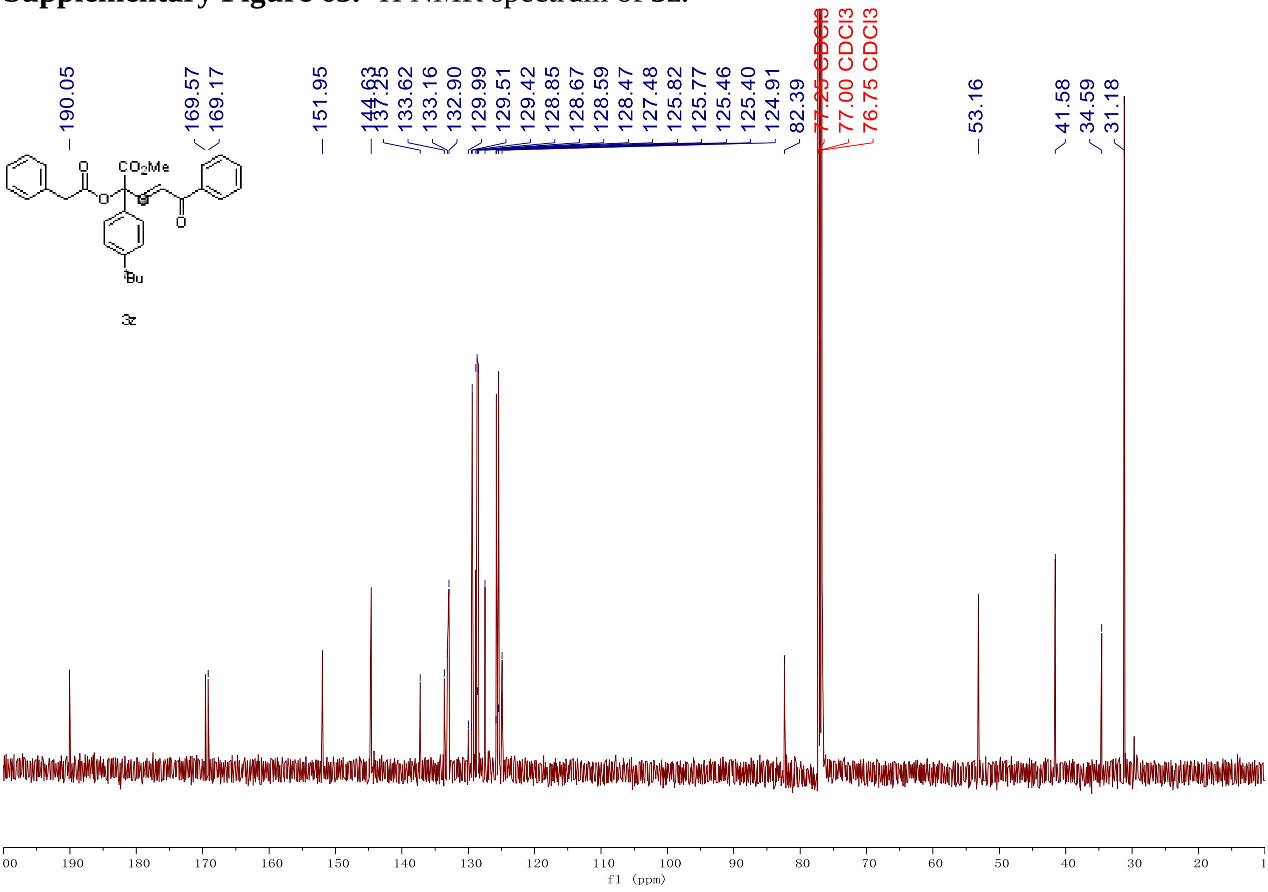

Supplementary Figure 64. ^{13}C NMR spectrum of **3z**.

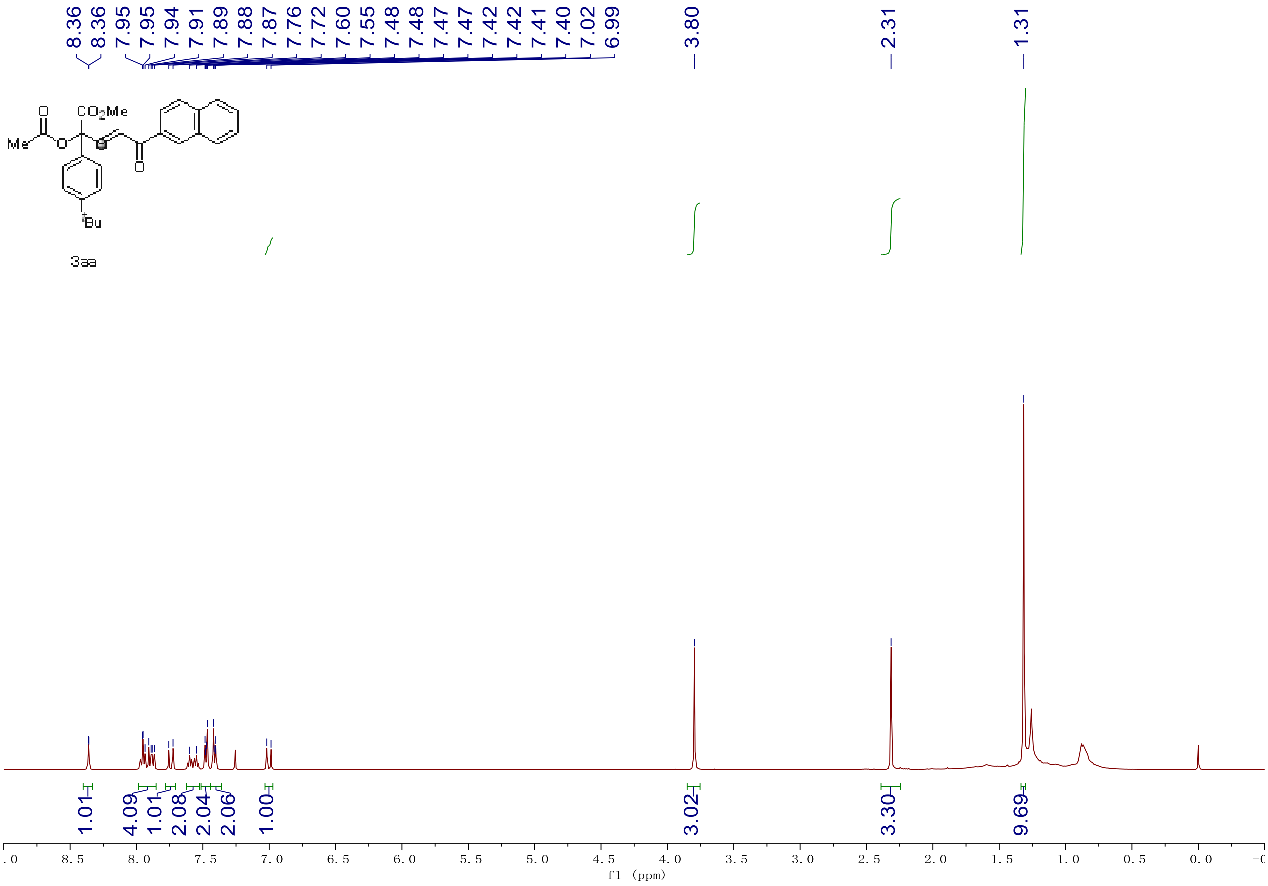

Supplementary Figure 65. ^{1}H NMR spectrum of **3aa**.

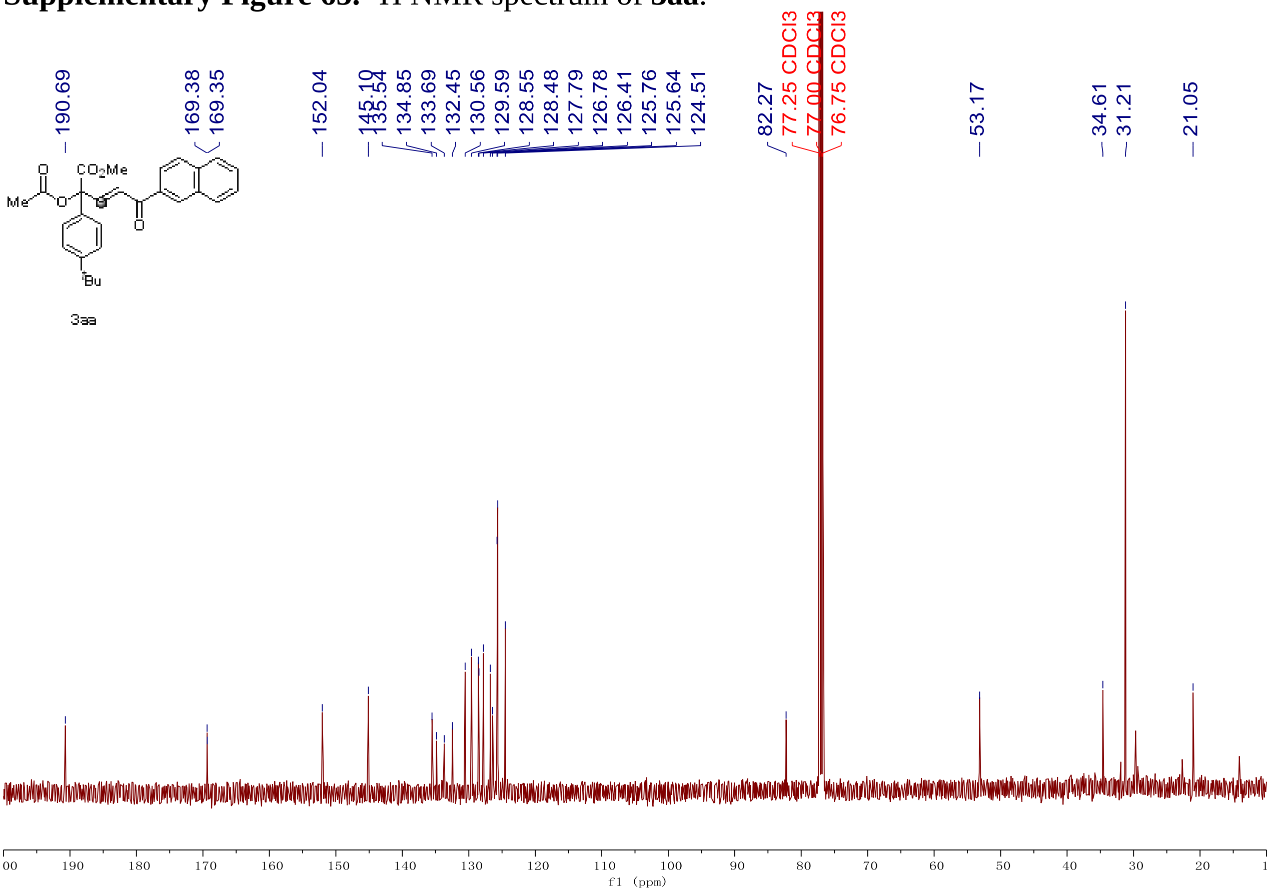

Supplementary Figure 66. ^{13}C NMR spectrum of **3aa**.

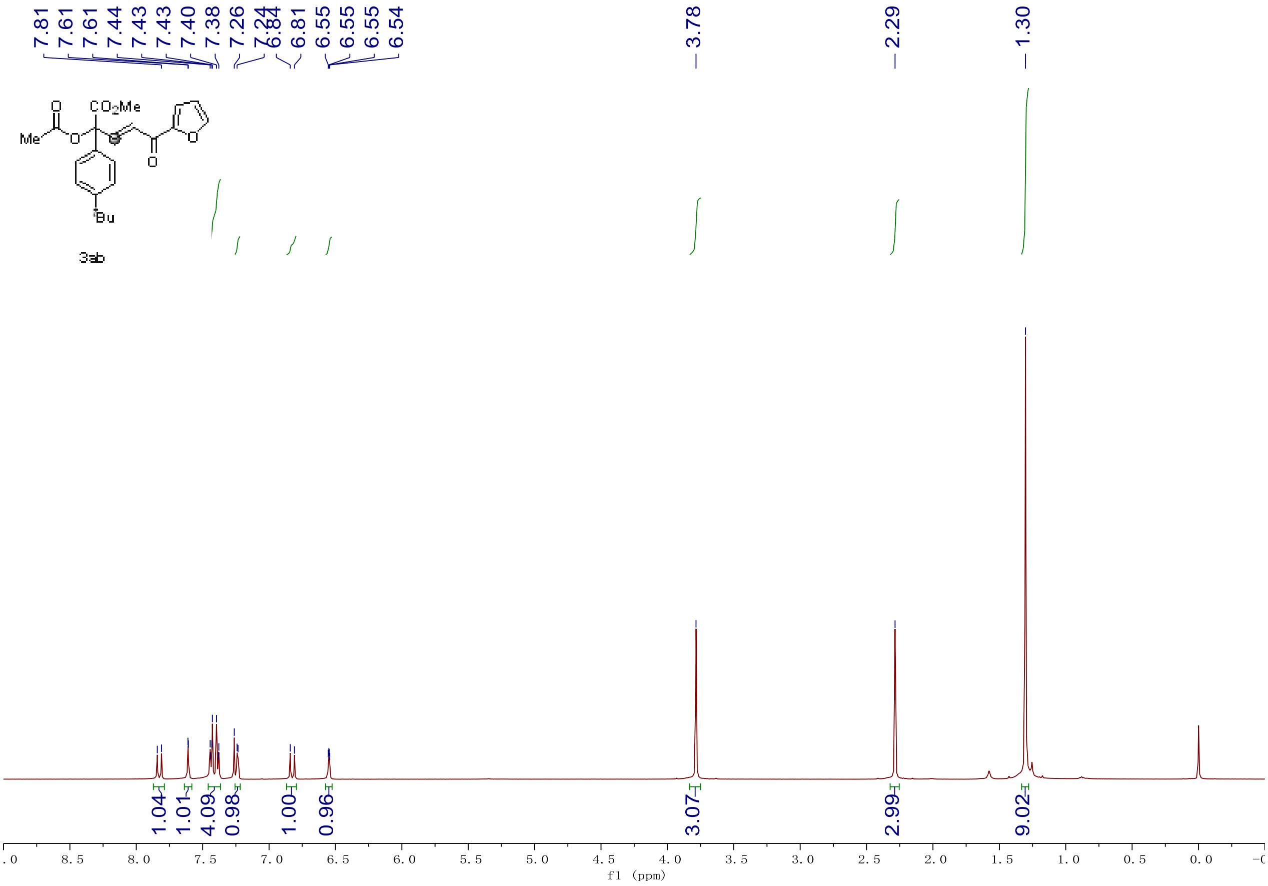

Supplementary Figure 67. ^{1}H NMR spectrum of **3ab**.

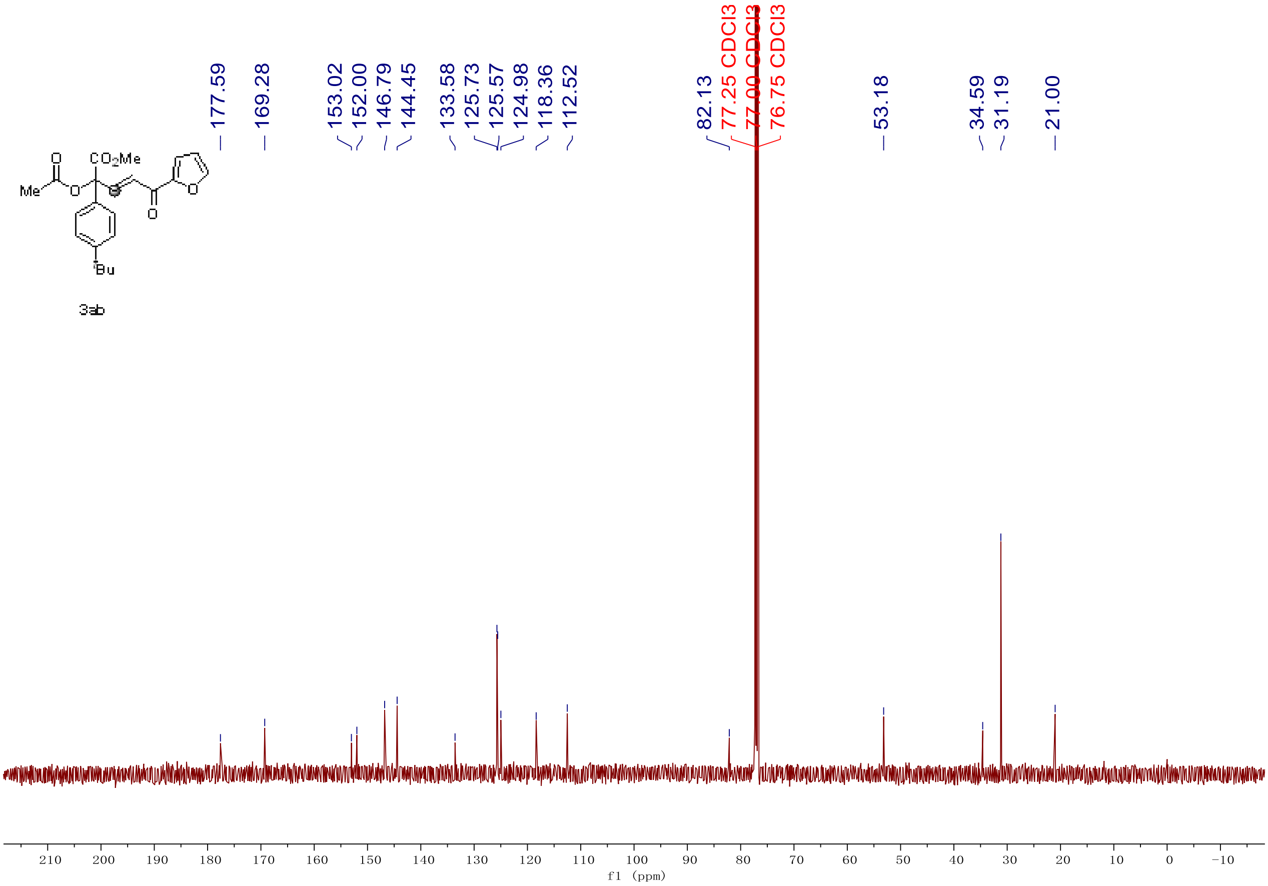

Supplementary Figure 68. ^{13}C NMR spectrum of **3ab**.

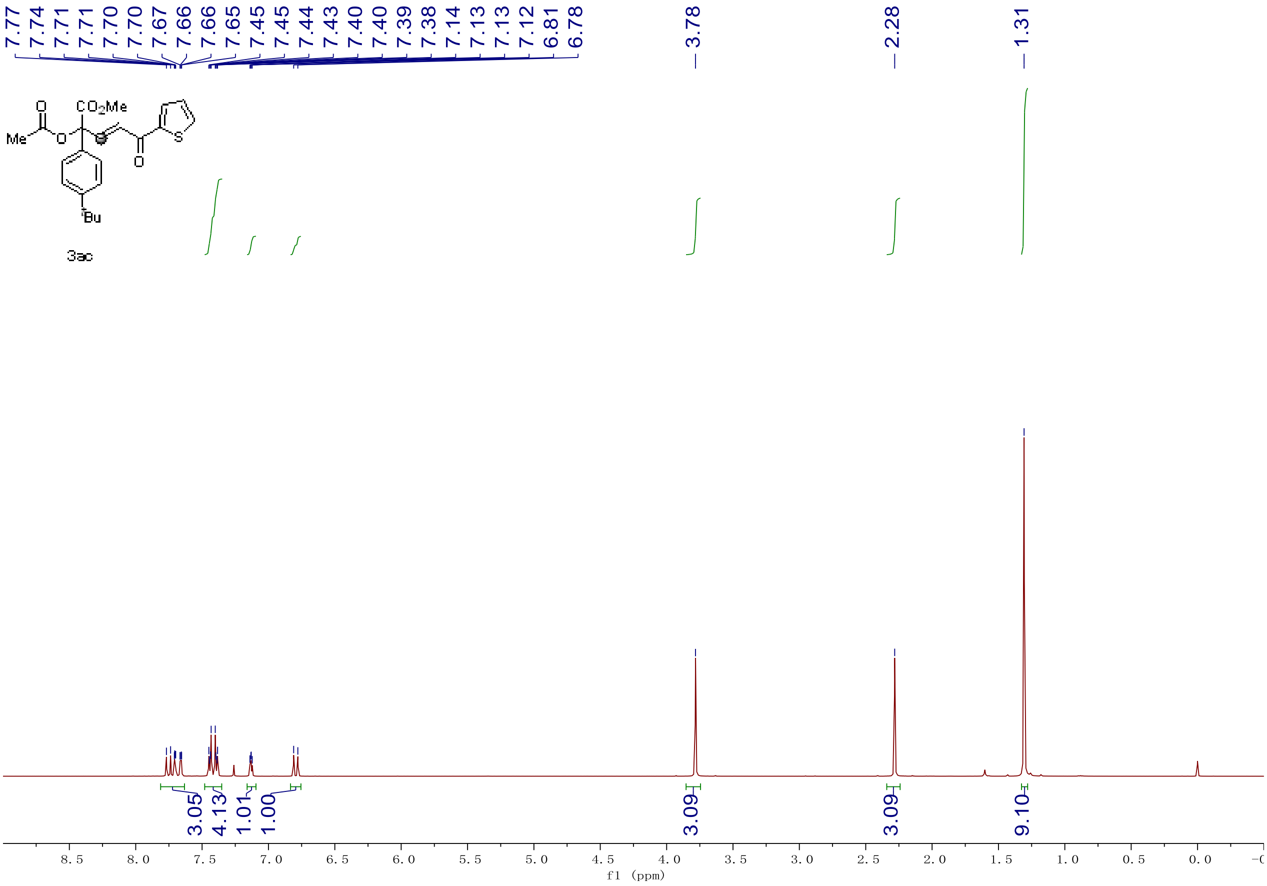

Supplementary Figure 69. ^{1}H NMR spectrum of **3ac**.

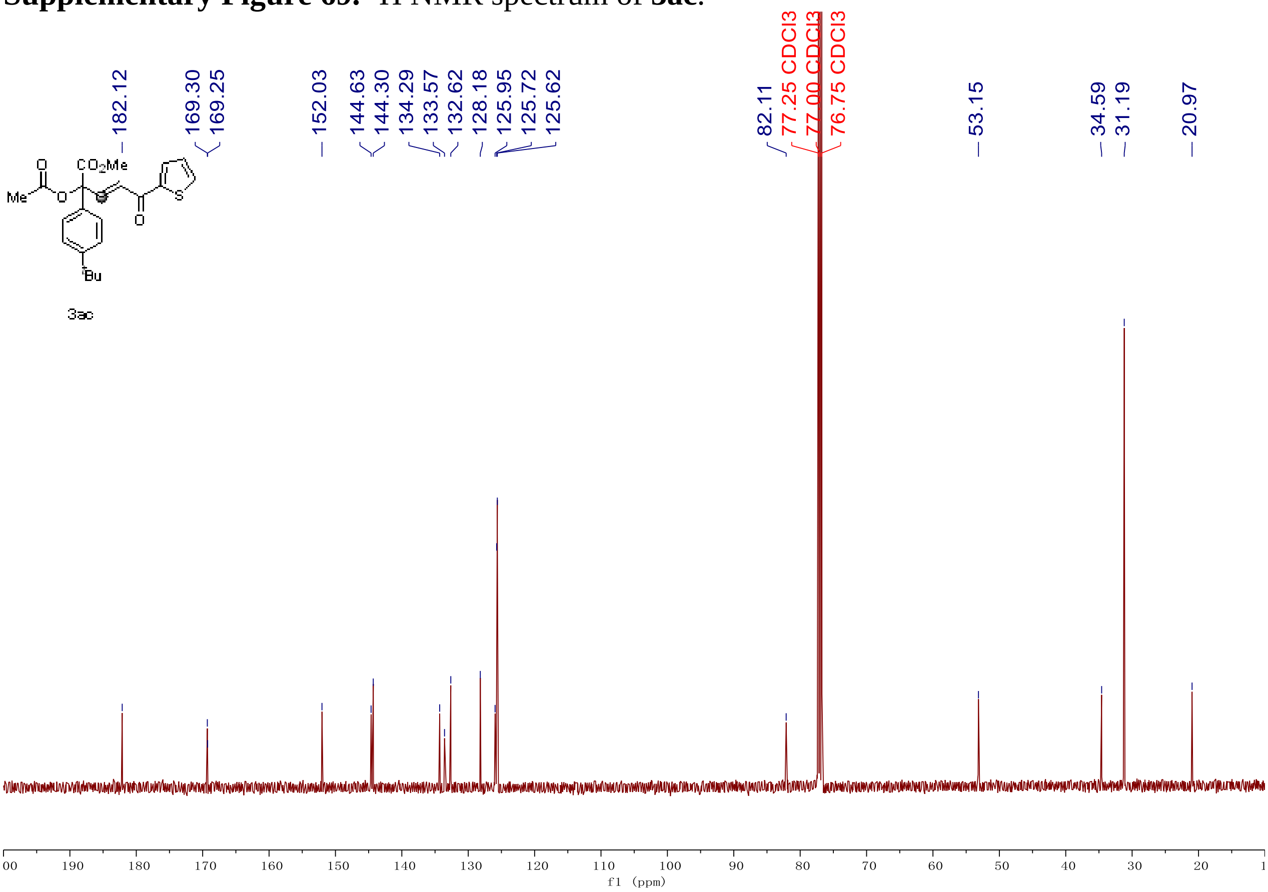

Supplementary Figure 70. ^{13}C NMR spectrum of **3ac**.

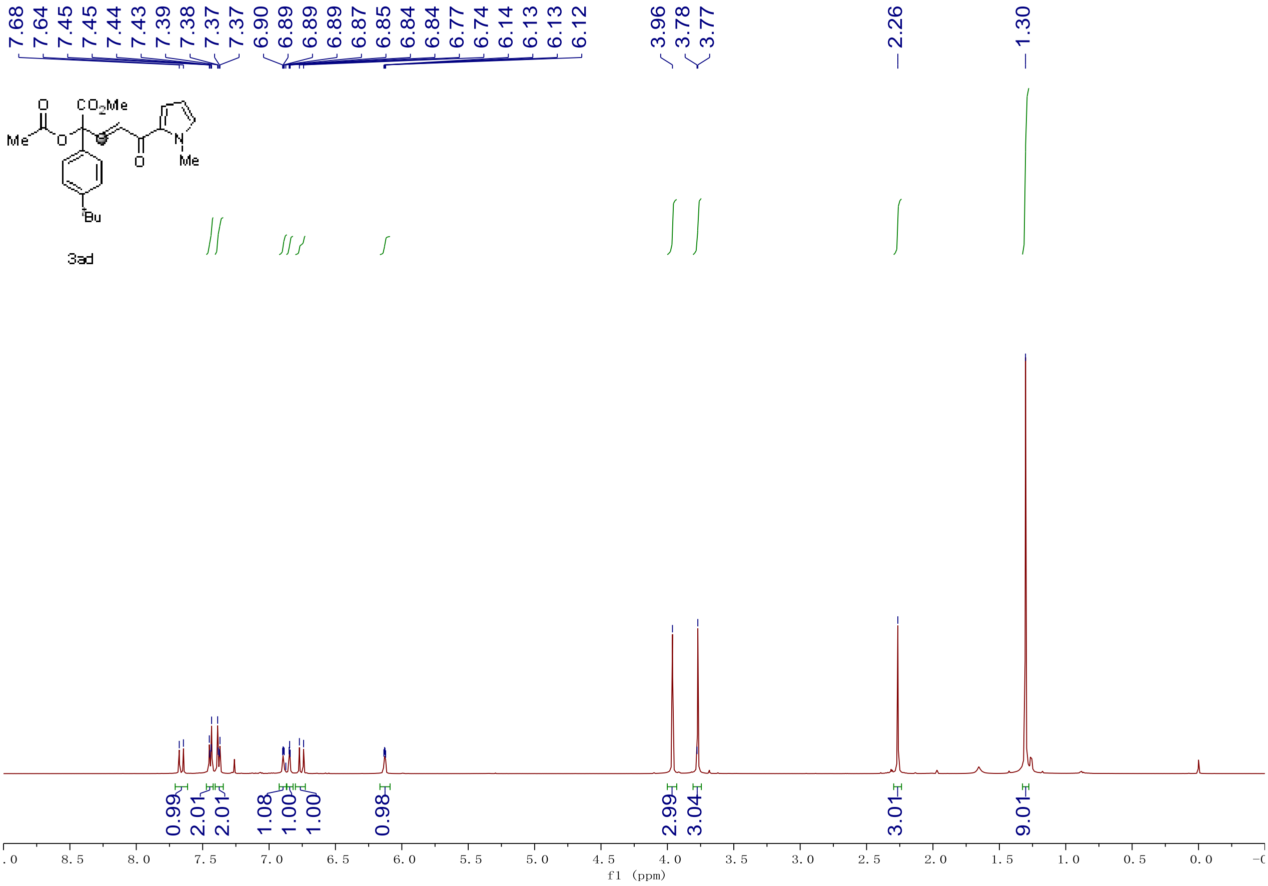

Supplementary Figure 71. ^{1}H NMR spectrum of **3ad**.

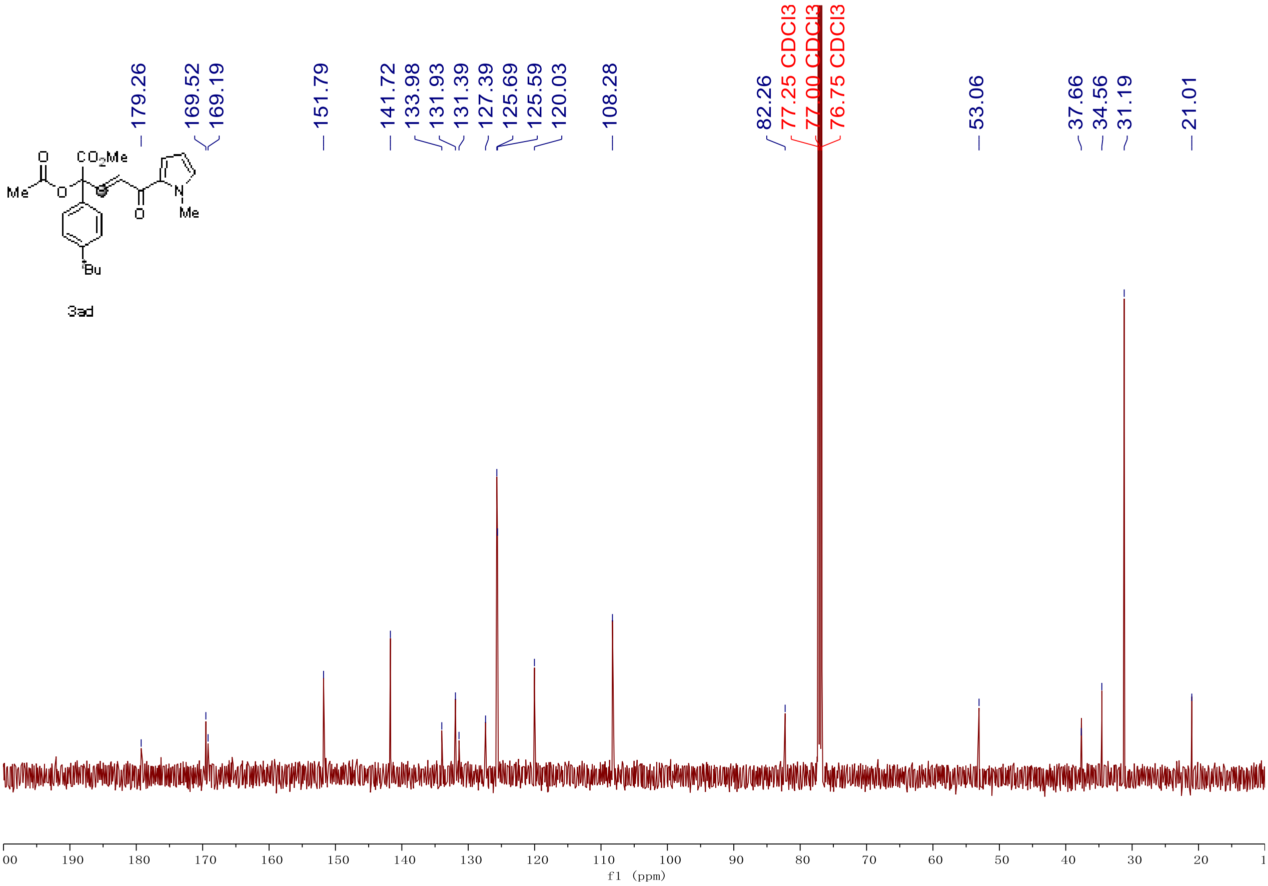

Supplementary Figure 72. ^{13}C NMR spectrum of **3ad**.

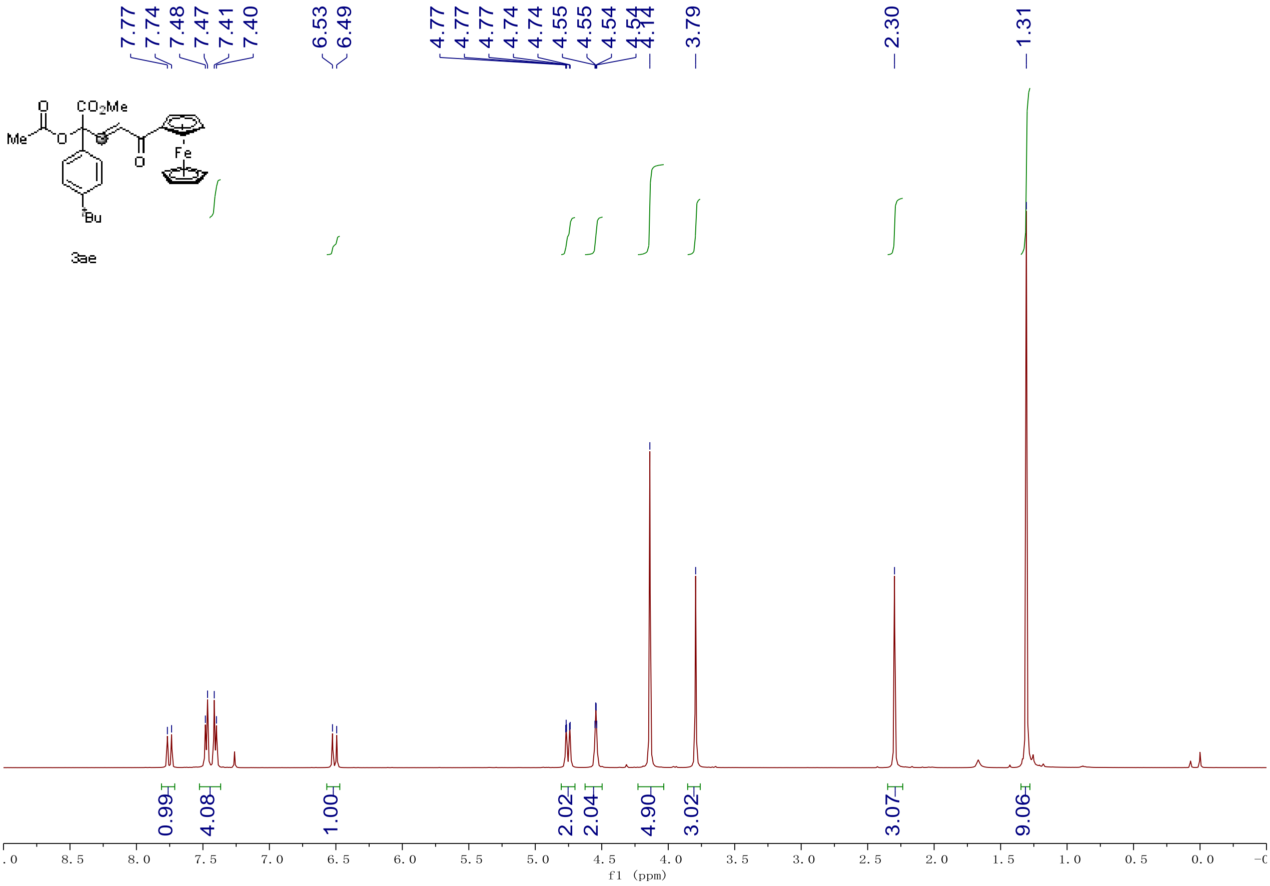

Supplementary Figure 73. ^{1}H NMR spectrum of **3ae**.

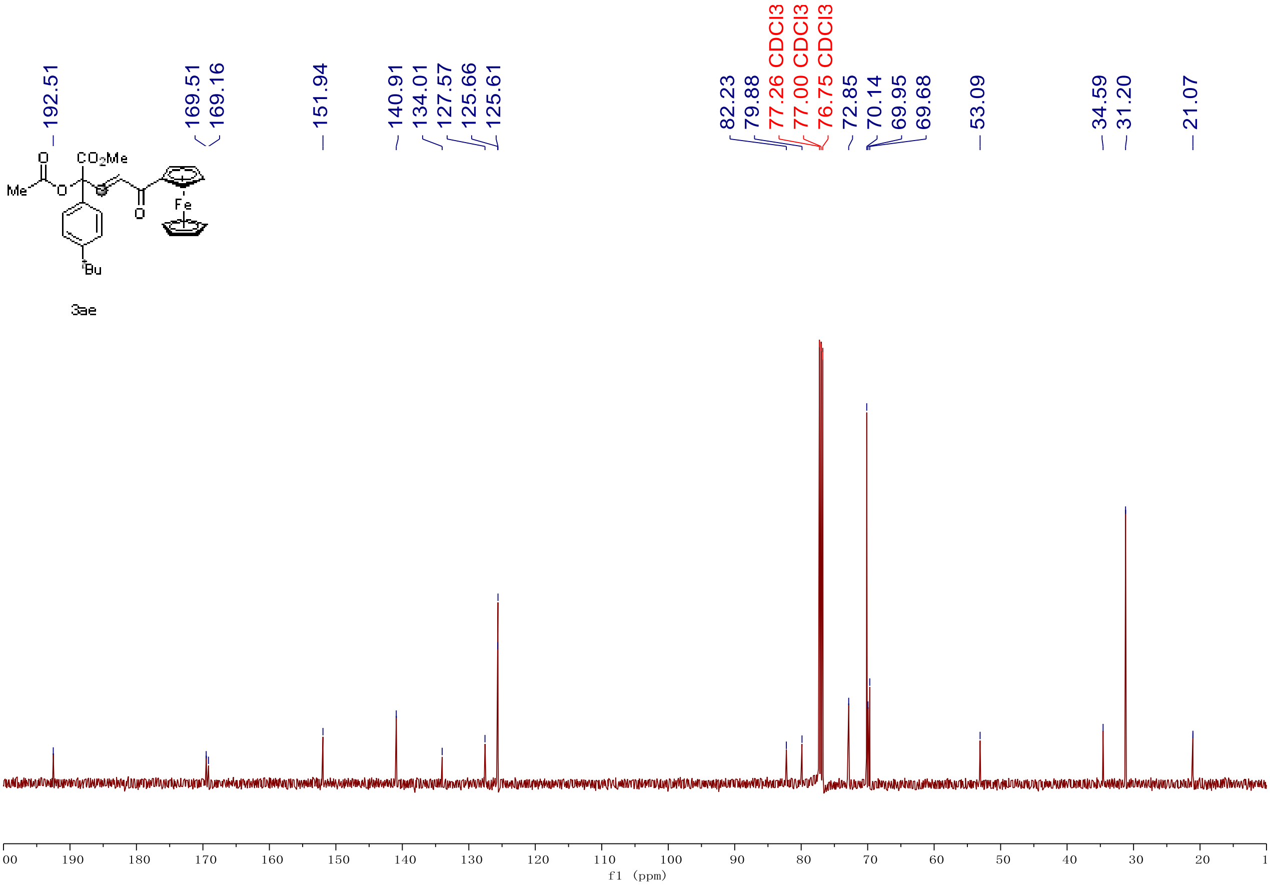

Supplementary Figure 74. ^{13}C NMR spectrum of **3ae**.

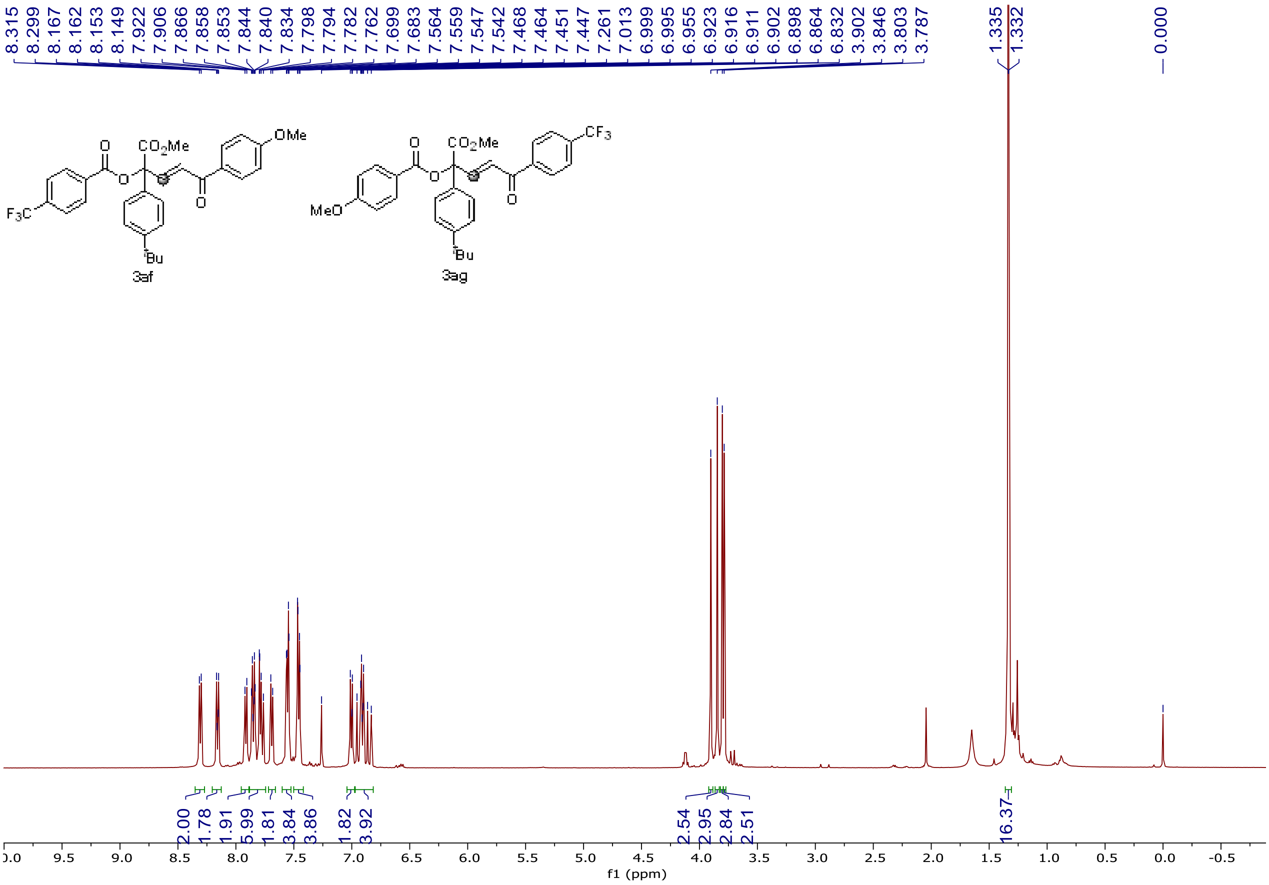

Supplementary Figure 75. ^{1}H NMR spectrum of **3af and 3ag**.

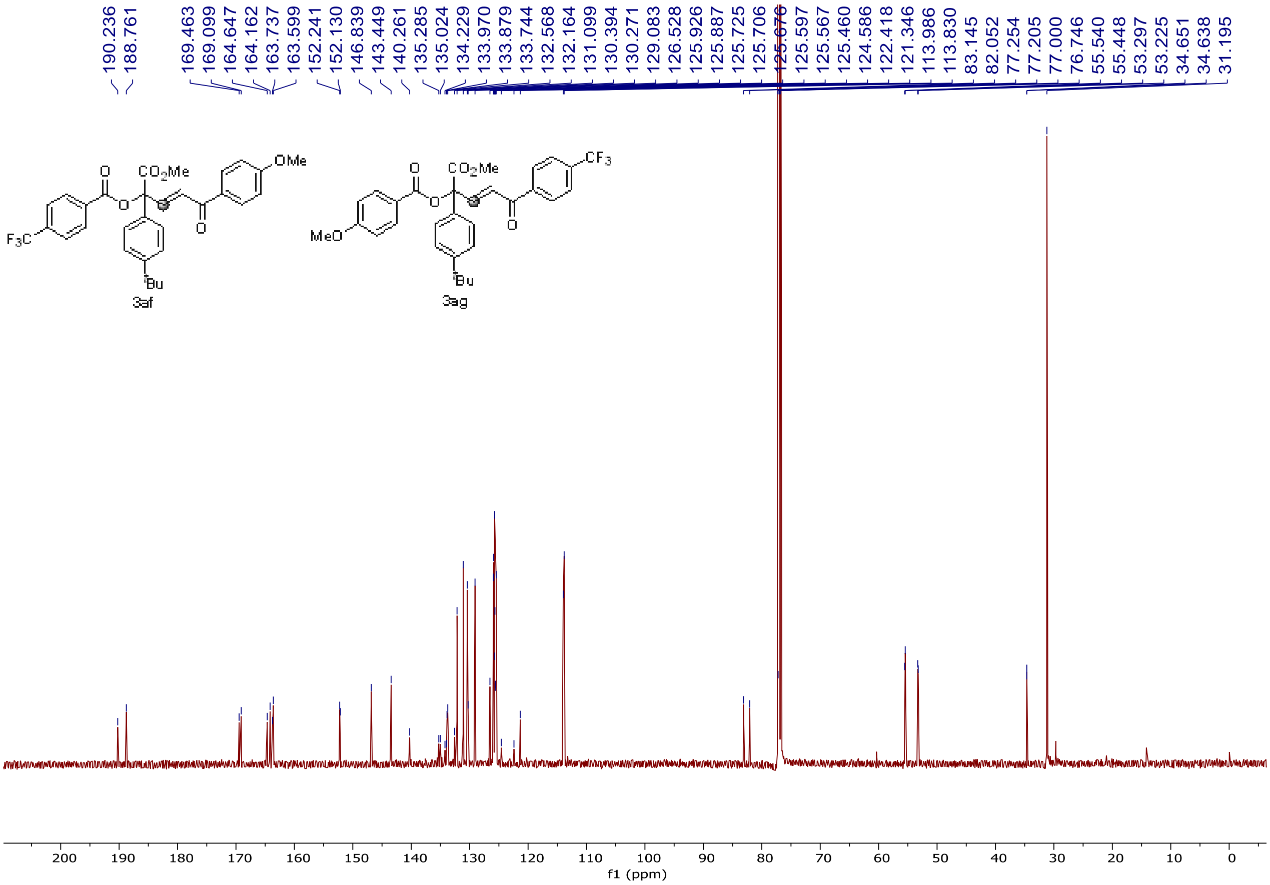

Supplementary Figure 76. ^{13}C NMR spectrum of **3af and 3ag**.

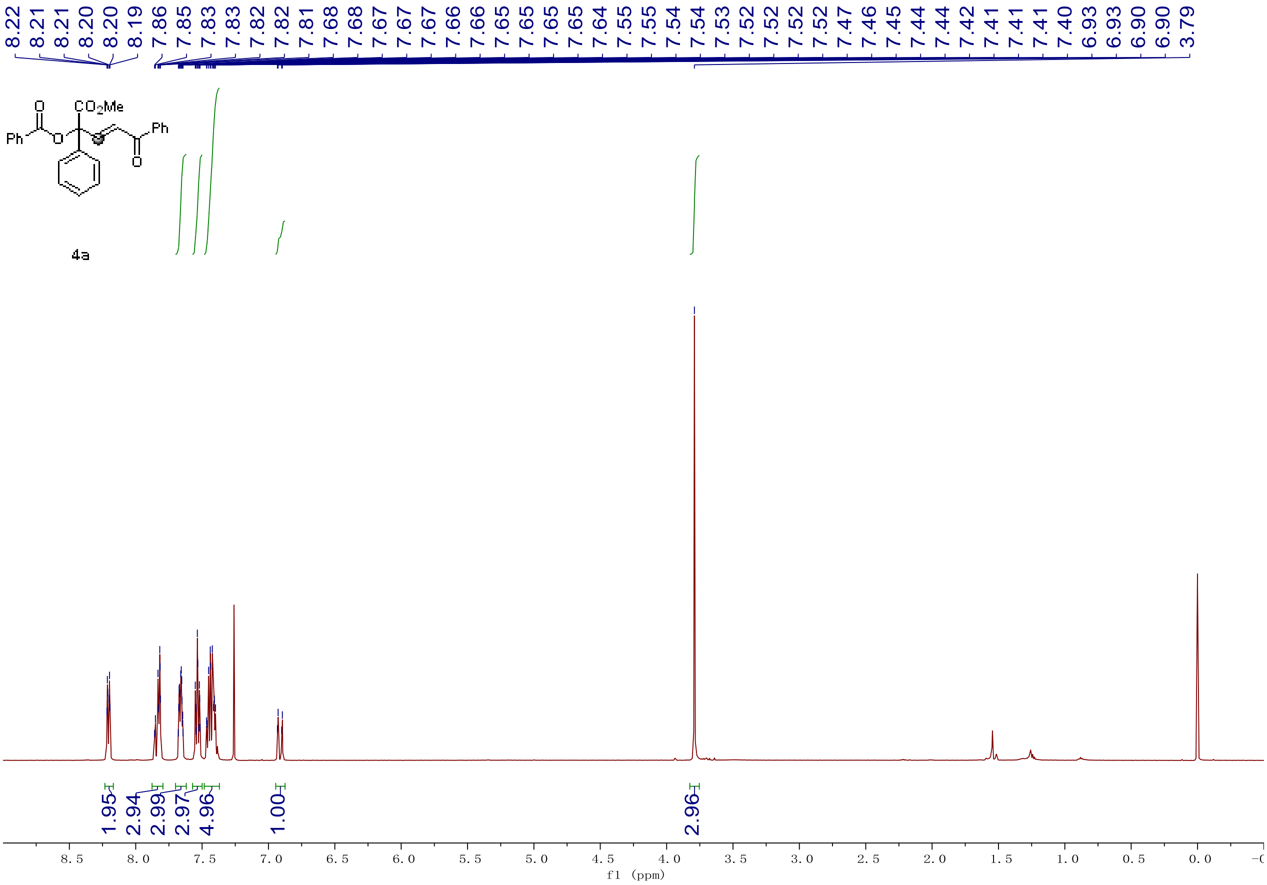

Supplementary Figure 77. ^{1}H NMR spectrum of **4a**.

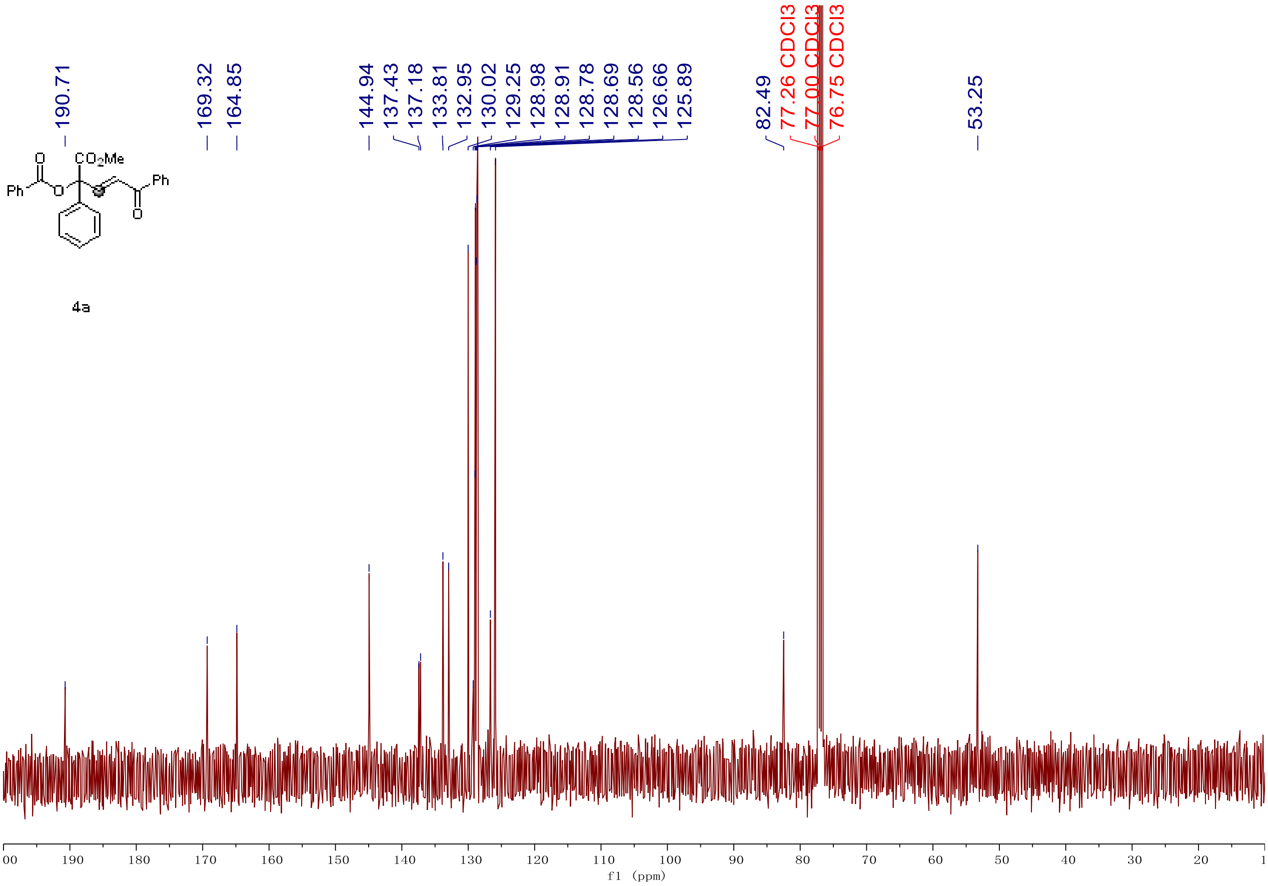

Supplementary Figure 78. ^{13}C NMR spectrum of **4a**.

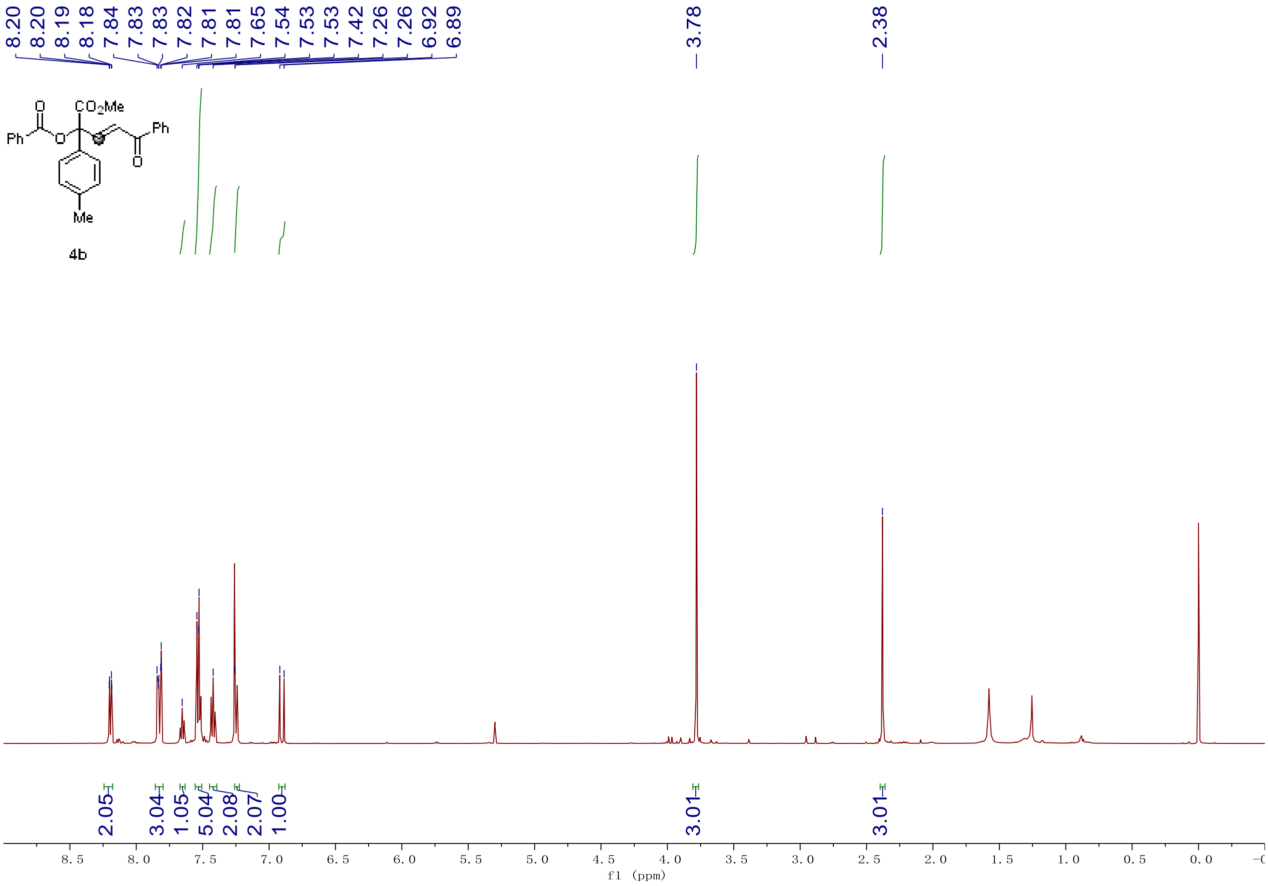

Supplementary Figure 79. ^{1}H NMR spectrum of **4b**.

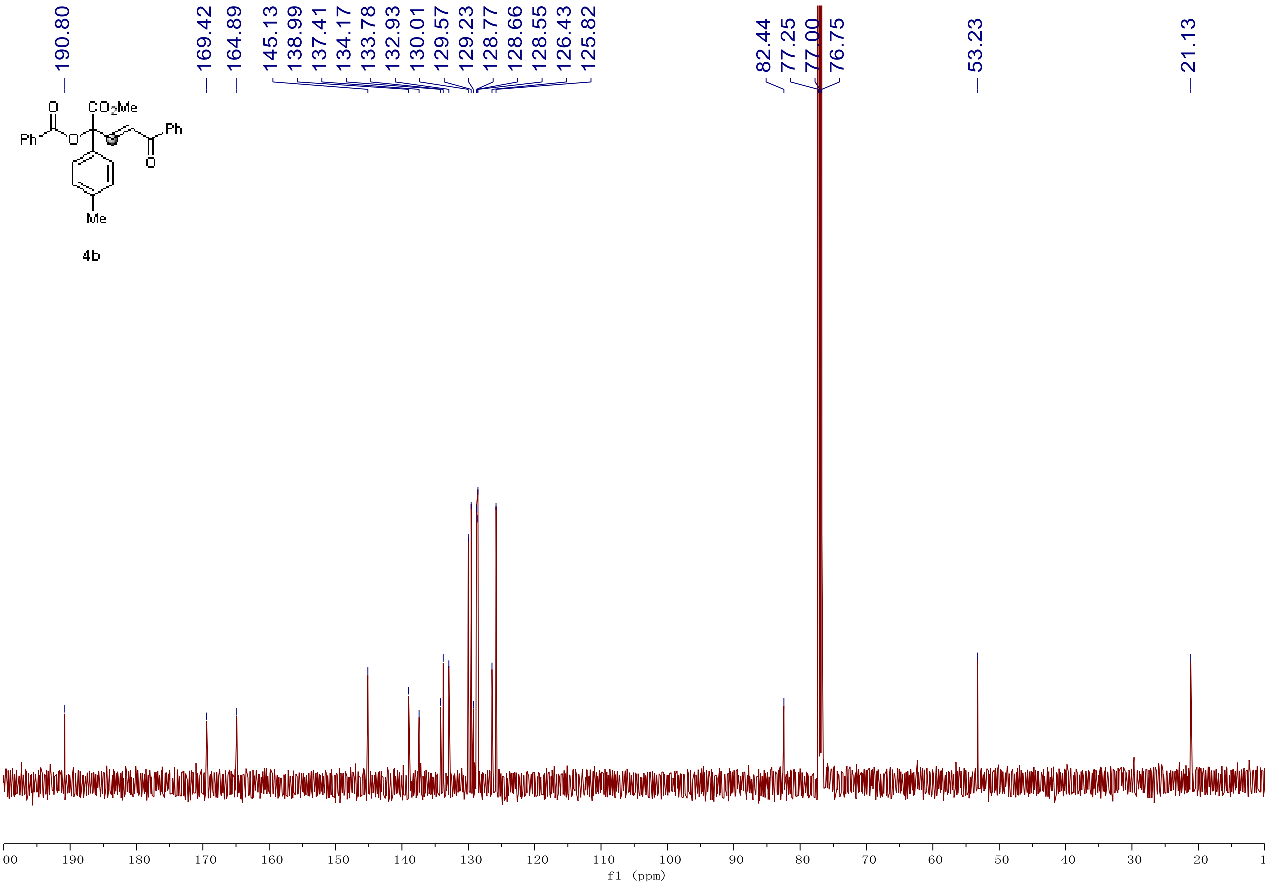

Supplementary Figure 80. ^{13}C NMR spectrum of **4b**.

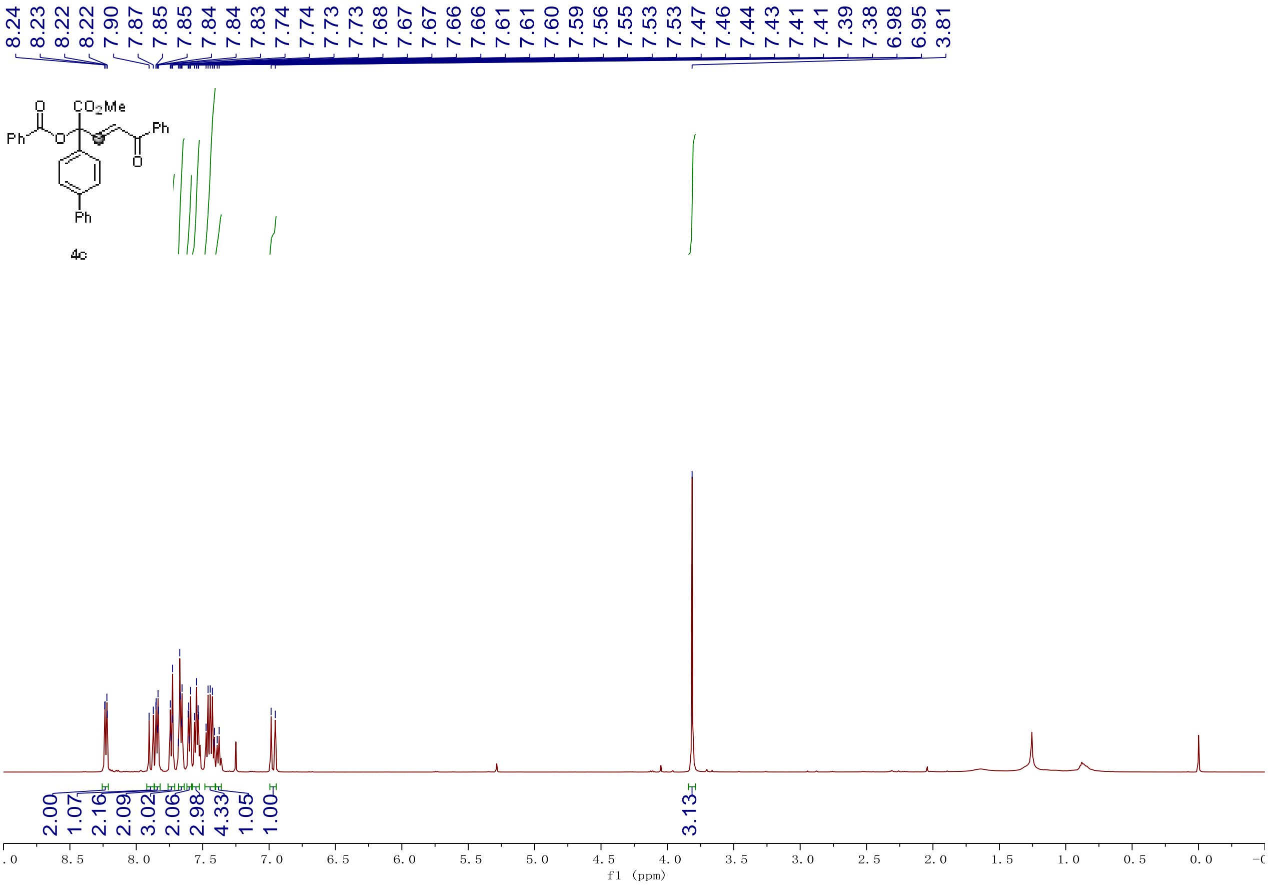

Supplementary Figure 81. ^{1}H NMR spectrum of **4c**.

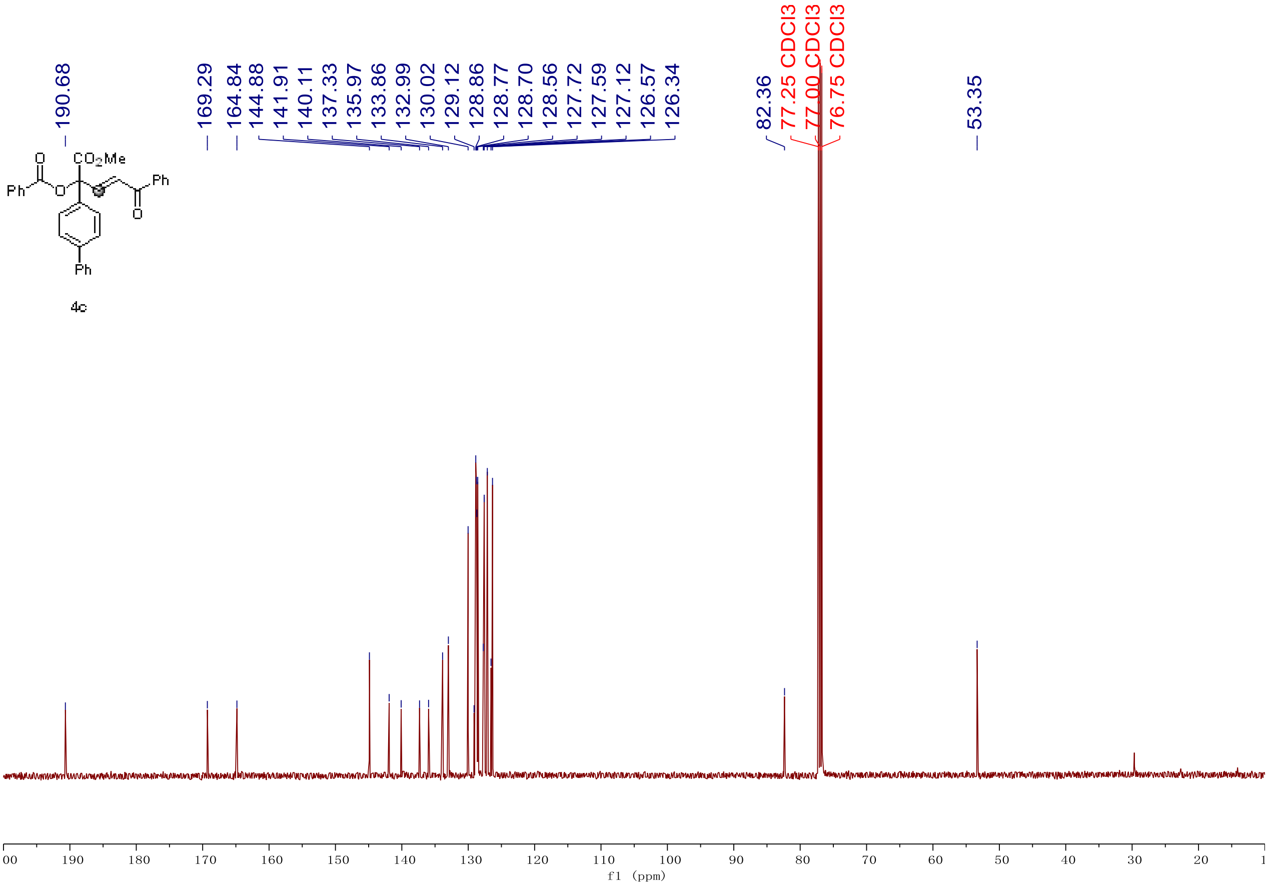

Supplementary Figure 82. ^{13}C NMR spectrum of **4c**.

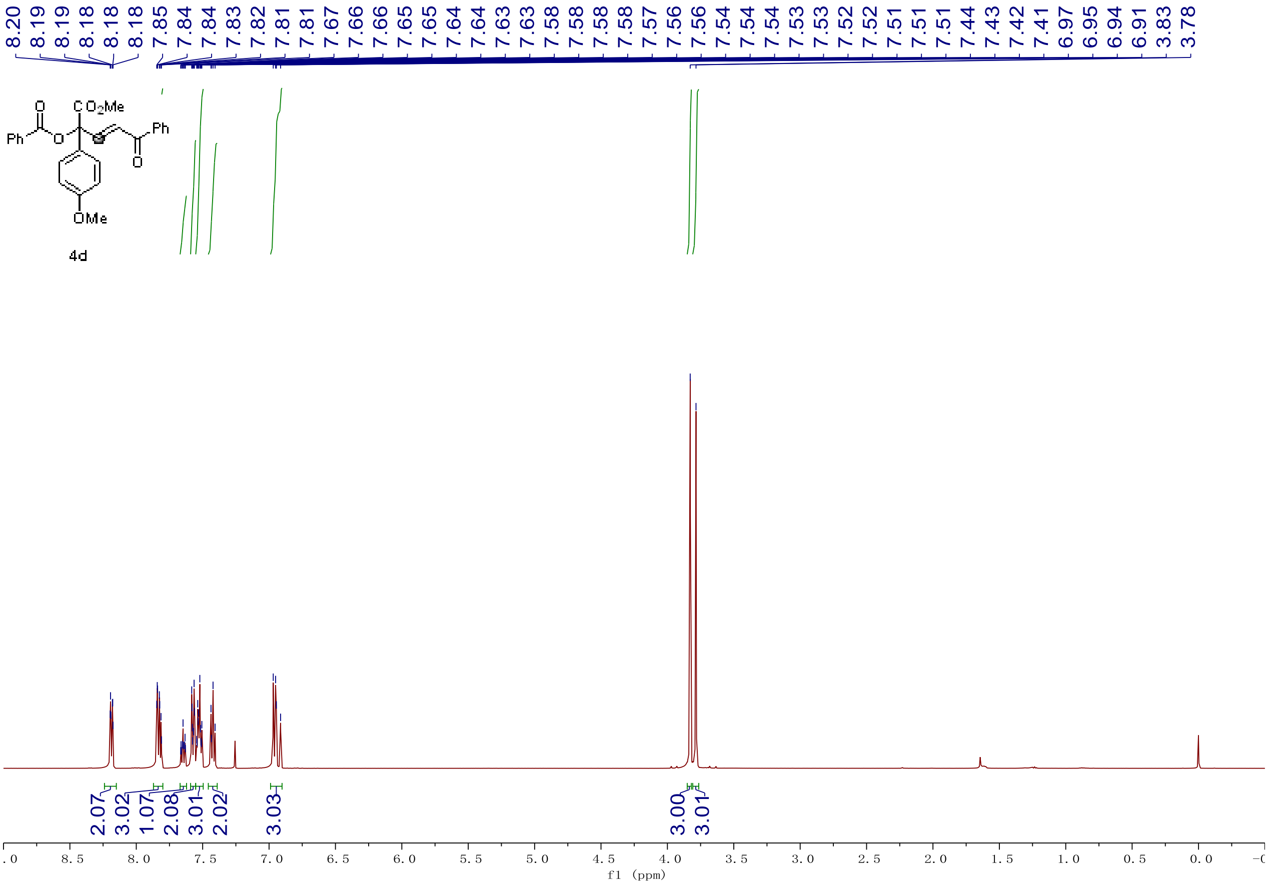

Supplementary Figure 83. ^{1}H NMR spectrum of **4d**.

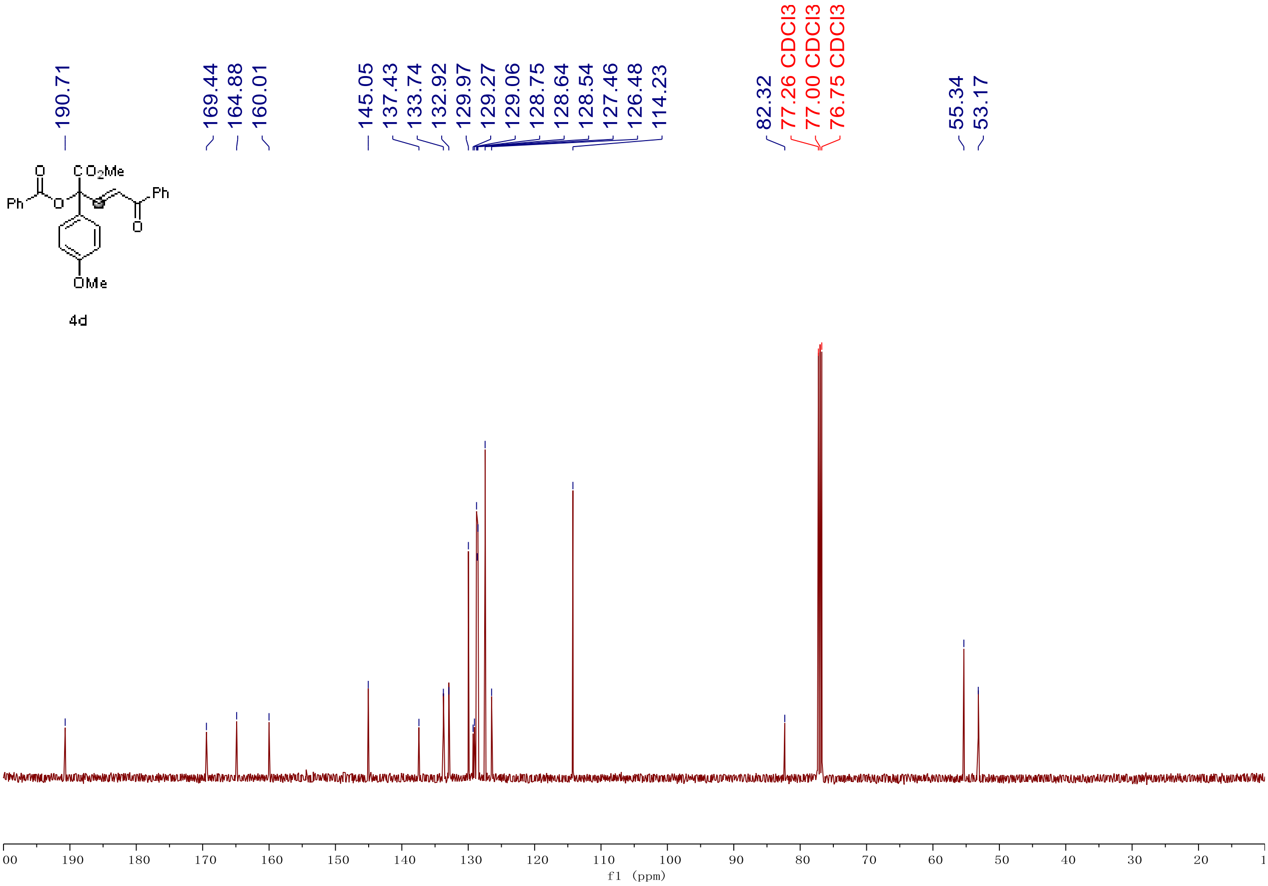

Supplementary Figure 84. ^{13}C NMR spectrum of **4d**.

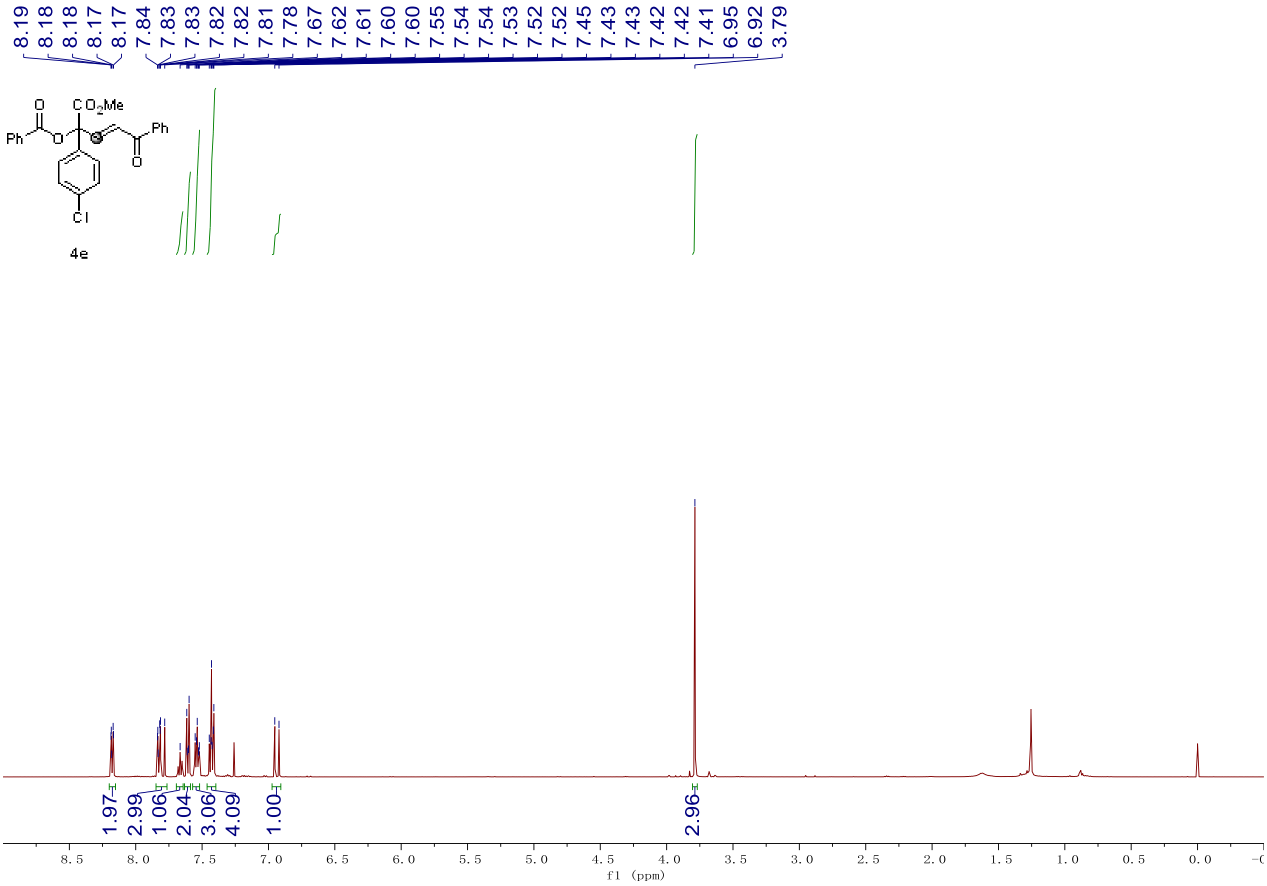

Supplementary Figure 85. ^{1}H NMR spectrum of **4e**.

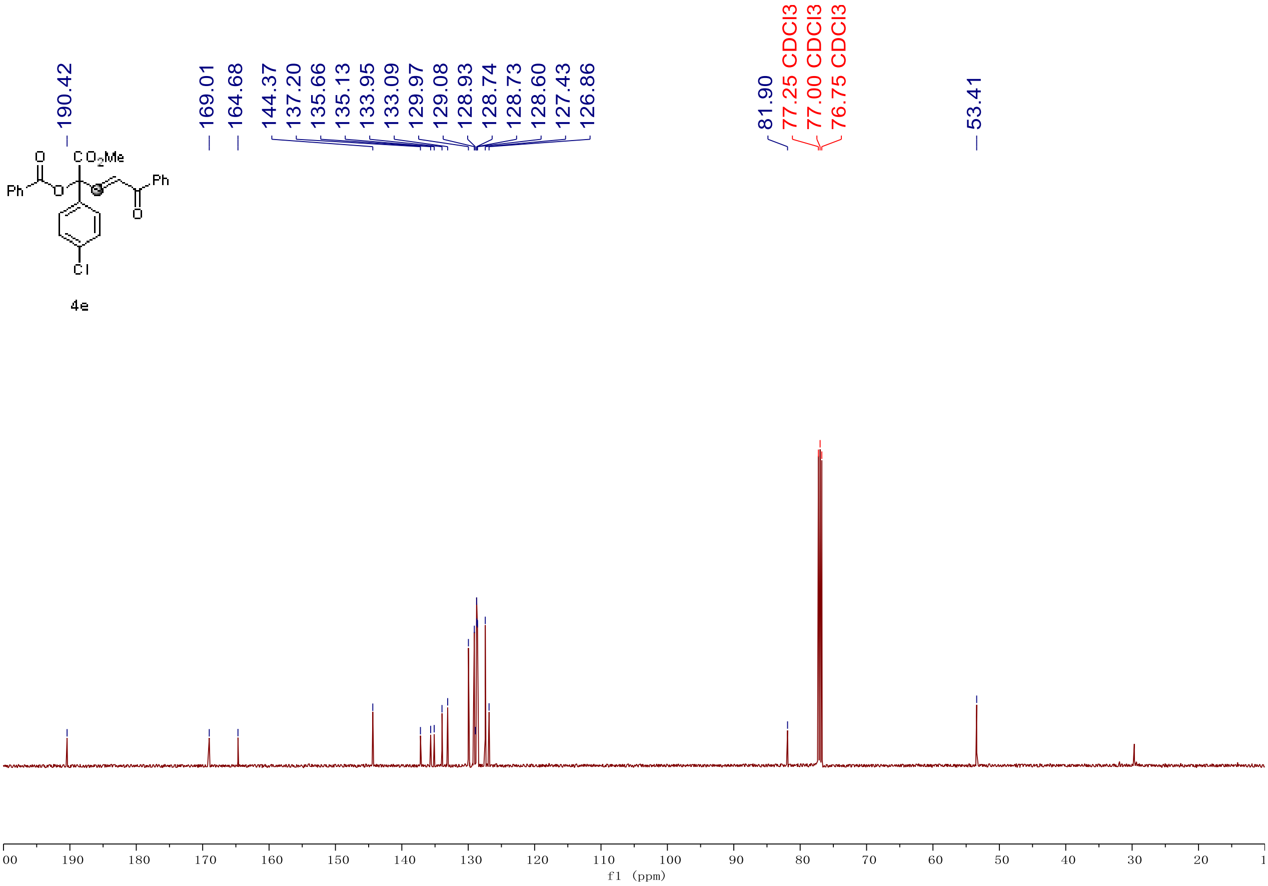

Supplementary Figure 86. ^{13}C NMR spectrum of **4e**.

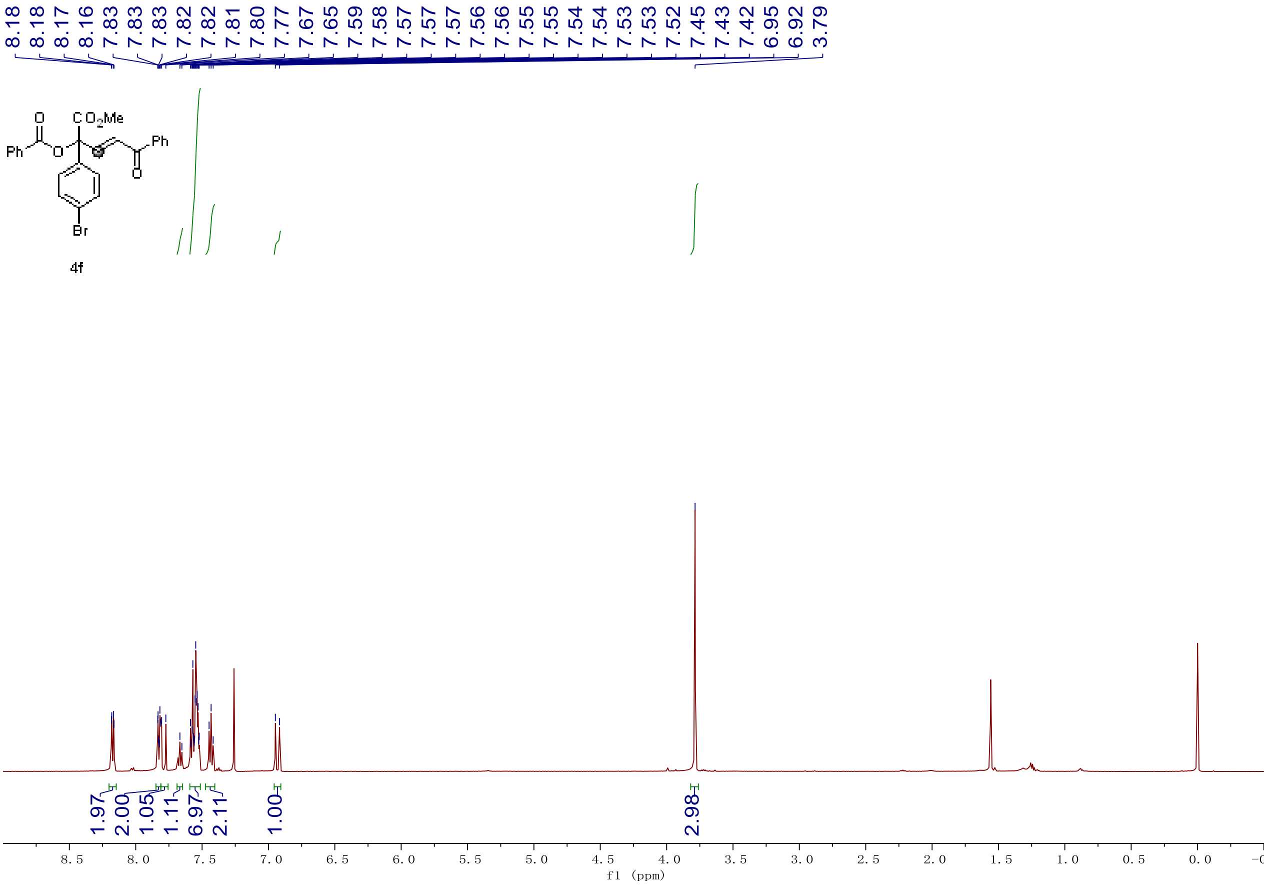

Supplementary Figure 87. ^{1}H NMR spectrum of **4f**.

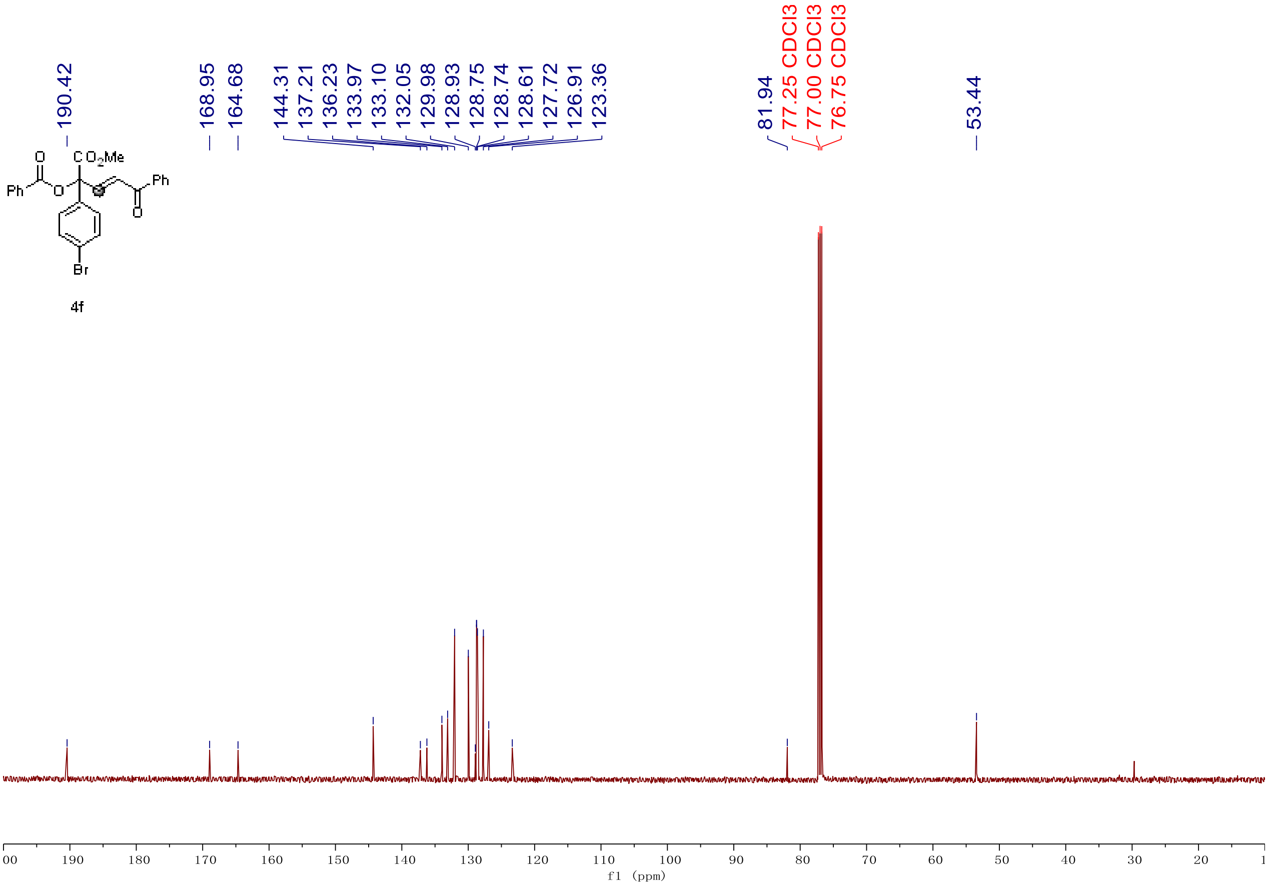

Supplementary Figure 88. ^{13}C NMR spectrum of **4f**.

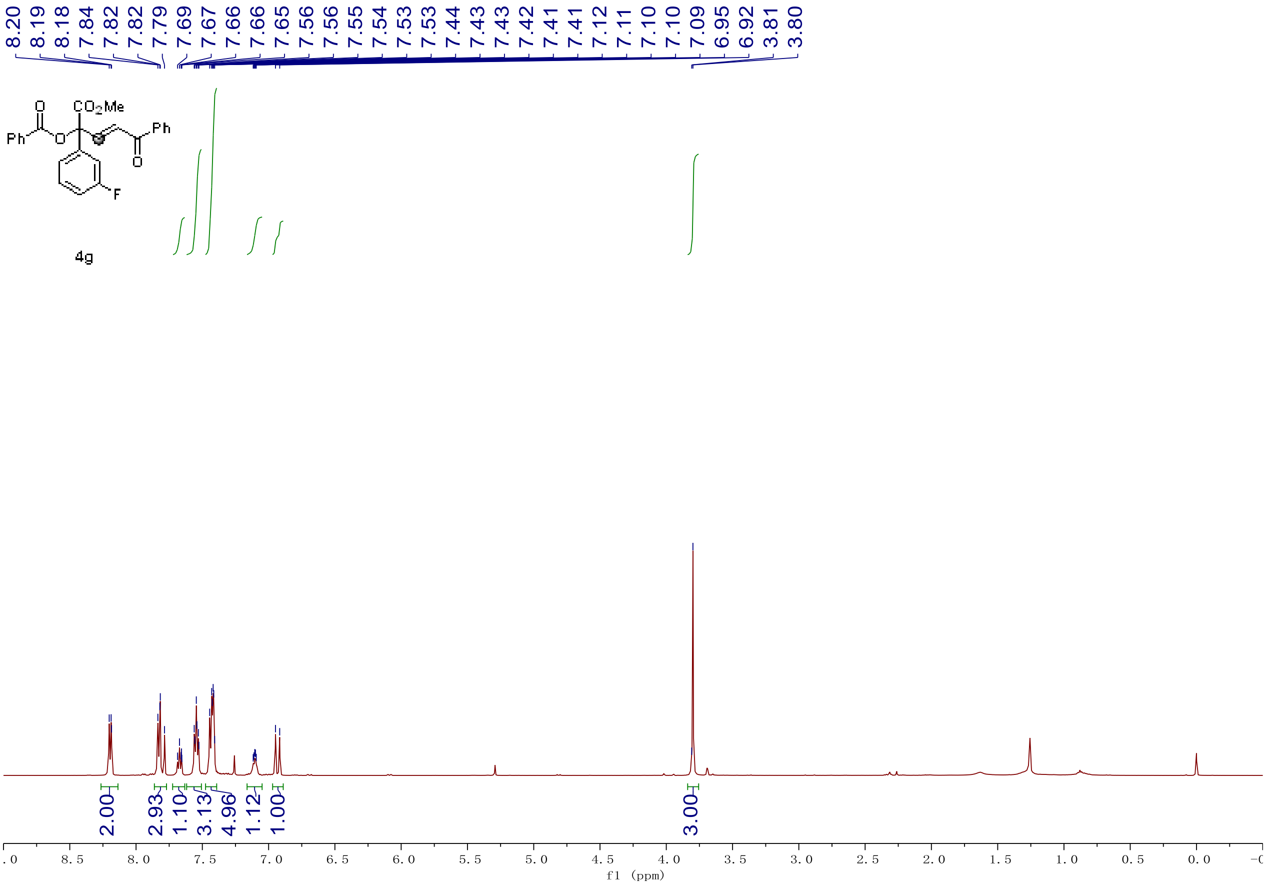

Supplementary Figure 89. ^{1}H NMR spectrum of **4g**.

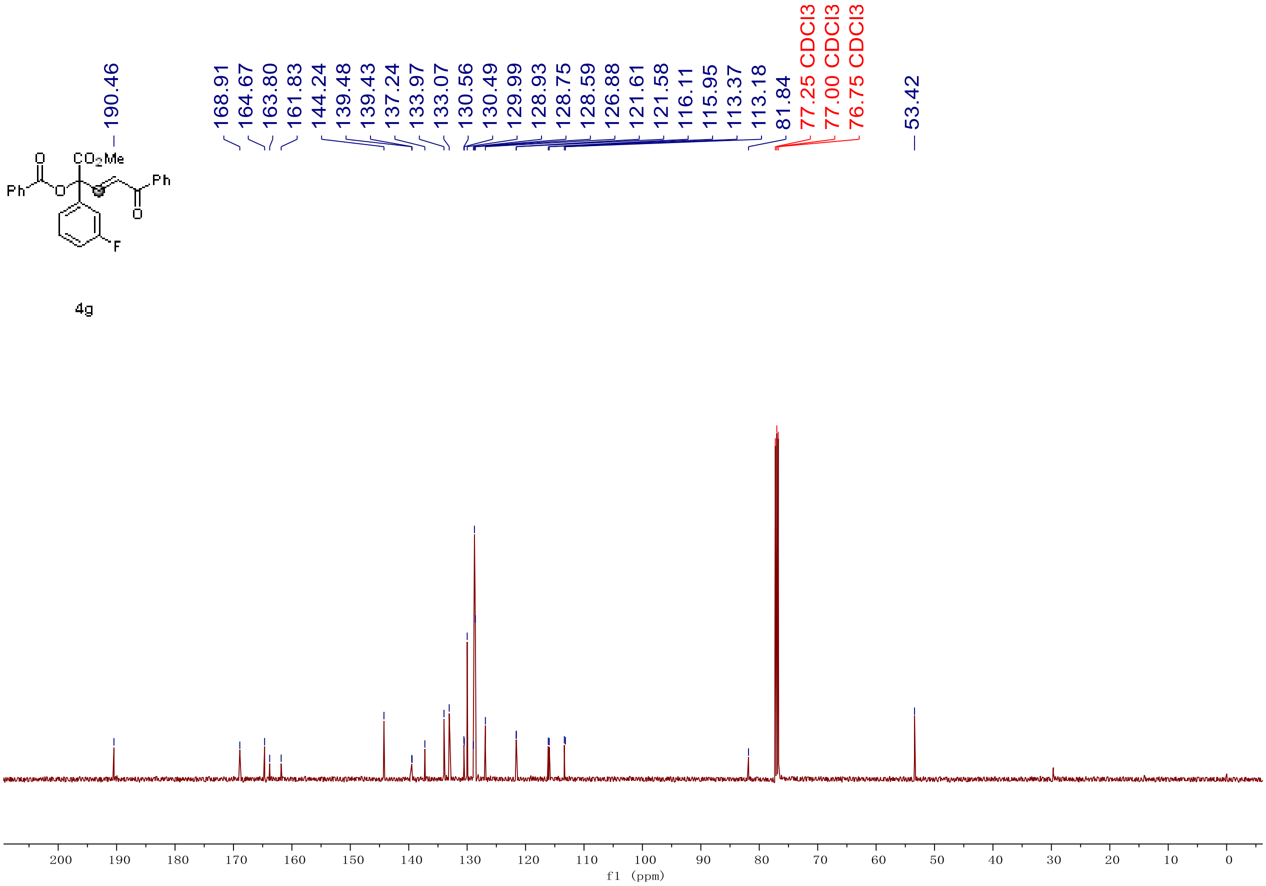

Supplementary Figure 90. ^{13}C NMR spectrum of **4g**.

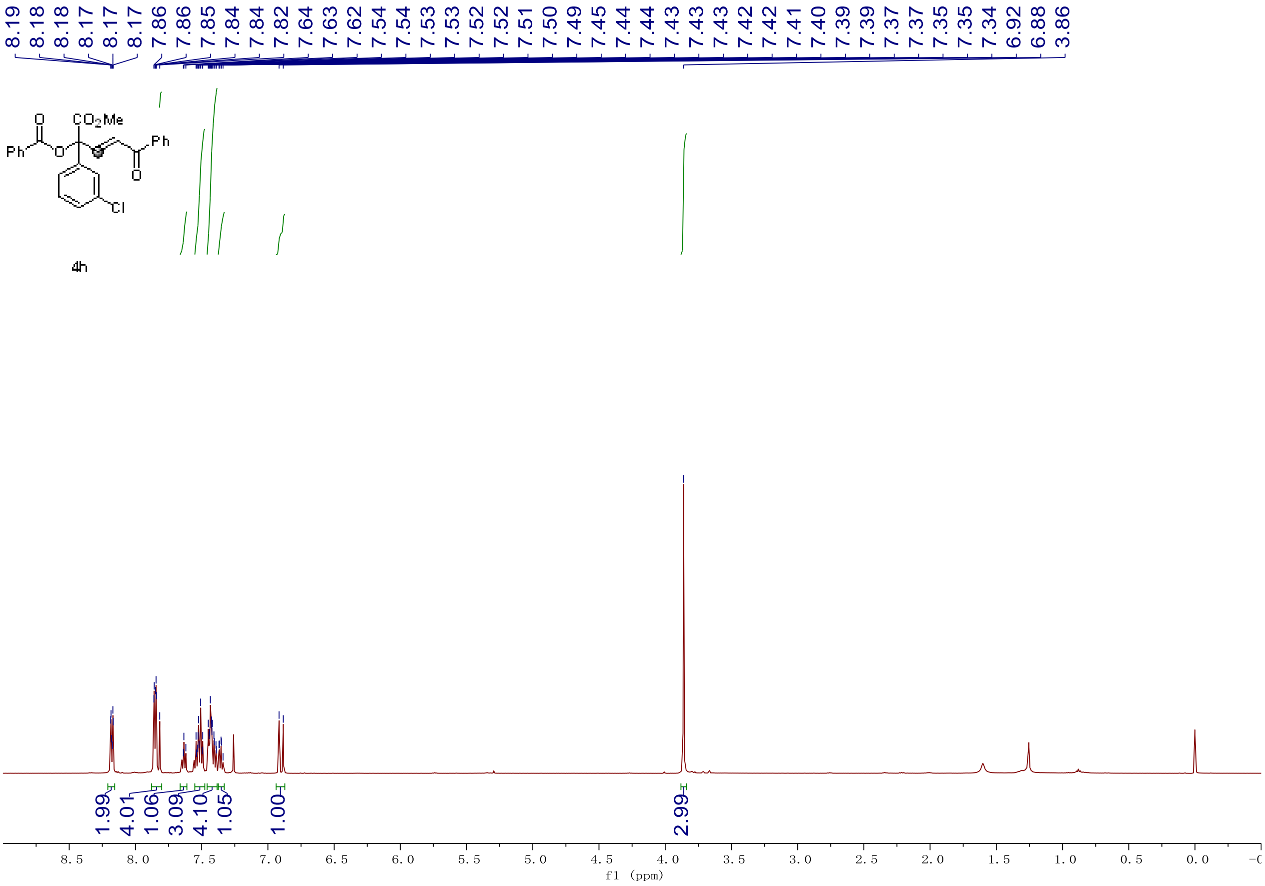

Supplementary Figure 91. ^{1}H NMR spectrum of **4h**.

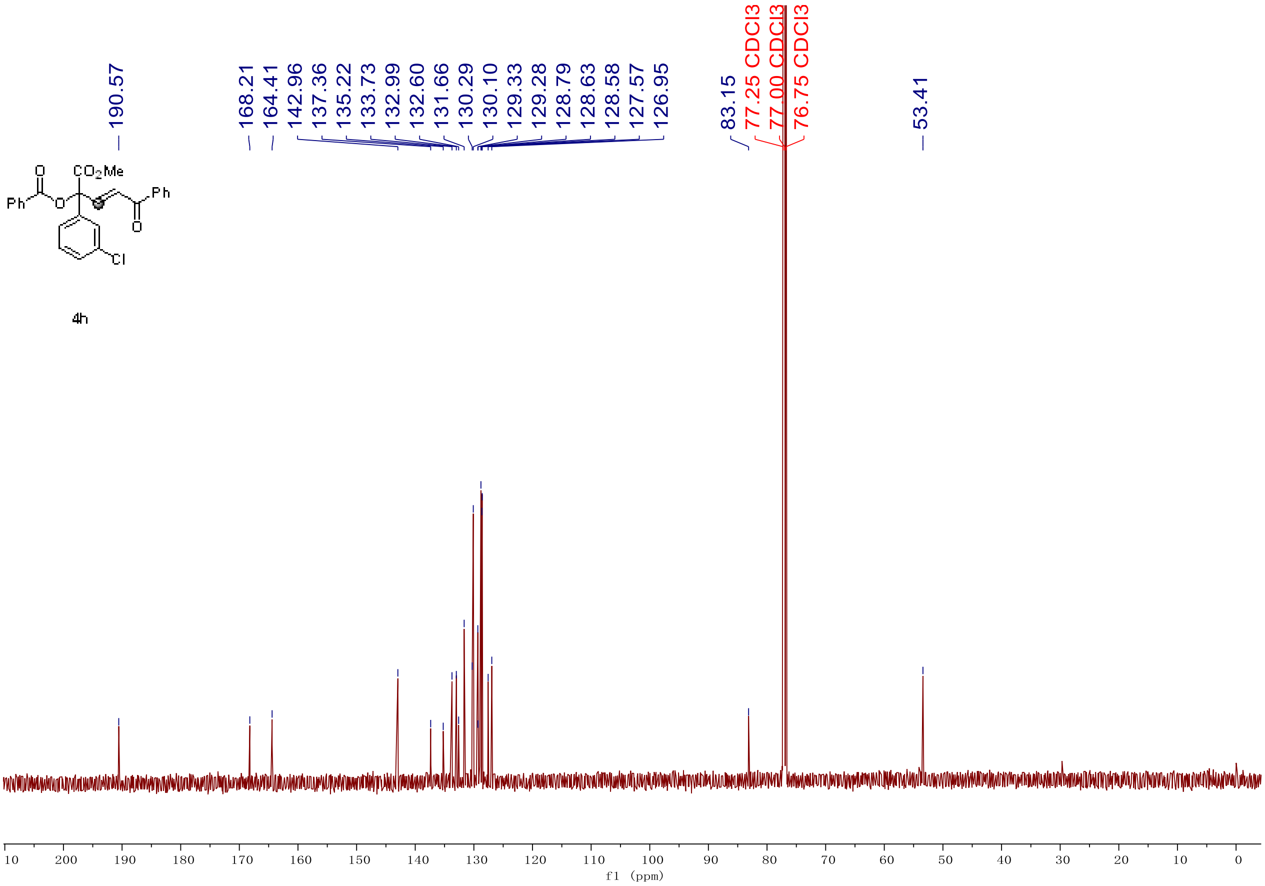

Supplementary Figure 92. ^{13}C NMR spectrum of **4h**.

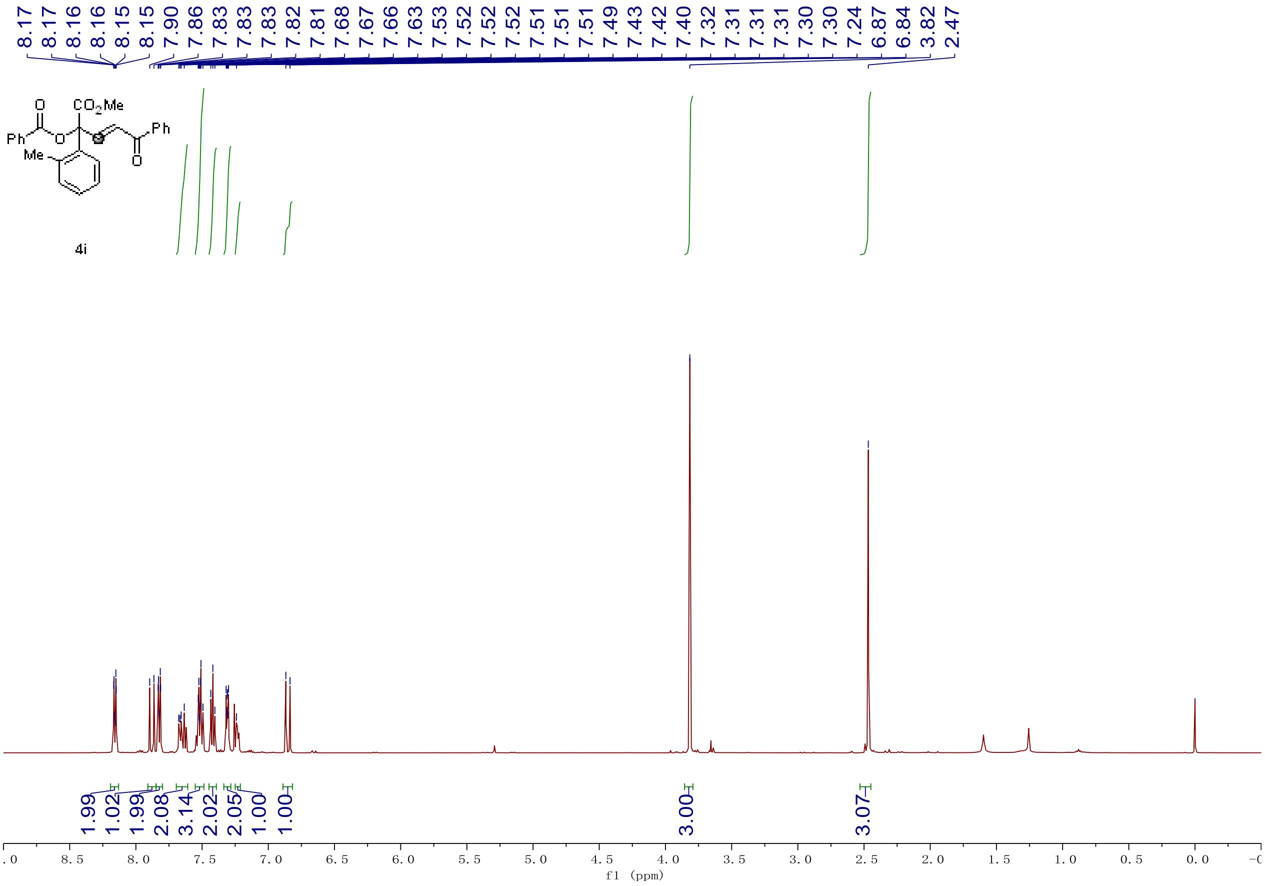

Supplementary Figure 93. ^{1}H NMR spectrum of **4i**.

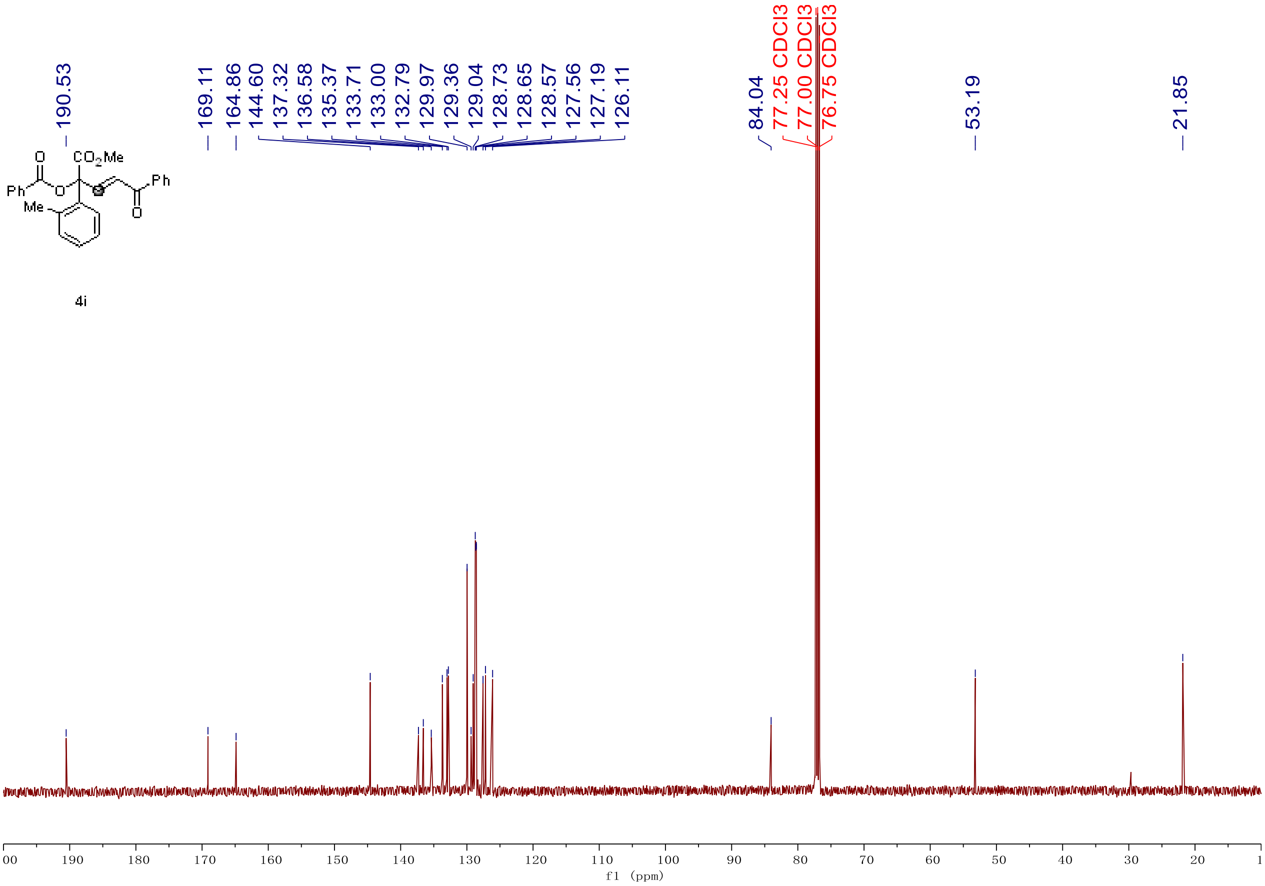

Supplementary Figure 94. ^{13}C NMR spectrum of **4i**.

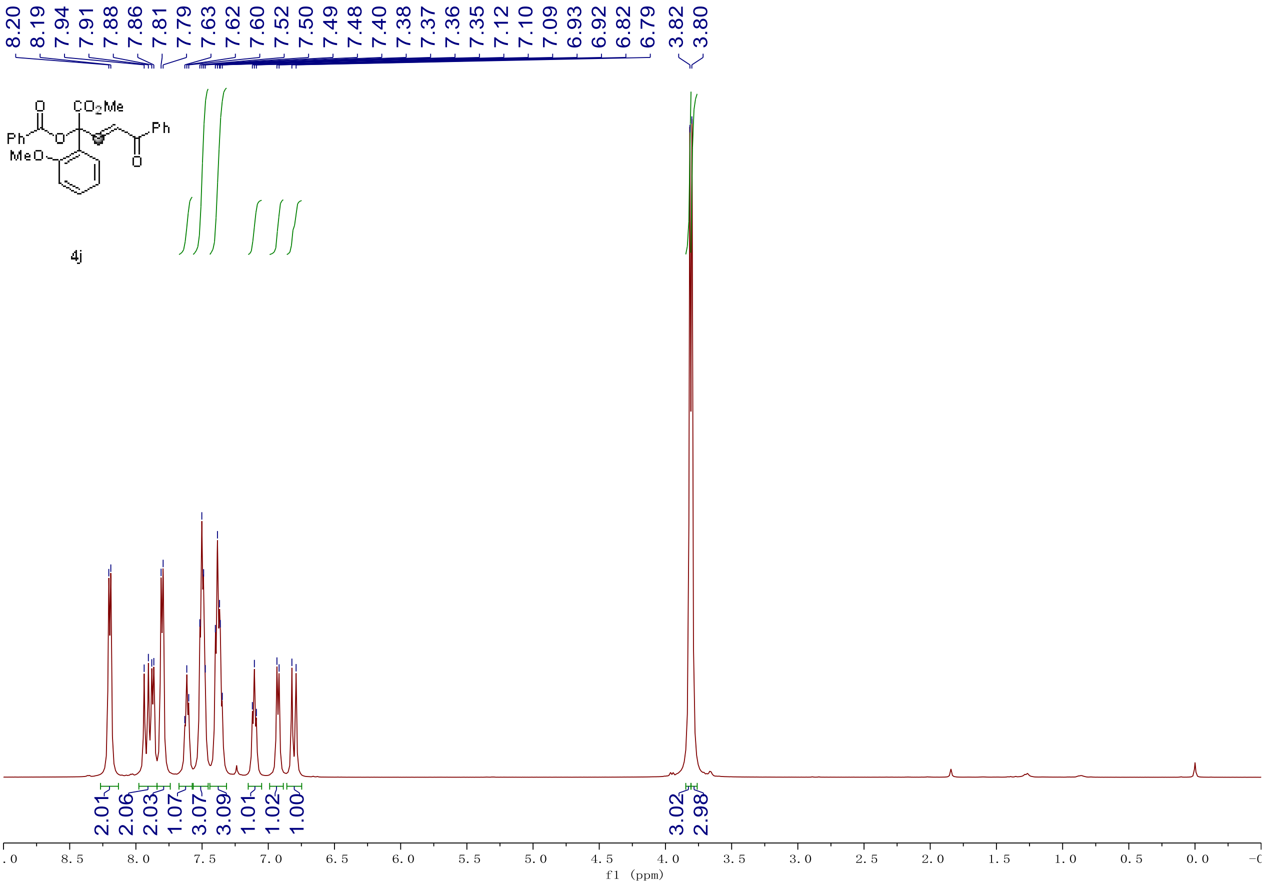

Supplementary Figure 95. ^{1}H NMR spectrum of **4j**.

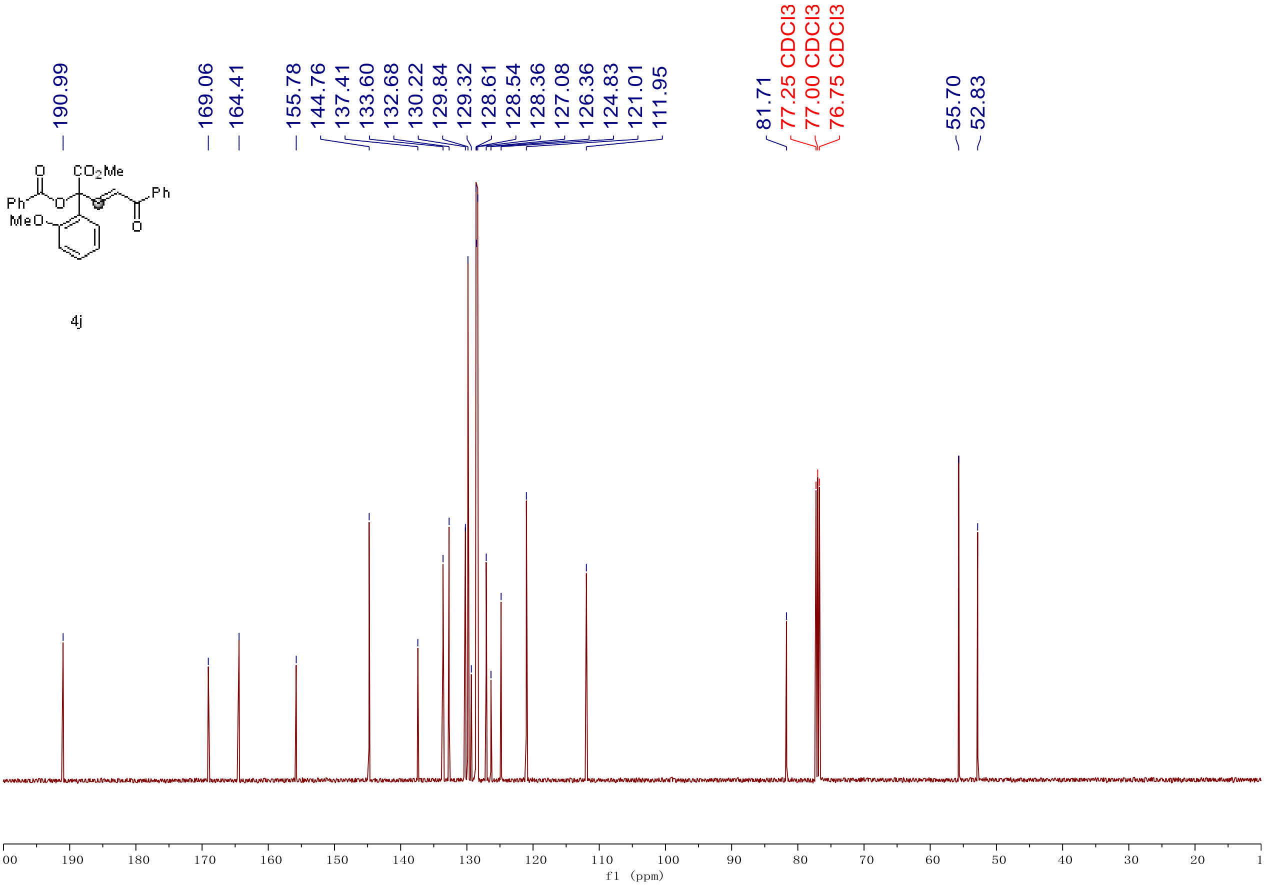

Supplementary Figure 96. ^{13}C NMR spectrum of **4j**.

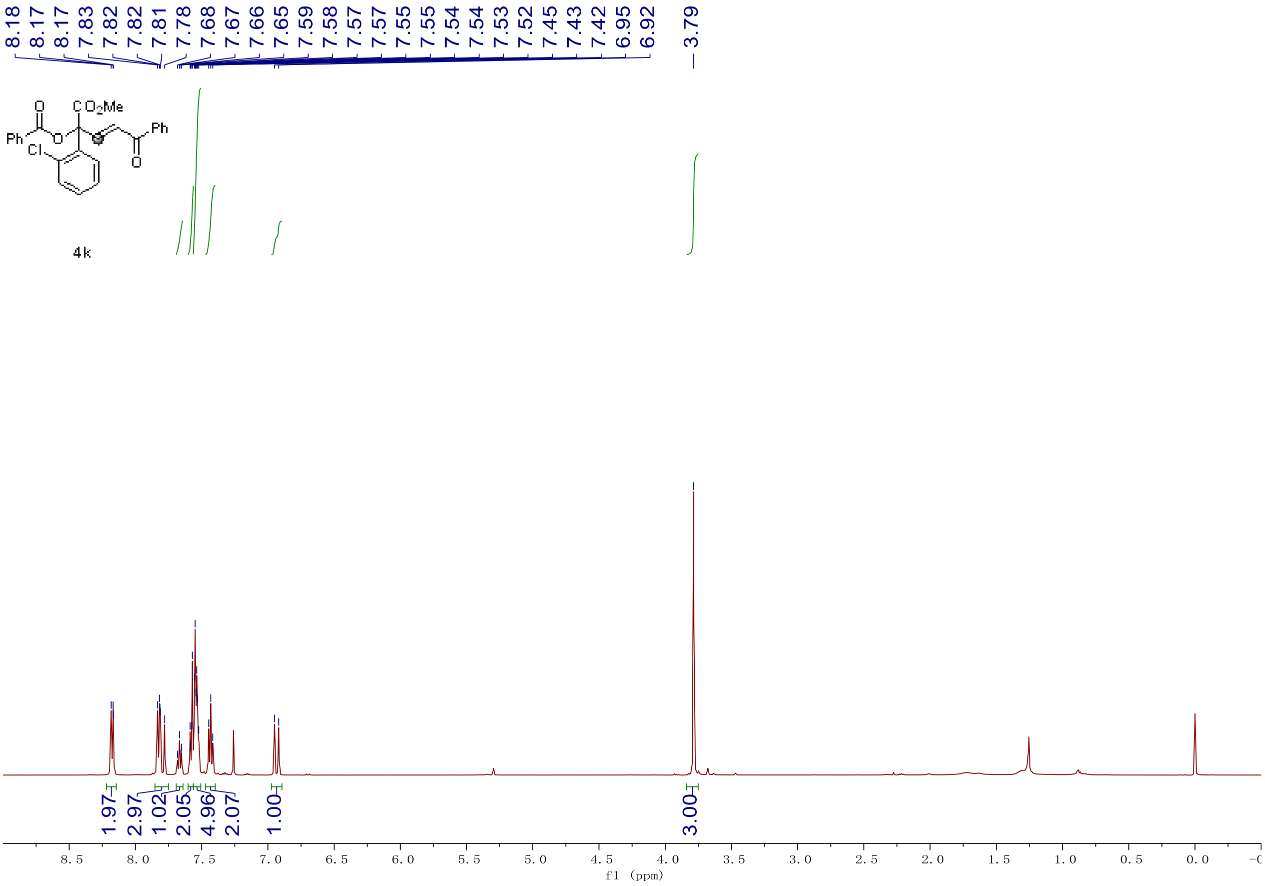

Supplementary Figure 97. ^{1}H NMR spectrum of **4k**.

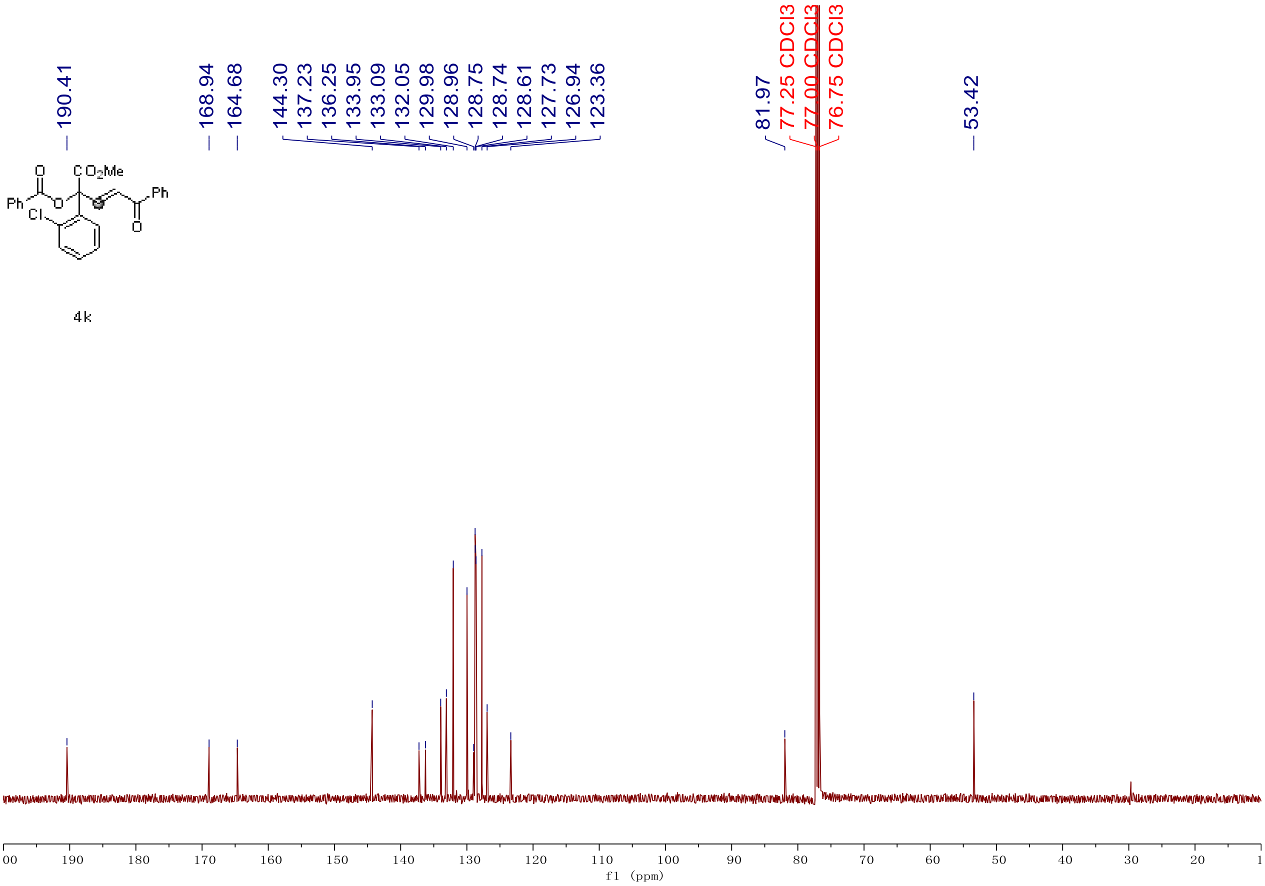

Supplementary Figure 98. ^{13}C NMR spectrum of **4k**.

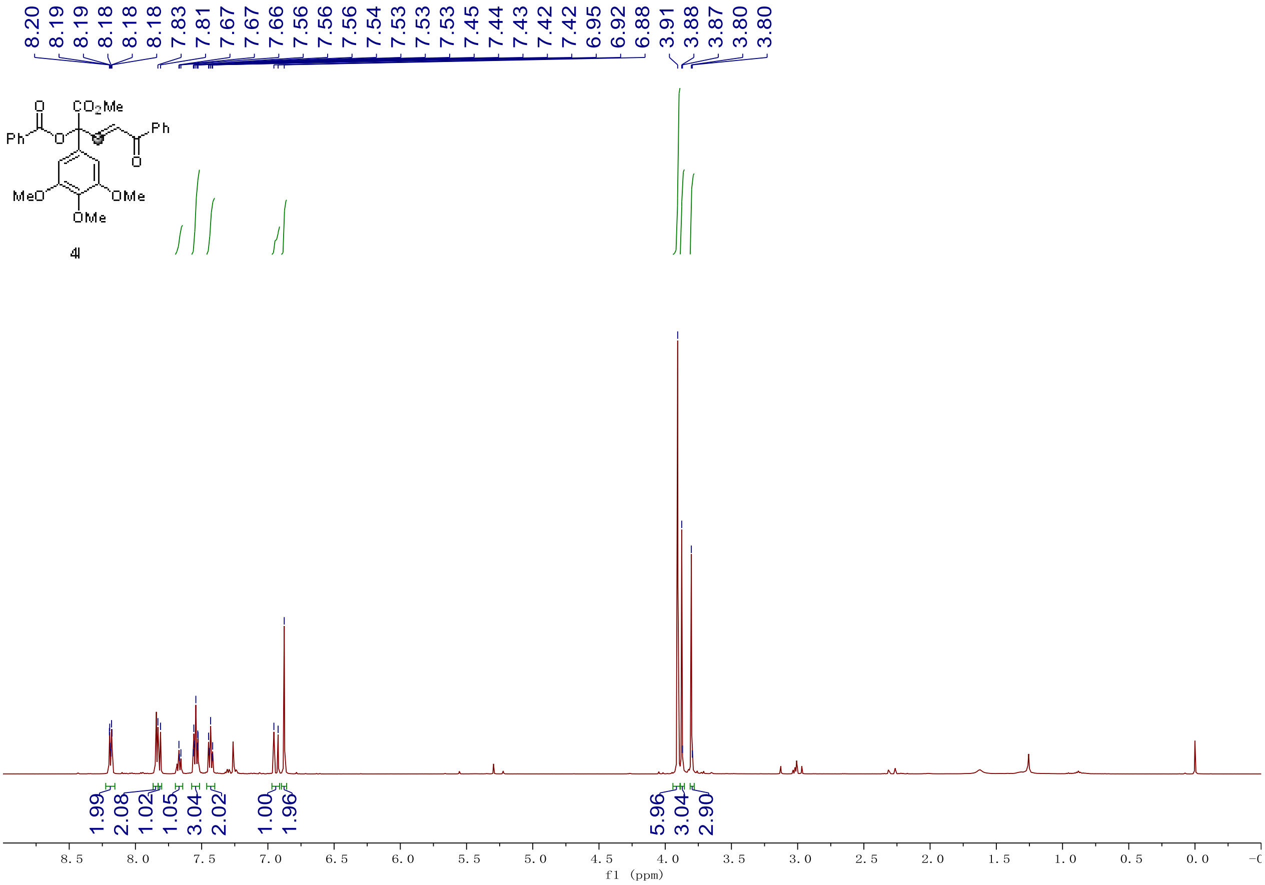

Supplementary Figure 99. ^{1}H NMR spectrum of **4l**.

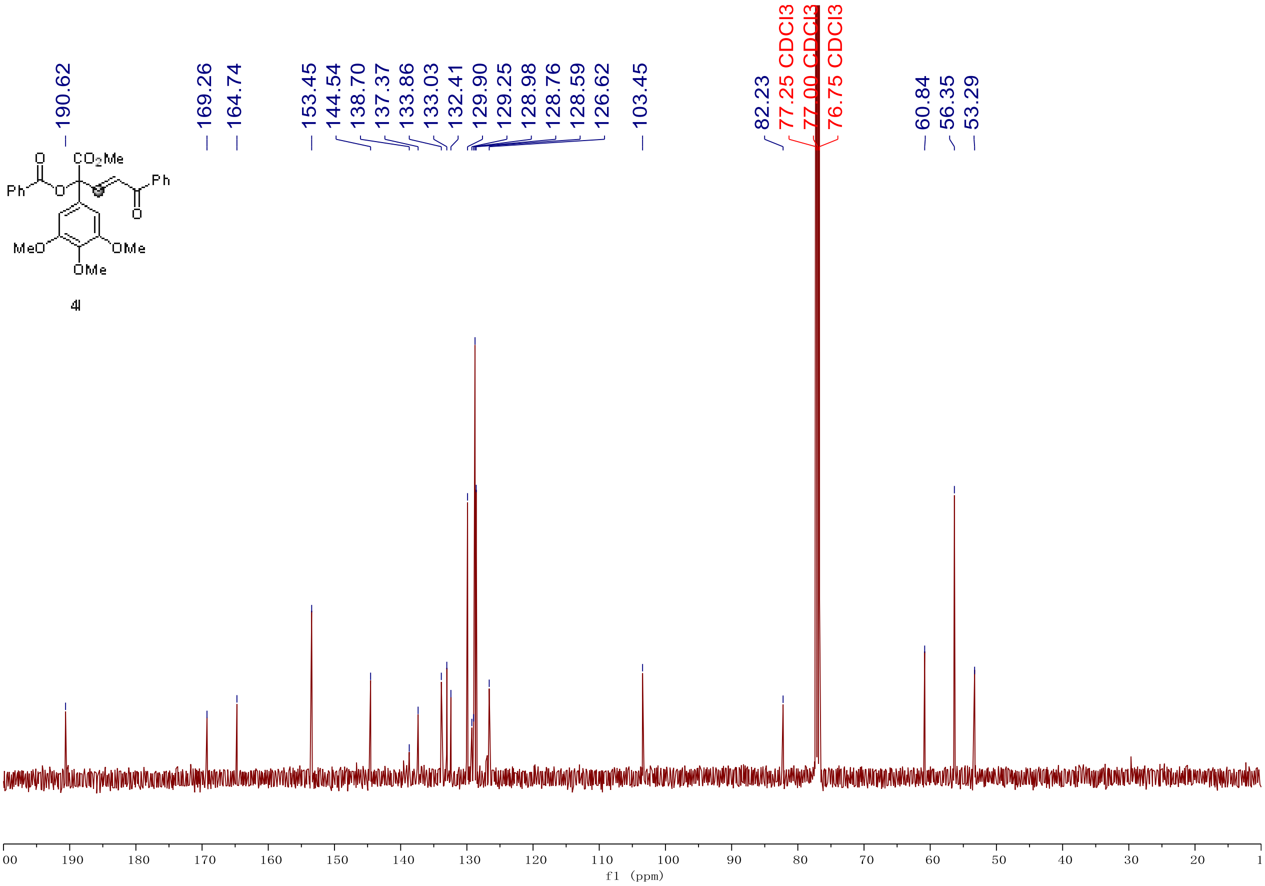

Supplementary Figure 100. ^{13}C NMR spectrum of **4l**.

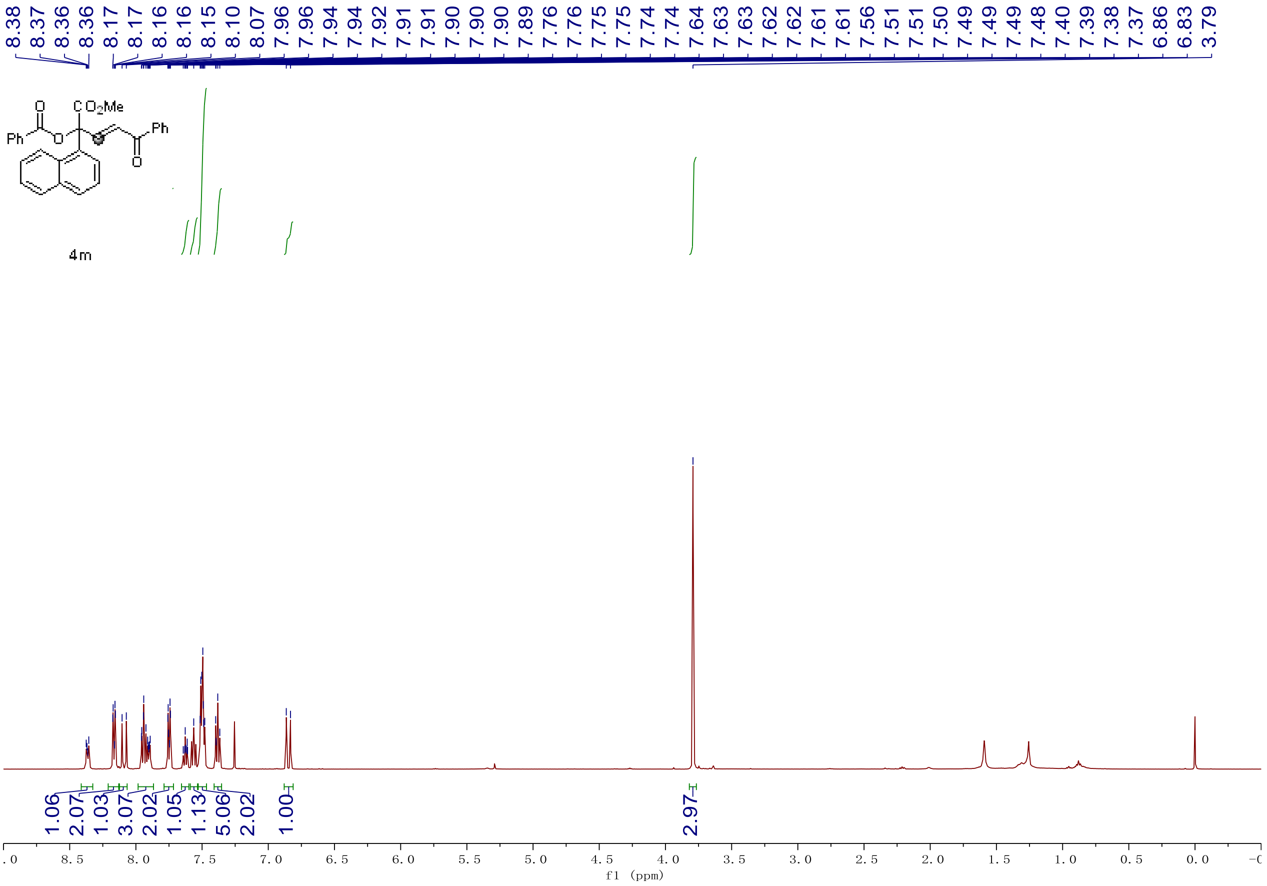

Supplementary Figure 101. ^{1}H NMR spectrum of **4m**.

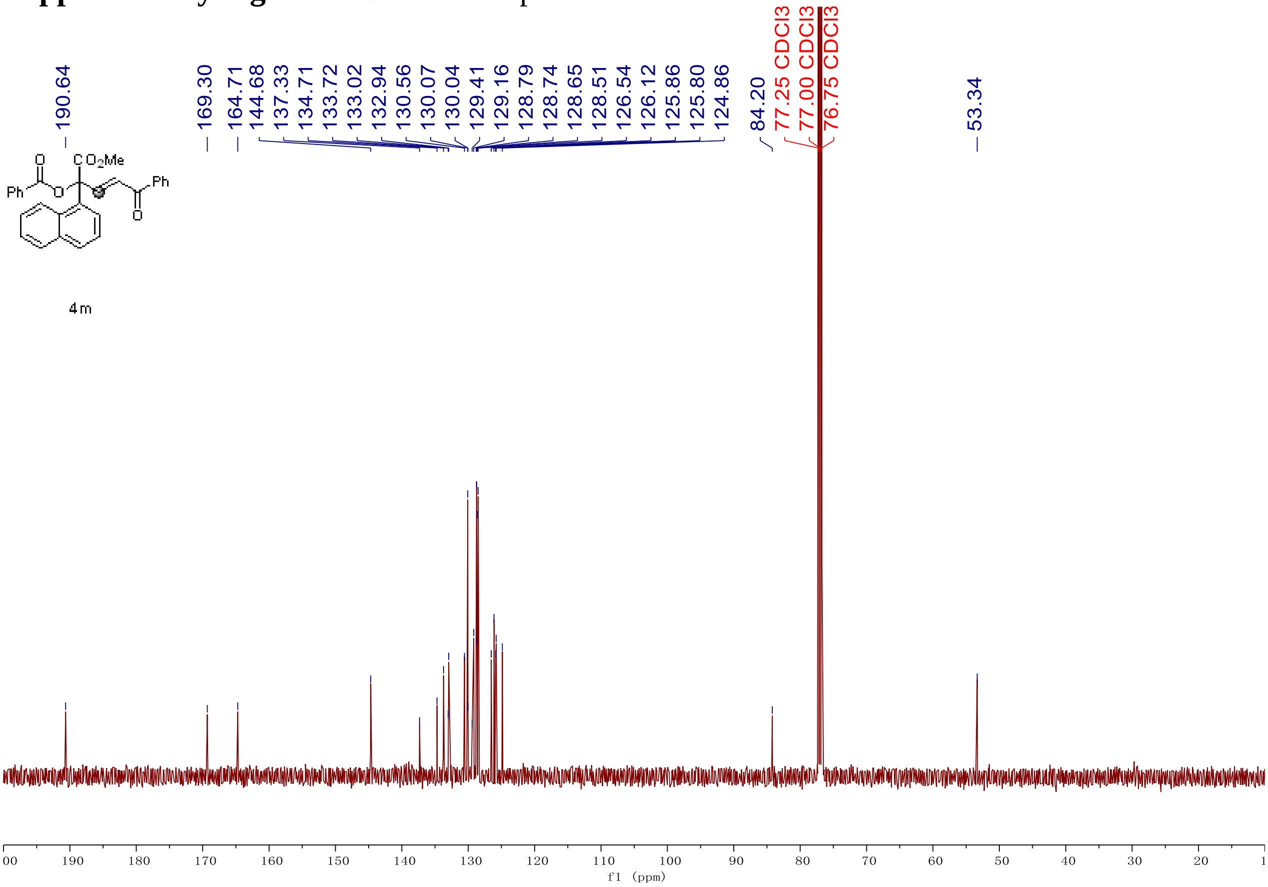

Supplementary Figure 102. ^{13}C NMR spectrum of **4m**.

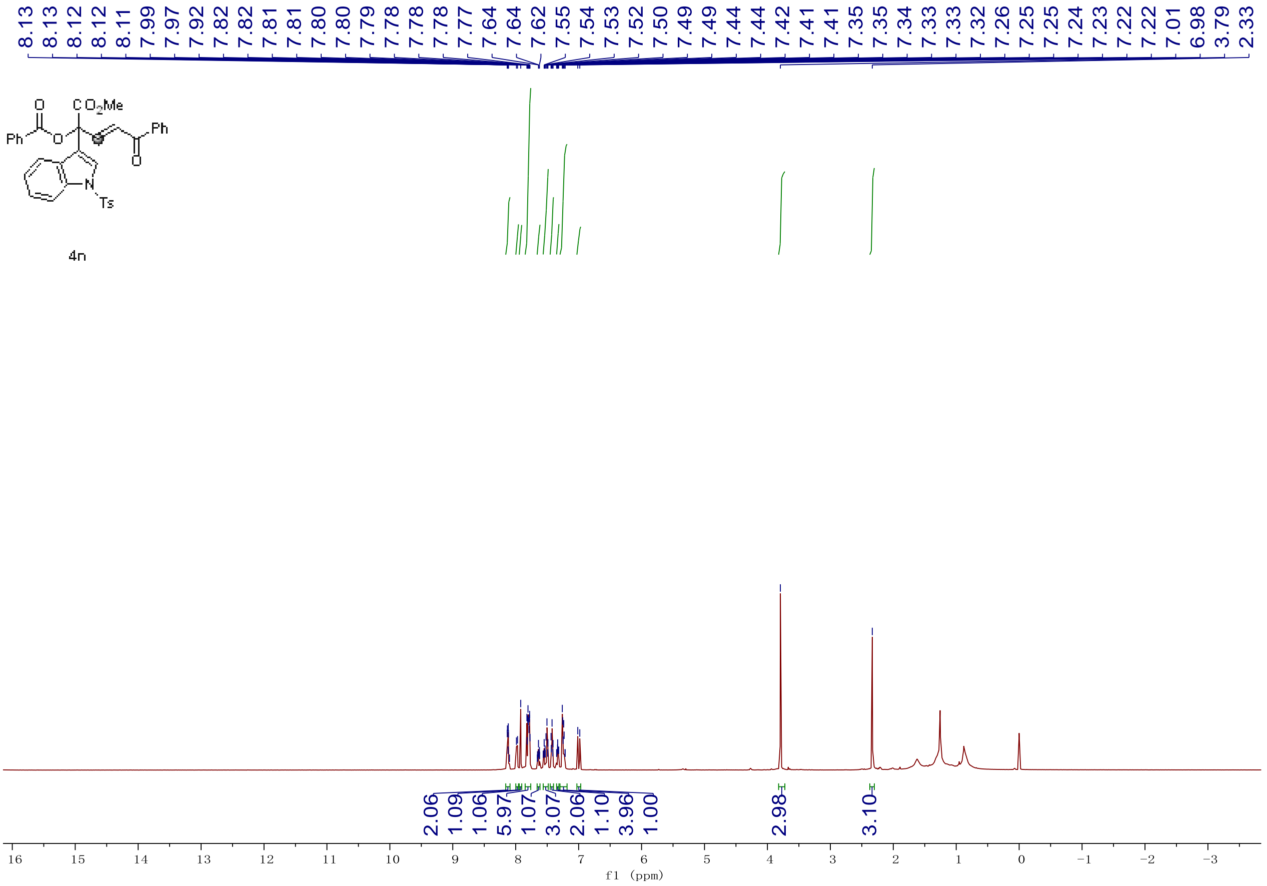

Supplementary Figure 103. ^{1}H NMR spectrum of **4n**.

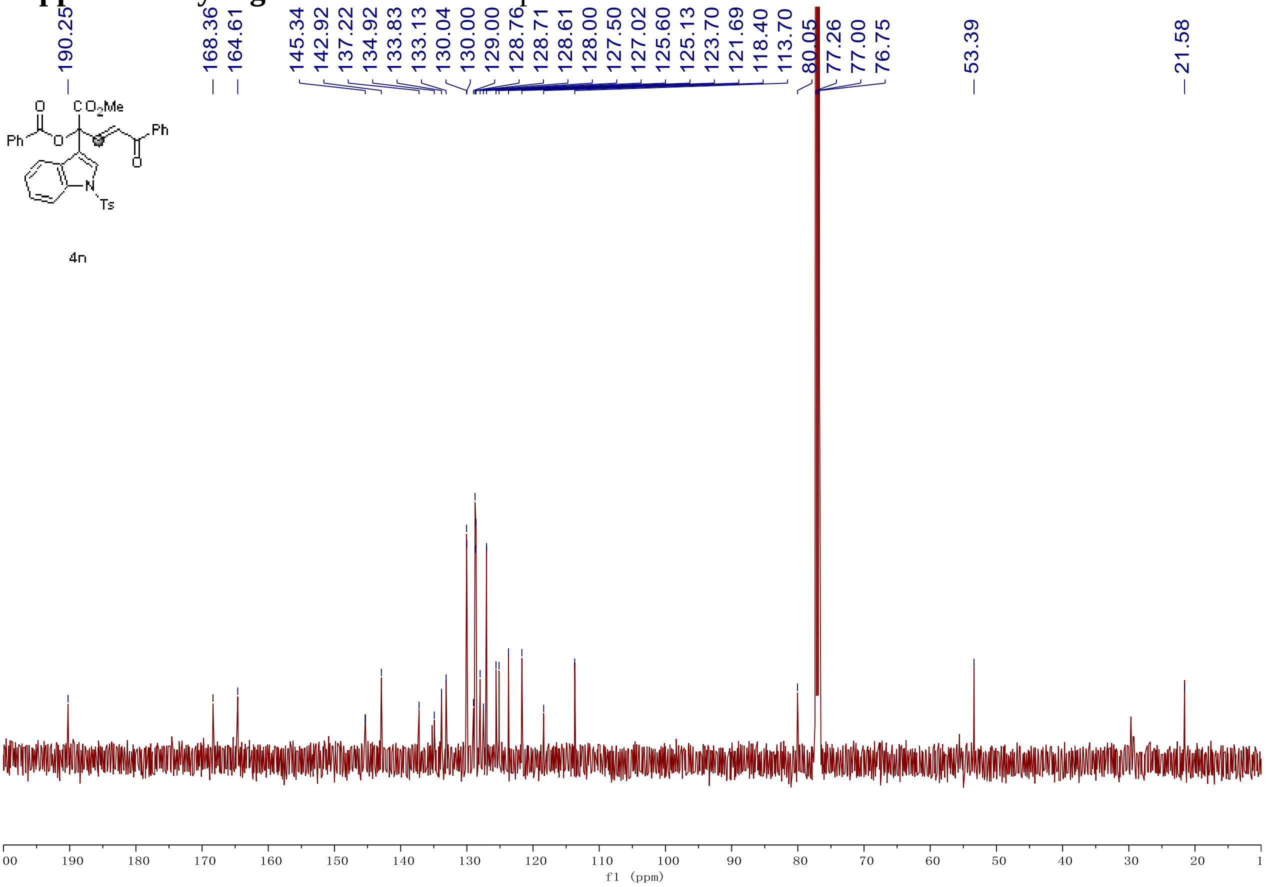

Supplementary Figure 104. ^{13}C NMR spectrum of **4n**.

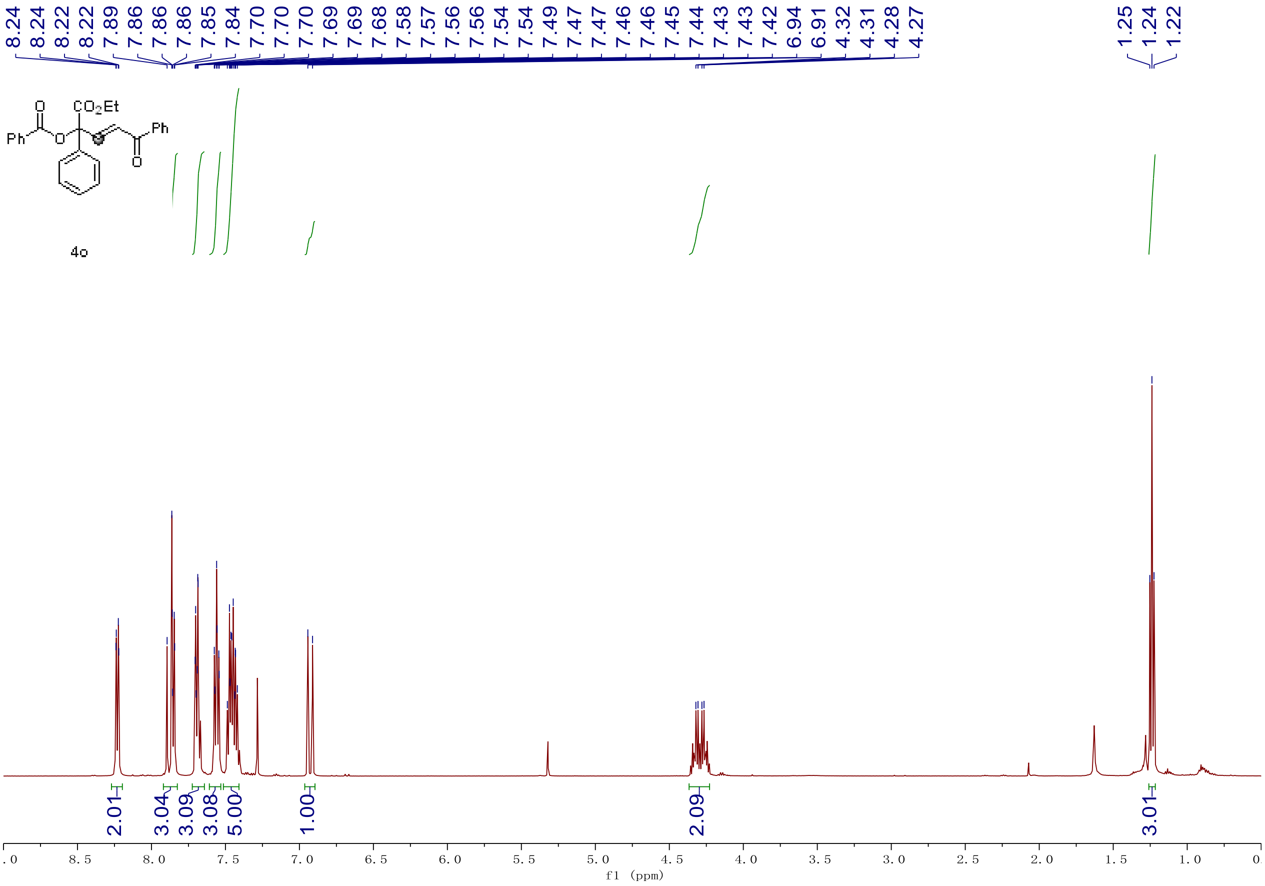

Supplementary Figure 105. ^{1}H NMR spectrum of **4o**.

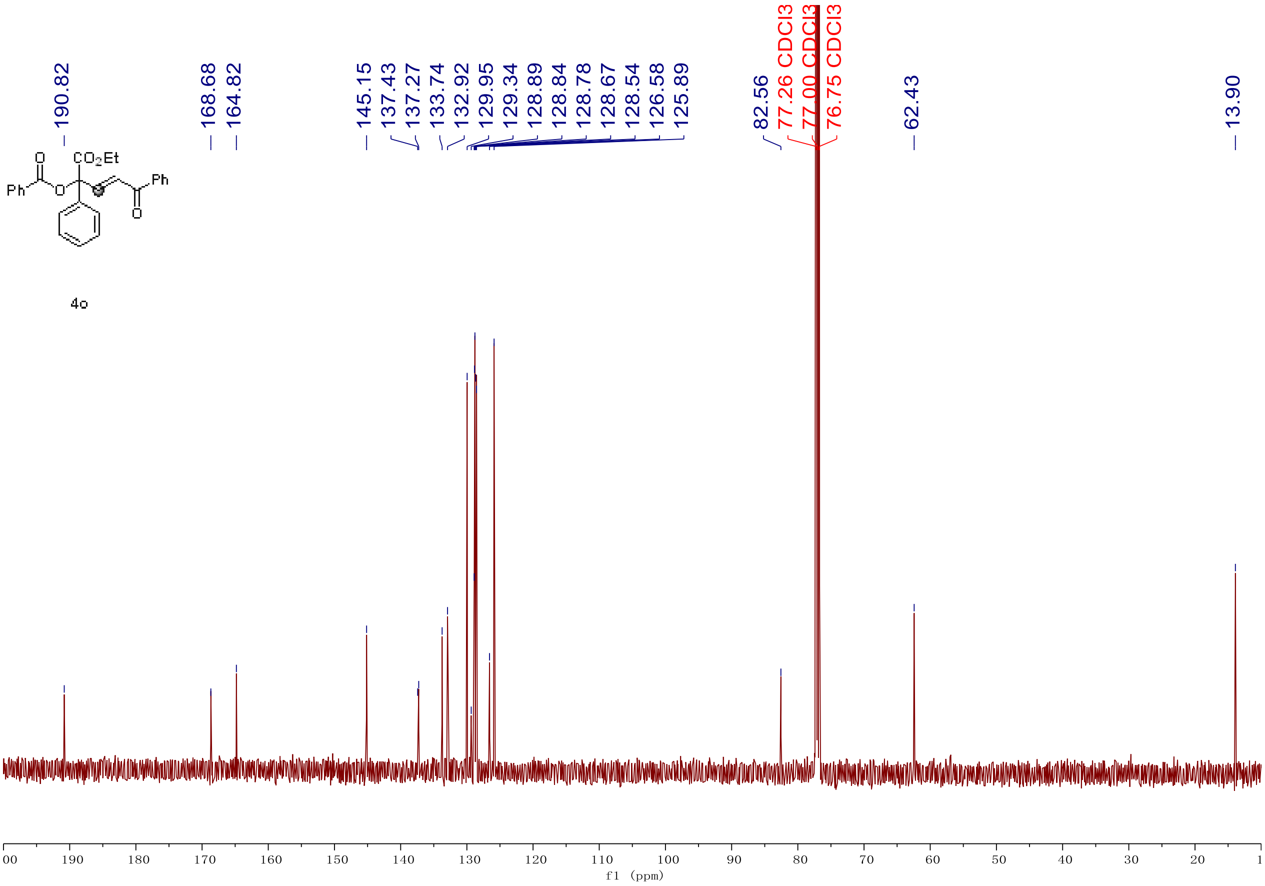

Supplementary Figure 106. ^{13}C NMR spectrum of **4o**.

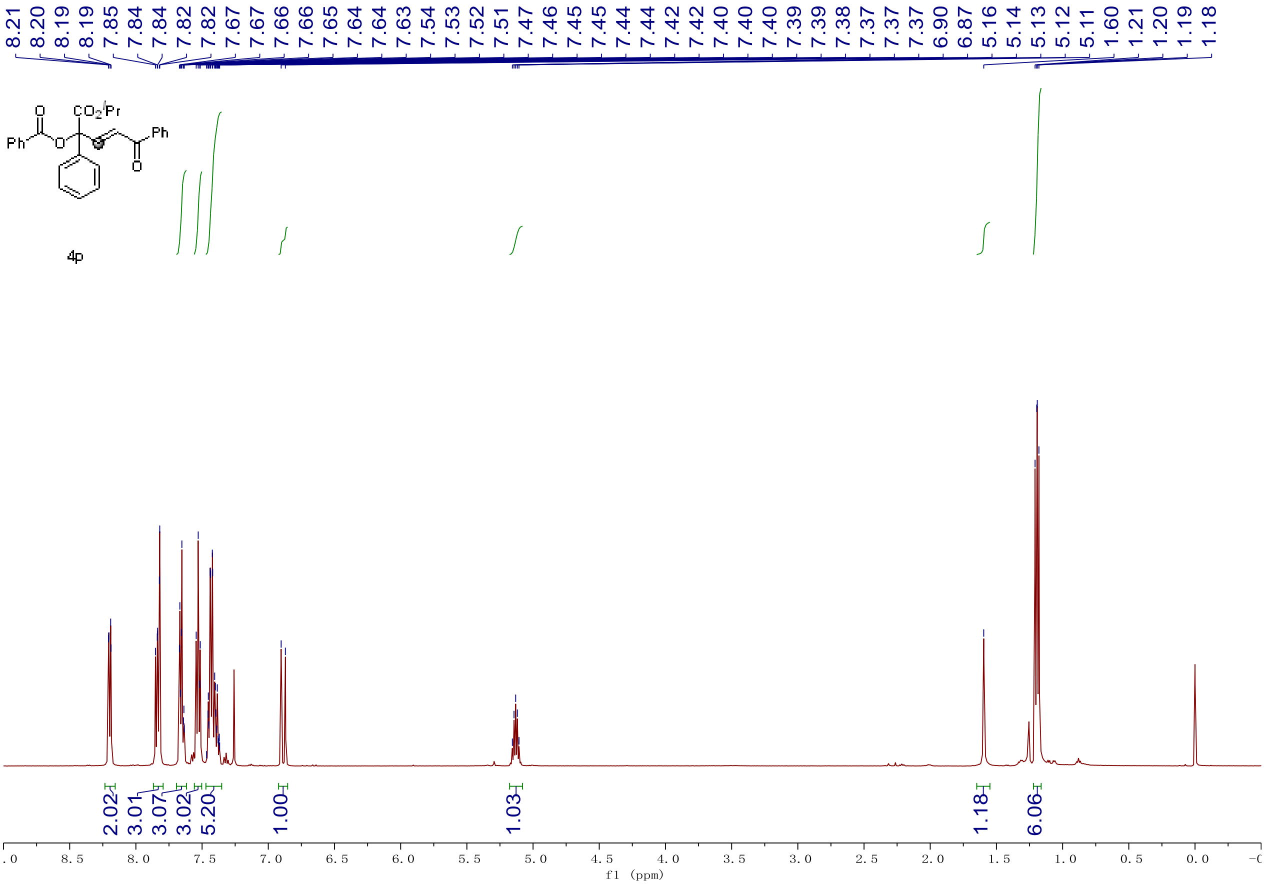

Supplementary Figure 107. ^{1}H NMR spectrum of **4p**.

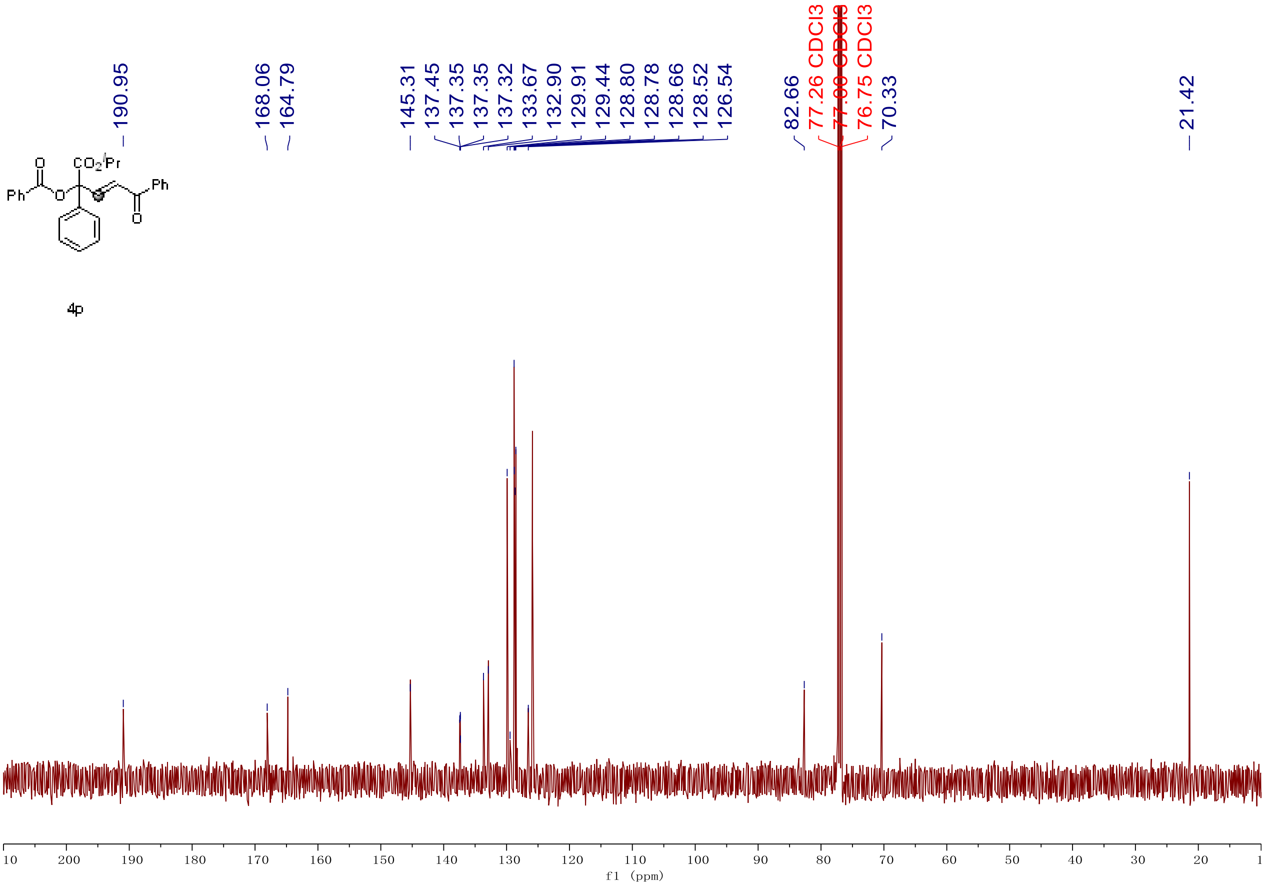

Supplementary Figure 108. ^{13}C NMR spectrum of **4p**.

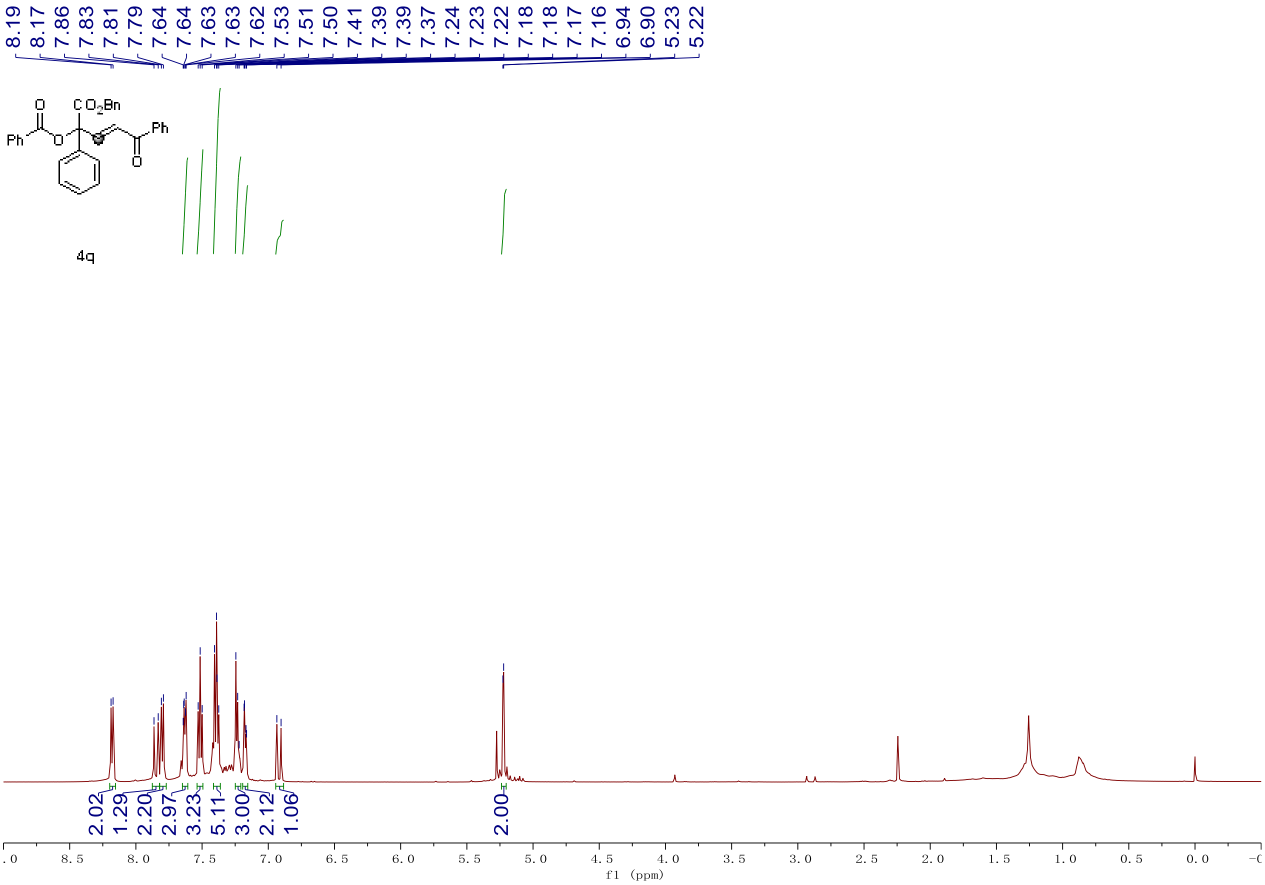

Supplementary Figure 109. ^{1}H NMR spectrum of **4q**.

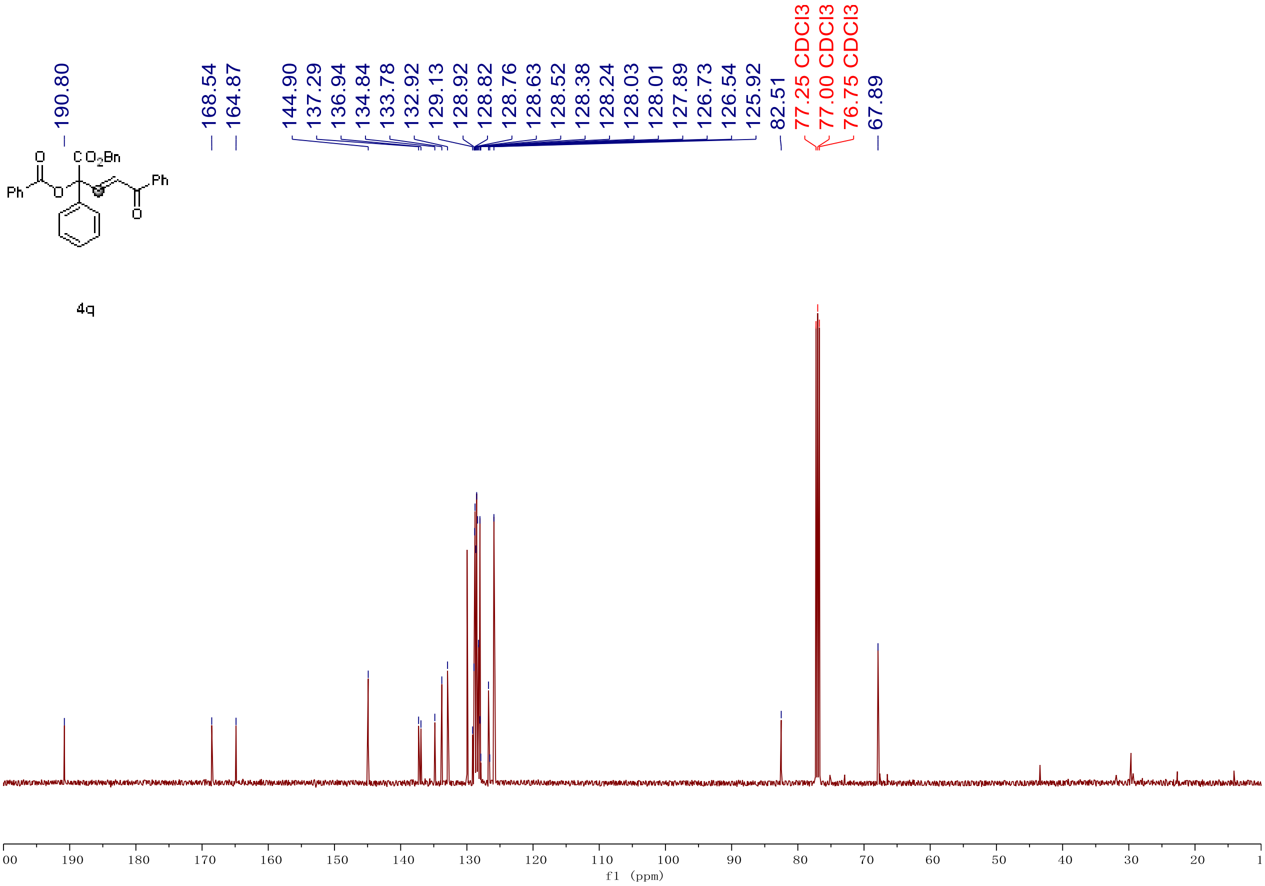

Supplementary Figure 110. ^{13}C NMR spectrum of **4q**.

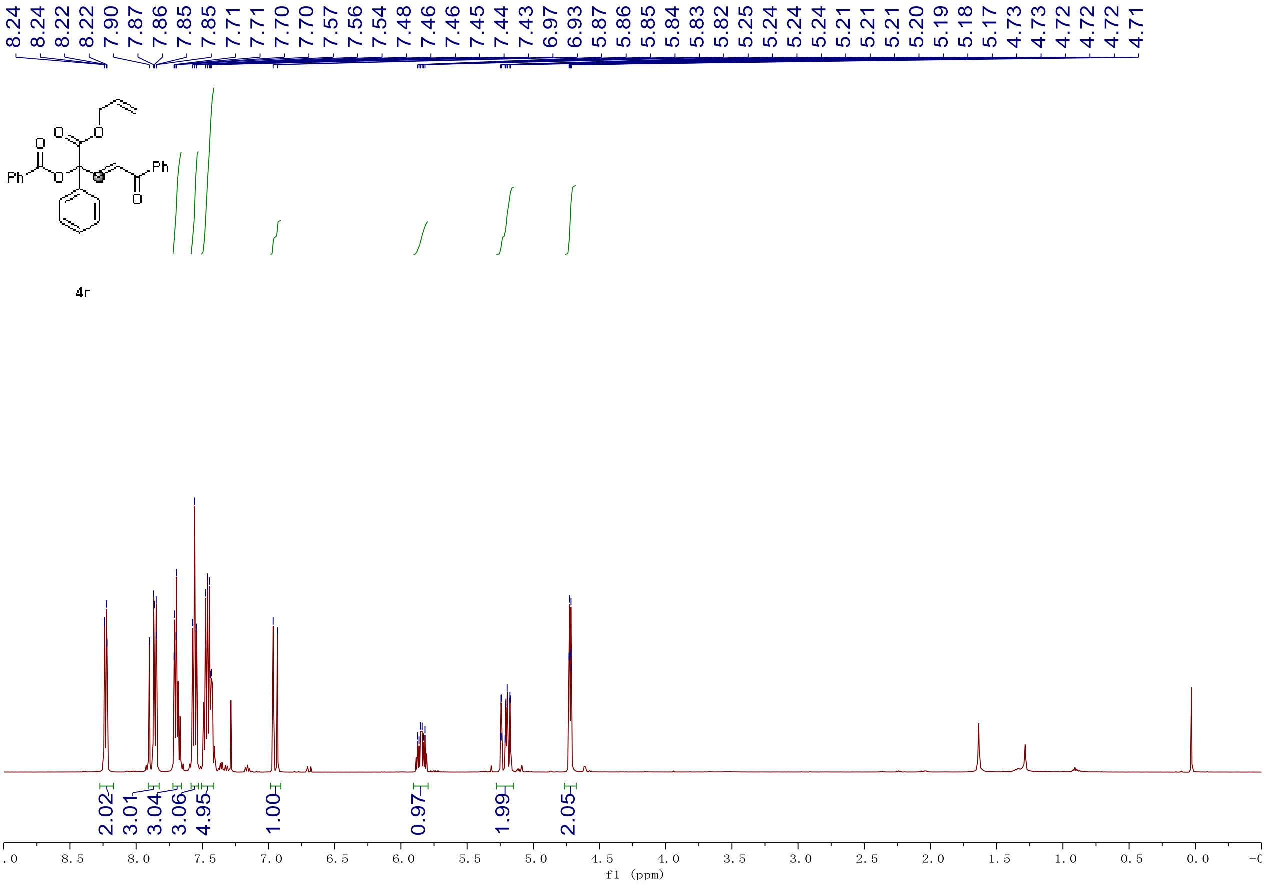

Supplementary Figure 111. ¹H NMR spectrum of **4r**.

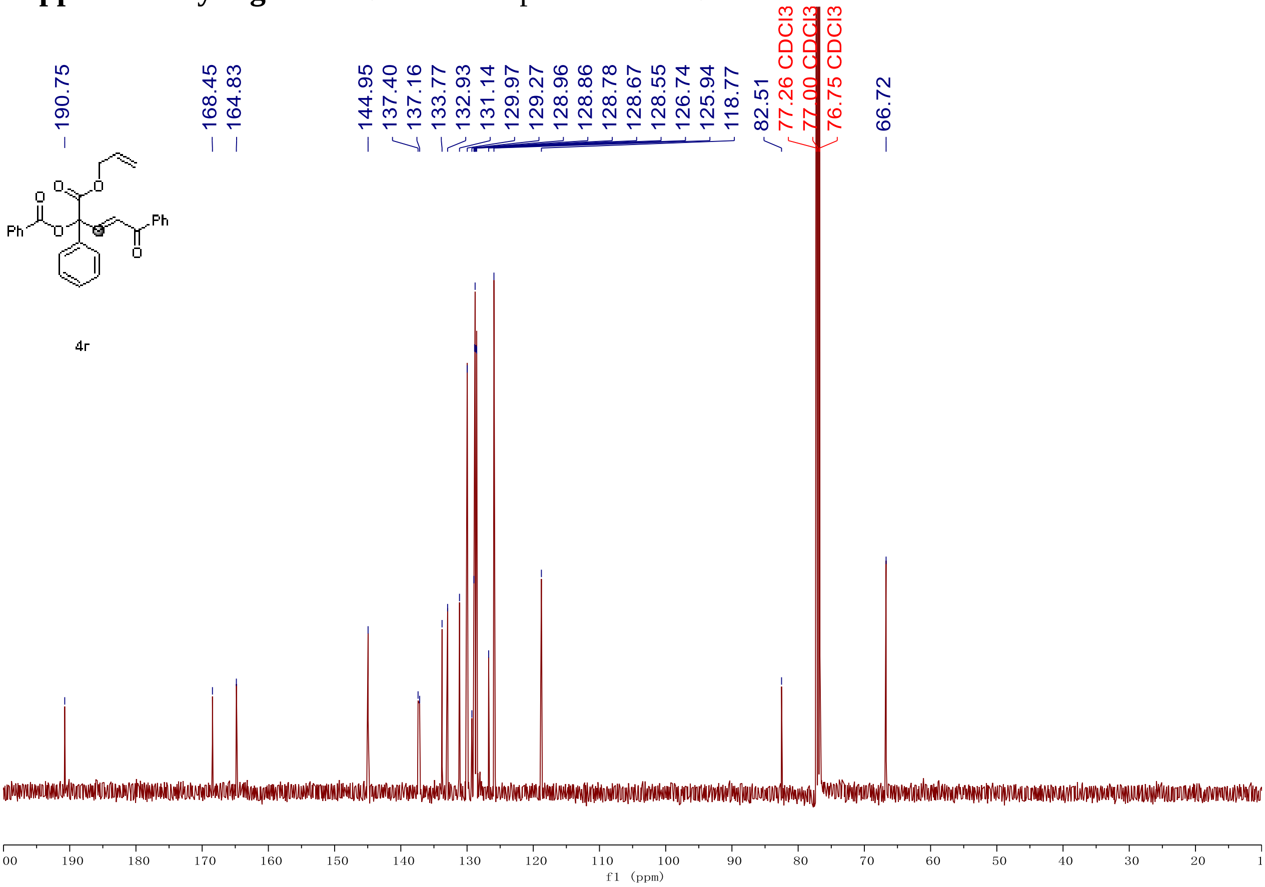

Supplementary Figure 112. ¹³C NMR spectrum of **4r**.

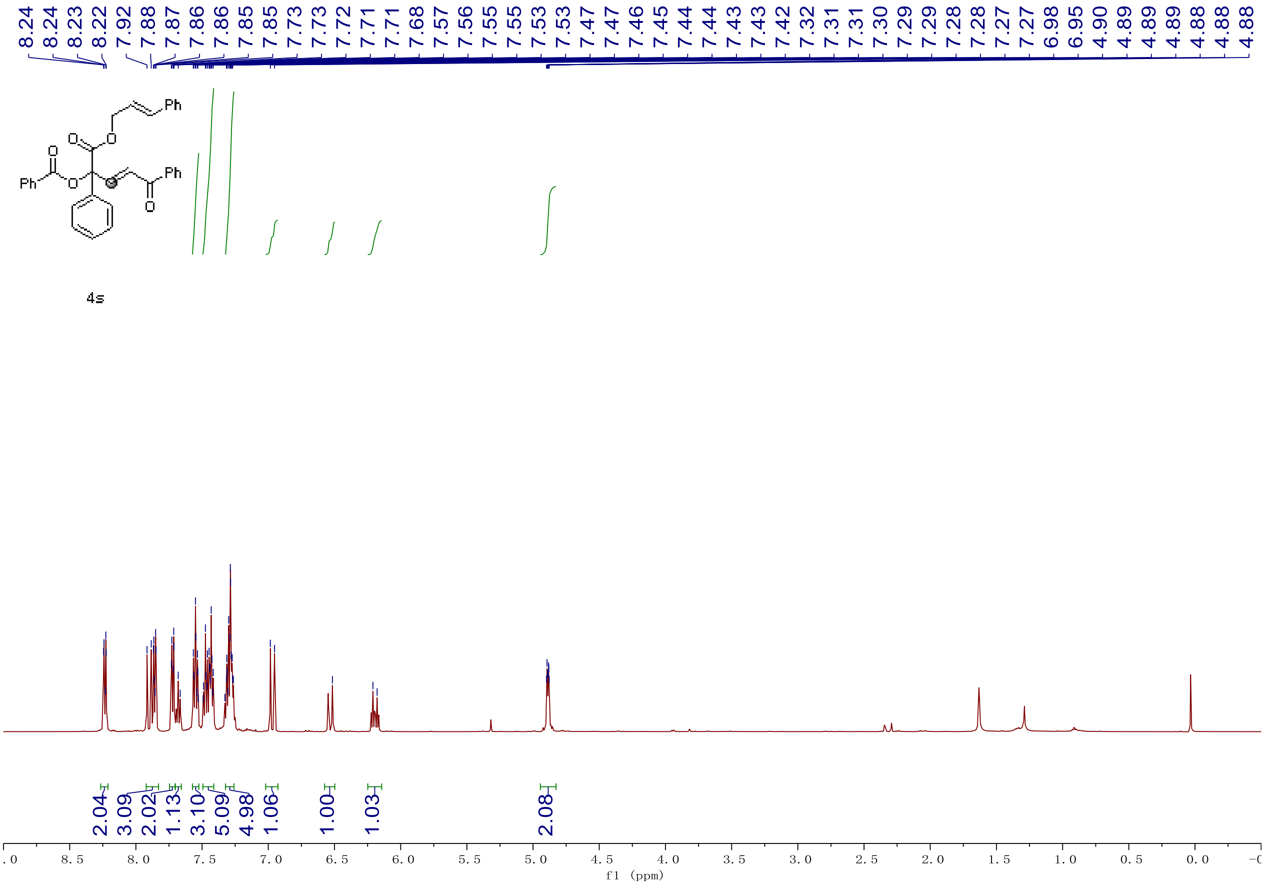

Supplementary Figure 113. ^{1}H NMR spectrum of **4s**.

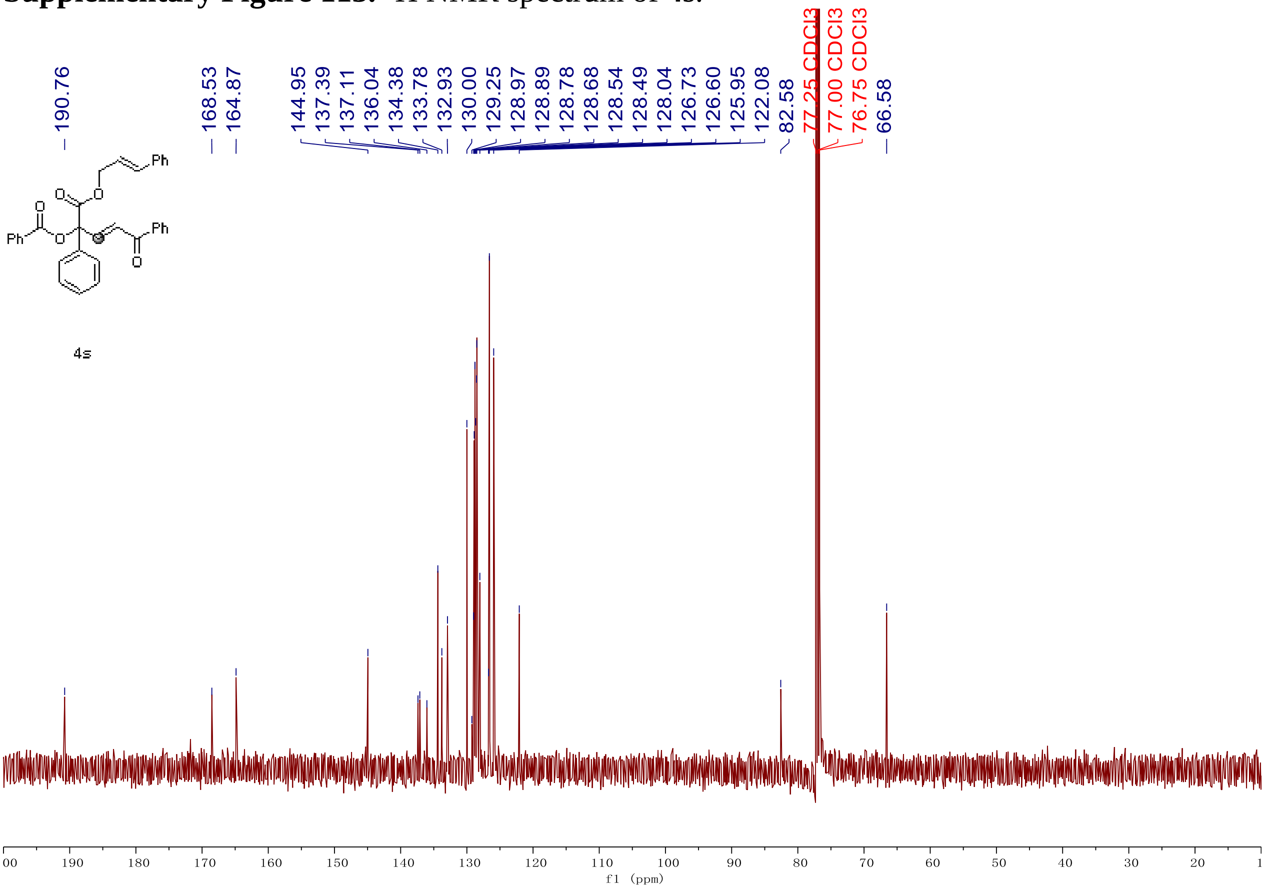

Supplementary Figure 114. ^{13}C NMR spectrum of **4s**.

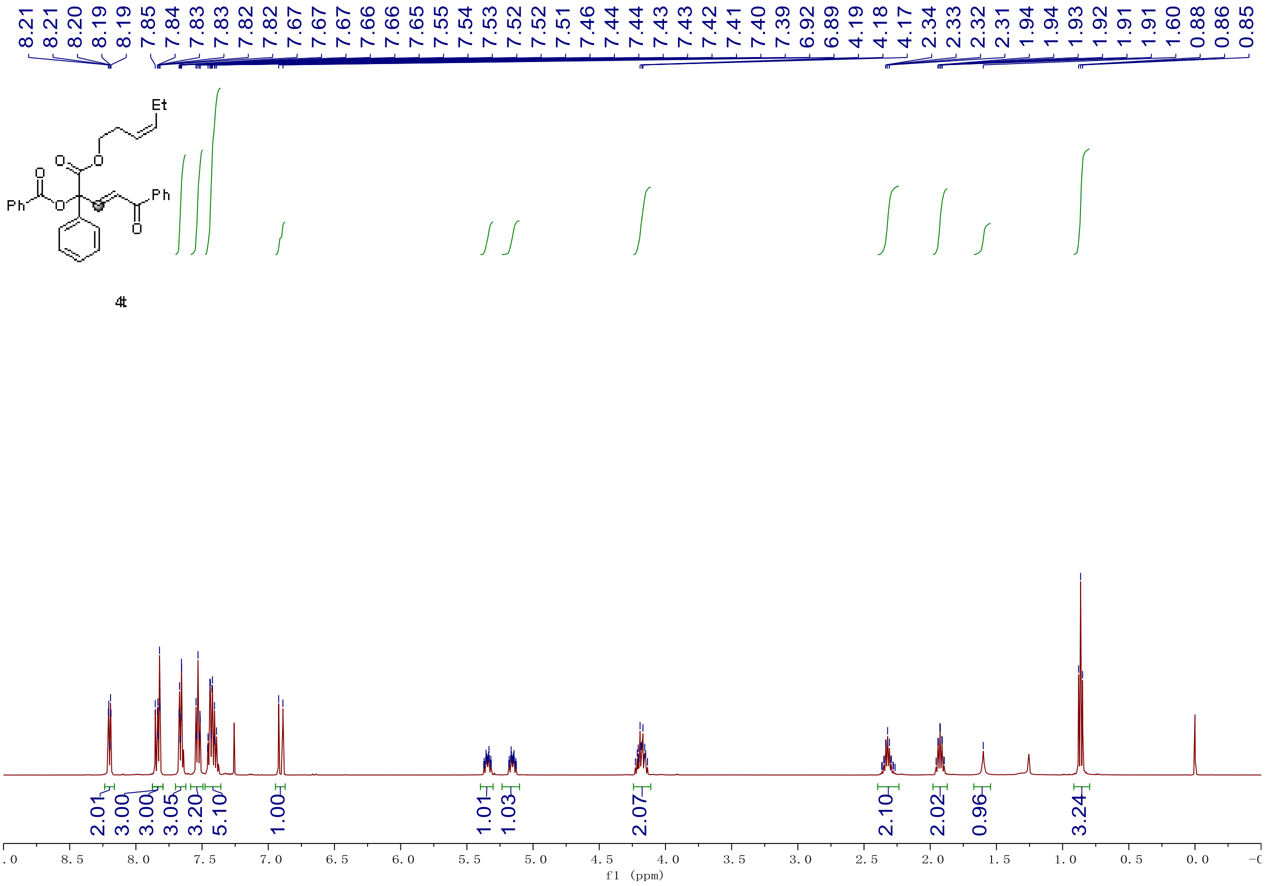

Supplementary Figure 115. ^{1}H NMR spectrum of **4t**.

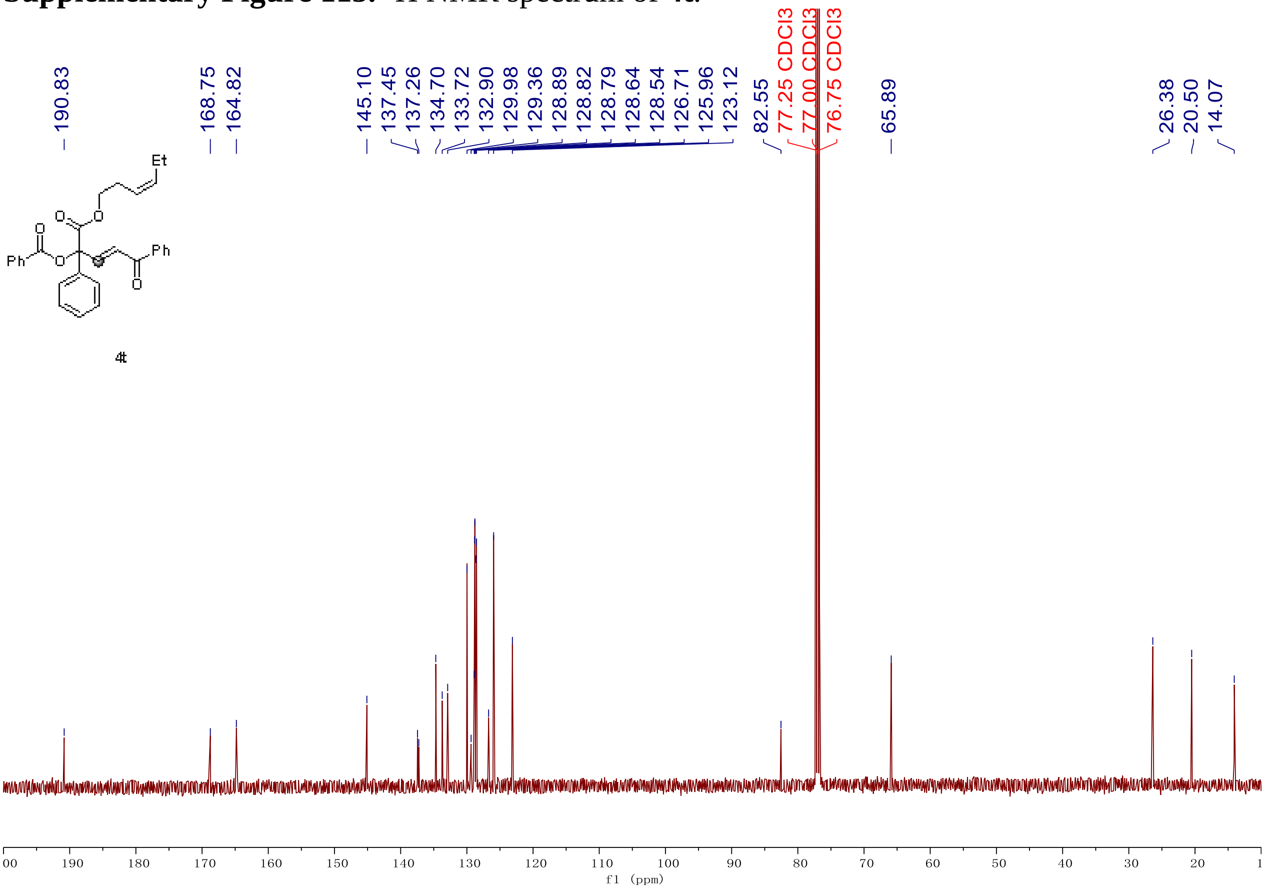

Supplementary Figure 116. ^{13}C NMR spectrum of **4t**.

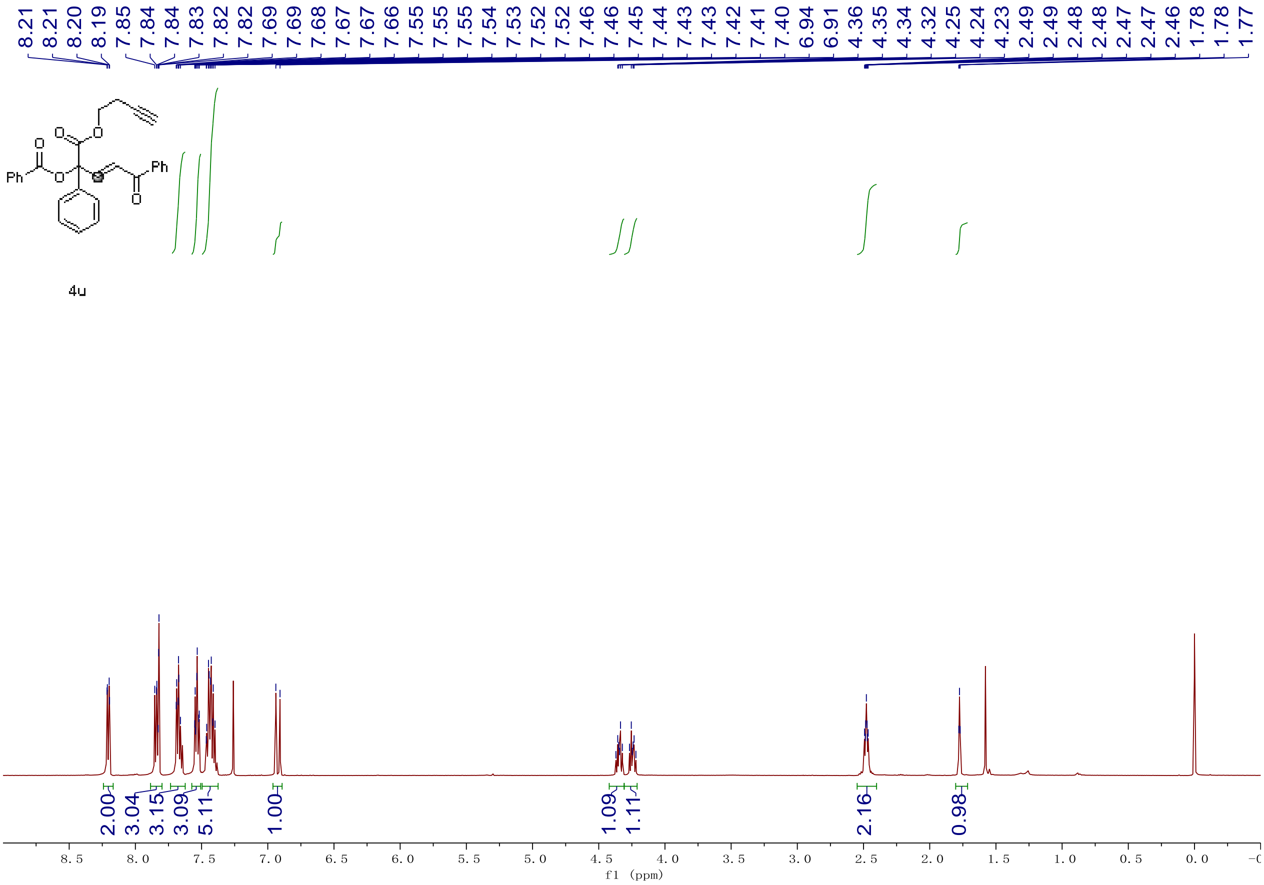

Supplementary Figure 117. ^{1}H NMR spectrum of **4u**.

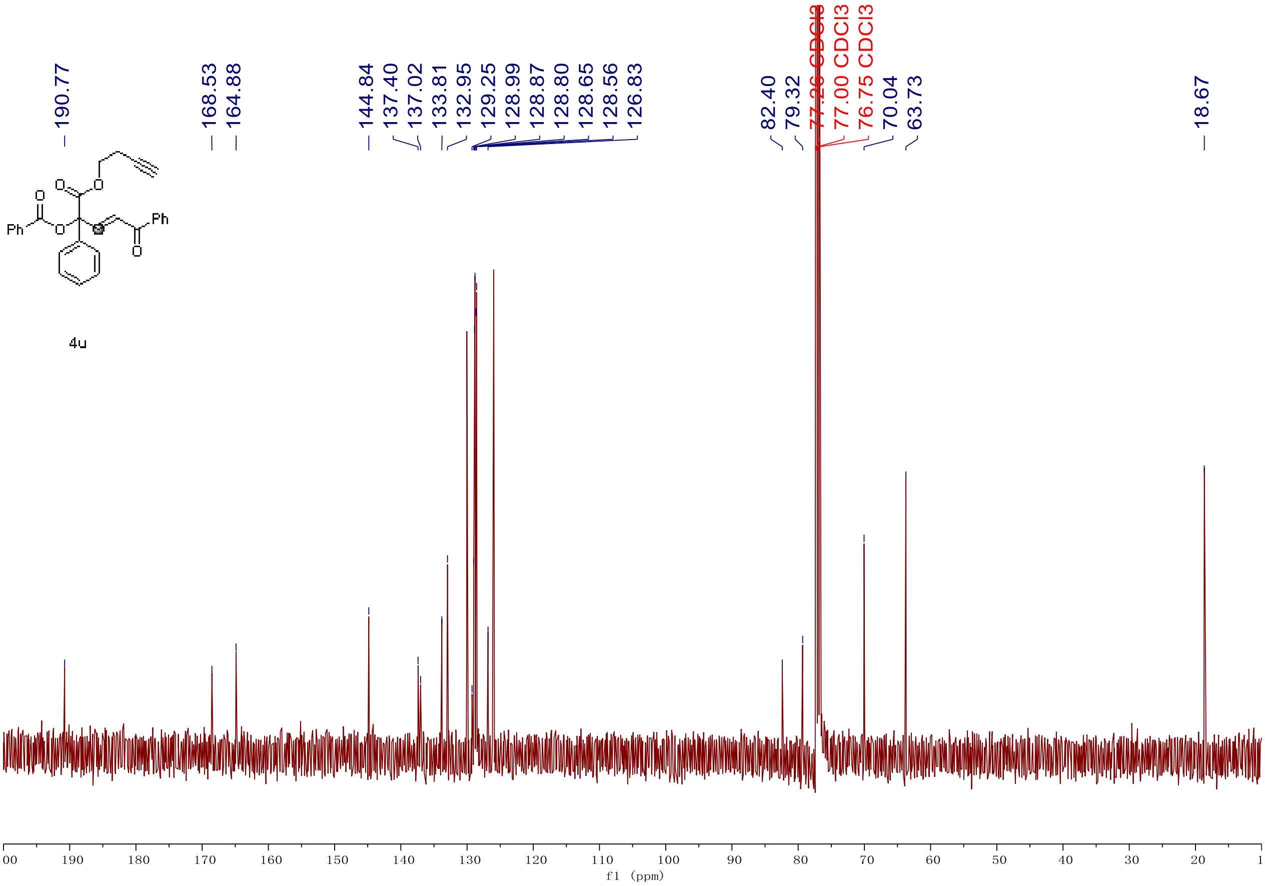

Supplementary Figure 118. ^{13}C NMR spectrum of **4u**.

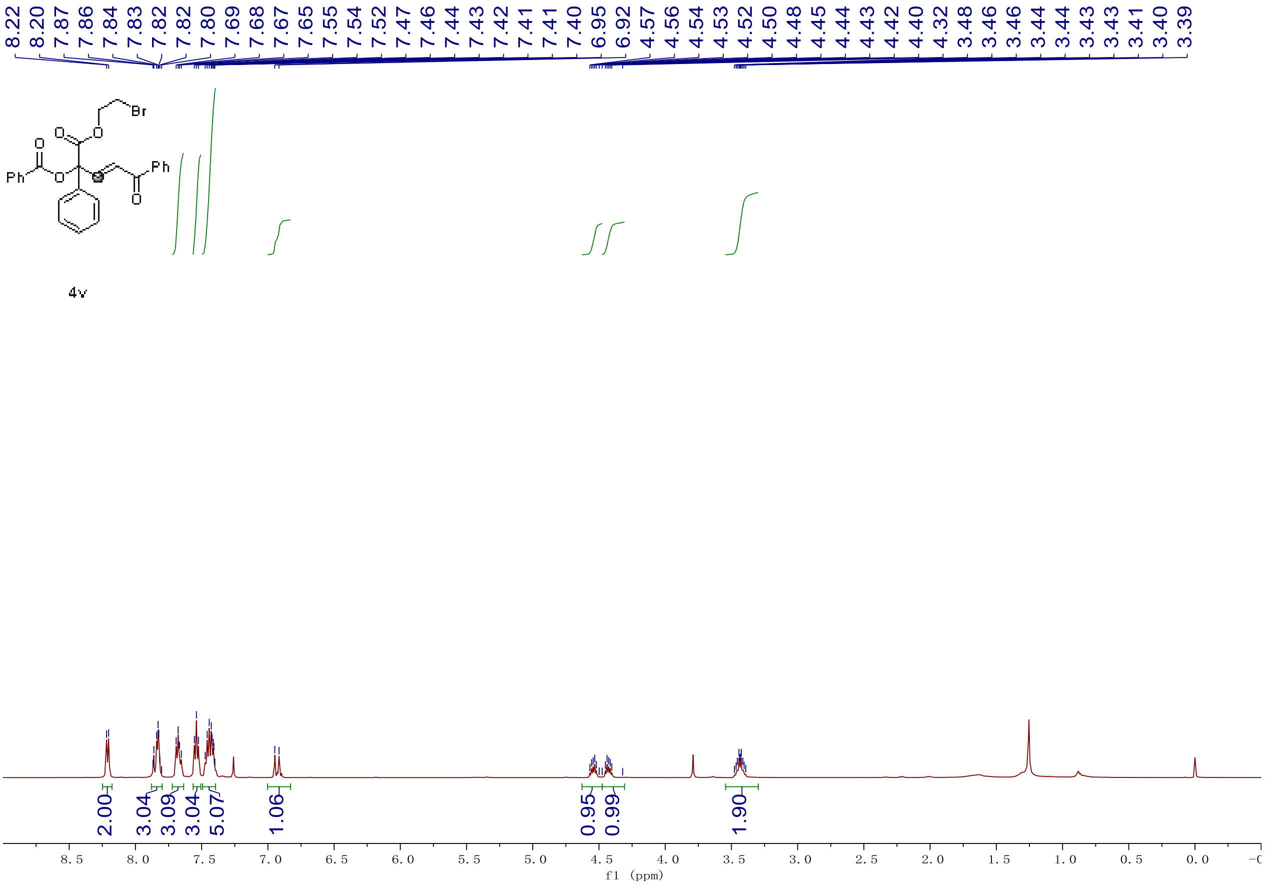

Supplementary Figure 119. ^{1}H NMR spectrum of **4v**.

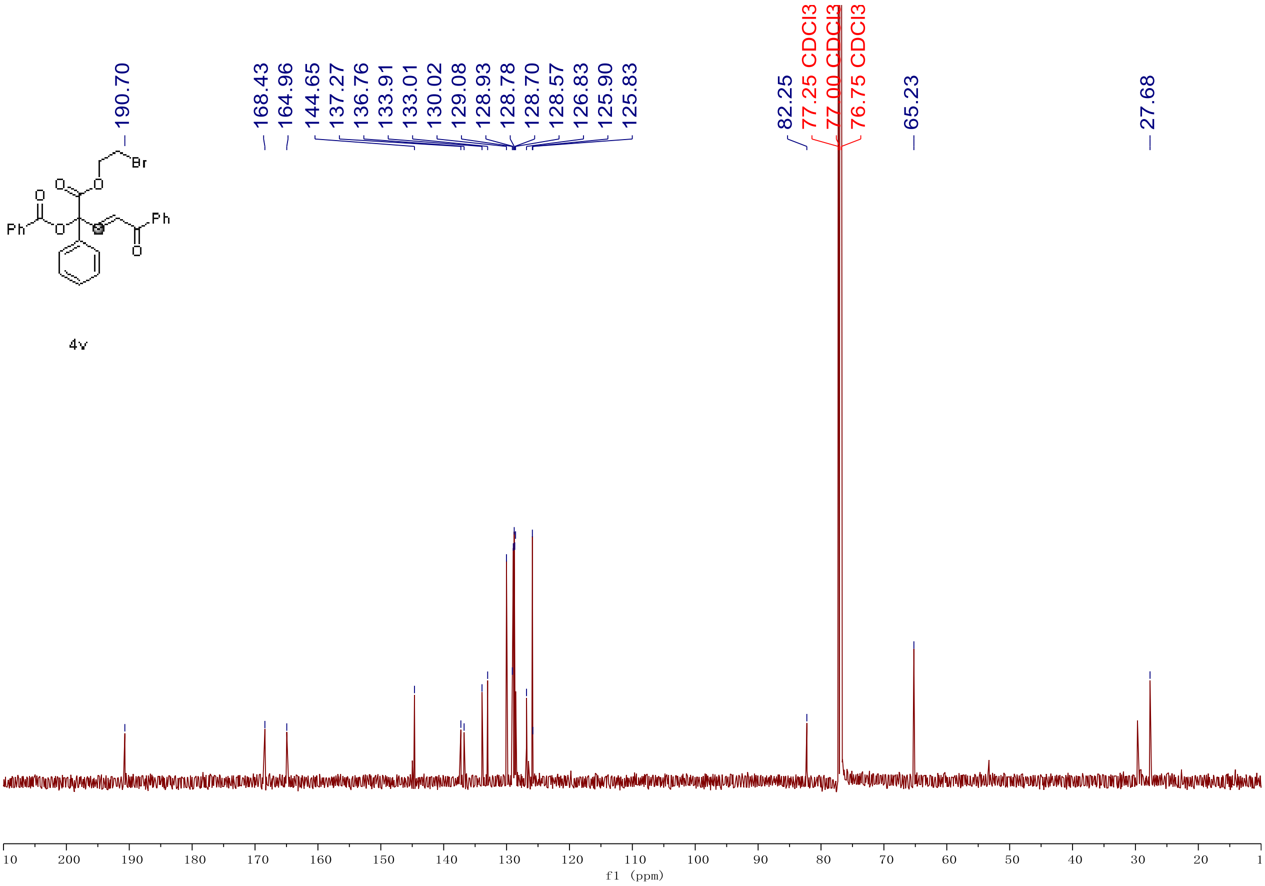

Supplementary Figure 120. ^{13}C NMR spectrum of **4v**.

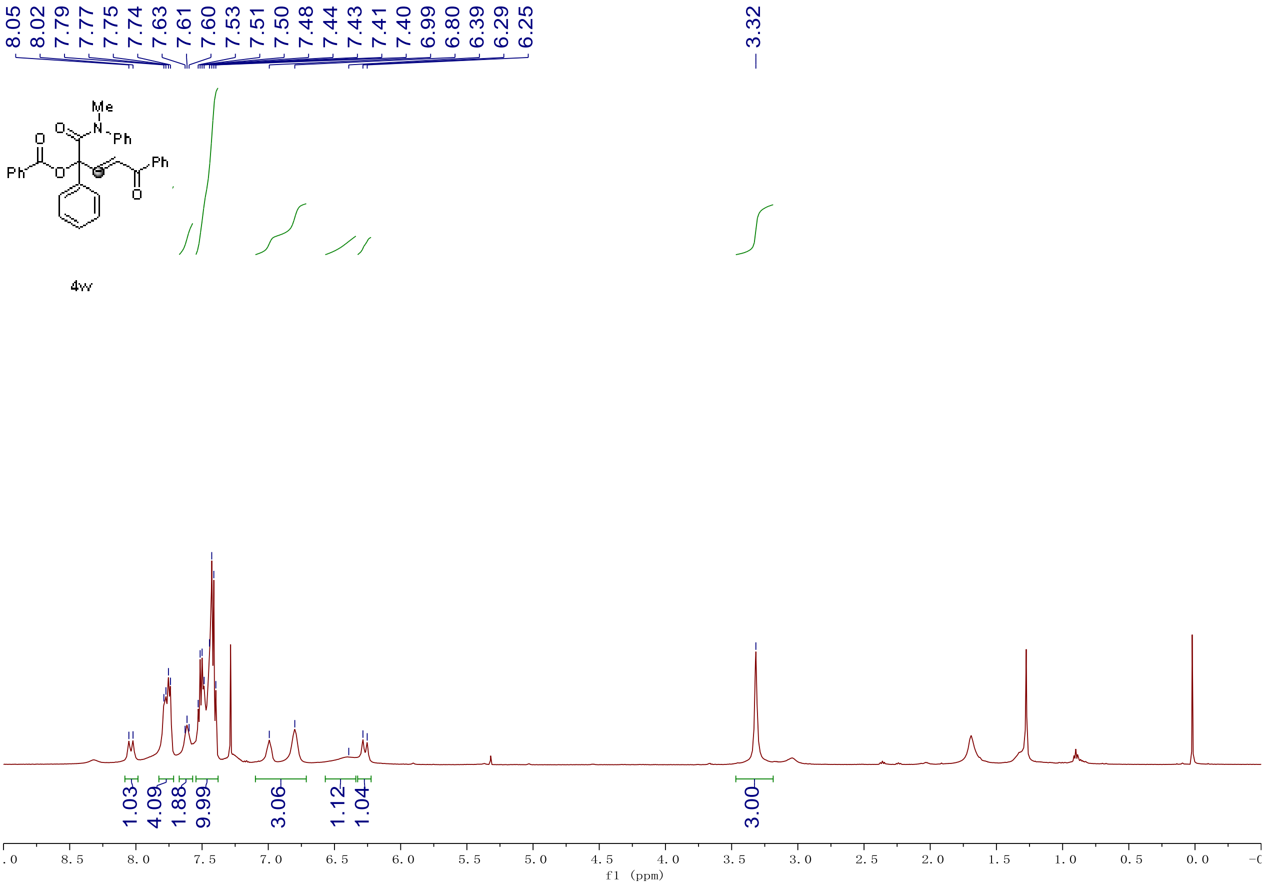

Supplementary Figure 121. ^{1}H NMR spectrum of **4w**.

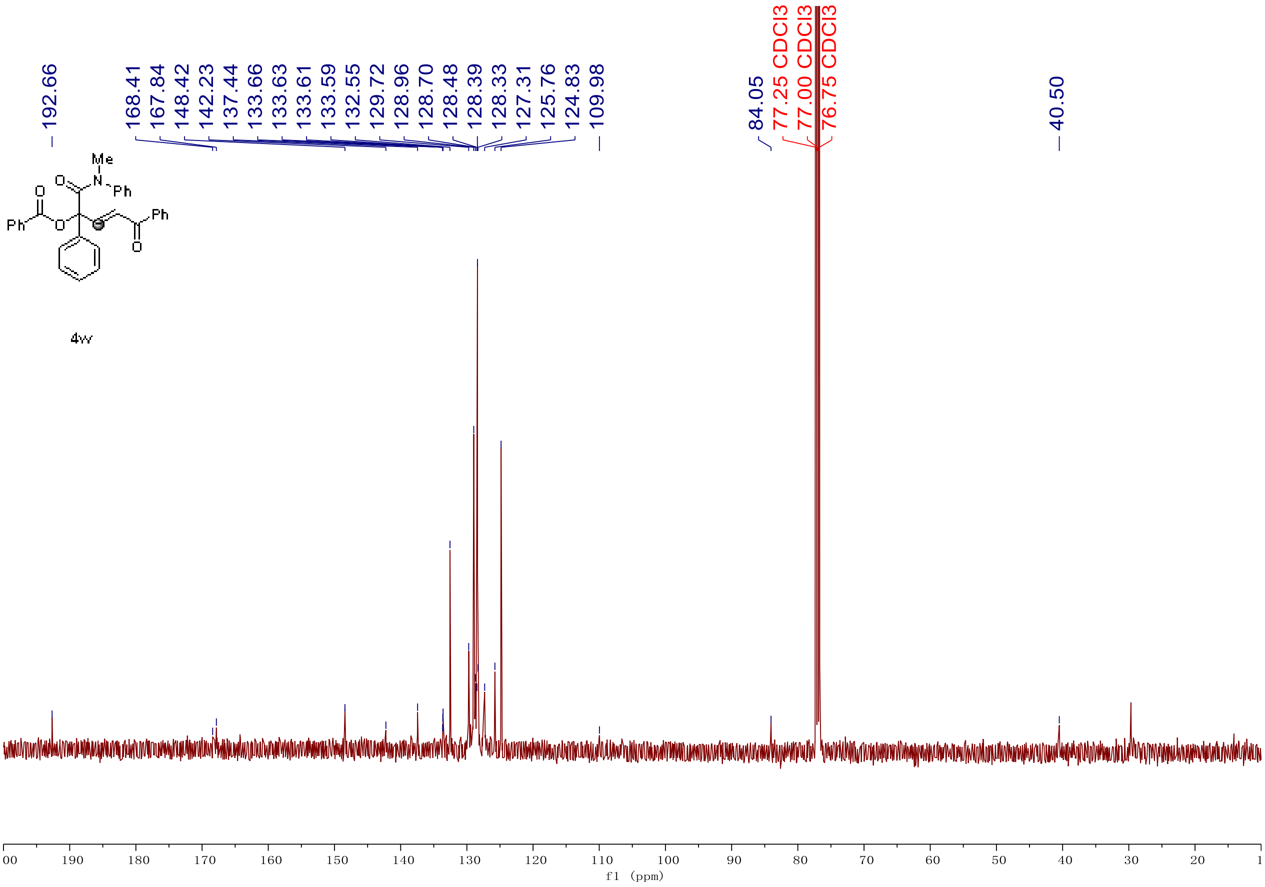

Supplementary Figure 122. ^{13}C NMR spectrum of **4w**.

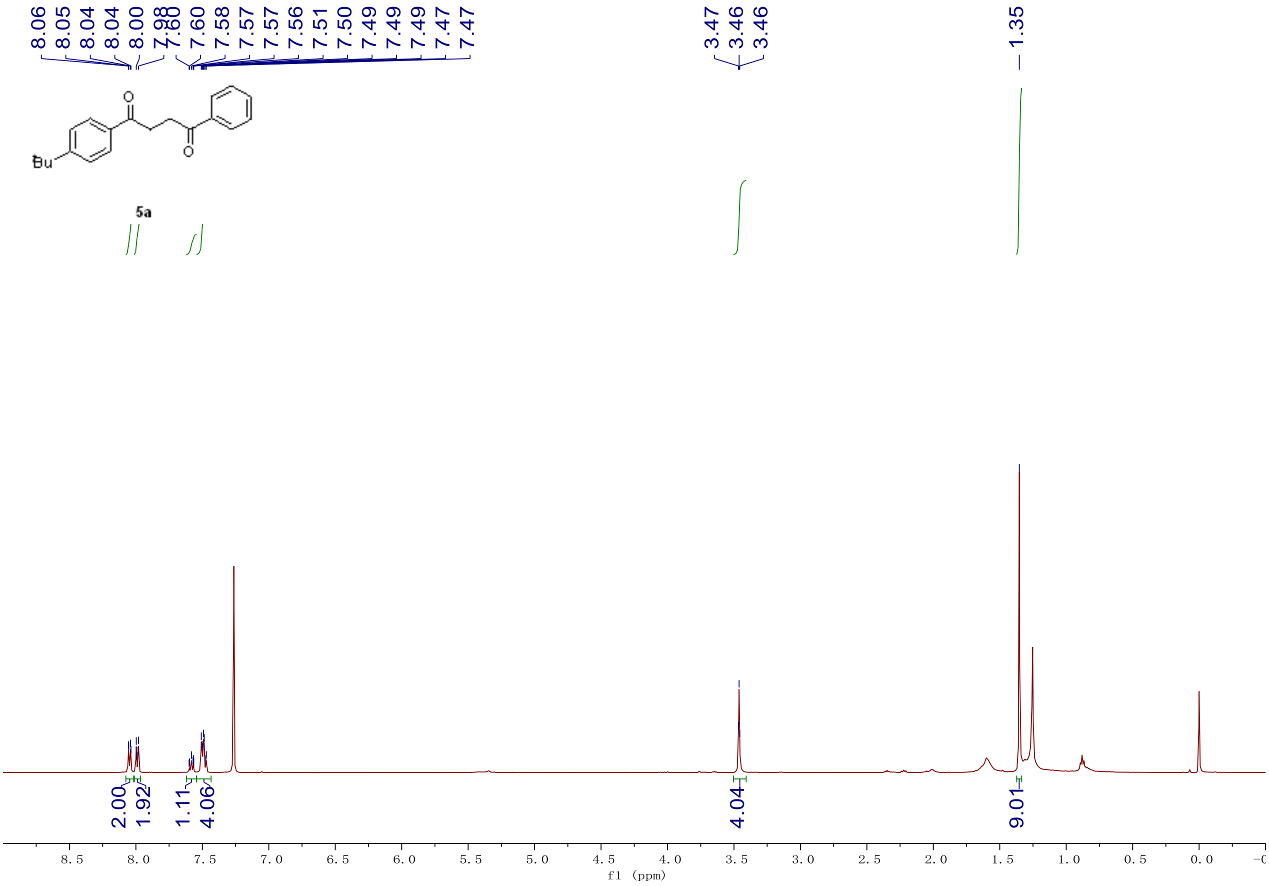

Supplementary Figure 123. 1H NMR spectrum of **5a.**

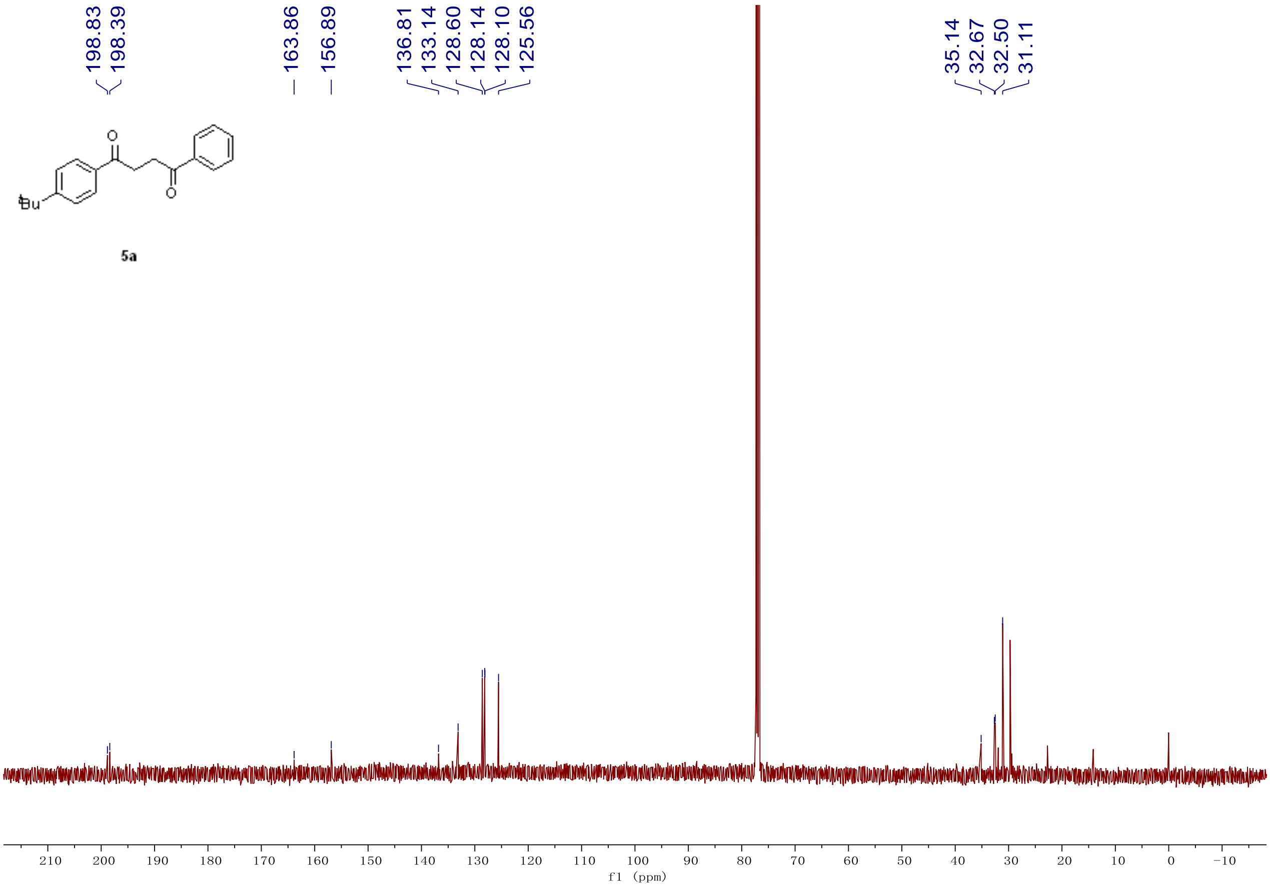

Supplementary Figure 124. ^{13}C NMR spectrum of **5a**.

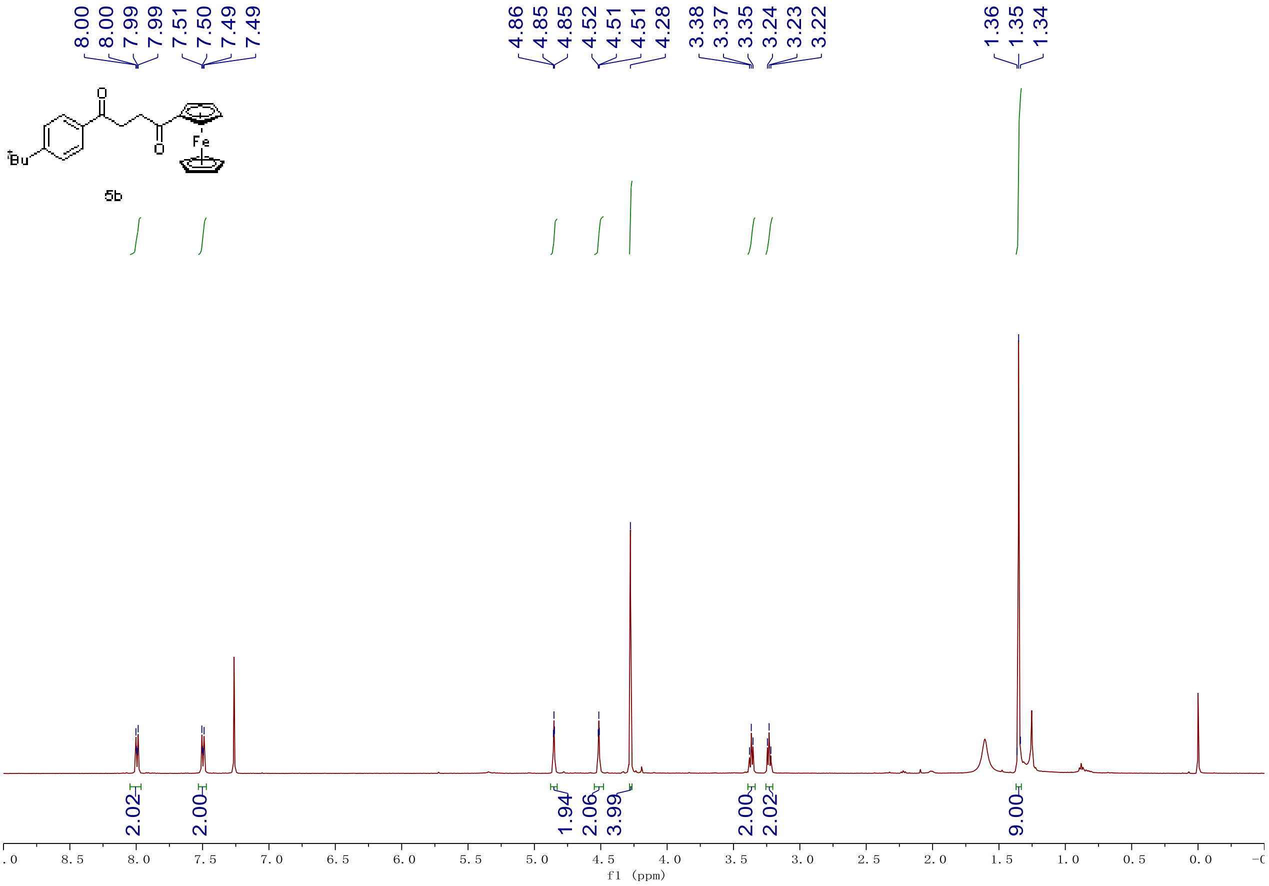

Supplementary Figure 125. ^{1}H NMR spectrum of **5b**.

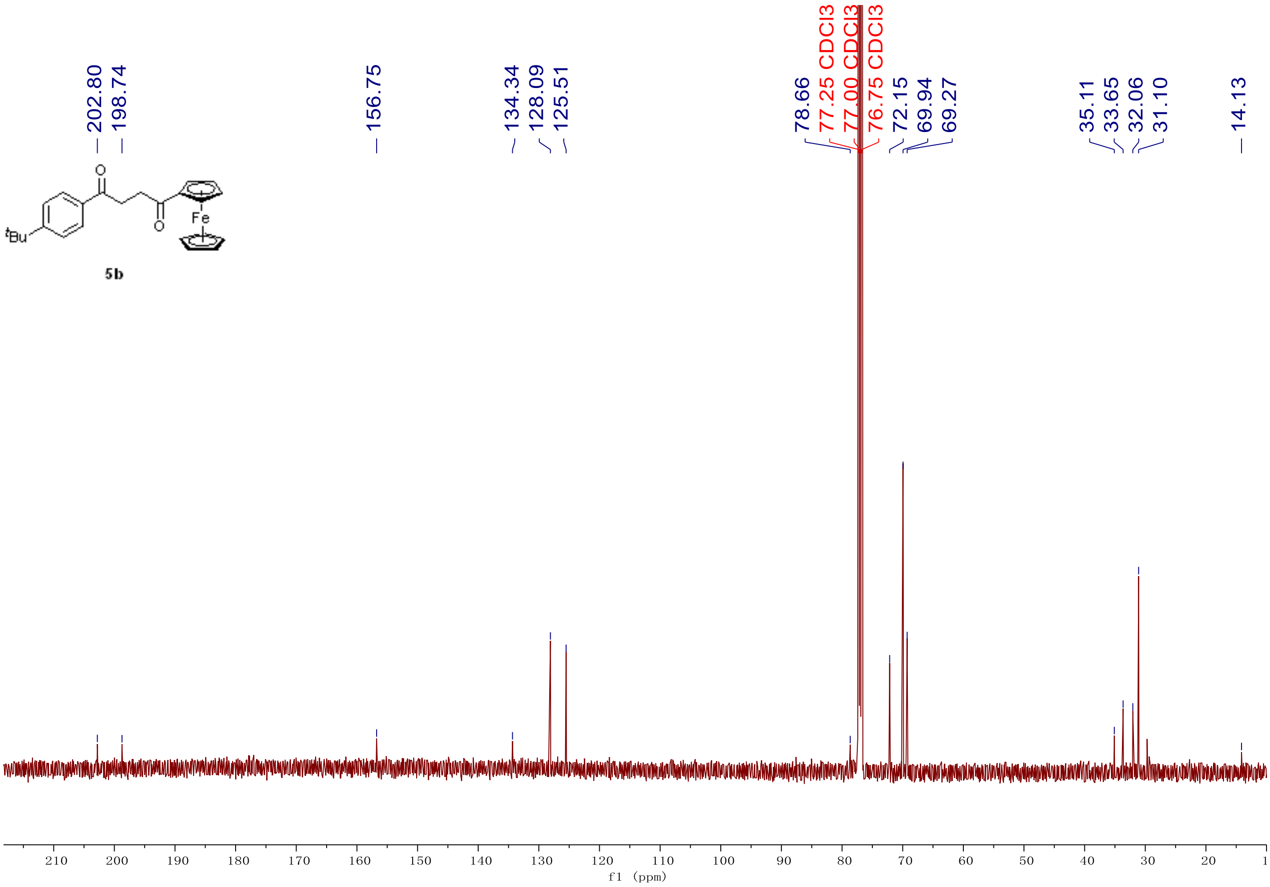

Supplementary Figure 126. ^{13}C NMR spectrum of **5b**.

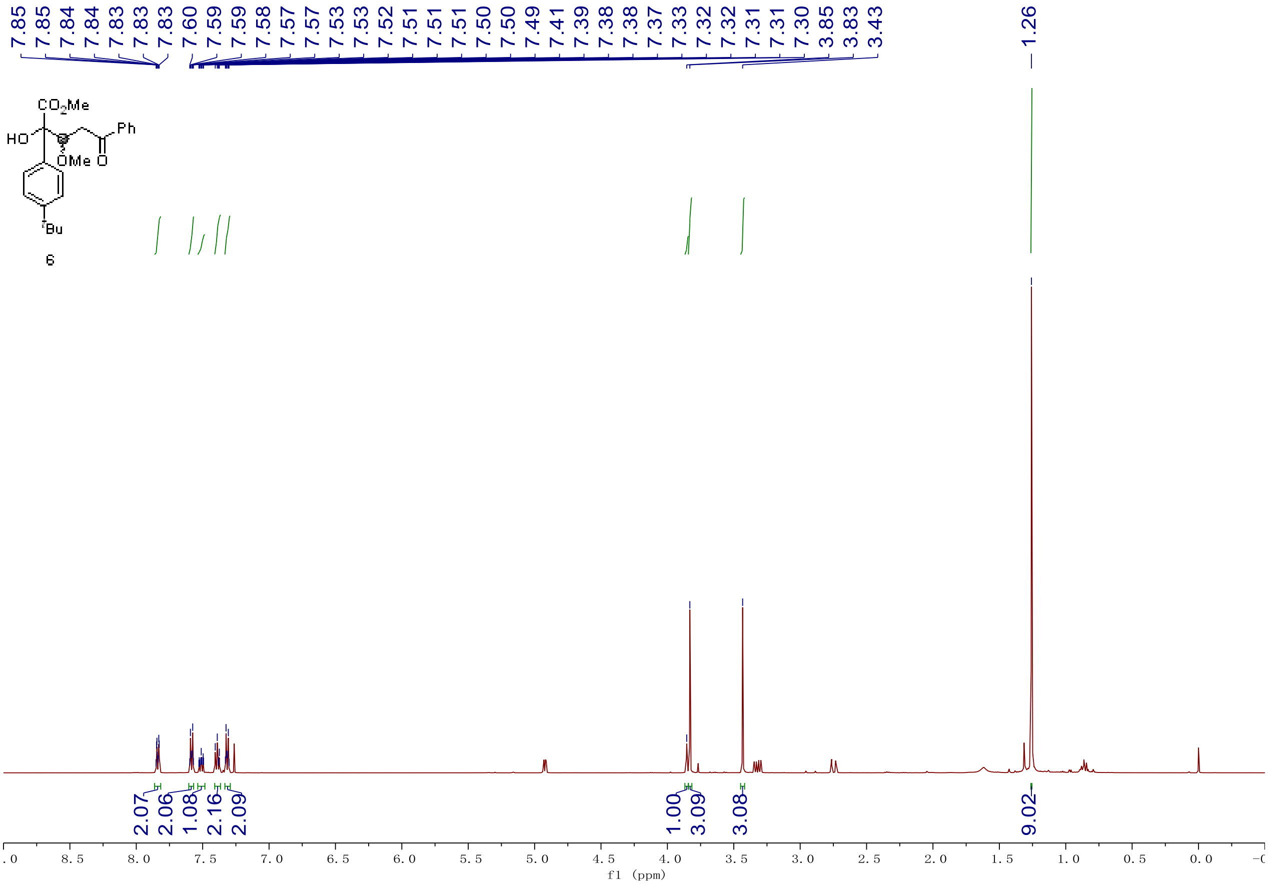

Supplementary Figure 127. ^{1}H NMR spectrum of **6**.

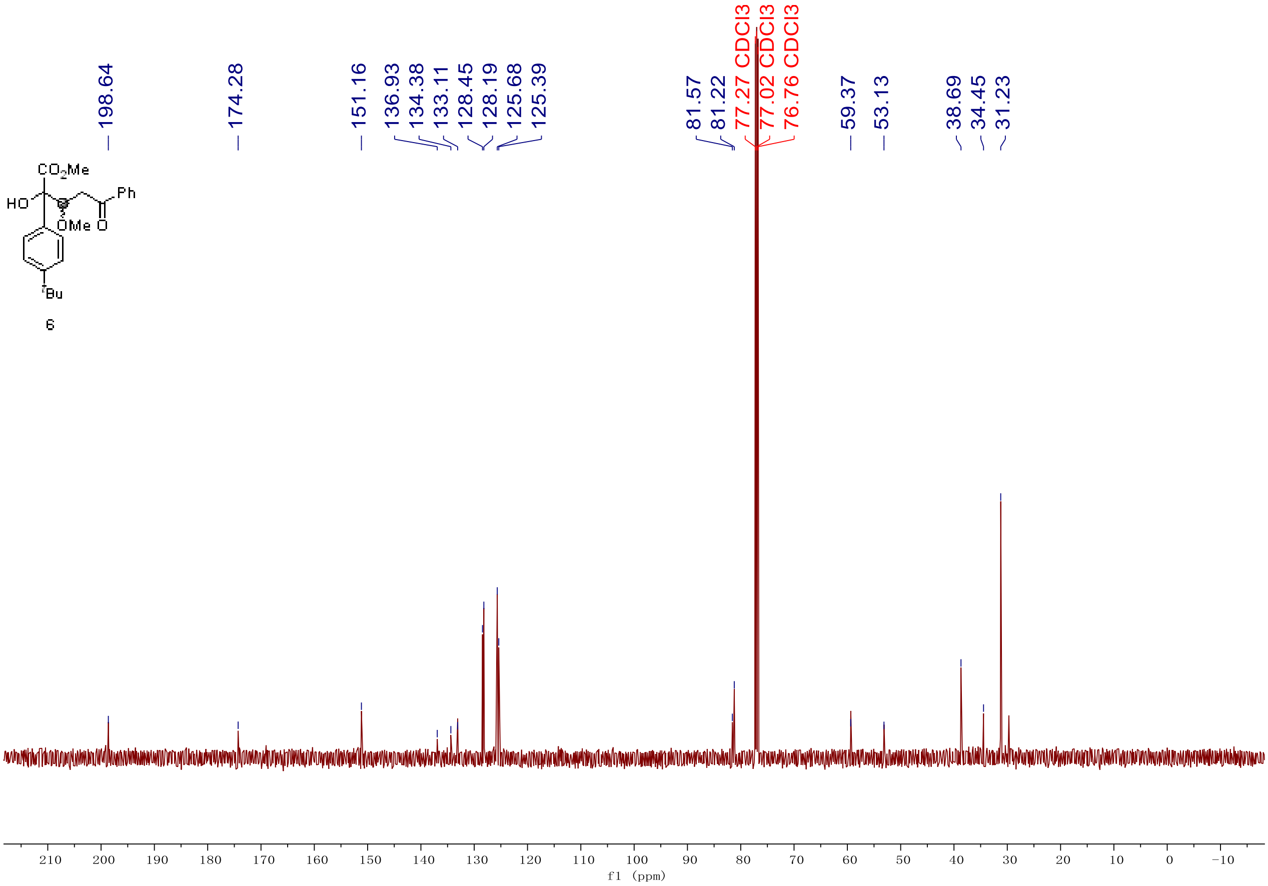

Supplementary Figure 128. ^{13}C NMR spectrum of **6**.

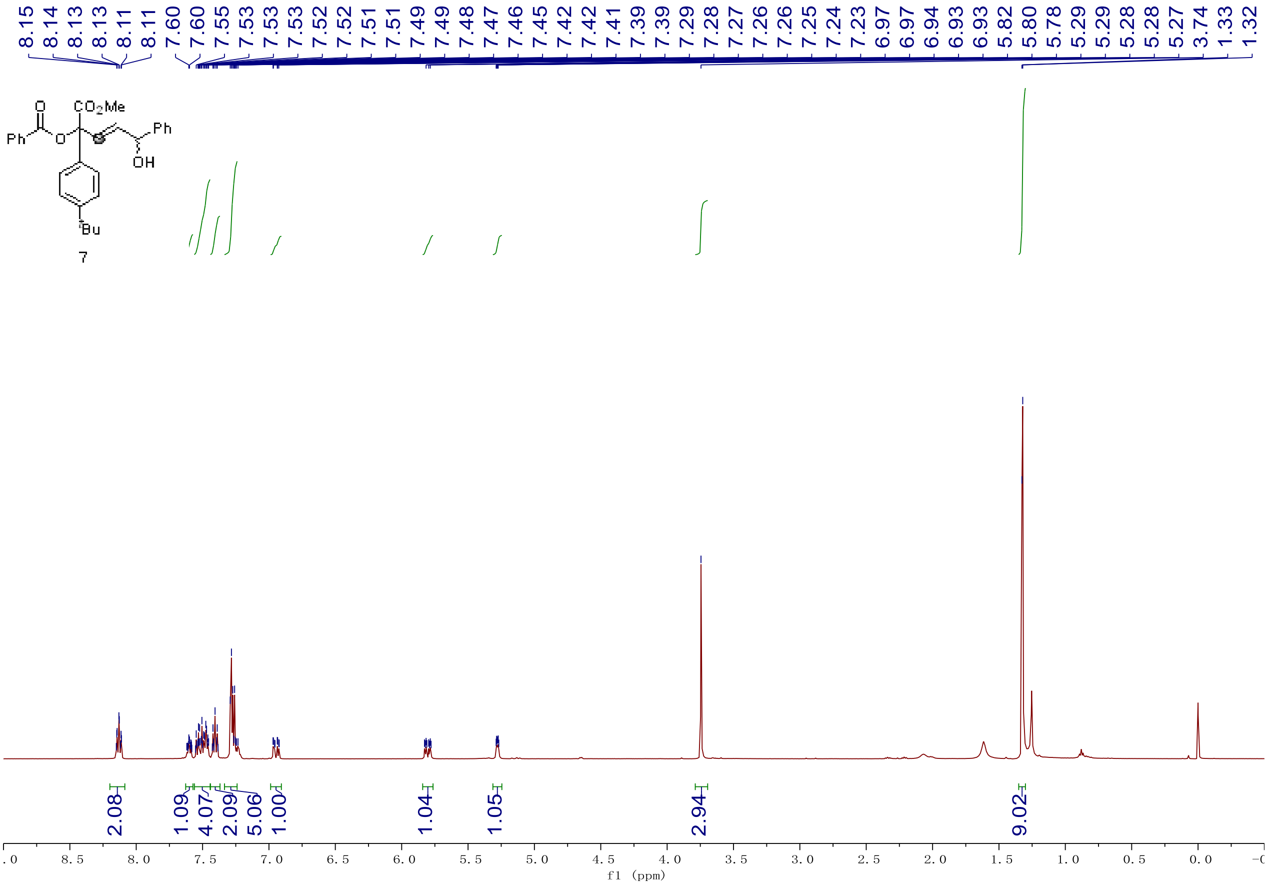

Supplementary Figure 129. ^{1}H NMR spectrum of **7.**

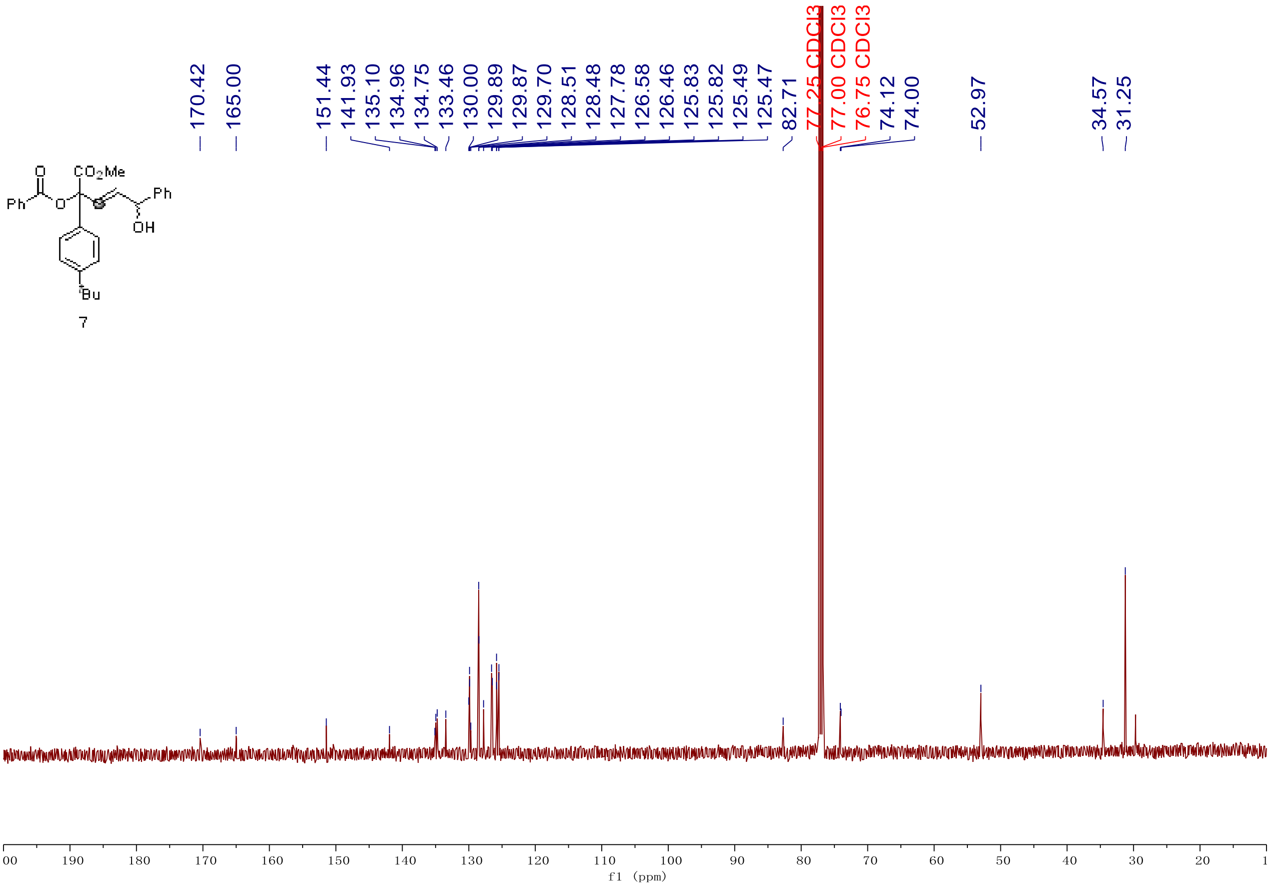

Supplementary Figure 130. ^{13}C NMR spectrum of **7**.

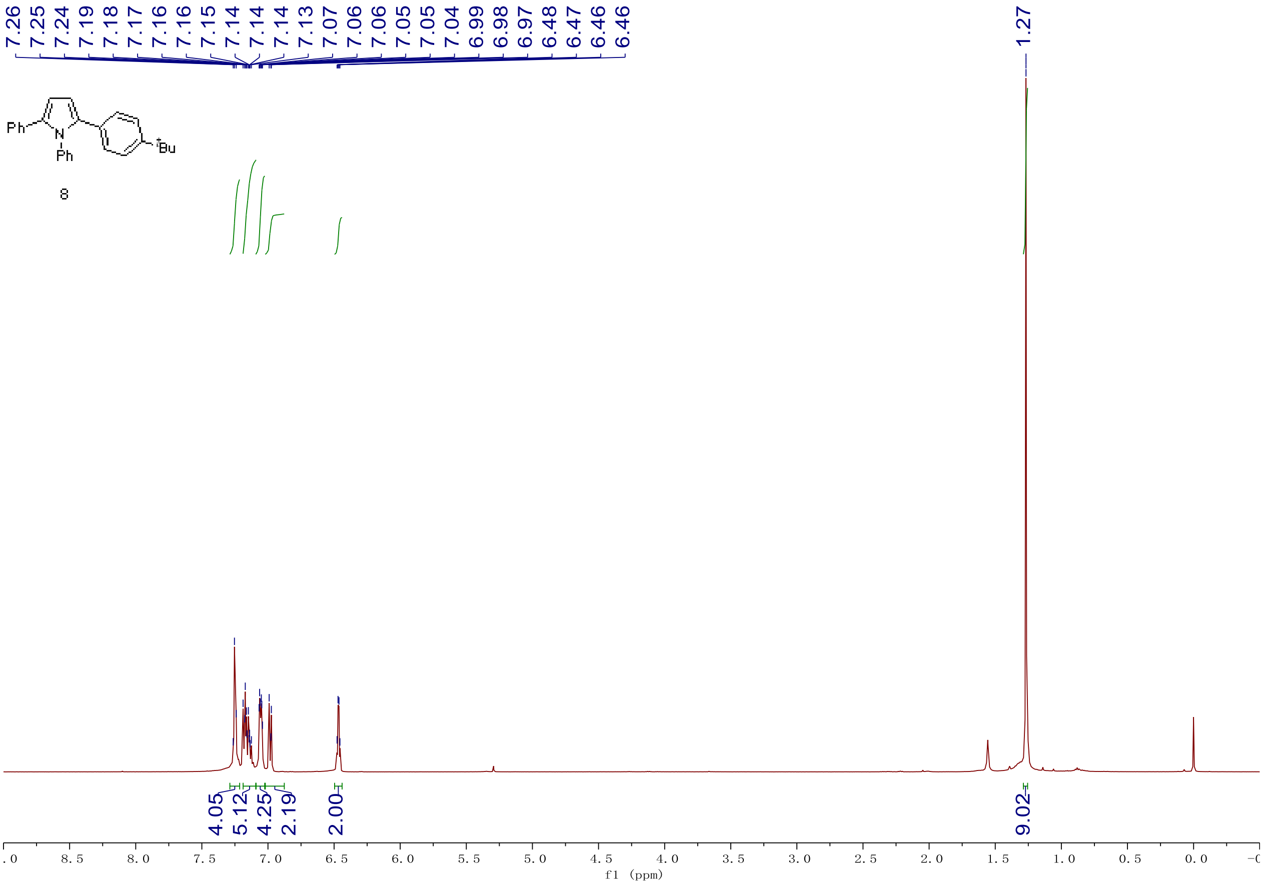

Supplementary Figure 131. ^{1}H NMR spectrum of **8**.

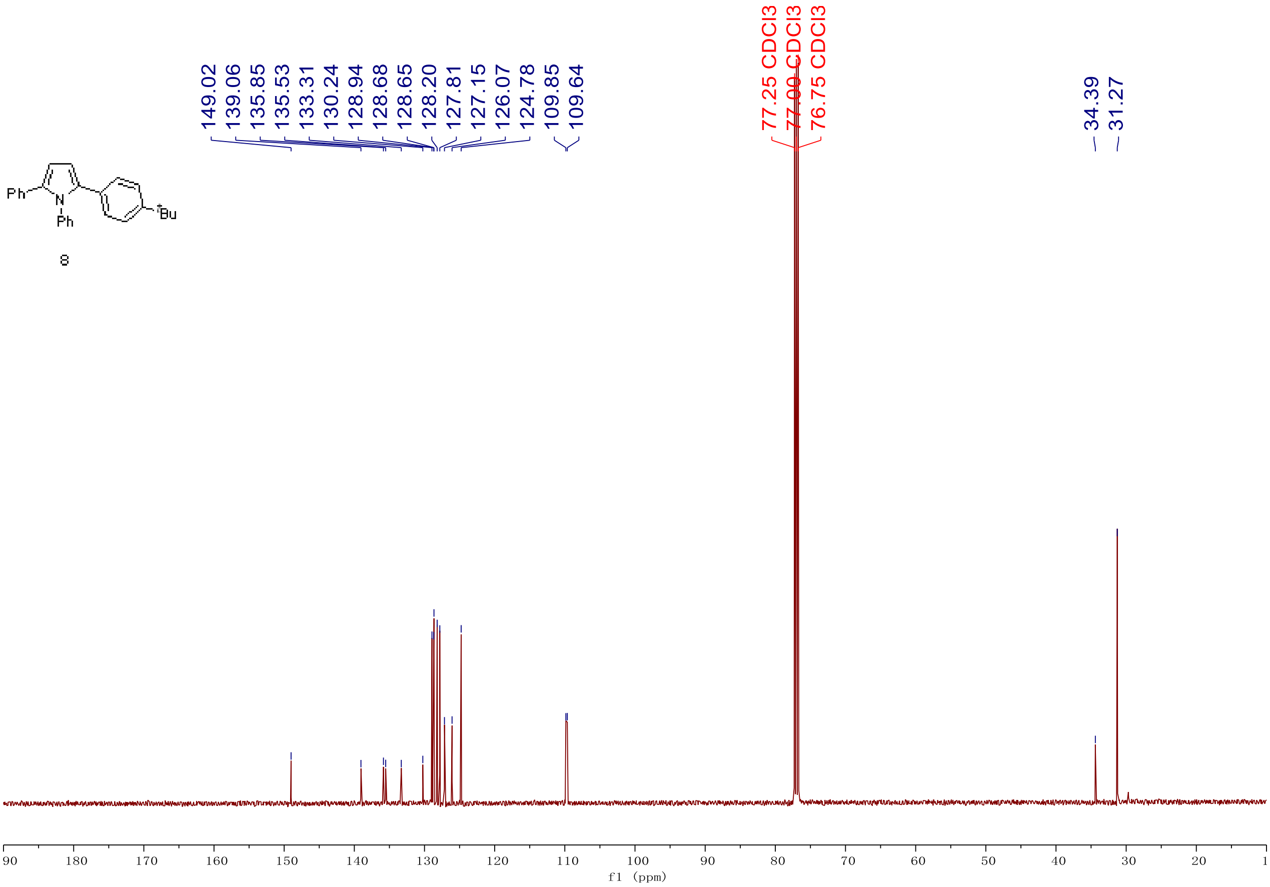

Supplementary Figure 132. ^{13}C NMR spectrum of **8.**

Supplementary Figure 133. X-ray crystal structure of **3a**.

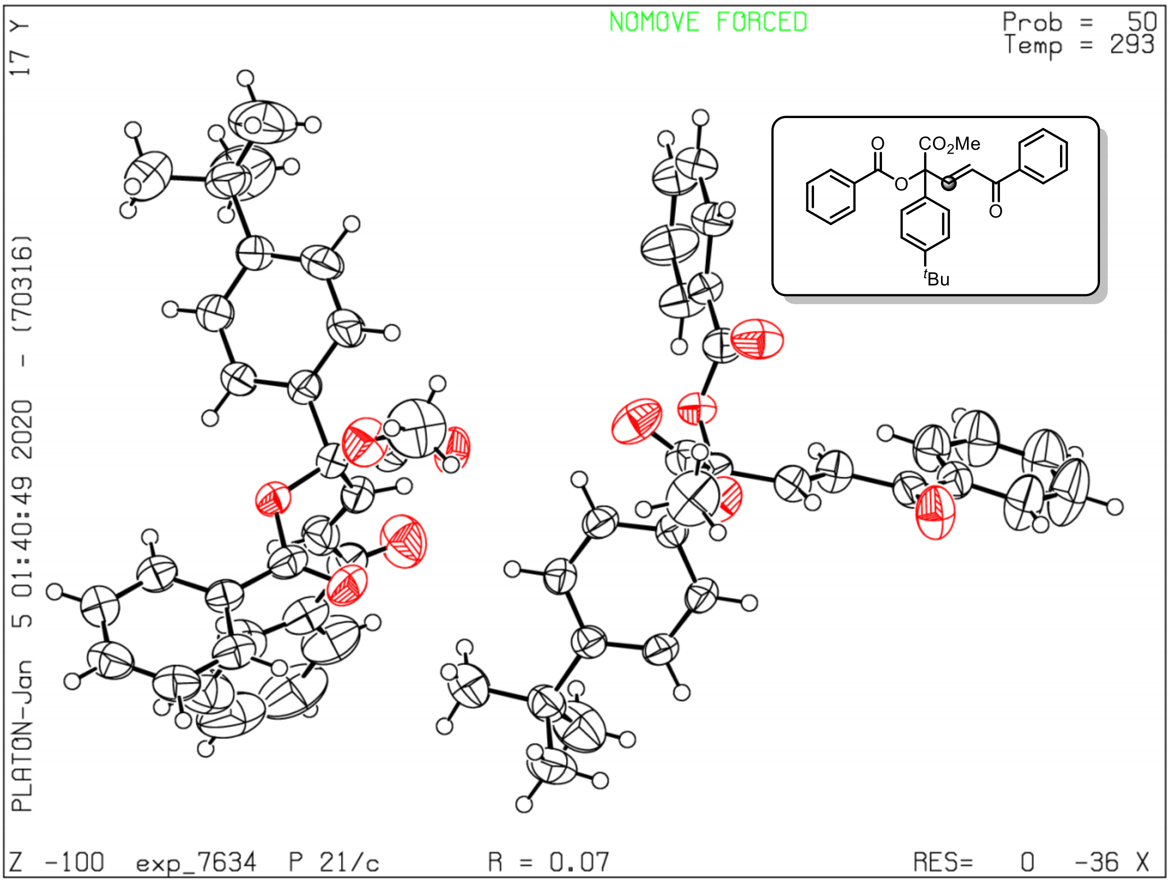

Supplementary References

1.Howard, J. L., Sagatov, Y., Repusseau, L., Schotten, C. & Browne, D. L. Controlling reactivity through liquid assisted grinding: the curious case of mechanochemical fluorination. *Green Chem.* **19,** 2798-2802 (2017).

2.Wasa, M., Liu, R. Y., Roche, S. P. & Jacobsen, E. N. Asymmetric Mannich Synthesis of α-Amino Esters by Anion-Binding Catalysis. *J. Am. Chem. Soc.* **136,** 12872-12875 (2014).

3.Keipour, H. & Ollevier, T. Iron-Catalyzed Carbene Insertion Reactions of α-Diazoesters into Si–H Bonds. *Org. Lett.* **19,** 5736-5739 (2017).

4.Santi, M. et al. Metal-Free Tandem Rearrangement/Lactonization: Access to 3,3-Disubstituted Benzofuran-2-(3H)-ones. *Angew. Chem. Int. Edit.* **58,** 7861-7865 (2019).

5.Yeh, T.-K. et al. Design, Synthesis, and Evaluation of Thiazolidine-2,4-dione Derivatives as a Novel Class of Glutaminase Inhibitors. *J. Med. Chem.* **60,** 5599-5612 (2017).

6.Misaki, T., Kawano, K. & Sugimura, T. Highly Z-Selective Asymmetric 1,4-Addition Reaction of 5H-Oxazol-4-ones with Alkynyl Carbonyl Compounds Catalyzed by Chiral Guanidines. *J. Am. Chem. Soc.* **133,** 5695-5697 (2011).

7.Ramtohul, Y. K. & Chartrand, A. Direct C-Arylation of β-Enamino Esters and Ketones with Arynes. *Org. Lett.* **9,** 1029-1032 (2007).
